# Pharmacokinetics of the CYP3A4 and CYP2B6 Inducer Carbamazepine and Its Drug–Drug Interaction Potential: A Physiologically Based Pharmacokinetic Modeling Approach

**DOI:** 10.3390/pharmaceutics13020270

**Published:** 2021-02-17

**Authors:** Laura Maria Fuhr, Fatima Zahra Marok, Nina Hanke, Dominik Selzer, Thorsten Lehr

**Affiliations:** Clinical Pharmacy, Saarland University, 66123 Saarbrücken, Germany; laura.fuhr@uni-saarland.de (L.M.F.); fatima.marok@uni-saarland.de (F.Z.M.); n.hanke@mx.uni-saarland.de (N.H.); dominik.selzer@uni-saarland.de (D.S.)

**Keywords:** physiologically based pharmacokinetic (PBPK) modeling, carbamazepine, carbamazepine-10,11-epoxide, drug–drug interactions (DDIs), cytochrome P450 3A4 (CYP3A4), cytochrome P450 2B6 (CYP2B6), induction

## Abstract

The anticonvulsant carbamazepine is frequently used in the long-term therapy of epilepsy and is a known substrate and inducer of cytochrome P450 (CYP) 3A4 and CYP2B6. Carbamazepine induces the metabolism of various drugs (including its own); on the other hand, its metabolism can be affected by various CYP inhibitors and inducers. The aim of this work was to develop a physiologically based pharmacokinetic (PBPK) parent−metabolite model of carbamazepine and its metabolite carbamazepine-10,11-epoxide, including carbamazepine autoinduction, to be applied for drug–drug interaction (DDI) prediction. The model was developed in PK-Sim, using a total of 92 plasma concentration−time profiles (dosing range 50–800 mg), as well as fractions excreted unchanged in urine measurements. The carbamazepine model applies metabolism by CYP3A4 and CYP2C8 to produce carbamazepine-10,11-epoxide, metabolism by CYP2B6 and UDP-glucuronosyltransferase (UGT) 2B7 and glomerular filtration. The carbamazepine-10,11-epoxide model applies metabolism by epoxide hydroxylase 1 (EPHX1) and glomerular filtration. Good DDI performance was demonstrated by the prediction of carbamazepine DDIs with alprazolam, bupropion, erythromycin, efavirenz and simvastatin, where 14/15 DDI AUC_last_ ratios and 11/15 DDI C_max_ ratios were within the prediction success limits proposed by Guest et al. The thoroughly evaluated model will be freely available in the Open Systems Pharmacology model repository.

## 1. Introduction

The anticonvulsant drug carbamazepine is known to induce multiple metabolizing enzymes. It is classified by the U.S. Food and Drug Administration (FDA) as a strong inducer (area under the plasma concentration−time curve (AUC) decrease of victim drug ≥ 80%) of cytochromes P450 (CYP) 3A4 and CYP2B6 [1]. Furthermore, carbamazepine itself is also metabolized by the respective enzymes [2], with metabolism via CYP3A4 to the pharmacologically active metabolite carbamazepine-10,11-epoxide as one of the main routes of elimination [3]. As a result, carbamazepine induces its own—as well as other drugs’—metabolism during multiple dose administration. Additionally, carbamazepine plasma levels can also be affected by enzyme inhibitors and inducers. Therefore, the coadministration of carbamazepine with other drugs, i.e., sensitive CYP3A4 or CYP2B6 substrates or perpetrators, can result in complex interaction patterns. Elevated carbamazepine plasma concentrations, caused by CYP3A4 inhibition, as well as elevated carbamazepine-10,11-epoxide plasma concentrations, caused by CYP3A4 induction, are associated with carbamazepine-related adverse events, including nausea, vomiting, drowsiness or mental confusion [4,5,6]. Additionally, as a strong enzyme inducer, carbamazepine significantly reduces plasma concentrations of coadministered victim drugs. For the CYP3A4 substrate simvastatin and the CYP2B6 substrate bupropion, AUC decreases of 82% and 90% were observed if coadministered with carbamazepine [7,8], risking loss of efficacy of those compounds.

As CYP3A4 is highly expressed in liver and intestine and metabolizes up to 50% of all marketed drugs independent of the drug class [9], interactions with a broad spectrum of substances are possible, for example with antiviral drugs (efavirenz) or antibiotics (erythromycin). The drug–drug interaction (DDI) with efavirenz illustrates the complexity of carbamazepine DDIs, as both compounds are CYP3A4 and CYP2B6 substrates as well as inducers and therefore, mutually induce each other’s metabolism [10].

As carbamazepine plays a crucial role in the treatment of epilepsy (recommended as first line treatment option by Cochrane [11] and included in the World Health Organization (WHO) Model List of Essential Medicines [12]), surveillance and examination of its DDI potential is important to ensure a safe drug therapy. The FDA recommends the use of carbamazepine as CYP3A4 and CYP2B6 inducer in clinical drug–drug interaction studies and it was applied in recent clinical trials, for example, with etonorgestrel, basimglurant or bitopertin [13]. In this case, the physiologically based pharmacokinetic (PBPK) modeling technique can come in as a helpful tool. With PBPK modeling, the pharmacokinetics (PK) of carbamazepine can be quantitatively described and the model can be coupled with other PBPK models to dynamically describe and predict DDIs. This modeling technique is recognized and recommended by the regulatory agencies FDA and the European Medicines Agency (EMA) [14,15]. More than 60% of PBPK models submitted to the FDA investigate DDIs. Several published studies investigate the DDI potential of carbamazepine using a PBPK modeling approach [13,16,17,18,19]. While carbamazepine is typically used as an inducer to investigate the interaction with a substrate, our study aims to provide a comprehensive overview on the pharmacokinetics of carbamazepine and its DDIs, investigating carbamazepine not only as inducer but also as victim drug.

Hence, the aims of the current study were (1) to develop a parent−metabolite PBPK model of carbamazepine and its main metabolite carbamazepine-10,11-epoxide, with implementation of carbamazepine autoinduction and (2) to apply the developed model for DDI predictions with carbamazepine as a perpetrator and victim drug.

The thoroughly evaluated model will be publicly available in the Open Systems Pharmacology (OSP) repository and can be applied to investigate and predict CYP3A4 and CYP2B6 DDIs. The Supplementary Materials to this paper will serve as a reference manual with detailed documentation of the model development and performance.

## 2. Materials and Methods

### 2.1. Software

The PBPK model was developed with PK-Sim and MoBi (Open Systems Pharmacology Suite 9.1, released under the GNU General Public License version 2 (GPLv2) license by the Open Systems Pharmacology community, www.open-systems-pharmacology.org, 2020). Parameter optimization (Monte-Carlo and Levenberg-Marquardt algorithms) and sensitivity analysis were performed with PK-Sim. Clinical study data from literature were digitized with Engauge Digitizer Version 10.12 (M. Mitchell [20], 2019) according to guidelines by Wojtyniak et al. [21]. Pharmacokinetic parameters were calculated and plots were created with R 3.6.2 (The R Foundation for Statistical Computing, Vienna, Austria, 2019).

### 2.2. Clinical Data

Plasma and saliva concentration−time profiles and fraction excreted (fe) unchanged in urine measurements of carbamazepine and carbamazepine-10,11-epoxide were collected and digitized from published clinical studies. Studies were selected to cover the administration of carbamazepine (1) over a broad dosing range, (2) in single- and multiple-dosing regimens and (3) for different carbamazepine formulations. Clinical studies investigating the oral administration of the metabolite carbamazepine-10,11-epoxide were also included. Studies were preferred if they were conducted with healthy participants without comedication and if frequent as well as late sampling data was provided.

The digitized clinical studies were split into a model building (training) dataset and a model evaluation (test) dataset. The studies for the training dataset were selected to inform the implemented pharmacokinetic processes by covering a broad dosing range, single- and multiple-dose studies, the application of different carbamazepine formulations, and information on saliva concentrations and urinary excretion of carbamazepine as well as plasma concentrations and urinary excretion of the metabolite carbamazepine-10,11-epoxide. An overview of all utilized clinical studies and their assignment to training or test dataset is documented in Table S1 of the Supplementary Materials.

### 2.3. Model Building

The modeling process was initiated with a literature search for physicochemical parameters and information on absorption, distribution, metabolism and excretion (ADME) processes. During model development, different reported parameter values and the impact of different ADME processes were tested. An overview of the ADME processes of carbamazepine is provided in the Supplementary Materials and corresponding literature parameters are listed in Table 1.

For simulations, virtual mean individuals were generated based on age, sex, ethnicity, body weight, and height as reported in the respective study protocols. If no information was provided, a 30-year-old, male, European individual with mean body weight and height characteristics from the PK-Sim population database was created. The PK-Sim expression database [22] was used to define the relative expression of relevant metabolizing enzymes in the different organs of the body. Model parameters that could not be described using information from literature were estimated by fitting the model to the observed data of the whole training dataset.

**Figure 1:**
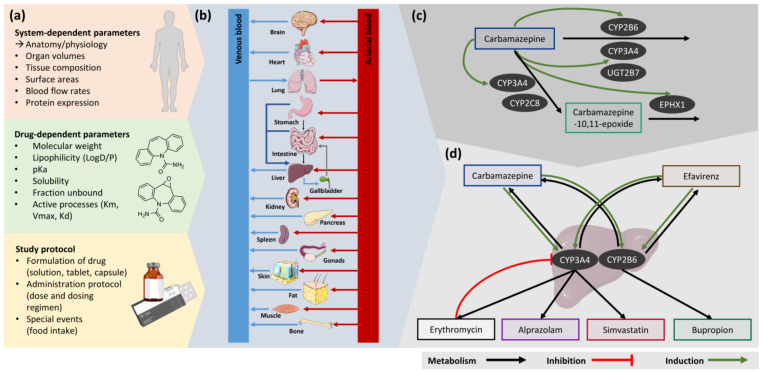
Schematic representation of the PBPK modeling workflow. (**a**) PBPK modeling requires system- and drug-dependent parameters, describing the anatomical and physiological characteristics of the individual and the properties of the simulated compound, respectively. Information on the study protocol of the described clinical study is relevant as well, e.g., formulation and administration of the simulated compound. (**b**) The PBPK model consists of multiple compartments, representing organs of the body, which are connected via the arterial and venous blood flows. (**c**) The final structure of the carbamazepine parent−metabolite PBPK model. (**d**) Overview of the modeled DDIs. Drawings by Servier, licensed under CC BY 3.0 [34]. CYP: cytochrome P450, EPHX1: epoxide hydroxylase 1, K_d_: dissociation constant, K_m_: Michaelis−Menten constant, pK_a_: acid dissociation constant, UGT: UDP-glucuronosyltransferase, V_max_: maximum velocity.

An overview of essential parameters needed to build a PBPK model, the whole-body PBPK model structure and implemented elimination processes are illustrated in Figure 1.

Elimination processes for carbamazepine include (1) metabolism by CYP3A4 and CYP2C8 to carbamazepine-10,11-epoxide, (2) metabolism by CYP3A4, CYP2B6 and UGT2B7 as well as hepatic clearance to cover further metabolic processes, (3) autoinduction of CYP3A4 and CYP2B6 and (4) passive glomerular filtration with tubular reabsorption [23]. The carbamazepine-10,11-epoxide metabolite model includes (1) metabolism by epoxide hydroxylase 1 (EPHX1) [24,25] and (2) renal elimination via passive glomerular filtration with tubular reabsorption.

Development of the parent−metabolite PBPK model was accomplished in a stepwise procedure. First, a model for the metabolite carbamazepine-10,11-epoxide was developed, based on three clinical studies that administered carbamazepine-10,11-epoxide. Metabolism by EPHX1 was implemented as a first-order clearance process according to Equation (1):

(1) $$v=\left[E\right]{*\mathrm{CL}}_{\mathrm{spec}}*S$$

where [E] = enzyme concentration, CL_spec_ = specific enzymatic clearance and S = substrate amount.

Passive glomerular filtration with reabsorption was described using a glomerular filtration rate (GFR) fraction < 1. The metabolite carbamazepine-10,11-epoxide model was subsequently combined with the parent carbamazepine model and the implemented parameter values were refined during development of the parent−metabolite model using the whole training dataset.

Metabolic pathways of carbamazepine were implemented using Michaelis−Menten kinetics, according to Equation (2):

(2) $$v=\;\frac{\left[E\right]{*k}_{\mathrm{cat}}*\left[S\right]}{K_{m}+\left[S\right]}$$

where [E] = enzyme concentration, k_cat_ = catalytic rate constant, [S] = substrate concentration and K_m_ = Michaelis−Menten constant.

Induction of CYP3A4, CYP2B6 and EPHX1 was implemented using a maximum effect (E_max_) model, as described in the Supplementary Materials Section 1.5. To inform the optimization of CYP3A4 induction, the carbamazepine-alprazolam DDI study was added to the training dataset. Renal clearance of carbamazepine, consisting of passive glomerular filtration with tubular reabsorption, was modeled using an estimated GFR fraction < 1.

Oral dosage forms of carbamazepine in the modeled clinical studies include solutions, suspensions, immediate release tablets and extended release tablets or capsules. To simulate solutions and suspensions, carbamazepine was modeled as a dissolved drug. The dissolution kinetics of the other formulations were described using Weibull functions. Different parameters were estimated for fasted or fed state, as Levy et al. and McLean et al. observed an increased carbamazepine absorption for ingestion of different carbamazepine formulations with food [26,27].

### 2.4. PBPK Model Evaluation

Model performance was evaluated (1) by comparing the predicted plasma concentration−time profiles to observed profiles and (2) by comparing predicted plasma concentration values to the corresponding observed values in goodness-of-fit plots, as well as (3) by comparing predicted with observed area under the plasma concentration−time curve (AUC) and maximum plasma concentration (C_max_) values. AUC values were calculated from the time of drug administration to the time of the last concentration measurement (AUC_last_). Predictions were considered successful if they did not deviate more than 2-fold from observed values.

For a quantitative description of the model performance, the mean relative deviation (MRD) of predicted plasma concentrations and the geometric mean fold error (GMFE) of predicted AUC_last_ and C_max_ values were calculated as described in the Supplementary Materials. We considered MRD and GMFE values ≤ 2 as adequate model performance metrics.

### 2.5. DDI Modeling

In addition to the previously described methods for PBPK model evaluation, the carbamazepine model was challenged by prediction of DDIs, with carbamazepine as CYP3A4 and CYP2B6 victim or perpetrator drug.

Clinical DDI studies with erythromycin, alprazolam, simvastatin, bupropion and efavirenz were available and used for DDI modeling. The previously developed PBPK models of erythromycin, alprazolam and efavirenz were downloaded from the OSP repository on GitHub (https://github.com/Open-Systems-Pharmacology [28,29,30]). The bupropion [31] and simvastatin [32] models were developed in our working group.

The parameters describing the induction of CYP3A4 and CYP2B6 by carbamazepine were already introduced during carbamazepine model building, as the compound induces its own metabolism. The mathematical implementation of the induction processes is described in Section 1.5 in the Supplementary Materials. The carbamazepine-alprazolam DDI study was used in the training dataset to inform the parametrization of the carbamazepine CYP3A4 induction. All other DDIs were purely predictive.

The DDI performance of all models except the efavirenz model (CYP2B6) was evaluated previously [28,29,30,31,32]. Therefore, all relevant interaction parameters were already implemented in the models and adopted in this project. The mathematical implementation of (1) the mechanism-based CYP3A4 inhibition by erythromycin, (2) the induction of CYP3A4 by efavirenz, (3) the induction of CYP2B6 by efavirenz and (4) the competitive inhibition of CYP3A4 by simvastatin are described in the Supplementary Materials. The drug-dependent parameters and interaction parameters of the previously developed models applied for carbamazepine DDI predictions are reproduced in Tables S6, S12, S15, S18 and S21 in the Supplementary Materials.

The performance of the efavirenz model as a CYP2B6 substrate and inducer was evaluated prior to DDI modeling with carbamazepine, using bupropion and rifampicin as CYP2B6 substrate and inducer, respectively. Based on this evaluation, the efavirenz model parameters were readjusted, which is further described in the Supplementary Materials.

### 2.6. DDI Model Evaluation

The DDI performance was assessed by comparison of predicted to observed victim drug plasma concentration−time profiles without and with coadministration of the perpetrator drug. Additionally, predicted DDI AUC_last_ ratios (Equation (3)) and DDI C_max_ ratios (Equation (4)) were compared to the respective observed ratios.

(3) $${\mathrm{DDI}\;\mathrm{AUC}}_{\mathrm{last}}\;\mathrm{ratio}\;=\;\frac{{\mathrm{AUC}}_{\mathrm{last}}{}_{\;\mathrm{victim}\;\mathrm{drug}\;\mathrm{during}\;\mathrm{coadministration}}}{{\mathrm{AUC}}_{\mathrm{last}}{}_{\;\mathrm{victim}\;\mathrm{drug}\;\mathrm{alone}}}$$

(4) $${\mathrm{DDI}\;C}_{\max}\;\mathrm{ratio}\;=\;\frac{C_{\max}{}_{\;\mathrm{victim}\;\mathrm{drug}\;\mathrm{during}\;\mathrm{coadministration}}}{C_{\max}{}_{\;\mathrm{victim}\;\mathrm{drug}\;\mathrm{alone}}}$$

As stated by Guest et al. [33], allowing up to 2-fold deviation of predicted to observed DDI ratios is not appropriate to assess the success of DDI predictions. For observed DDI ratios of 1 (no interaction), the 2-fold deviation would allow predicted DDI ratios between 0.5 (induction) and 2 (weak to moderate inhibition), which could overstate the DDI performance for weak interactions. Therefore, the prediction success limits proposed by Guest et al. [33] were used to evaluate the DDI predictions, accepting 20% deviation for observed DDI ratios approaching 1.

For each DDI, GMFEs of the predicted DDI AUC_last_ ratios and DDI C_max_ ratios were calculated, as described in the Supplementary Materials.

## 3. Results

### 3.1. PBPK Model Building

The parent−metabolite PBPK model of carbamazepine and carbamazepine-10,11-epoxide was built and evaluated using 40 clinical studies of oral administration, covering a broad dosing range (50–800 mg), different formulations as well as single- and multiple-dose regimens. In three of the included studies, the metabolite carbamazepine-10,11-epoxide was administered. In total, 58 and 34 plasma concentration−time profiles, and 4 and 5 fraction excreted unchanged in urine profiles, of carbamazepine and carbamazepine-10,11-epoxide were used, respectively. Additionally, 3 saliva concentration−time profiles were available for carbamazepine. All utilized clinical studies are listed in Table S1 of the Supplementary Materials.

Metabolism of carbamazepine by CYP2C8, CYP2B6 and UGT2B7 was described using K_m_ and k_cat_ values from literature. Two metabolic processes by CYP3A4 were implemented, as carbamazepine is metabolized by CYP3A4 to carbamazepine-10,11-epoxide as well as to hydroxylated metabolites [35]. For both reactions, K_m_ values were taken from literature, while k_cat_ values were optimized.

A half-maximal effective concentration EC_50_ = 20.0 µM (mean value calculated from literature values) was applied to describe the CYP3A4 induction. Although literature values for CYP2B6 EC_50_ were reported, the same EC_50_ = 20.0 µM was applied to describe the CYP2B6 induction, assuming that induction of both enzymes by carbamazepine is mediated via activation of the same nuclear receptor (constitutive androstane receptor [CAR]) [36]. The associated E_max_ values were optimized. Induction of EPHX1 was implemented as well, based on reports of an increase in carbamazepine-10,11-epoxide clearance during chronic carbamazepine treatment [37]. As no information on EC_50_ or E_max_ for EPHX1 was available, EC_50_ = 20.0 µM was used, assuming induction via activation of CAR as well, and E_max_ was optimized.

All implemented metabolic processes are summarized in Figure 1c. Drug-dependent parameters of carbamazepine and carbamazepine-10,11-epoxide are listed in Table 1, details on the distribution and localization of the implemented enzymes are provided in Table S45 of the Supplementary Materials.

**Figure 2:**
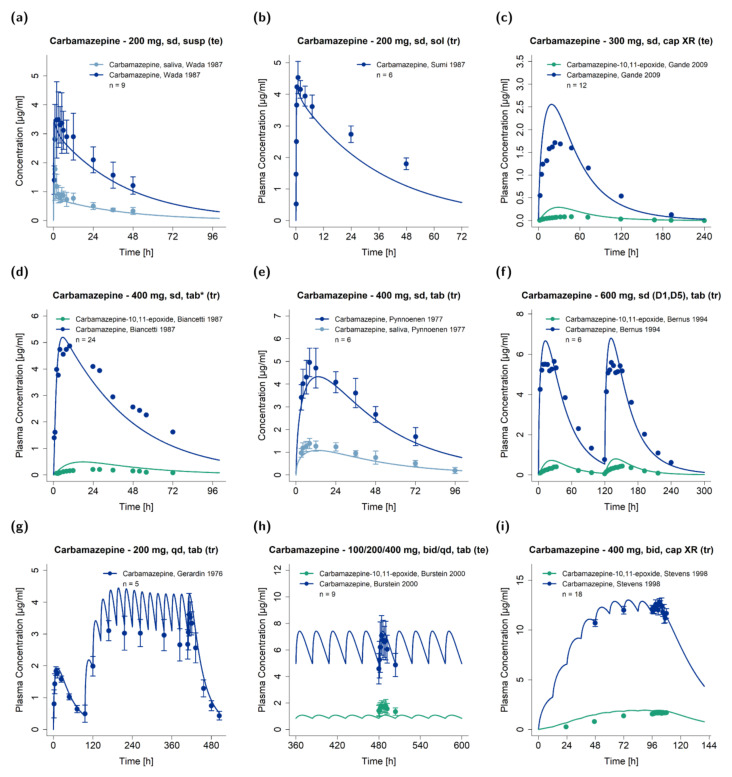
Model predictions of carbamazepine (dark blue: plasma, light blue: saliva) and carbamazepine-10,11-epoxide (green) concentration−time profiles of exemplary studies after (**a**–**e**) single- and (**f**–**i**) multiple-dose administration of different carbamazepine formulations [70,71,72,73,74,75,76,77,78] in comparison to observed data. Observed data are shown as dots ± SD (if available), simulations are shown as solid lines. Detailed information about the study protocols and model simulations of all clinical studies used to evaluate the carbamazepine model performance are provided in the Supplementary Materials. bid: twice daily, cap: capsule, D: day, n: number of subjects, qd: once daily, sd: single dose, sol: solution, susp: suspension, tab: tablet, tab*: tablet with concomitant food intake, te: test dataset, tr: training dataset, XR: extended release.

Figure 2 shows exemplary predictions of plasma concentration−time profiles compared to observed clinical data. Predicted compared to observed plasma concentration−time profiles of all studies are shown in the Supplementary Materials on a linear and semi-logarithmic scale.

**Figure 3:**
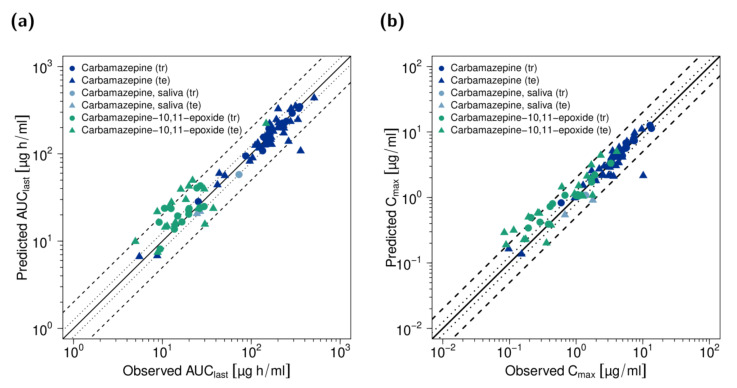
Performance of the carbamazepine parent−metabolite PBPK model. Predicted compared to observed (**a**) AUC_last_ values and (**b**) C_max_ values of carbamazepine and carbamazepine-10,11-epoxide of all analyzed studies. The line of identity is shown as solid line; 1.25-fold deviation is shown as dotted lines; 2-fold deviation is shown as dashed lines. AUC_last_: area under the plasma concentration−time curve from dosing to the last concentration measurement, C_max_: maximum plasma concentration, te: test dataset, tr: training dataset.

Plasma concentration goodness-of-fit plots along with MRD values for all analyzed studies are provided in the Supplementary Materials. In total, 94% and 69% of all carbamazepine and carbamazepine-10,11-epoxide plasma concentrations lie within the 2-fold acceptance limits, with overall MRD values of 1.38 and 1.76, respectively. Figure 3 shows predicted compared to observed AUC_last_ and C_max_ values. Low overall GMFEs of 1.20 and 1.57 for carbamazepine and carbamazepine-10,11-epoxide AUC_last_ values, as well as 1.24 and 1.65 for carbamazepine and carbamazepine-10,11-epoxide C_max_ values, respectively, demonstrate a good model performance. Table S4 lists all AUC_last_ and C_max_ values with the corresponding GMFEs.

Sensitivity analysis of a simulation of 400 mg three times daily orally administered carbamazepine with a parameter perturbation of 1000% and a sensitivity threshold of 0.5 revealed that the carbamazepine AUC is mainly sensitive to the carbamazepine fraction unbound in plasma (literature), while the carbamazepine-10,11-epoxide AUC is sensitive to carbamazepine-10,11-epoxide fraction unbound in plasma (literature), EPHX1 clearance of carbamazepine-10,11-epoxide (optimized), K_m_ and k_cat_ of carbamazepine CYP3A4 metabolism to carbamazepine-10,11-epoxide (literature and optimized, respectively) and carbamazepine EPHX1 E_max_ (optimized). The full quantitative results of the sensitivity analysis of all tested parameters are documented in the Supplementary Materials.

### 3.2. DDI Modeling

A total number of seven DDI studies, providing eight victim drug plasma concentration−time profiles and seven metabolite plasma concentration−time profiles, were used to evaluate the DDI performance of the carbamazepine parent−metabolite PBPK model. Those include studies with CYP3A4 victim drugs (alprazolam and simvastatin), a CYP3A4 inhibitor (erythromycin), a CYP2B6 victim drug (bupropion) as well as a CYP3A4 and CYP2B6 victim and perpetrator drug (efavirenz). The carbamazepine DDI network is illustrated in Figure 1d.

The DDI potential of carbamazepine as CYP3A4 substrate was assessed using three DDI studies with erythromycin as mechanism-based CYP3A4 inhibitor and substrate. In two studies a single dose of carbamazepine was applied after pretreatment with multiple doses of erythromycin [4,79]. In the third study, patients were pretreated with multiple doses of carbamazepine before coadministration of multiple doses of erythromycin [80], resulting in significant induction of CYP3A4 before the administration of the CYP3A4 inhibitor erythromycin.

The DDI potential of carbamazepine as CYP3A4 and CYP2B6 inducer was assessed using DDI studies with alprazolam and simvastatin as CYP3A4 and bupropion as CYP2B6 victim drugs. In those studies, pretreatment with multiple doses of carbamazepine was initiated to ensure significant enzyme induction before a single oral dose of the respective victim drugs was administered [7,8,81]. In the utilized efavirenz−carbamazepine DDI study, both compounds were administered in multiple oral dose regimens. The effect of drug coadministration was examined for each drug. Information on all utilized DDI studies along with detailed study protocols, demographics and references is provided in Tables S13, S16, S19, S22 and S24 in the Supplementary Materials.

**Figure 4:**
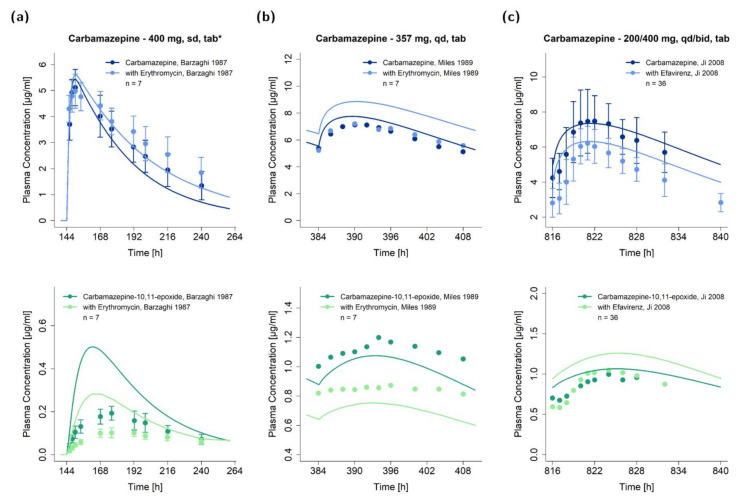
Victim drug plasma concentration−time profiles of the modeled drug–drug interactions with carbamazepine as victim drug (first row: carbamazepine, second row: metabolite carbamazepine-10,11-epoxide). Predictions of the victim drug plasma concentrations during the erythromycin−carbamazepine DDI (**a**) without and (**b**) with carbamazepine pretreatment [79,80] and (**c)** the efavirenz−carbamazepine DDI [8] are shown in comparison to observed data. Observed data are shown as dots ± SD (if available); predictions are shown as solid lines. Details on the study protocols and model simulations of all investigated DDI studies are provided in the Supplementary Materials. md: multiple dose, n: number of individuals, sd: single dose, tab: tablet, tab*: tablet with concomitant food intake.

**Figure 5:**
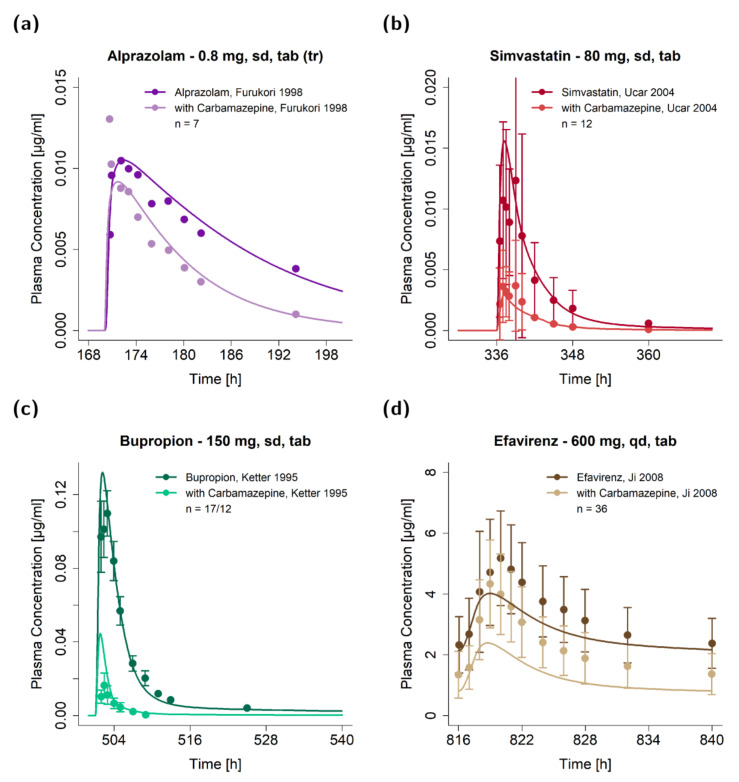
Victim drug plasma concentration−time profiles of the modeled drug–drug interactions with carbamazepine as perpetrator drug. Predictions of the victim drug plasma concentrations during the (**a**) carbamazepine−alprazolam DDI [81], **(b)** carbamazepine−simvastatin DDI [7] (**c**) carbamazepine−bupropion DDI [8] and (**d**) carbamazepine−efavirenz DDI [10] are shown in comparison to observed data. Observed data are shown as dots ± SD (if available); predictions are shown as solid lines. Details on the study protocols and model simulations of all investigated DDI studies are provided in the Supplementary Materials. md: multiple dose, n: number of individuals, sd: single dose, tab: tablet.

The DDI performance of the carbamazepine model with carbamazepine as victim or perpetrator drug is shown in Figure 4 and Figure 5, respectively. Plots show predicted victim drug plasma concentration−time profiles, with and without coadministration of the perpetrator drug, compared to observed data. Predicted compared to observed plasma concentration−time profiles of all DDI studies are also depicted in the Supplementary Materials on a semi-logarithmic and linear scale.

**Figure 6:**
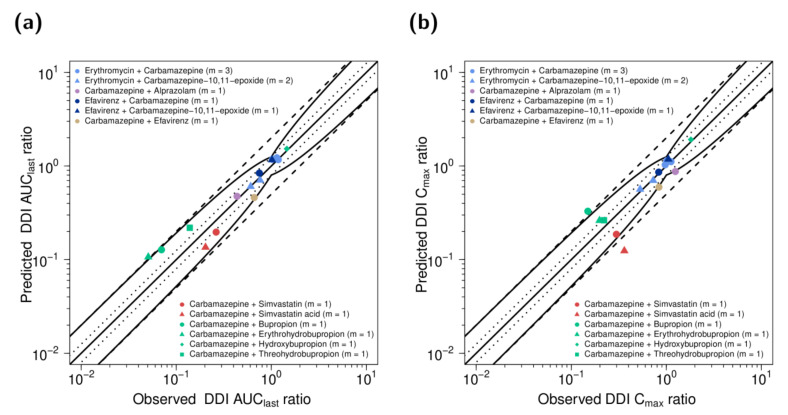
DDI performance of the carbamazepine parent−metabolite PBPK model. Predicted compared to observed (**a**) DDI AUC_last_ ratios and (**b**) DDI C_max_ ratios of all analyzed DDI studies. Dots represent the victim drug; triangles, diamonds and squares of the same color represent respective metabolites. The line of identity is shown as a straight solid line; the curved solid lines mark the prediction success limits proposed by Guest et al. [33]. A 1.25-fold deviation is shown as dotted lines; 2-fold deviation is shown as dashed lines. Details on the study protocols and all individual DDI AUC_last_ and DDI C_max_ ratios are provided in the Supplementary Materials. AUC_last_: area under the plasma concentration−time curve from dosing to the last concentration measurement, C_max_: maximum plasma concentration, DDI: drug–drug interaction, m: number of studies.

Predicted compared to observed DDI AUC_last_ and DDI C_max_ ratios are visualized in Figure 6 and are listed along with the corresponding GMFE values in Section 5 of the Supplementary Materials. 14/15 DDI AUC_last_ ratios and 11/15 DDI C_max_ ratios were within the prediction success limits proposed by Guest et al., with low overall GMFEs of 1.26 and 1.30 for all predicted DDI AUC_last_ and C_max_ ratios, respectively.

## 4. Discussion

In the presented study, a whole-body parent−metabolite PBPK model of carbamazepine and its main metabolite carbamazepine-10,11-epoxide was successfully established. The model adequately describes and predicts the plasma (and saliva) concentration−time profiles and the urinary excretion of carbamazepine and its main metabolite over a broad carbamazepine dosing range (50–800 mg) for oral administration of different formulations in single- and multiple-dose regimens, with and without concomitant food intake. The good model performance has been shown by a thorough evaluation of the PBPK model. Furthermore, the model was successfully applied for DDI simulations and predictions with erythromycin, alprazolam, simvastatin, bupropion and efavirenz.

The developed PBPK model includes a detailed description of carbamazepine metabolism via CYP3A4, CYP2C8, CYP2B6 and UGT2B7 and of carbamazepine-10,11-epoxide metabolism via EPHX1, including carbamazepine (auto-)induction of CYP3A4, CYP2B6 and EPHX1. Relevant ADME processes were predominantly parametrized using literature values. Only very few parameters were optimized, including lipophilicity, GFR fraction of parent and metabolite, CYP3A4 k_cat_ values, EPHX1 clearance as well as E_max_ values of the induction processes. The lipophilicity of a compound is used to calculate the organ permeabilities in PK-Sim. As logP is used as a surrogate input parameter for lipophilicity and might not fully describe the permeability properties of a compound, the lipophilicity value was optimized. GFR fraction was optimized to a value < 1, as passive reabsorption of carbamazepine along the renal tubule after glomerular filtration due to its high permeability is described in literature [23]. The CYP3A4 k_cat_ values were optimized, to correctly describe the plasma concentrations of the metabolite, while the EPHX1 clearance had to be optimized, as no information was available in the literature.

Predicted plasma concentration−time profiles of carbamazepine-10,11-epoxide showed discrepancies in comparison to observed data for some studies (Figure 2 and Figures S2–S5). The model tends to overpredict the data, while the observed plasma concentration−time profiles exhibit high variability of unknown origin. We reviewed the implemented formation, distribution and degradation processes of carbamazepine-10,11-epoxide, to understand the deviation between observations and model predictions. The formation of carbamazepine-10,11-epoxide is mediated by carbamazepine CYP3A4 and CYP2C8 metabolism, evaluated during DDI predictions. The good DDI performance of the parent−metabolite model indicates a reasonable implementation of the formation of carbamazepine-10,11-epoxide. As carbamazepine-10,11-epoxide is almost completely metabolized via EPHX1 [24,82], and high interindividual variability of EPHX1 activity was presumed [83], the variability observed in clinical study data might be caused by variability in EPHX1 metabolism. As no information on EPHX1 activity is provided in the reviewed studies, EPHX1 variability could not be reasonably reflected in the model. Furthermore, P-glycoprotein (P-gp) is discussed as carbamazepine-10,11-epoxide transporter [84]. Due to lack of conclusive evidence and studies parametrizing this transport, P-gp transport was not implemented. Overall, the carbamazepine-10,11-epoxide PBPK model was carefully developed, including studies of direct oral administration of carbamazepine-10,11-epoxide, and the model evaluation, including overall GMFE values of 1.57 and 1.65 for AUC_last_ and C_max_, for carbamazepine-10,11-epoxide yet indicates an adequate model performance.

The observed concentrations of carbamazepine in saliva are also well captured, demonstrating the good description of carbamazepine distribution. Correct description of saliva concentrations can be useful for further model applications, as saliva is used as a surrogate for plasma sampling in clinical practice (ratio saliva:plasma = 1:4, reflecting the free fraction of carbamazepine [48]).

With regard to drug transporters, P-gp is also discussed as a carbamazepine transporter, but without conclusive evidence in the literature [84,85,86,87,88]. P-gp would impact the carbamazepine pharmacokinetics by limiting the absorption from the gastrointestinal tract, hindering the penetration into the central nervous system and increasing the urinary excretion [89]. How strong this impact would be, with a drug as lipophilic as carbamazepine (logP of 1.45–2.77 [38,39,40]), is not clear. As there is conflicting information, and in the absence of in vitro studies of carbamazepine transport by P-gp in the literature, transport via P-gp was not implemented into the model. However, the model successfully describes the absorption of low (50 mg) and high (800 mg) carbamazepine doses as well as the urinary excretion of the unchanged drug.

As carbamazepine induces its own metabolism by activation of nuclear receptors resulting in an increased CYP3A4 and CYP2B6 expression, the pharmacokinetics of carbamazepine are quite complex. CYP enzyme induction is highly variable, with reported EC_50_ and E_max_ values ranging between 4.3–108 µM and 1.9–24.7 for CYP3A4 and 22–145 µM and 3.1–29.1 for CYP2B6 induction, respectively. The mean EC_50_ = 20 µM of all included CYP3A4 induction studies was selected for all implemented induction processes, assuming that induction of those enzymes is the result of carbamazepine activation of the CAR receptor [36]. The incorporated CYP3A4 induction was evaluated by prediction of multiple dose carbamazepine studies from the test dataset, as well as by prediction of the carbamazepine−simvastatin DDI, showing a good DDI performance with a predicted DDI AUC_last_ ratio of 0.20 compared to the observed ratio of 0.26.

The implementation of CYP3A4 metabolism of carbamazepine was further evaluated via the prediction of three erythromycin−carbamazepine DDI studies. For single dose carbamazepine administration (negligible CYP3A4 induction) the effect on carbamazepine is very well described with predicted compared to observed DDI AUC_last_ ratios of 1.22 and 1.14, respectively (study by Barzaghi et al. [79]) and 1.17 and 1.18, respectively (study by Wong et al. [4]). For multiple dose carbamazepine administration (considerable CYP3A4 induction) the effect on carbamazepine is also well described, with predicted compared to observed DDI AUC_last_ ratios of 1.18 and 1.03, respectively (study by Miles et al. [80]). In all cases, the effect of erythromycin on carbamazepine-10,11-epoxide plasma concentrations is well captured, with predicted compared to observed DDI AUC_last_ ratios of 0.60 and 0.61, respectively (study by Barzaghi et al. [79]) and 0.71 and 0.76, respectively (study by Miles et al. [80]). As carbamazepine-10,11-epoxide is mainly formed by CYP3A4 metabolism, it can be assumed that the fraction of carbamazepine metabolized via CYP3A4, and the effect of erythromycin on carbamazepine CYP3A4 metabolism, are accurately implemented in the applied models.

The E_max_ for CYP2B6 induction was identified during the carbamazepine model parameter identification and the description of CYP2B6 induction in the model was evaluated by prediction of the carbamazepine-bupropion and the efavirenz−carbamazepine DDIs. The simulated effect of carbamazepine on bupropion is underpredicted, showing predicted compared to observed DDI AUC_last_ ratios of 0.13 and 0.07, respectively. In this study, bupropion was administered to 12 patients with major affective disorders, who previously had chronic carbamazepine monotherapy and to 17 healthy individuals as control group. Carbamazepine doses for each patient as well as duration of their carbamazepine therapy were not provided in the study report. Furthermore, in the respective study, the pharmacokinetics of bupropion with and without carbamazepine coadministration were not investigated in a cross-over fashion, and therefore the results might be significantly influenced by the CYP2B6 genotypes of the two different study populations, because CYP2B6 polymorphism is a major determinant of bupropion pharmacokinetics.

The efavirenz−carbamazepine DDI was well described, with predicted compared to observed DDI AUC_last_ ratios of 0.84 and 0.75 for carbamazepine, and 1.16 and 1.06 for carbamazepine-10,11-epoxide, respectively, for the effect of efavirenz on carbamazepine. The interaction between efavirenz and carbamazepine is quite complex, as both compounds are substrates and well as inducers of CYP3A4 and CYP2B6, while CYP3A4 metabolism plays only a minor role for efavirenz [90]. Regarding the effect of carbamazepine on efavirenz, the effect is also well described with predicted compared to observed DDI AUC_last_ ratios of 0.46 and 0.66, respectively. The correct prediction of the impact of a perpetrator drug on the pharmacokinetics of a victim drug indicates that the perpetrator model adequately describes the drug concentrations at the sites of interaction and that the victim drug model simulates the right amount of drug eliminated via the affected pathway. The presented efavirenz−carbamazepine DDI example illustrates the value and power of PBPK DDI modeling, which allows us to dynamically compute the changes of perpetrator and victim drug plasma and tissue concentrations, as well as drug concentration-dependent induction of enzyme expression over time.

Several published studies investigate the pharmacokinetics of carbamazepine using PBPK modeling, (1) to predict the pharmacokinetics of carbamazepine in the pediatric population [91], (2) to describe gastrointestinal absorption of different carbamazepine formulations [92], (3) to investigate the DDI with levonorgestrel [93] and (4) to investigate the DDI performance of victim drug models with carbamazepine as enzyme inducer [15,16,17,18] using the default Simcyp parent−metabolite PBPK template model of carbamazepine and carbamazepine-10,11-epoxide [19].

In contrast to the previously published models, we used a large set of clinical data for model development (58 and 34 plasma profiles, as well as 4 and 5 fraction excreted in urine profiles, of carbamazepine and carbamazepine-10,11-epoxide, respectively). Additionally, our model provides an extensive overview on carbamazepine pharmacokinetics, including (1) the description of the metabolite carbamazepine-10,11-epoxide, (2) a detailed and mechanistic implementation of carbamazepine metabolism and autoinduction using in vitro literature parameter values, (3) the ability to describe different formulations applied in fasted or fed state and (4) thorough evaluation of metabolic and inductive processes in DDI simulations with CYP3A4 and CYP2B6 victim and perpetrator drugs. Carbamazepine is typically investigated as enzyme inducer. The presented study also investigated carbamazepine as a victim drug–a scenario which should not be neglected as illustrated in the complex interaction with efavirenz, where both compounds mutually influence their pharmacokinetics.

The presented carbamazepine parent−metabolite PBPK model can be applied to investigate and predict DDI scenarios with carbamazepine as CYP3A4 and CYP2B6 inducer and substrate. Such predictions can be used to support the design of clinical DDI studies of new drugs. As carbamazepine is prescribed as a long-term treatment of epilepsy, at times it might be coadministered with interacting drugs in clinical practice. In this case, the presented model could be applied to guide dose recommendations. As carbamazepine is a known inducer of further enzymes and transporters, e.g., CYP2C9 [1] or P-gp [94], future applications of the model could include the implementation of those inductions, as soon as PBPK models of sensitive substrates and the corresponding clinical DDI studies become available.

## 5. Conclusions

A comprehensive whole-body parent−metabolite PBPK model of carbamazepine and its main metabolite carbamazepine-10,11-epoxide was successfully established. The model includes metabolism of carbamazepine by CYP3A4, CYP2C8, CYP2B6 and UGT2B7; all Michaelis−Menten constants and most of the metabolic rate constants for these reactions were implemented using published in vitro values. In addition, it incorporates the (auto-)induction of CYP3A4, CYP2B6 and the carbamazepine-10,11-epoxide hydroxylase EPHX1 by carbamazepine. The model can be applied to predict plasma concentration−time profiles of carbamazepine and carbamazepine-10,11-epoxide administered in single- and multiple-dose regimens or different formulations. Furthermore, the presented model was thoroughly challenged and evaluated by prediction of DDIs in an extensive DDI network with five perpetrator and victim drugs and different study protocols. Noteworthy is the good prediction of the complex efavirenz−carbamazepine DDI with its mutual induction of CYP3A4 and CYP2B6. The good DDI performance is fully documented in the Supplementary Materials and the model is considered qualified for CYP3A4 and CYP2B6 DDI prediction. The modeling files will be shared with the scientific community in the Open Systems Pharmacology model repository (www.open-systems-pharmacology.org).

## Figures and Tables

**Table 1:** Drug-dependent parameters of carbamazepine and carbamazepine-10,11-epoxide.

Parameter	Unit	Model	Literature	Reference
**Carbamazepine**				
Molecular weight	g/mol	236.27 (Lit)	236.27	[38]
Lipophilicity	Log Units	2.00 (Fit)	1.45; 2.1; 2.45; 2.77	[38,39,40]
Solubility (FaHIF)	µg/mL	336 (Lit)	170; 283; 306; 336	[41,42,43,44]
Fraction unbound	%	25 (Lit)	21; 24; 25	[45,46,47,48]
K_m_ (CYP3A4) → CBZ-E	µM	248 (Lit)	119; 248; 442; 630	[2,49,50,51]
k_cat_ (CYP3A4) → CBZ-E	1/min	0.75 (Fit)	1.17; 1.7; 4.87; 5.3 ^b^	[2,49,50,51]
K_m_ (CYP2C8) → CBZ-E	µM	757 (Lit)	757	[50]
k_cat_ (CYP2C8) → CBZ-E	1/min	0.67 (Lit)	0.67 ^b^	[50]
K_m_ (CYP3A4)	µM	282 (Lit)	282	[35]
k_cat_ (CYP3A4)	1/min	0.20 (Fit)	0.16 ^b^	[35]
K_m_ (CYP2B6)	µM	420 (Lit)	420	[35]
k_cat_ (CYP2B6)	1/min	0.43 (Lit)	0.43 ^b^	[35]
K_m_ (UGT2B7)	µM	214 (Lit)	214	[52]
k_cat_ (UGT2B7)	1/min	9.53 × 10^−3^ (Lit)	9.53 × 10^−3^ ^c^	[52]
CL_hep_	1/min	0.02 (Fit)	-	-
GFR fraction	-	0.03 (Fit)	-	-
EC_50_ (CYP3A4)	µM	20.00 ^a^ (Lit)	4.3–137	[53,54,55,56,57,58,59,60]
E_max_ (CYP3A4)	-	6.00 (Fit)	1.9–23	[53,54,55,56,57,58,59,60]
EC_50_ (CYP2B6)	µM	20.00 ^a^ (Asm)	22–145	[60,61,62]
E_max_ (CYP2B6)	-	17.00 (Fit)	3.1–21.5	[60,61,62]
EC_50_ (EPHX1)	µM	20.00 ^a^ (Asm)	-	-
E_max_ (EPHX1)	-	3.25 (Fit)	-	-
Intestinal permeability	cm/s	4.3 × 10^−4^ (Lit)	4.3 × 10^−4^	[63]
Partition coefficients	-	Rodgers and Rowlands		[64,65]
Cellular permeabilities	cm/s	PK-Sim Standard		[66]
**Carbamazepine-10,11-epoxide**				
Molecular weight	g/mol	252.27 (Lit)	252.27	[67]
Lipophilicity	Log Units	1.00 (Fit)	1.58; 1.97	[67]
Solubility	µg/mL	1340 (Lit)	1340	[67]
Fraction unbound	%	51.8 (Lit)	46.8; 49.0; 47.0; 51.8; 50.0	[68]
CL_spec_ (EPHX1)	1/min	0.01 (Fit)	-	-
GFR fraction	-	0.21 (Fit)	-	-
Intestinal permeability	cm/s	5.0 × 10^−3^ (Fit)	-	-
Partition coefficients	-	Rodgers and Rowlands		[64,65]
Cellular permeabilities	cm/s	PK-Sim Standard		[66]

Asm: assumption, CBZ-E: carbamazepine-10,11-epoxide, CL_hep_: hepatic clearance, CL_spec_: specific clearance, CYP: cytochrome P450, GFR: glomerular filtration rate, EC_50_: half maximal effective concentration, E_max_: maximum effect, EPHX1: epoxide hydroxylase 1, FaHIF: fasted human intestinal fluid, Fit: fitted in parameter optimization, K_m_: Michaelis−Menten constant, k_cat_: catalytic rate constant, Lit: literature, UGT: UDP-glucuronosyltransferase, V_max_: maximum reaction velocity. ^a^ mean of literature values of EC_50_ (CYP3A4), assumed for all EC_50_ values. ^b^ k_cat_ values calculated within PK-Sim from V_max_/recombinant enzyme. ^c^ k_cat_ value calculated within PK-Sim from V_max_ = 0.79 pmol/min/microsomal protein, derived from in vitro assays in microsomes, assuming a microsomal UGT2B7 content of 82.9 pmol/mg microsomal protein [69]; k_cat_ = V_max_/UGT2B7 content microsomes.

## Data Availability

All modeling files including utilized clinical study data can be found here: https://github.com/Open-Systems-Pharmacology.

## Supplementary Materials

The following are available online at https://www.mdpi.com/1999-4923/13/2/270/s1: Comprehensive reference manual, providing documentation of the complete model performance assessment. Section 1: Physiologically based pharmacokinetic (PBPK) modeling. Section 2: Carbamazepine, Section 3: Efavirenz. Section 4: Efavirenz drug-gene interactions (DGI). Section 5: Carbamazepine drug-drug interactions (DDI). Section 6: Efavirenz drug-drug interactions (DDI). Section 7: System-dependent parameters.
