# Supplementary material for: Pharmacokinetics of the CYP3A4 and CYP2B6 Inducer Carbamazepine and Its Drug–Drug Interaction Potential: A Physiologically Based Pharmacokinetic Modeling Approach
Source: Pharmaceutics. 2021 Feb 17;13(2):270. doi: 10.3390/pharmaceutics13020270 (PMC7922031; doi:10.3390/pharmaceutics13020270)
Supplement: Supplementary file 1 [file pharmaceutics-13-00270-s001.pdf]

pharmaceutics

Pharmacokinetics of the CYP3A4 and CYP2B6 inducer carbamazepine and its drug-drug interaction potential: a physiologically based pharmacokinetic modeling approach

Supplementary Materials

Laura Maria Fuhr[1], Fatima Zahra Marok[1], Nina Hanke[1], Dominik Selzer[1], Thorsten Lehr[1]

[1]Clinical Pharmacy, Saarland University, Saarbrücken, Germany

Funding
This research was funded by the German Federal Ministry of Education and Research (BMBF), grant number 031L0161C ("OSMOSES"). We acknowledge support by the Deutsche Forschungsgemeinschaft (DFG, German Research Foundation) and Saarland University within the funding program Open Access Publishing.

Conflict of Interest
Thorsten Lehr has received research grants from the German Federal Ministry of Education and Research (grant 031L0161C). Laura Maria Fuhr, Fatima Zahra Marok, Nina Hanke and Dominik Selzer declare no conflict of interest.

Corresponding Author
Thorsten Lehr, PhD
Clinical Pharmacy, Saarland University
Campus C2 2
66123 Saarbrücken, Germany
ORCID: 0000 0002 8372 1465
Phone: +49 681 302 70255
Email: thorsten.lehr@mx.uni-saarland.de

Contents

1 Physiologically based pharmacokinetic (PBPK) modeling 5
1.1 PBPK model building . . . 5
1.2 Virtual individuals . . . 5
1.3 PBPK model evaluation . . . 6
1.4 Sensitivity analysis . . . 6
1.5 Mathematical implementation of drug-drug interactions (DDIs) . . . 7

2 Carbamazepine 9
2.1 PBPK model building . . . 9
2.2 Carbamazepine and Carbamazepine-10,11-epoxide clinical studies . . . 11
2.3 Carbamazepine and Carbamazepine-10,11-epoxide drug-dependent parameters 14
2.4 Profiles . . . 16
2.4.1 Carbamazepine-10,11-epoxide model . . . 16
2.4.2 Carbamazepine parent-metabolite model . . . 18
2.5 Model evaluation . . . 31
2.5.1 Plasma concentration goodness-of-fit plots . . . 31
2.5.2 Mean relative deviation of predicted plasma concentrations . . . 34
2.5.3 AUC_{last} and C_{max} goodness-of-fit plots . . . 37
2.5.4 Geometric mean fold error of predicted AUC_{last} and C_{max} values . . . 39
2.5.5 Sensitivity analysis . . . 43

3 Efavirenz 45
3.1 PBPK model building . . . 45
3.2 Efavirenz clinical studies . . . 47
3.3 Efavirenz drug-dependent parameters . . . 49
3.4 Profiles . . . 51
3.5 Model evaluation . . . 59
3.5.1 Plasma concentration goodness-of-fit plots . . . 59
3.5.2 Mean relative deviation of predicted plasma concentrations . . . 60
3.5.3 AUC_{last} and C_{max} goodness-of-fit plots . . . 61
3.5.4 Geometric mean fold error of predicted AUC_{last} and C_{max} values . . . 62
3.5.5 Sensitivity analysis . . . 63

4 Efavirenz drug-gene interactions (DGI) 65
4.1 DGI modeling - general . . . 65
4.2 Efavirenz clinical DGI studies . . . 66
4.3 Profiles . . . 67
4.4 DGI AUC_{last} and C_{max} ratio goodness-of-fit plots . . . 69
4.5 Geometric mean fold error of predicted DGI AUC_{last} and C_{max} ratios . . . 70

5 Carbamazepine drug-drug interactions (DDI) 71
5.1 DDI modeling - general . . . 71
5.2 Erythromycin-carbamazepine DDI . . . 72
5.2.1 Erythromycin drug-dependent parameters . . . 73
5.2.2 Erythromycin-carbamazepine clinical DDI studies . . . 74
5.2.3 Profiles . . . 75
5.2.4 DDI AUC_{last} and C_{max} ratio goodness-of-fit plots . . . 77
5.2.5 Geometric mean fold error of predicted DDI AUC_{last} and C_{max} ratios . . . 78

5.3 Carbamazepine-alprazolam DDI . . . 79
5.3.1 Alprazolam drug-dependent parameters . . . 80
5.3.2 Carbamazepine-alprazolam clinical DDI studies . . . 81
5.3.3 Profiles . . . 82
5.3.4 DDI AUC_{last} and C_{max} ratio goodness-of-fit plots . . . 83
5.3.5 Geometric mean fold error of predicted DDI AUC_{last} and C_{max} ratios . . . 84
5.4 Carbamazepine-simvastatin DDI . . . 85
5.4.1 Simvastatin drug-dependent parameters . . . 86
5.4.2 Carbamazepine-simvastatin clinical DDI studies . . . 89
5.4.3 Profiles . . . 90
5.4.4 DDI AUC_{last} and C_{max} ratio goodness-of-fit plots . . . 91
5.4.5 Geometric mean fold error of predicted DDI AUC_{last} and C_{max} ratios . . . 92
5.5 Carbamazepine-bupropion DDI . . . 93
5.5.1 Bupropion drug-dependent parameters . . . 94
5.5.2 Carbamazepine-bupropion clinical DDI studies . . . 97
5.5.3 Profiles . . . 98
5.5.4 DDI AUC_{last} and C_{max} ratio goodness-of-fit plots . . . 100
5.5.5 Geometric mean fold error of predicted DDI AUC_{last} and C_{max} ratios . . . 101
5.6 Efavirenz-carbamazepine DDI . . . 102
5.6.1 Efavirenz-carbamazepine clinical DDI studies . . . 103
5.6.2 Profiles . . . 104
5.6.3 DDI AUC_{last} and C_{max} ratio goodness-of-fit plots . . . 106
5.6.4 Geometric mean fold error of predicted DDI AUC_{last} and C_{max} ratios . . . 107

6 Efavirenz drug-drug interactions (DDI) 108
6.1 DDI modeling - general . . . 108
6.2 Efavirenz-midazolam DDI . . . 109
6.2.1 Midazolam drug-dependent parameters . . . 110
6.2.2 Efavirenz-midazolam clinical DDI studies . . . 111
6.2.3 Profiles . . . 112
6.2.4 DDI AUC_{last} and C_{max} ratio goodness-of-fit plots . . . 114
6.2.5 Geometric mean fold error of predicted DDI AUC_{last} and C_{max} ratios . . . 115
6.3 Efavirenz-alfentanil DDI . . . 116
6.3.1 Alfentanil drug-dependent parameters . . . 117
6.3.2 Efavirenz-alfentanil clinical DDI studies . . . 118
6.3.3 Profiles . . . 119
6.3.4 DDI AUC_{last} and C_{max} ratio goodness-of-fit plots . . . 121
6.3.5 Geometric mean fold error of predicted DDI AUC_{last} and C_{max} ratios . . . 122
6.4 Efavirenz-bupropion DDI . . . 123
6.4.1 Efavirenz-bupropion clinical DDI studies . . . 124
6.4.2 Profiles . . . 125
6.4.3 DDI AUC_{last} and C_{max} ratio goodness-of-fit plots . . . 127
6.4.4 Geometric mean fold error of predicted DDI AUC_{last} and C_{max} ratios . . . 128
6.5 Rifampin-efavirenz DDI . . . 129
6.5.1 Rifampin drug-dependent parameters . . . 130
6.5.2 Rifampin-efavirenz clinical DDI studies . . . 131
6.5.3 Profiles . . . 132
6.5.4 DDI AUC_{last} and C_{max} ratio goodness-of-fit plots . . . 134
6.5.5 Geometric mean fold error of predicted DDI AUC_{last} and C_{max} ratios . . . 135
6.6 Efavirenz-voriconazole DDI . . . 136
6.6.1 Voriconazole drug-dependent parameters . . . 137
6.6.2 Efavirenz-voriconazole clinical DDI studies . . . 138

6.6.3 Profiles 139
6.6.4 DDI AUC_{last} and C_{max} ratio goodness-of-fit plots 141
6.6.5 Geometric mean fold error of predicted DDI AUC_{last} and C_{max} ratios . . . 142

7 System-dependent parameters **143**

Abbreviations **148**

1 Physiologically based pharmacokinetic (PBPK) modeling

1.1 PBPK model building

PBPK model building was initiated with a literature search to collect physicochemical parameters, information on absorption, distribution, metabolism and excretion (ADME) processes and clinical studies of intravenous and oral administration in single- and multiple-dose regimens. In addition to drug plasma concentration-time profiles, fraction excreted unchanged in urine or feces measurements and tissue or saliva concentrations should be integrated if available. The clinical study data were digitized and divided into a model building (training) dataset and a model evaluation (test) dataset. The studies for the training dataset were selected to include studies covering the whole published dosing range, single- and multiple-dose studies and information on tissue or saliva concentrations and urinary or fecal excretion, if available. Model input parameter values that could not be informed from literature were optimized by fitting the model to observed data of the whole training dataset.

1.2 Virtual individuals

Virtual mean individuals were generated for each study according to the reported age, weight, height, sex and ethnicity. If no information was provided, a default individual was created (30 years of age, male, European, mean weight and height characteristics from the PK-Sim population database). Enzymes, transporters and binding partners were incorporated according to current literature. The PK-Sim expression database [1] was used to define their relative expression in the different organs of the body. Details and references for the implementation of relevant metabolizing enzymes, transport proteins and protein binding partners are provided in Section 7.

1.3 PBPK model evaluation

Model performance was evaluated (1) by comparing the predicted plasma concentration-time profiles to observed profiles and (2) by comparing predicted plasma concentration values to the corresponding observed values in goodness-of- fit plots, as well as (3) by comparing predicted with observed area under the plasma concentration-time curve (AUC) and maximum plasma concentration (C_{max}) values. AUC values (predicted as well as observed) were calculated from the time of drug administration to the time of the last concentration measurement (AUC_{last}). For a quantitative description of the model performance, the mean relative deviation (MRD) of predicted plasma concentrations and the geometric mean fold error ($GMFE$) of predicted AUC_{last} and C_{max} values were calculated according to Equations S1 and S2. We considered MRD and GMFE values < 2 as an adequate model performance metrics.

$$MRD = 10^x, \text{ with } x = \sqrt{\frac{1}{m}\sum_{i=1}^{m}(\log_{10} c_{pred,i} - \log_{10} c_{obs,i})^2} \quad \text{(S1)}$$

where $c_{pred,i}$ = predicted plasma concentration, $c_{obs,i}$ = corresponding observed plasma concentration and m = number of observed values.

$$GMFE = 10^x, \text{ with } x = \frac{1}{n}\sum_{i=1}^{n}|\log_{10}(\frac{PK_{pred,i}}{PK_{obs,i}})| \quad \text{(S2)}$$

where $PK_{pred,i}$ = predicted AUC_{last} or C_{max} value, $PK_{obs,i}$ = corresponding observed AUC_{last} or C_{max} value and n = the number of studies.

1.4 Sensitivity analysis

Sensitivity of the final models to single parameter values (local sensitivity analysis) was measured as relative change of the AUC. Sensitivity analysis was carried out using a relative perturbation of 1000% (variation range 10.0, maximum number of 9 steps). Parameters were included into the analysis if they were optimized, if they are associated with optimized parameters or if they might have a strong impact due to calculation methods used in the model.

The sensitivity to a parameter value was calculated as the ratio of the relative change of the simulated AUC to the relative variation of the parameter around its value used in the final model according to Equation S3.

$$S = \frac{\Delta AUC}{AUC} \cdot \frac{p}{\Delta p} \quad \text{(S3)}$$

where S = sensitivity of the AUC to the examined model parameter, ΔAUC = change of the simulated AUC, AUC = simulated AUC with the original parameter value, Δp = change of the examined parameter value, p = original parameter value. A sensitivity of +1.0 signifies that a 10% increase of the examined parameter value causes a 10% increase of the simulated AUC.

1.5 Mathematical implementation of drug-drug interactions (DDIs)

Competitive inhibition

Competitive inhibitors compete with the substrate for binding to the active site of an enzyme or transporter. As binding of the inhibitor is reversible, the inhibition can be overcome by high substrate concentrations (concentration-dependency); The maximum reaction velocity (v_{max}) remains unaffected, while the Michaelis-Menten constant (K_m) is increased ($K_{m,app}$, Equation S4). The reaction velocity (v) during co-administration of substrate and competitive inhibitor is described by Equation S5 [2]:

$$K_{m,app} = K_m \cdot \left(1 + \frac{[I]}{K_i}\right) \tag{S4}$$

$$v = \frac{v_{max} \cdot [S]}{K_{m,app} + [S]} \tag{S5}$$

where $K_{m,app}$ = Michaelis-Menten constant in the presence of inhibitor, K_m = Michaelis-Menten constant, $[I]$ = free inhibitor concentration, K_i = dissociation constant of the inhibitor-enzyme or inhibitor-transporter complex, v = reaction velocity, v_{max} = maximum reaction velocity, $[S]$ = free substrate concentration.

Mechanism-based inactivation

Mechanism-based inactivation is an irreversible inhibition. The baseline activity of the protein is regained after clearance of the inactivator and de novo synthesis of the inactivated protein (time-dependency). The degradation rate constant (k_{deg}) of respective protein is increased ($k_{deg,app}$, Equation S6), while the synthesis rate (R_{syn}) remains unaffected. The enzyme or transporter turnover during administration of a mechanism-based inactivator is described by Equation S7. Mechanism-based inactivators are also competitive inhibitors. Therefore, the reaction velocity is calculated accodring to Equation S8, substituting K_m by $K_{m,app}$ [2]:

$$k_{deg,app} = k_{deg} + \left(\frac{k_{inact} \cdot [I]}{K_I + [I]}\right) \tag{S6}$$

$$\frac{dE(t)}{dt} = R_{syn} - k_{deg,app} \cdot E(t) \tag{S7}$$

$$v = \frac{v_{max} \cdot [S]}{K_{m,app} + [S]} = \frac{k_{cat} \cdot E(t) \cdot [S]}{K_{m,app} + [S]} \tag{S8}$$

where $k_{deg,app}$ = enzyme or transporter degradation rate constant in the presence of mechanism-based inactivator, k_{deg} = enzyme or transporter degradation rate constant, k_{inact} = maximum inactivation rate constant, $[I]$ = free inactivator concentration, K_I = concentration for half-maximal inactivation, $E(t)$ = enzyme or transporter concentration, R_{syn} = enzyme or transporter synthesis rate, v = reaction velocity, v_{max} = maximum reaction velocity, $[S]$ = free substrate concentration, $K_{m,app}$ = Michaelis-Menten constant in the presence of inactivator, k_{cat} = catalytic rate constant.

Induction

Induction of enzymes and transporters is mediated by the activation of nuclear receptors (constitutive androstane receptor (CAR) or pregnane X receptor (PXR)), increasing gene expression. The baseline activity of the protein is regained after clearance of the inducer and degradation of the induced protein (time-dependency). The enzyme or transporter synthesis rate (R_{syn}) is increased ($R_{syn,app}$, Equation S9), while its degradation rate constant (k_{deg}) remains unaffected. The enzyme or transporter turnover during administration of an inducer is described by S10 [2], the reaction velocity is described by Equation S11:

$$R_{syn,app} = R_{syn} \cdot \left(1 + \frac{E_{max} \cdot [Ind]}{EC_{50} + [Ind]}\right) \tag{S9}$$

$$\frac{dE(t)}{dt} = R_{syn,app} - k_{deg} \cdot E(t) \tag{S10}$$

$$v = \frac{v_{max} \cdot [S]}{K_m + [S]} = \frac{k_{cat} \cdot E(t) \cdot [S]}{K_m + [S]} \tag{S11}$$

where $R_{syn,app}$ = enzyme or transporter synthesis rate in the presence of inducer, R_{syn} = enzyme or transporter synthesis rate, E_{max} = maximal induction effect in vivo, $[Ind]$ = free inducer concentration, EC_{50} = concentration for half-maximal induction in vivo, $E(t)$ = enzyme or transporter concentration, k_{deg} = enzyme or transporter degradation rate constant, v = reaction velocity, v_{max} = maximum reaction velocity, $[S]$ = free substrate concentration, K_m = Michaelis-Menten constant, k_{cat} = catalytic rate constant.

2 Carbamazepine

2.1 PBPK model building

The anticonvulsive carbamazepine is used for the treatment of epilepsy and trigeminal neuralgia [3]. It is a BCS Class II drug of low solubility and high permeability. The drug is mainly cleared from the body by enzymatic degradation. About 20-40% of the administered carbamazepine is metabolized to carbamazepine-10,11-epoxide [4, 5]. Cytochrome P450 (CYP) 3A4 and CYP2C8 are the major enzymes catalyzing this conversion, while the contribution of CYP2C8 is between 20-40% [6]. 10% of the dose are metabolized to hydroxylated metabolites, mainly by CYP3A4 and CYP2B6 [7]. Less than 3% are excreted unchanged in the urine [8] and less than 1% are excreted unchanged in the bile [9]. Carbamazepine is a strong inducer (AUC decrease of a victim drug ≥ 80%) of CYP3A4 and CYP2B6 [10]. As a result, carbamazepine induces its own - as well as other drugs' - metabolism during multiple-dose administration. Carbamazepine-10,11-epoxide is almost completely converted to its trans-diol form via epoxide hydroxylase 1 (EPHX1) [5, 11] and it is assumed that carbamazepine also induces EPHX1 clearance after multiple-dose administration [4].

The parent-metabolite PBPK model of carbamazepine and carbamazepine-10,11-epoxide was established using 40 clinical studies of oral administration, covering a broad range of doses (50–800 mg) in single- and multiple-dose administration of different formulations. In three of the included studies, the metabolite carbamazepine-10,11-epoxide itself was orally administered. In total, the clinical studies provided 58 plasma concentration-time profiles, 3 saliva concentration-time profiles and 4 fraction excreted unchanged in urine measurements of carbamazepine, as well as 34 plasma concentration-time profiles (24 as metabolite, 10 as administered drug) and 5 fraction excreted unchanged in urine measurements of carbamazepine-10,11-epoxide. All clinical studies are listed in Table S1.

As elimination processes in the carbamazepine model (1) metabolism by CYP3A4 and CYP-2C8 to carbamazepine-10,11-epoxide, (2) metabolism by CYP3A4, CYP2B6 and UDP-glucuronosyltransferase (UGT) 2B7 as well as a hepatic clearance to cover further metabolic processes, (3) auto-induction of CYP3A4 and CYP2B6 and (4) passive glomerular filtration with tubular reabsorption [12] were implemented. The carbamazepine-10,11-epoxide metabolite model includes (1) metabolism by EPHX1 [11, 13] and (2) renal elimination via passive glomerular filtration. The metabolic processes implemented in the parent-metabolite PBPK model are summarized in Figure S1. The drug-dependent parameters of carbamazepine and carbamazepine-10,11-epoxide are listed in Table S2, details on the distribution and localization of the implemented enzymes are provided in Table S45.

The carbamazepine-10,11-epoxide PBPK model was developed first, based on the three clinical studies (10 plasma concentration-time profiles) that administered carbamazepine-10,11-epoxide. Metabolism by EPHX1 was implemented as a first-order clearance process, and passive glomerular filtration was described using a GFR fraction < 1. The metabolite carbamazepine-10,11-epoxide model was subsequently combined with the parent carbamazepine model and the parameter values were used as starting values for the establishment of the parent-metabolite model. Metabolic pathways of carbamazepine were implemented using Michaelis-Menten kinetics. All K_m and k_{cat} values were taken from literature, except for the two implemented CYP3A4 processes where k_{cat} was optimized. As CYP induction by carbamazepine is mediated via activation of the CAR [14], the same EC_{50} = 20.0 µmol/l was applied to describe the CYP3A4, CYP2B6 as well as the EPHX1 induction by carbamazepine. The associated E_{max} values were optimized. To inform the optimization of the CYP3A4 E_{max}, the carbamazepine-alprazolam DDI study was added to the training dataset. Renal elimination was described using an estimated GFR fraction < 1. Oral dosage forms of carbamazepine in the analyzed clinical studies include

solutions, suspensions, immediate release tablets and extended release tablets or capsules. To simulate solutions and suspensions, carbamazepine was modeled as dissolved drug. The dissolution kinetics of the other formulations were described using Weibull functions. Different Weibull functions were optimized for fasted or fed state, as an increased carbamazepine absorption was observed for ingestion with food [15, 16].

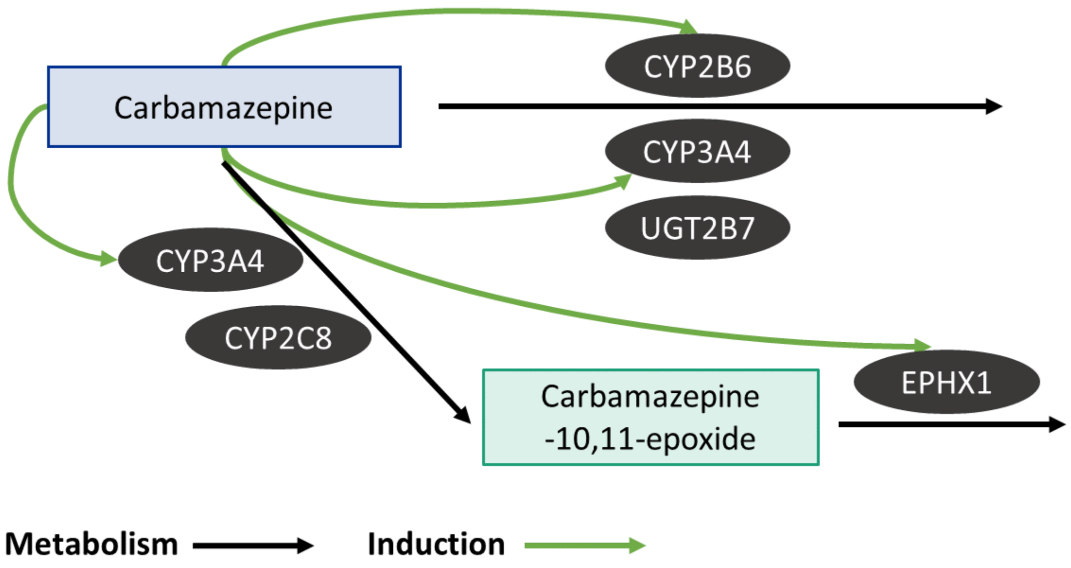

Figure S1: Metabolic pathways of carbamazepine. Carbamazepine is metabolized by CYP3A4 and CYP2C8 to its main metabolite carbamazepine-10,11-epoxide, which is then further metabolized via EPHX1. Other metabolic pathways of carbamazepine clearance are metabolism by CYP2B6 and CYP3A4 to 3-hydroxycarbamazepine and by UGT2B7 to glucuronide metabolites. Carbamazepine is an inducer of CYP3A4, CYP2B6 and EPHX1

Figures S2 and S3 show the good performance of the carbamazepine-10,11-epoxide model after oral application of carbamazepine-10,11-epoxide in linear and semi-logarithmic plots of predicted compared to observed plasma concentration-time profiles. Goodness-of-fit plots comparing all predicted to the corresponding observed plasma concentration values are presented in Figure S7 and correlation of predicted and observed AUC_{last} and C_{max} values is presented in Figure S10.

The precise performance of the carbamazepine parent-metabolite model is demonstrated in linear and semi-logarithmic plots of predicted compared to observed plasma and saliva concentration-time profiles in Figures S4 and S5. Predicted fraction excreted unchanged in urine profiles in comparison to observed values are presented in Figure S6. Goodness-of-fit plots for plasma concentrations and fractions excreted to urine values are presented in Figures S8 and S9, respectively. The correlation of predicted and observed AUC_{last} and C_{max} values is presented in Figure S11. MRD values of plasma concentration predictions for all studies are given in Table S3 . Predicted and observed AUC_{last} and C_{max} values and overall GMFE values of all studies are listed in Table S4.

2.2 Carbamazepine and Carbamazepine-10,11-epoxide clinical studies

Table S1: Clinical studies used for the development of the carbamazepine parent-metabolite PBPK model

Dose [mg]	Route	Dataset	n	Healthy [%]	Females [%]	Age[a] [years]	Weight[a] [kg]	Height[a] [cm]	CBZE measured	Reference
**Carbamazepine-10,11-epoxide**										
50	po (susp), sd	training	1	100	0	31	65	-	NA	Tomson 1983 [5]
50	po (susp), sd	test	1	100	0	27	63	-	NA	Tomson 1983 [5]
100	po (susp), sd	training	1	100	0	34	69	-	NA	Tomson 1983 [5]
100	po (susp), sd	test	1	100	100	50	53	-	NA	Tomson 1983 [5]
100	po (susp), sd	test	1	100	0	31	65	-	NA	Tomson 1983 [5]
150	po (sol), sd	test	6	-	33	25.7 (23-32)	56.2 (44-59)	-	NA	Sumi 1987 [17]
200	po (susp), sd	training	1	100	0	34	69	-	NA	Tomson 1983 [5]
200	po (susp), sd	test	1	100	100	50	53	-	NA	Tomson 1983 [5]
100	po (tab), sd	training	6	100	33	(20-43)	(55-75)	-	NA	Pisani 1990 [18]
100	po (tab), sd	test	6	100	33	(20-50)	(50-74)	-	NA	Pisani 1992 [19]
**Carbamazepine**										
100/10	po (susp), sd; iv (2 h)	test	1	100	0	43	76	-	no	Gerardin 1990 [20]
100/10	po (susp), sd; iv (2 h)	test	1	100	0	49	89	-	no	Gerardin 1990 [20]
50	po (sol), sd	training	1	100	0	29	-	-	no	Rawlins 1975 [21]
100	po (sol), sd	test	1	100	0	29	-	-	no	Rawlins 1975 [21]
200	po (susp), sd	test	1	100	100	29	62	-	no	Eichelbaum 1985 [4]
200	po (sol), sd	test	1	100	0	29	-	-	no	Rawlins 1975 [21]
200	po (sol), sd	training	6	-	33	25.7 (23-32)	56.2 (44-59)	-	no	Sumi 1987 [17]
200	po (susp), sd	test	1	100	100	50	53	-	no	Tomson 1983 [5]
200	po (susp), sd	test	9	100	0	-	69.1 (53-104)	-	no	Wada 1978 [22]
100	po (tab), sd	test	6	100	0	-	-	-	no	Gerardin 1976 [23]
200	po (tab), sd	test	12	100	0	29.15 (25-30)	67.5 (55-81)	-	yes	Bedada 2015 [24]
200	po (tab), sd	test	12	100	0	(28-32)	(58-76)	-	yes	Bedada 2016 [25]
200	po (tab), sd	test	1	0	0	57	80	-	yes	Eichelbaum 1975 [26]
200	po (tab), sd	test	1	0	0	50	80	-	yes	Eichelbaum 1975 [26]

-: not given, bid: twice daily, cap: capsule, D: day, iv: intravenous, NA: not applicable, po: oral, qd: once daily, sd: single dose, sol: solution, susp: suspension, tab: tablet, tid: three times daily, XR: extended release

[a] mean (range)

[b] 6 mg/kg

Table S1: Clinical studies used for the development of the carbamazepine parent-metabolite PBPK model *(continued)*

Dose [mg]	Route	Dataset	n	Healthy [%]	Females [%]	Age[a] [years]	Weight[a] [kg]	Height[a] [cm]	CBZE measured	Reference
200	po (tab), sd	test	6	100	0	-	-	-	no	Gerardin 1976 [23]
200	po (tab), sd	test	10	100	0	(22-35)	(62-75)	-	yes	Kim 2005 [27]
200	po (tab), sd	test	24	100	0	(21-35)	(61-93)	-	no	Meyer 1992 [28]
200	po (tab), sd	test	20	100	20	(22-36)	(50-98)	-	no	Meyer 1998 [29]
200	po (tab), sd	test	8	100	0	23 (20-25)	70 (65-75)	-	yes	Shahzadi 2011 [30]
200	po (tab), sd	test	12	100	0	(18-32)	-	-	no	SaintSalvi 1987 [31]
200	po (tab), sd	test	9	100	0	-	69.1 (53-104)	-	no	Wada 1978 [22]
400	po (-, fed), sd	test	7	-	-	(21-27)	(70-90)	-	yes	Barzaghi 1987 [32]
400	po (-, fed), sd	training	24	100	-	(20-40)	-	-	yes	Bianchetti 1987 [33]
400	po (-), sd	training	1	100	0	37	78	-	yes	Faigle 1975 [8]
400	po (-), sd	test	1	100	0	34	83	-	yes	Faigle 1975 [8]
400	po (tab), sd	test	24	100	-	31.8 (20-52)	69.8 (50-96)	-	no	Kovacevic 2009 [34]
400	po (tab), sd	test	5	100	0	25.4	72 8	-	no	Morselli 1975 [35]
400	po (tab), sd	training	6	100	0	(21-22)	(62-77)	-	no	Pynnoenen 1977 [36]
400	po (-), sd	test	12	100	50	(20-31)	-	-	no	Strandjord [37]
400	po (-), sd	test	8	100	0	(24-36)	(72-96)	-	no	Wong 1983 [38]
415.8[b]	po (tab), sd	training	6	100	50	25.8	69.3	-	no	Levy 1975 [15]
415.8[b]	po (tab, fed), sd	training	6	100	50	25.8	69.3	-	no	Levy 1975 [15]
600	po (tab), sd	test	8	100	0	(24-35)	83.4	-	yes	Dalton 1985 [39]
600	po (tab), sd	test	8	100	0	(23-26)	81	-	yes	Dalton 1985a [40]
600	po (tab), sd	test	6	100	0	29	-	-	no	Gerardin 1976 [23]
800	po (tab, fed), sd	test	6	100	50	(21-32)	-	-	no	Cotter 1977 [41]
100/<br>200/<br>400	D1-D3: po (tab), bid<br>D4-D6: po (tab), bid<br>D7-D35: po (tab), qd	test	9	100	37.5	31 (24-43)	-	-	yes	Burstein 2000 [42]
100/<br>200/<br>400	D1-D3: po (tab), bid<br>D4-D6: po (tab), bid<br>D7-D35: po (tab), qd	training	16	100	0	-	-	-	yes	Moller 2001 [43]

-: not given, bid: twice daily, cap: capsule, D: day, iv: intravenous, NA: not applicable, po: oral, qd: once daily, sd: single dose, sol: solution, susp: suspension, tab: tablet, tid: three times daily, XR: extended release

[a] mean (range)

[b] 6 mg/kg

Table S1: Clinical studies used for the development of the carbamazepine parent-metabolite PBPK model *(continued)*

Dose [mg]	Route	Dataset	n	Healthy [%]	Females [%]	Age[a] [years]	Weight[a] [kg]	Height[a] [cm]	CBZE measured	Reference
200	D1: po (tab), bid<br>D2-D21: po (tab), tid	test	1	0	0	57	80	-	yes	Eichelbaum 1975 [26]
200	D1: po (tab), bid<br>D2-D21: po (tab), tid	test	1	0	0	50	80	-	yes	Eichelbaum 1975 [26]
200	po (tab), qd	training	5	100	0	-	77 (69-84)	-	no	Gerardin 1976 [23]
200	po (tab), qd	test	1	100	0	-	84	-	no	Gerardin 1976 [23]
200	po (tab), qd	test	1	100	0	-	80	-	no	Gerardin 1976 [23]
200	po (tab), qd	test	1	100	0	-	73	-	no	Gerardin 1976 [23]
200	po (tab), qd	test	1	100	0	-	69	-	no	Gerardin 1976 [23]
200	po (tab), qd	test	1	100	0	-	79	-	no	Gerardin 1976 [23]
200/<br><br>400	D1-D3: po (tab), qd<br>D4-D6: po (tab), bid<br>D7-D21: po (tab), qd	training	36	100	31	30 (20-45)	75.5 (54-92)	-	yes	Ji 2008 [44]
357	po (tab), qd	test	7	100	0	25.4	78.8	-	yes	Miles 1989 [45]
600	D1,D5: po (tab), sd	training	6	100	0	23.3 (22-26)	67.5 (62-67)	-	yes	Bernus 1994 [46]
400	po (tab XR), sd	test	-	-	-	-	-	-	no	Graf 1990 [47]
400	po (tab XR), sd	test	36	100	0	(20-55)	-	-	yes	Licht 2005 [48]
400	po (tab XR), sd	test	18	100	-	33 (29-37)	72 (70-81)	-	no	Kovacevic 2009 [34]
400	po (tab XR, fed), sd	test	14	100	0	(18-45)	-	-	no	Kshirsagar 2014 [49]
600	po (tab XR), sd	training	19	100	0	24 (19-27)	75	-	no	Licht 2005 [48]
400	po (tab XR), bid	training	18	100	33	27.5	71.4	171.5	yes	Stevens 1998 [50]
300	po (cap XR), sd	test	12	100	33	-	-	-	yes	Gande 2009 [51]
400	po (cap XR), sd	training	12	100	0	33.8 (21-48)	81.3	176.8	yes	McLean 2001 [16]
400	po (cap XR, fed), sd	training	12	100	0	33.8 (21-48)	81.3	176.8	yes	McLean 2001 [16]
400	po (cap XR), bid	training	18	100	33	27.5	71.4	171.5	yes	Stevens 1998 [50]

-: not given, bid: twice daily, cap: capsule, D: day, iv: intravenous, NA: not applicable, po: oral, qd: once daily, sd: single dose, sol: solution, susp: suspension, tab: tablet, tid: three times daily, XR: extended release

[a] mean (range)

[b] 6 mg/kg

2.3 Carbamazepine and Carbamazepine-10,11-epoxide drug-dependent parameters

Table S2: Drug-dependent parameters of the carbamazepine and carbamazepine-10,11-epoxide PBPK model

Parameter	Unit	Model	Literature	Reference	Description
**Carbamazepine**					
MW	g/mol	236.27 (Lit)	236.27	[54]	Molecular weight
logP	Log Units	2.00 (Fit)	1.45, 2.10, 2.45, 2.77	[54–56]	Lipophilicity
Solubility (pH)	mg/ml	0.336 (6.2) (Lit)	0.170 (6.2), 0.283 (7.0), 0.306 (6.9), 0.336 (6.2)	[57–60]	Solubility FaHIF
fu	%	25.0 (Lit)	21.0, 24.0, 25.0	[3, 36, 61, 62]	Fraction unbound in plasma
K_m (CYP3A4) CBZE	µmol/l	248.0 (Lit)	119.0, 248.0, 442.0, 630.0	[6, 63–65]	CYP3A4 Michaelis-Menten constant
k_{cat} (CYP3A4) CBZE	1/min	0.75 (Fit)	1.17, 1.70, 4.87, 5.30[b]	[6, 63–65]	CYP3A4 catalytic rate constant
K_m (CYP2C8)	µmol/l	757.0 (Lit)	757.0	[64]	CYP2C8 Michaelis-Menten constant
k_{cat} (CYP2C8)	1/min	0.67 (Lit)	0.67[b]	[64]	CYP2C8 catalytic rate constant
K_m (CYP2B6)	µmol/l	420.0 (Lit)	420.0	[7]	CYP2B6 Michaelis-Menten constant
k_{cat} (CYP2B6)	1/min	0.43 (Lit)	0.43[b]	[7]	CYP2B6 catalytic rate constant
K_m (CYP3A4)	µmol/l	282.0 (Lit)	282.0	[7]	CYP3A4 Michaelis-Menten constant
k_{cat} (CYP3A4)	1/min	0.20 (Fit)	0.16[b]	[7]	CYP3A4 catalytic rate constant
K_m (UGT2B7)	µmol/l	214.0 (Lit)	214.0	[52]	UGT2B7 Michaelis-Menten constant
k_{cat} (UGT2B7)	1/min	9.53E-3 (Lit)	9.53E-3[c]	[52]	UGT2B7 catalytic rate constant
CL_{hep}	1/min	0.02 (Fit)	-	-	Unspecified hepatic clearance
GFR fraction	-	0.03 (Fit)	-	-	Fraction of filtered drug in the urine
EC_{50} (CYP3A4)	µmol/l	20.00[a] (Lit)	4.3 - 137	[66–73]	Concentration for half-maximal induction
E_{max} (CYP3A4)	-	6.00 (Fit)	1.90 - 23.0	[66–73]	CYP3A4 maximum induction effect
EC_{50} (CYP2B6)	µmol/l	20.0[a] (Asm)	22 - 145	[73–75]	Concentration for half-maximal induction
E_{max} (CYP2B6)	-	17.0 (Fit)	3.10 - 21.50	[73–75]	CYP2B6 maximum induction effect
EC_{50} (EPHX1)	µmol/l	20.0[a] (Asm)	-	-	Concentration for half-maximal induction
E_{max} (EPHX1)	-	3.25 (Fit)	-	-	EPHX1 maximum induction effect
Intestinal permeability	cm/min	2.58E-2 (Lit)	2.58E-2	[76]	Transcellular intestinal permeability
Partition coefficients	-	Diverse	Rodgers and Rowlands	[77, 78]	Cell to plasma partition coefficients

asm: assumption, calc: calculated, CBZE: carbamazepine-10,11-epoxide, CL_{hep}: hepatic clearance, CL_{spec}: specific clearance, CYP: cytochrome P450, EPHX1: epoxide hydroxylase 1, FaHIF: fasted human intestinal fluid, IR: immediate release, fit: optimized during parameter optimization, lit: literature, UGT: UDP-glucuronosyltransferase, XR: extended release

[a] mean of literature values for EC_{50} (CYP3A4), assumed for all EC_{50} values

[b] k_{cat} values calculated within PK-Sim from V_{max}/recombinant enzyme

[c] k_{cat} value calculated within PK-Sim from V_{max} = 0.79 pmol/min/microsomal protein [52], assuming a microsomal UGT2B7 content of 82.9 pmol/mg microsomal protein [53], kcat = Vmax/ UGT2B7 content microsomes

Table S2: Drug-dependent parameters of the carbamazepine and carbamazepine-10,11-epoxide PBPK model *(continued)*

Parameter	Unit	Model	Literature	Reference	Description
Cellular permeability	cm/min	0.02 (Calc)	PK-Sim Standard	[79]	Permeability into the cellular space
IR tablet (fasted) Weibull time	min	200.0	-	-	Dissolution time (50% dissolved)
IR tablet (fasted) Weibull shape	-	0.74	-	-	Dissolution profile shape
IR tablet (fed) Weibull time	min	100.0	-	-	Dissolution time (50% dissolved)
IR tablet (fed) Weibull shape	-	1.20	-	-	Dissolution profile shape
XR tablet (fasted) Weibull time	min	767.2	-	-	Dissolution time (50% dissolved)
XR tablet (fasted) Weibull shape	-	0.76	-	-	Dissolution profile shape
XR tablet (fed) Weibull time	min	436.5	-	-	Dissolution time (50% dissolved)
XR tablet (fed) Weibull shape	-	1.16	-	-	Dissolution profile shape
XR capsule (fed) Weibull time	min	361.4	-	-	Dissolution time (50% dissolved)
XR capsule (fed) Weibull shape	-	2.13	-	-	Dissolution profile shape
XR capsule (fasted) Weibull time	min	439.5	-	-	Dissolution time (50% dissolved)
XR capsule (fasted) Weibull shape	-	0.7	-	-	Dissolution profile shape
**Carbamazepine-10,11-epoxide**					
logP	Log Units	1.00 (Fit)	1.58, 1.97	[80]	Lipophilicity
Solubility	mg/ml	1.34 (Lit)	1.34	[80]	Solubility
fu	%	51.8 (Lit)	46.8-51.8	[35]	Fraction unbound in plasma
CL_{spec} (EPHX1)	1/min	0.01 (Fit)	0.05	-	EPHX1 first-order clearance
GFR fraction	-	0.21 (Fit)	-	-	Fraction of filtered drug in the urine
Intestinal permeability	cm/min	0.3 (Fit)	-	-	Transcellular intestinal permeability
Partition coefficients	-	Diverse	Rodgers and Rowlands	[77, 78]	Cell to plasma partition coefficients
Cellular permeability	cm/min	1.61E-3 (Calc)	PK-Sim Standard	[79]	Permeability into the cellular space
Tablet Weibull time	min	200.0	-	-	Dissolution time (50% dissolved)
Tablet Weibull shape	-	0.75	-	-	Dissolution profile shape

asm: assumption, calc: calculated, CBZE: carbamazepine-10,11-epoxide, CL_{hep}: hepatic clearance, CL_{spec}: specific clearance, CYP: cytochrome P450, EPHX1: epoxide hydroxylase 1, FaHIF: fasted human intestinal fluid, IR: immediate release, fit: optimized during parameter optimization, lit: literature, UGT: UDP-glucuronosyltransferase, XR: extended release

[a] mean of literature values for EC_{50} (CYP3A4), assumed for all EC_{50} values

[b] k_{cat} values calculated within PK-Sim from V_{max}/recombinant enzyme

[c] k_{cat} value calculated within PK-Sim from V_{max} = 0.79 pmol/min/microsomal protein [52], assuming a microsomal UGT2B7 content of 82.9 pmol/mg microsomal protein [53], kcat = Vmax/ UGT2B7 content microsomes

2.4 Profiles

2.4.1 Carbamazepine-10,11-epoxide model

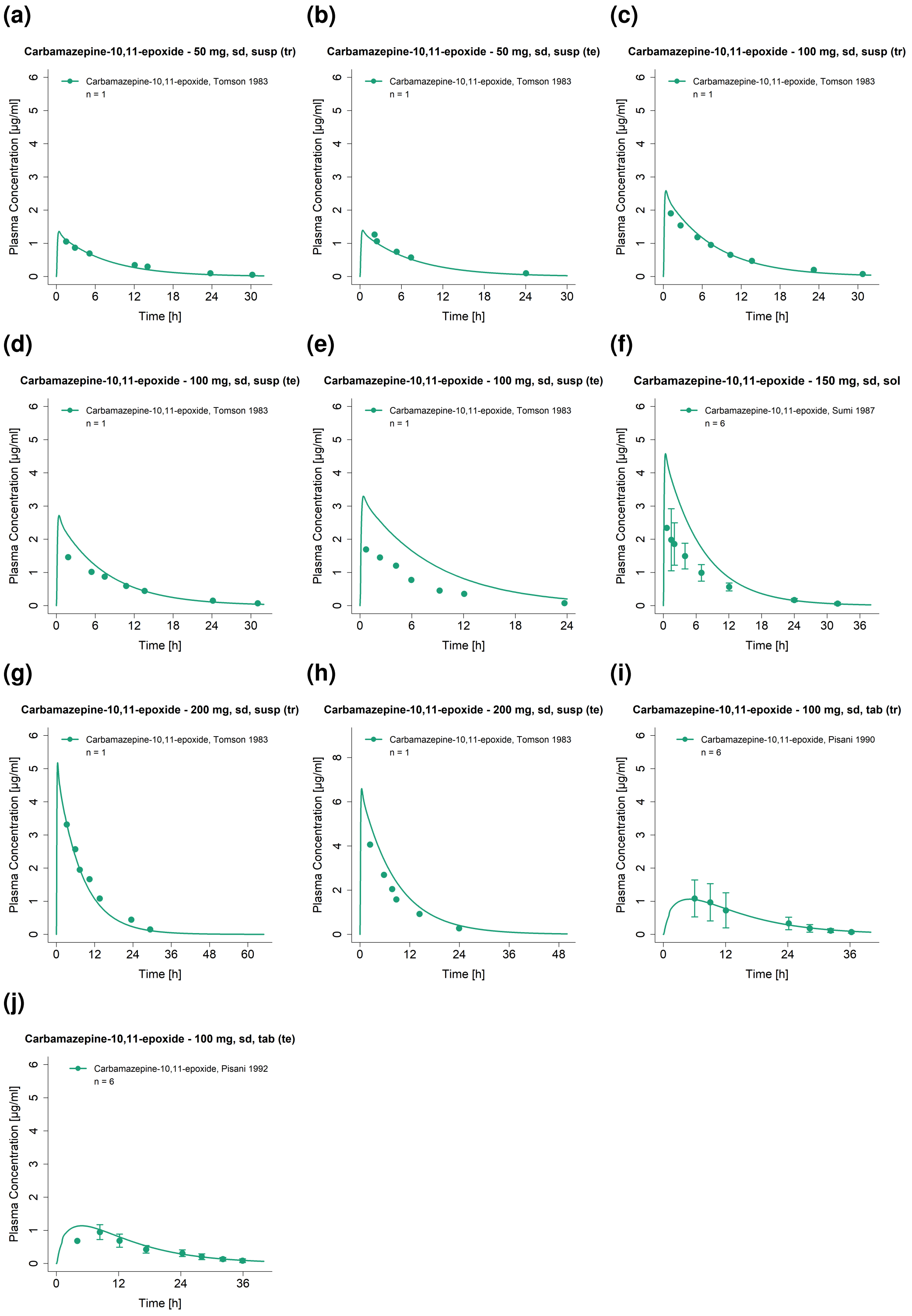

Figure S2: Predicted compared to observed carbamazepine-10,11-epoxide plasma concentration-time profiles (linear) after oral administration of carbamazepine-10,11-epoxide. Observed data are shown as dots ± standard deviation; model predictions are shown as solid lines. Details on dosing regimens, study populations and literature references are listed in Table S1. sd: single dose, sol: solution, susp: suspension, tab: tablet

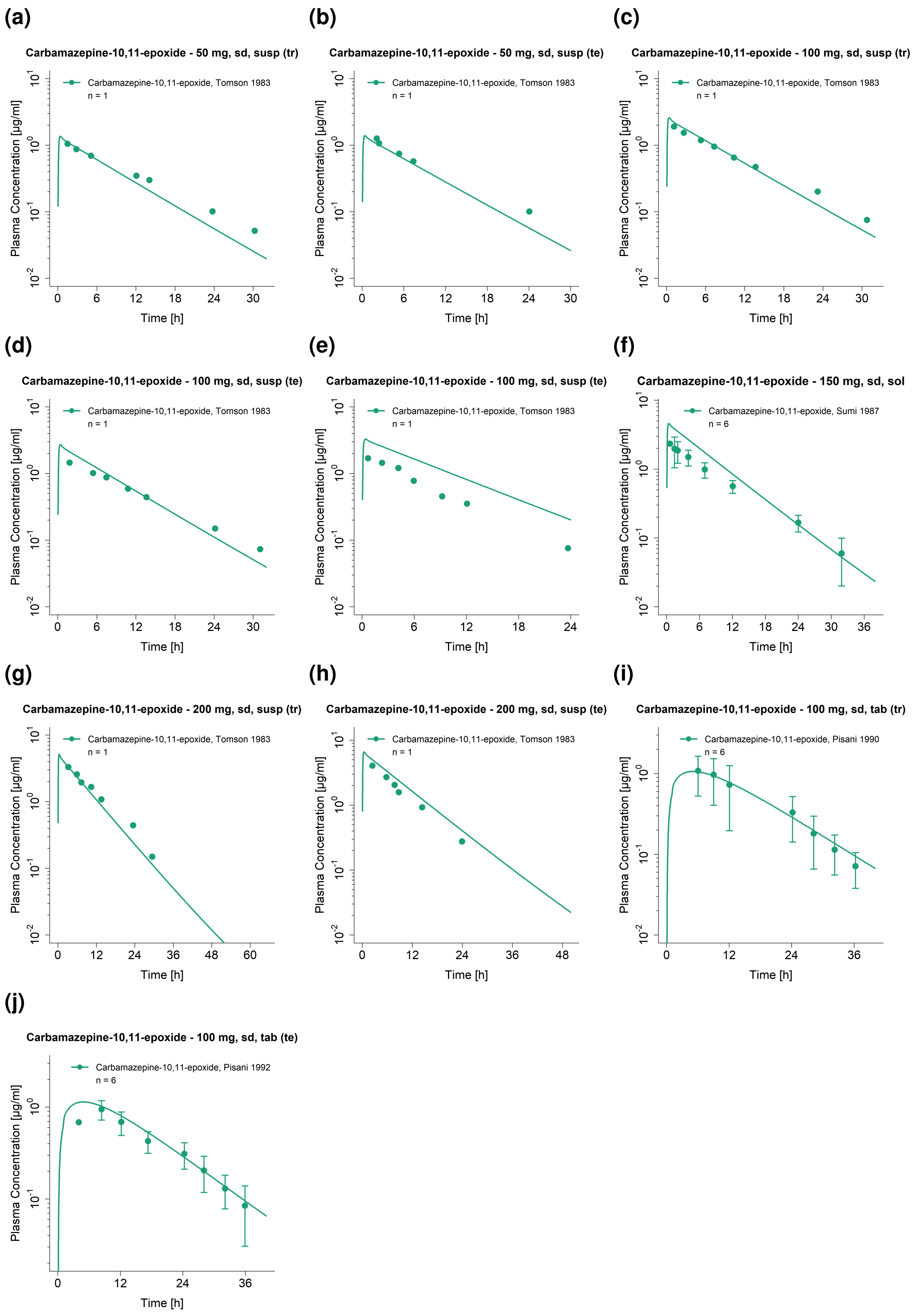

Figure S3: Predicted compared to observed carbamazepine-10,11-epoxide plasma concentration-time profiles (semi-logarithmic) after oral administration of carbamazepine-10,11-epoxide. Observed data are shown as dots ± standard deviation; model predictions are shown as solid lines. Details on dosing regimens, study populations and literature references are listed in Table S1. sd: single dose, sol: solution, susp: suspension, tab: tablet

2.4.2 Carbamazepine parent-metabolite model

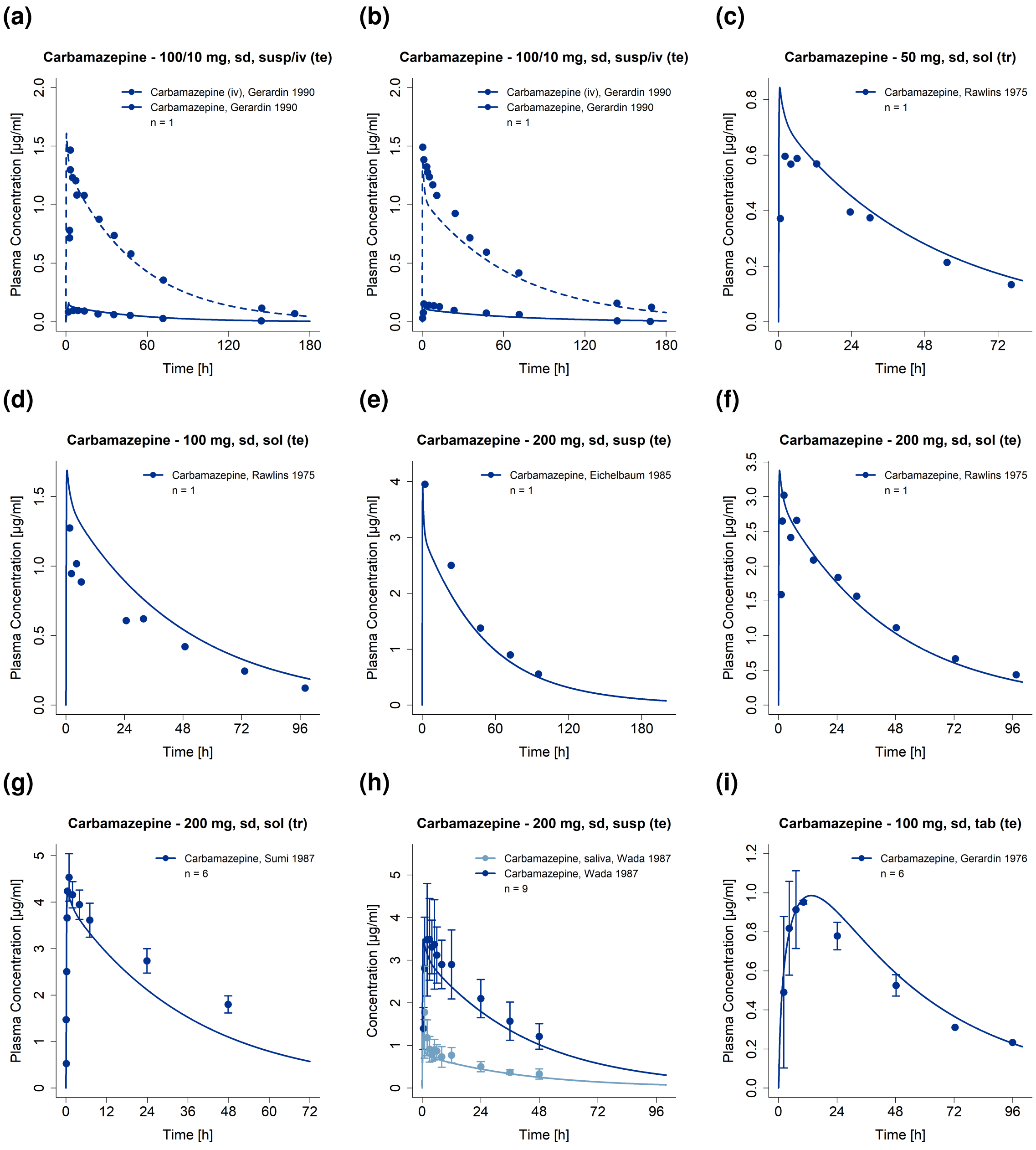

Figure S4: Predicted compared to observed carbamazepine and carbamazepine-10,11-epoxide plasma (and saliva) concentration-time profiles (linear) after intravenous and oral administration of carbamazepine. Observed data are shown as dots ± standard deviation; model predictions are shown as solid lines. Details on dosing regimens, study populations and literature references are listed in Table S1. bid: twice daily, cap: capsule, D: day, iv: intravenous, qd: once daily, sd: single dose, sol: solution, susp: suspension, tab: tablet, tab*: tablet with concomitant food intake, tid: three times daily, XR: extended release

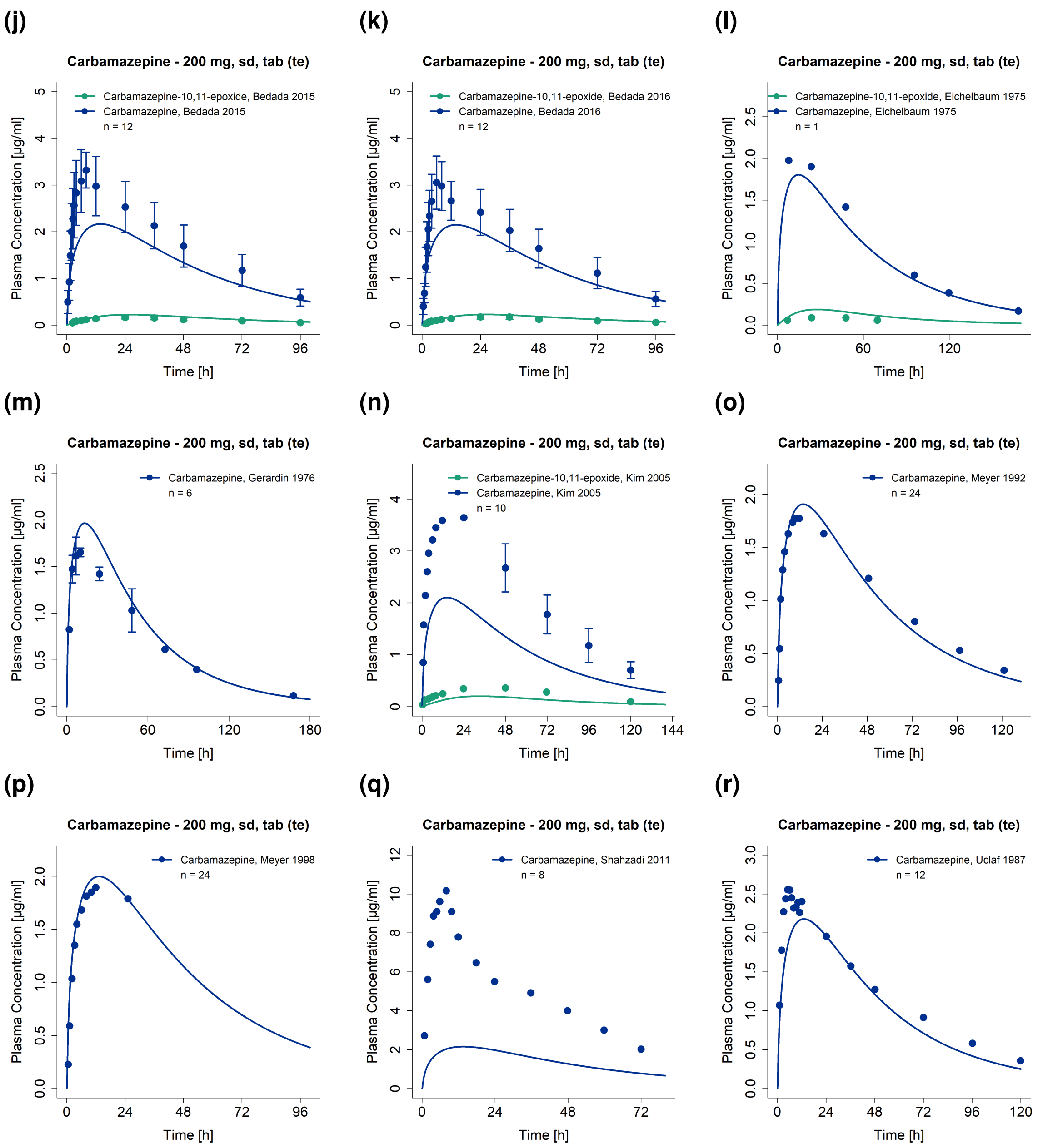

Figure S4: Predicted compared to observed carbamazepine and carbamazepine-10,11-epoxide plasma (and saliva) concentration-time profiles (linear) after intravenous and oral administration of carbamazepine. Observed data are shown as dots ± standard deviation; model predictions are shown as solid lines. Details on dosing regimens, study populations and literature references are listed in Table S1. bid: twice daily, cap: capsule, D: day, iv: intravenous, qd: once daily, sd: single dose, sol: solution, susp: suspension, tab: tablet, tab*: tablet with concomitant food intake, tid: three times daily, XR: extended release *(continued)*

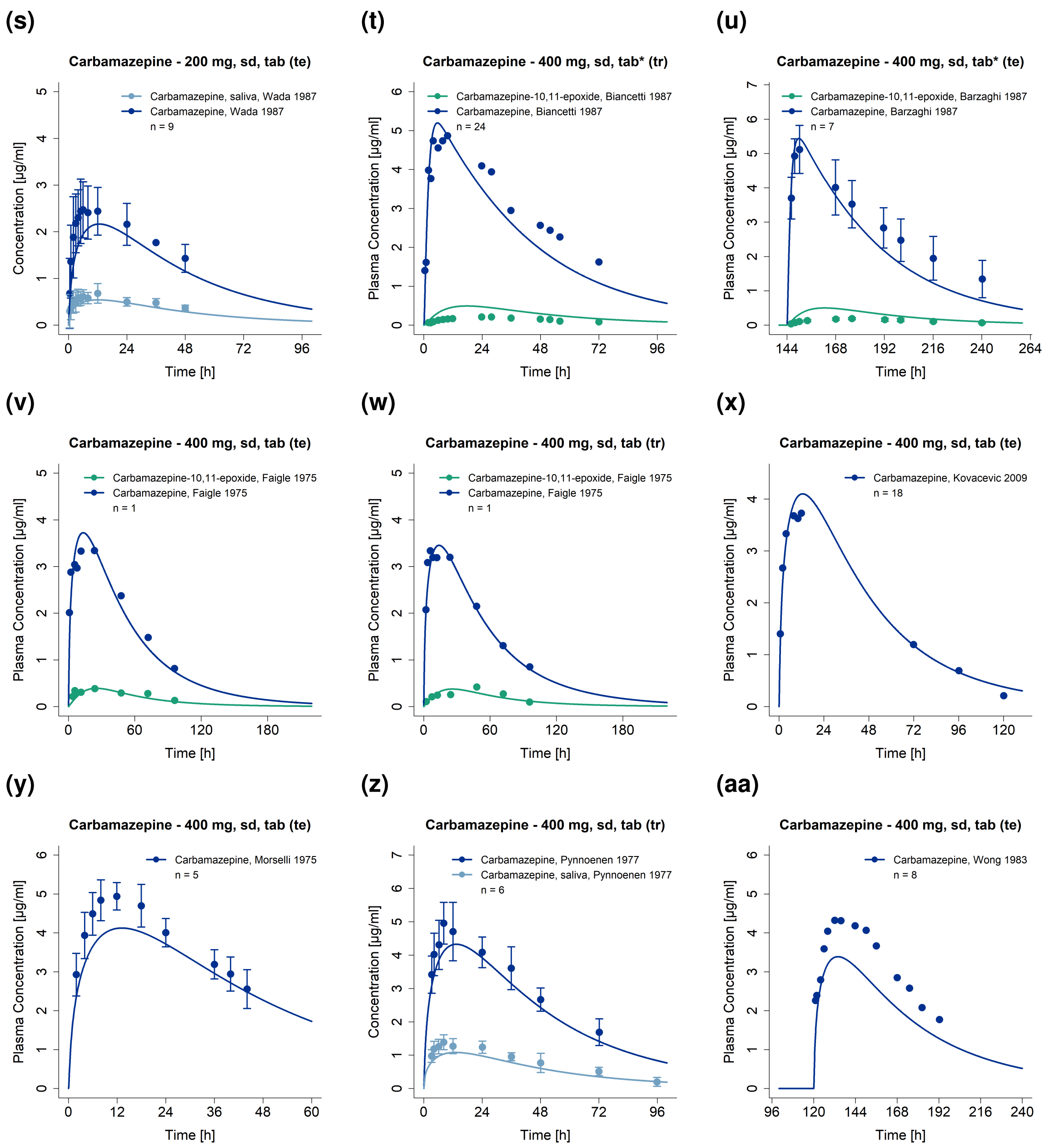

Figure S4: Predicted compared to observed carbamazepine and carbamazepine-10,11-epoxide plasma (and saliva) concentration-time profiles (linear) after intravenous and oral administration of carbamazepine. Observed data are shown as dots ± standard deviation; model predictions are shown as solid lines. Details on dosing regimens, study populations and literature references are listed in Table S1. bid: twice daily, cap: capsule, D: day, iv: intravenous, qd: once daily, sd: single dose, sol: solution, susp: suspension, tab: tablet, tab*: tablet with concomitant food intake, tid: three times daily, XR: extended release *(continued)*

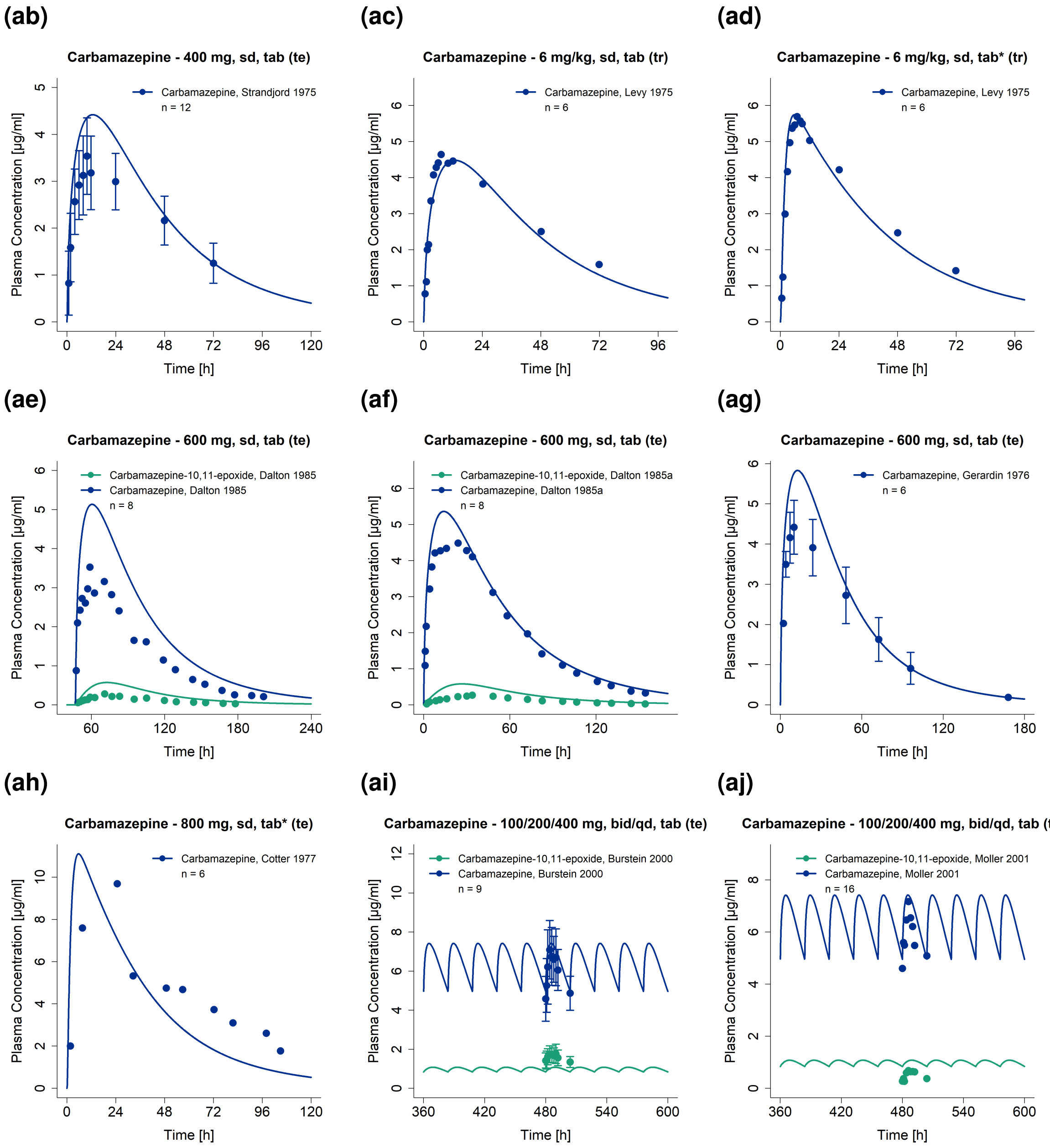

Figure S4: Predicted compared to observed carbamazepine and carbamazepine-10,11-epoxide plasma (and saliva) concentration-time profiles (linear) after intravenous and oral administration of carbamazepine. Observed data are shown as dots ± standard deviation; model predictions are shown as solid lines. Details on dosing regimens, study populations and literature references are listed in Table S1. bid: twice daily, cap: capsule, D: day, iv: intravenous, qd: once daily, sd: single dose, sol: solution, susp: suspension, tab: tablet, tab*: tablet with concomitant food intake, tid: three times daily, XR: extended release *(continued)*

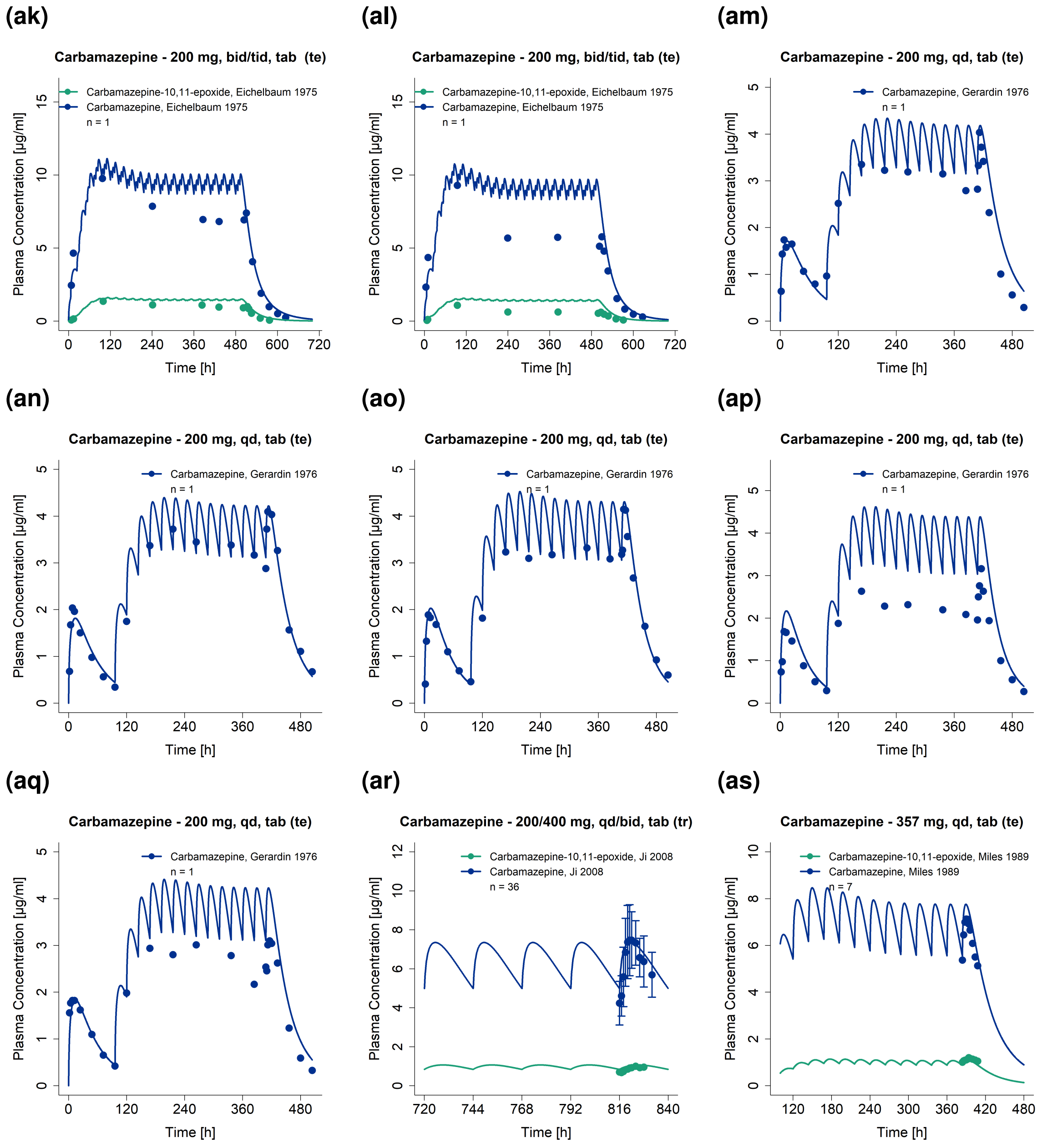

Figure S4: Predicted compared to observed carbamazepine and carbamazepine-10,11-epoxide plasma (and saliva) concentration-time profiles (linear) after intravenous and oral administration of carbamazepine. Observed data are shown as dots ± standard deviation; model predictions are shown as solid lines. Details on dosing regimens, study populations and literature references are listed in Table S1. bid: twice daily, cap: capsule, D: day, iv: intravenous, qd: once daily, sd: single dose, sol: solution, susp: suspension, tab: tablet, tab*: tablet with concomitant food intake, tid: three times daily, XR: extended release *(continued)*

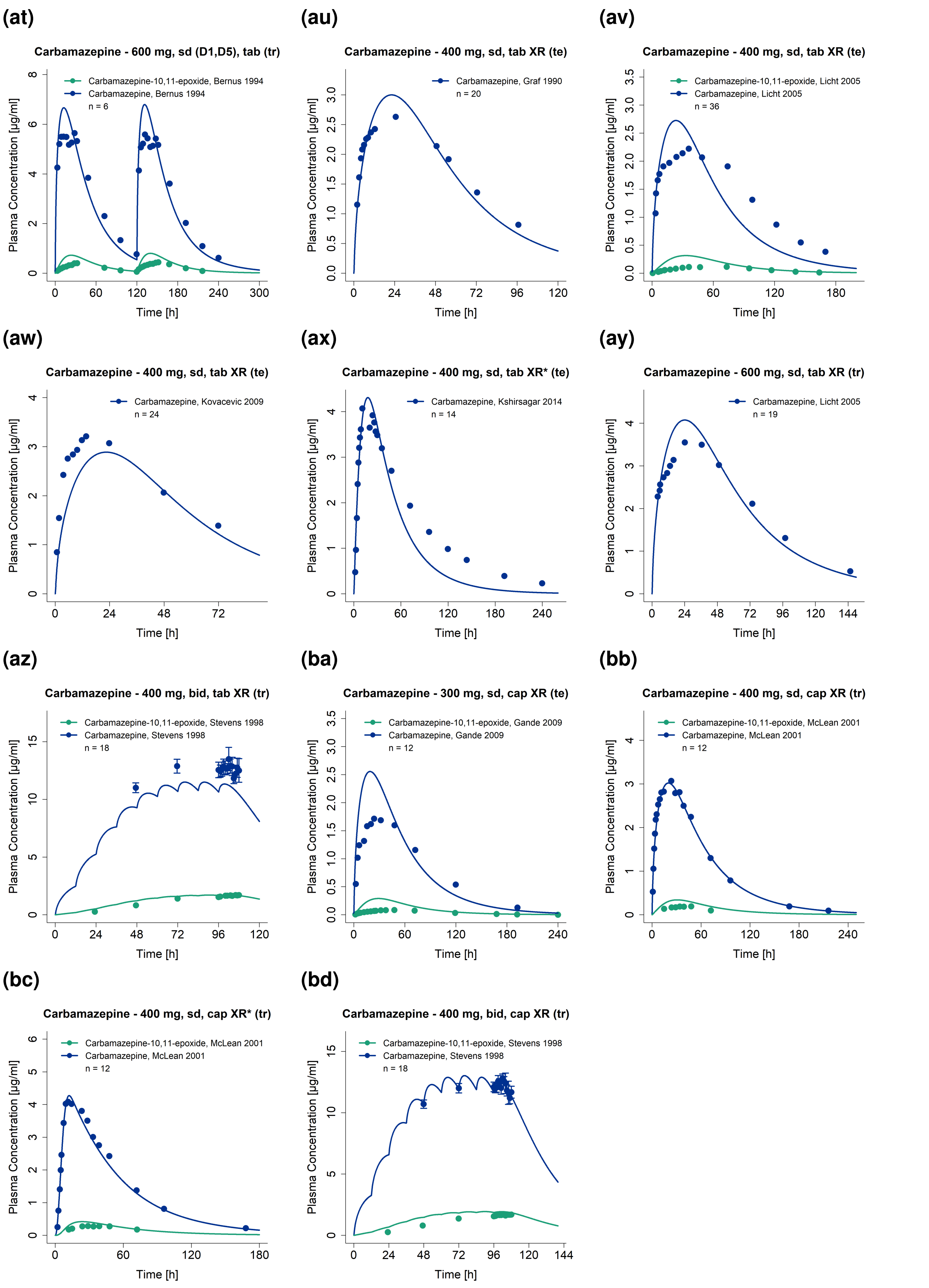

Figure S4: Predicted compared to observed carbamazepine and carbamazepine-10,11-epoxide plasma (and saliva) concentration-time profiles (linear) after intravenous and oral administration of carbamazepine. Observed data are shown as dots ± standard deviation; model predictions are shown as solid lines. Details on dosing regimens, study populations and literature references are listed in Table S1. bid: twice daily, cap: capsule, D: day, iv: intravenous, qd: once daily, sd: single dose, sol: solution, susp: suspension, tab: tablet, tab*: tablet with concomitant food intake, tid: three times daily, XR: extended release *(continued)*

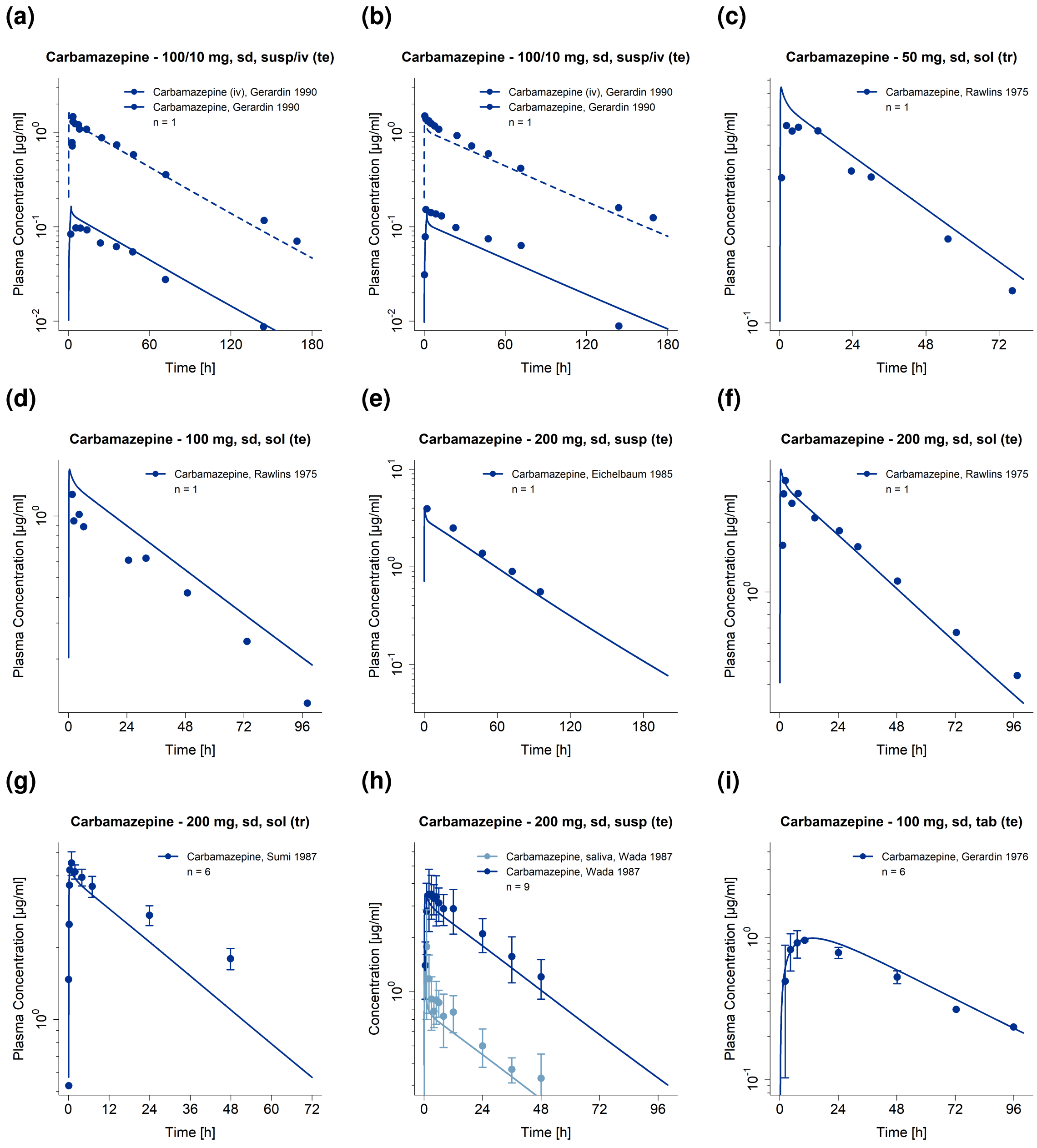

Figure S5: Predicted compared to observed carbamazepine and carbamazepine-10,11-epoxide plasma (and saliva) concentration-time profiles (semi-logarithmic) after intravenous and oral administration of carbamazepine. Observed data are shown as dots ± standard deviation; model predictions are shown as solid lines. Details on dosing regimens, study populations and literature references are listed in Table S1. bid: twice daily, cap: capsule, D: day, iv: intravenous, qd: once daily, sd: single dose, sol: solution, susp: suspension, tab: tablet, tab*: tablet with concomitant food intake, tid: three times daily, XR: extended release

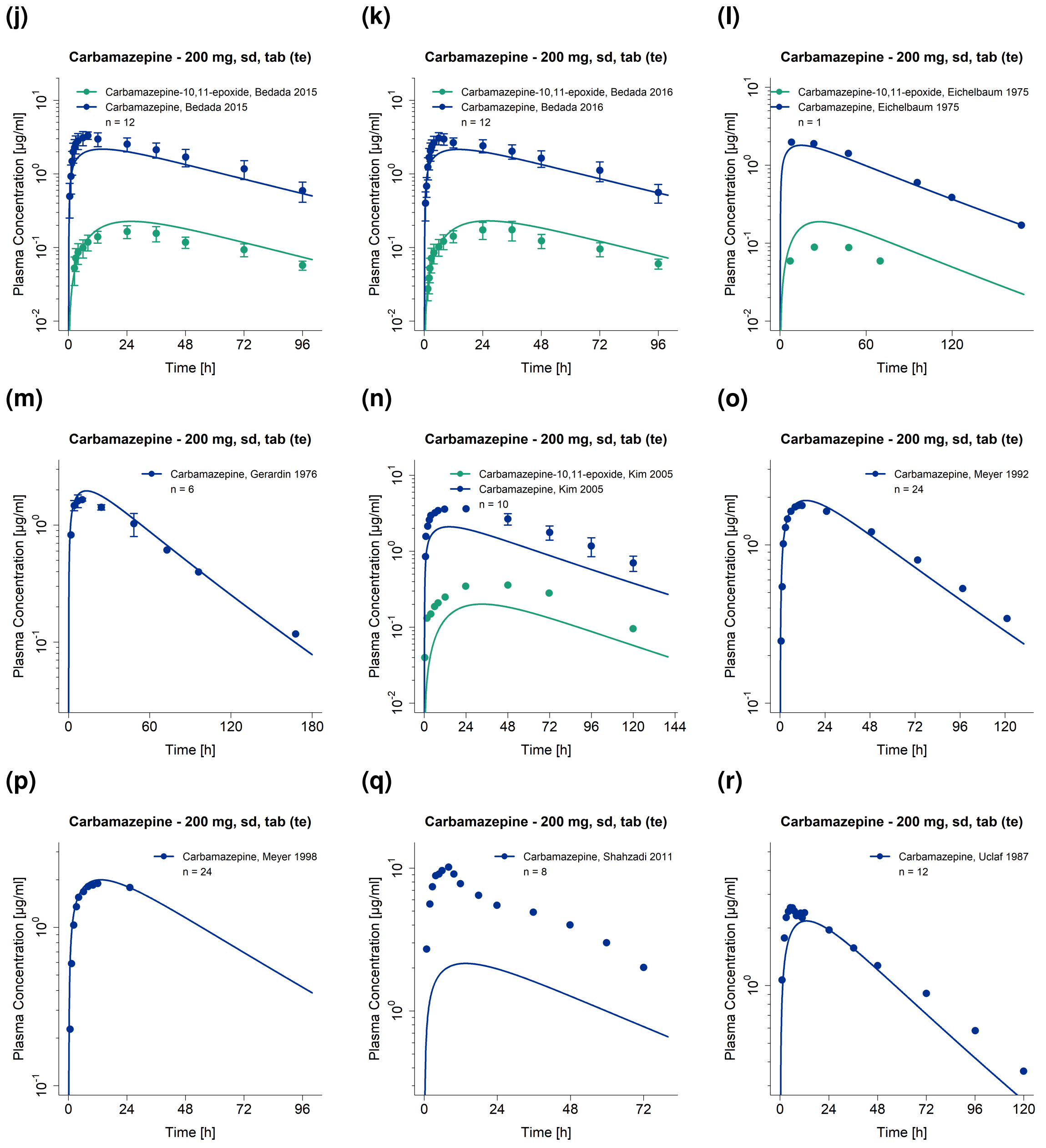

Figure S5: Predicted compared to observed carbamazepine and carbamazepine-10,11-epoxide plasma (and saliva) concentration-time profiles (semi-logarithmic) after intravenous and oral administration of carbamazepine. Observed data are shown as dots ± standard deviation; model predictions are shown as solid lines. Details on dosing regimens, study populations and literature references are listed in Table S1. bid: twice daily, cap: capsule, D: day, iv: intravenous, qd: once daily, sd: single dose, sol: solution, susp: suspension, tab: tablet, tab*: tablet with concomitant food intake, tid: three times daily, XR: extended release *(continued)*

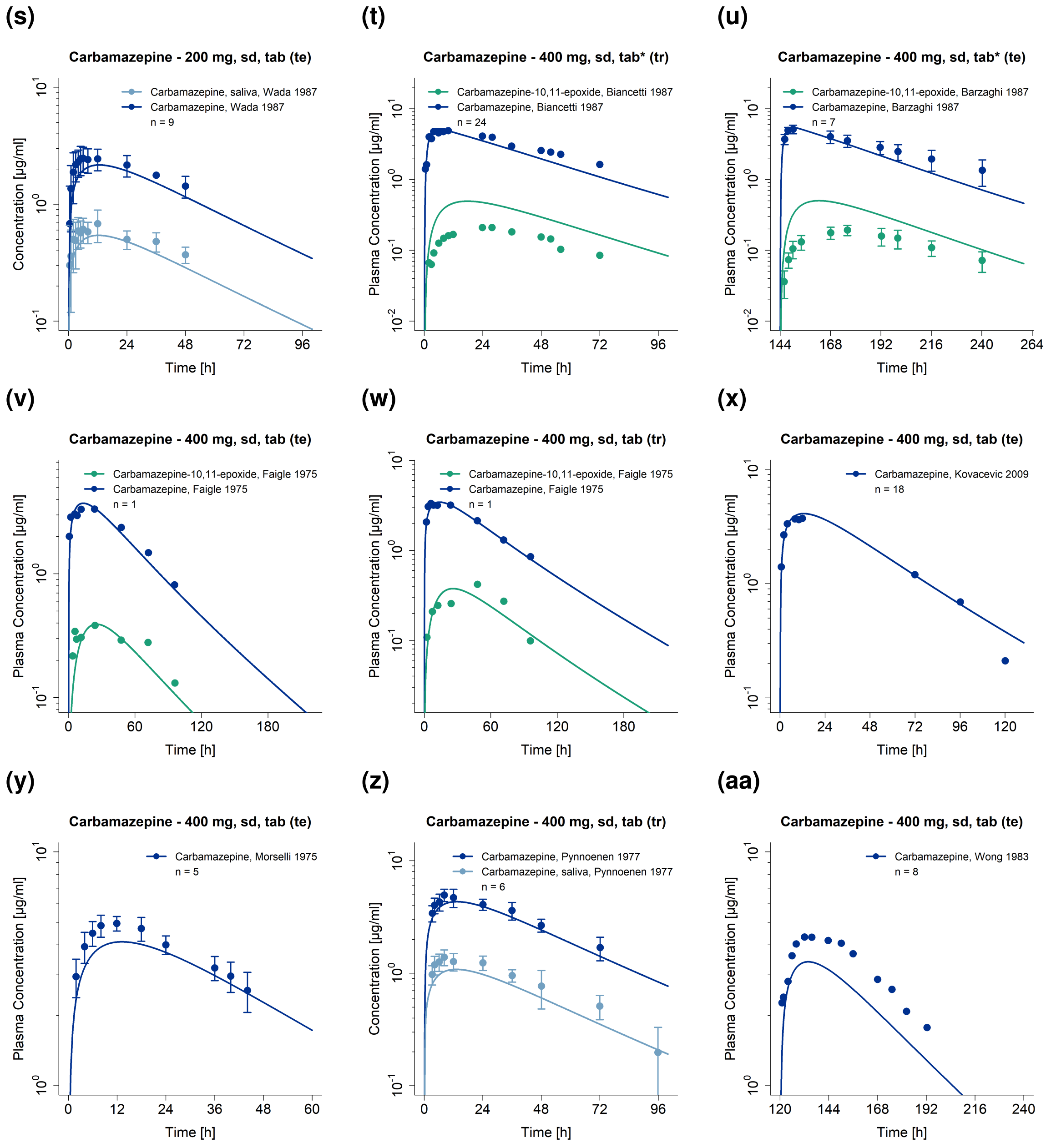

Figure S5: Predicted compared to observed carbamazepine and carbamazepine-10,11-epoxide plasma (and saliva) concentration-time profiles (semi-logarithmic) after intravenous and oral administration of carbamazepine. Observed data are shown as dots ± standard deviation; model predictions are shown as solid lines. Details on dosing regimens, study populations and literature references are listed in Table S1. bid: twice daily, cap: capsule, D: day, iv: intravenous, qd: once daily, sd: single dose, sol: solution, susp: suspension, tab: tablet, tab*: tablet with concomitant food intake, tid: three times daily, XR: extended release *(continued)*

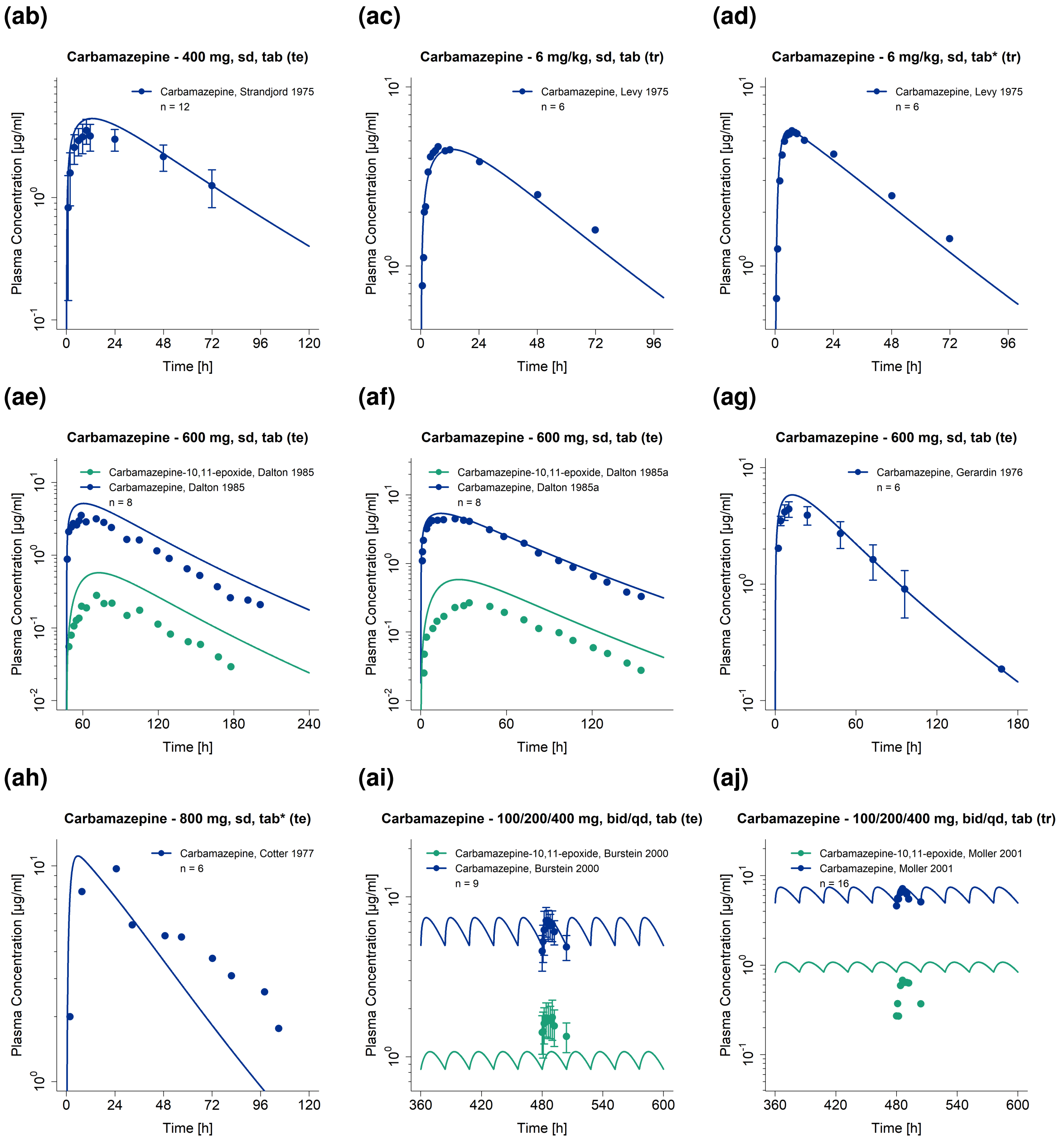

Figure S5: Predicted compared to observed carbamazepine and carbamazepine-10,11-epoxide plasma (and saliva) concentration-time profiles (semi-logarithmic) after intravenous and oral administration of carbamazepine. Observed data are shown as dots ± standard deviation; model predictions are shown as solid lines. Details on dosing regimens, study populations and literature references are listed in Table S1. bid: twice daily, cap: capsule, D: day, iv: intravenous, qd: once daily, sd: single dose, sol: solution, susp: suspension, tab: tablet, tab*: tablet with concomitant food intake, tid: three times daily, XR: extended release *(continued)*

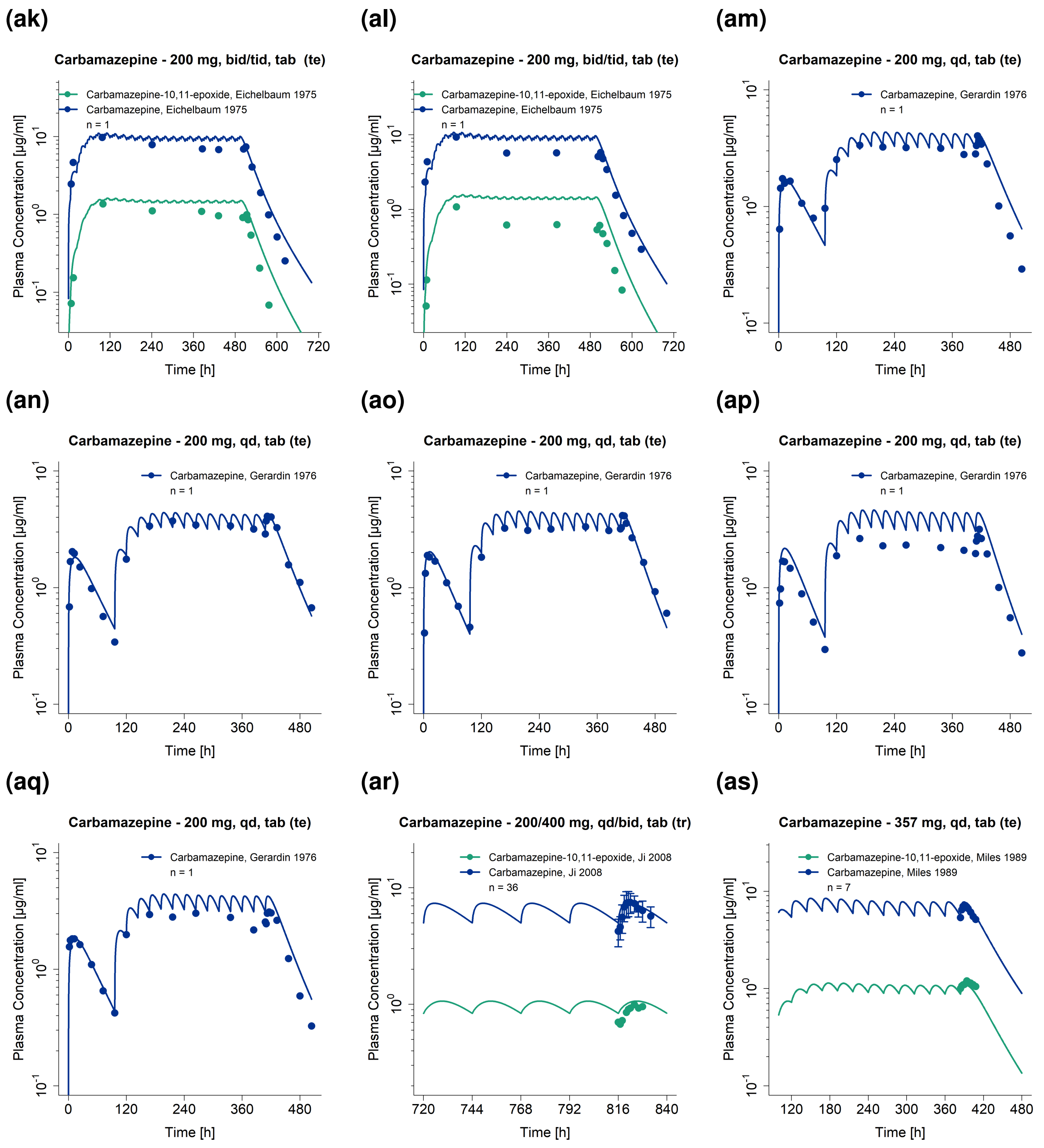

Figure S5: Predicted compared to observed carbamazepine and carbamazepine-10,11-epoxide plasma (and saliva) concentration-time profiles (semi-logarithmic) after intravenous and oral administration of carbamazepine. Observed data are shown as dots ± standard deviation; model predictions are shown as solid lines. Details on dosing regimens, study populations and literature references are listed in Table S1. bid: twice daily, cap: capsule, D: day, iv: intravenous, qd: once daily, sd: single dose, sol: solution, susp: suspension, tab: tablet, tab*: tablet with concomitant food intake, tid: three times daily, XR: extended release *(continued)*

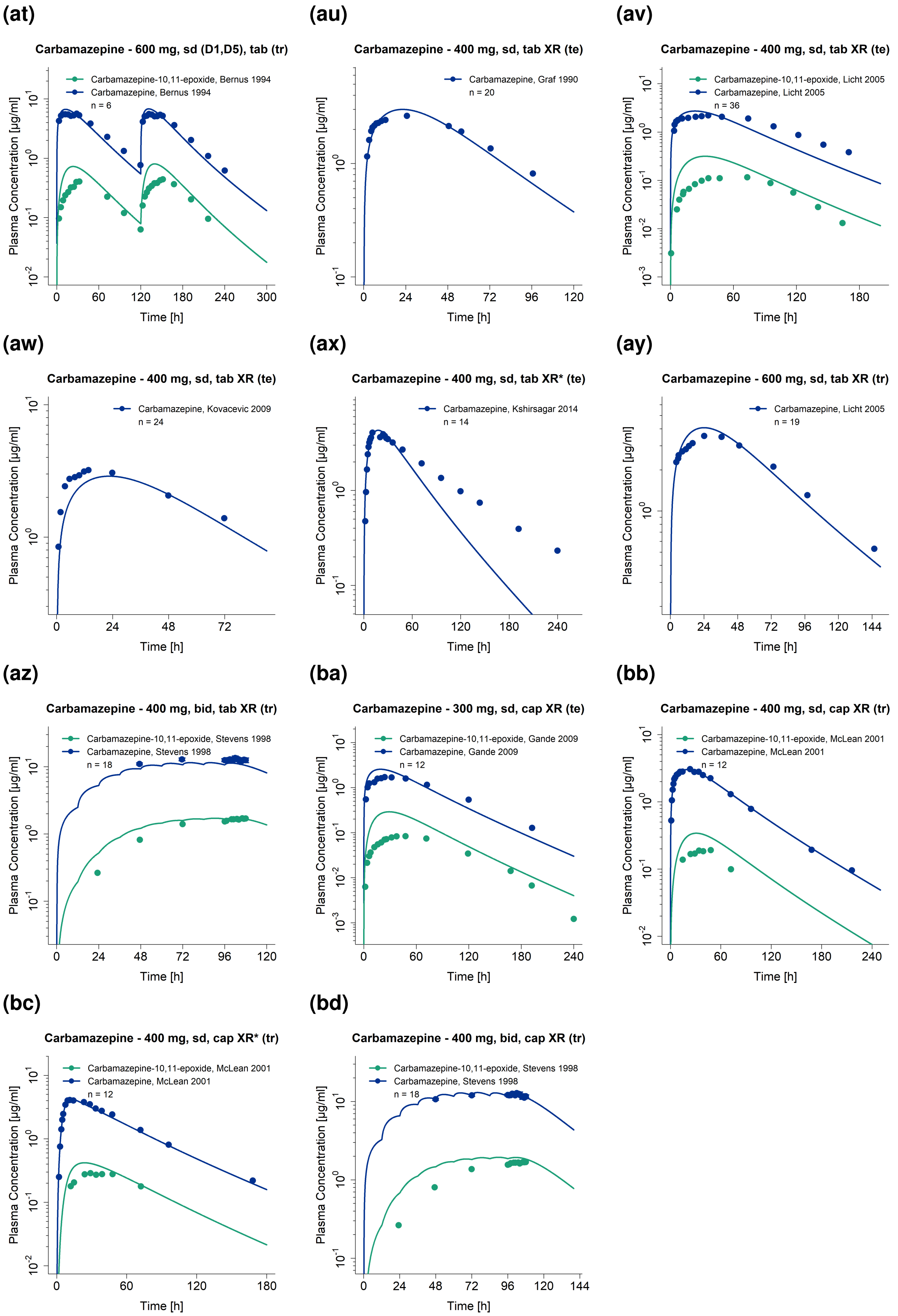

Figure S5: Predicted compared to observed carbamazepine and carbamazepine-10,11-epoxide plasma (and saliva) concentration-time profiles (semi-logarithmic) after intravenous and oral administration of carbamazepine. Observed data are shown as dots ± standard deviation; model predictions are shown as solid lines. Details on dosing regimens, study populations and literature references are listed in Table S1. bid: twice daily, cap: capsule, D: day, iv: intravenous, qd: once daily, sd: single dose, sol: solution, susp: suspension, tab: tablet, tab*: tablet with concomitant food intake, tid: three times daily, XR: extended release *(continued)*

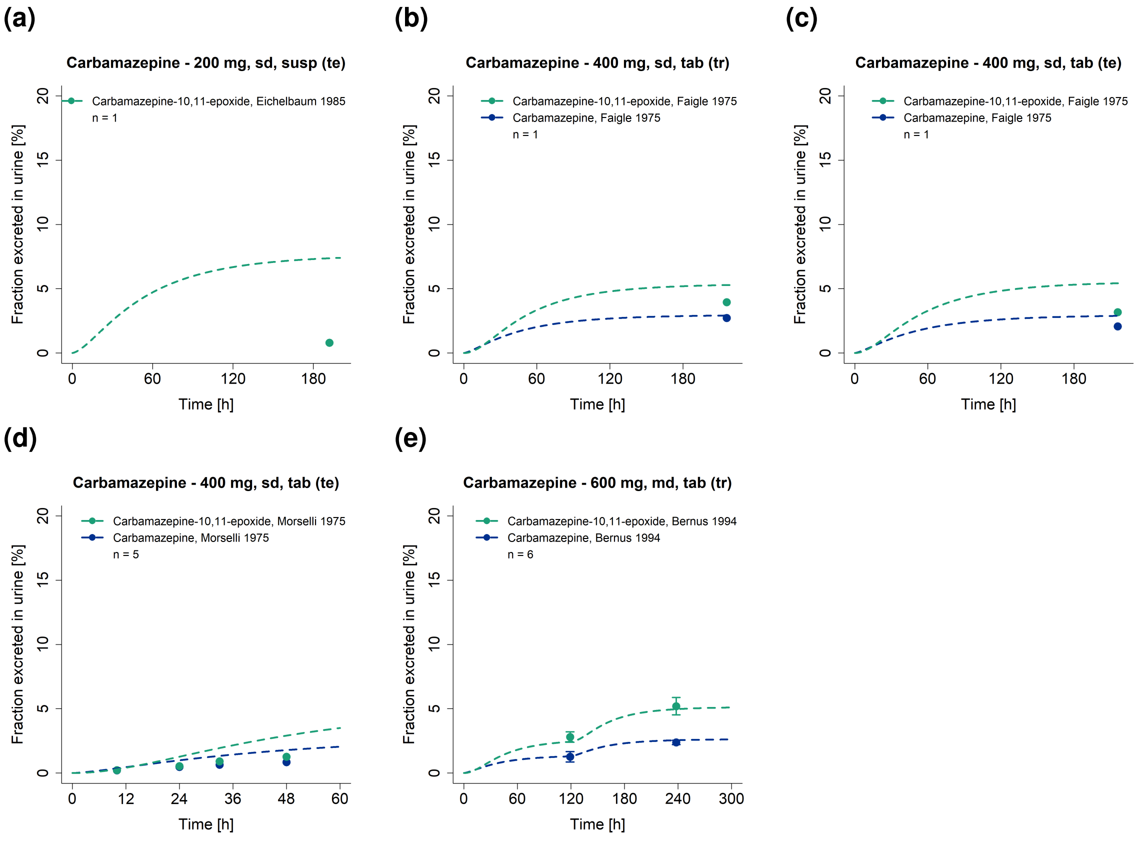

Figure S6: Predicted compared to observed carbamazepine and carbamazepine-10,11-epoxide fractions excreted unchanged in urine profiles (linear). Observed data are shown as dots ± standard deviation; model predictions are shown as solid lines. Details on dosing regimens, study populations and literature references are listed in Table S1. md: multiple dose, susp: suspension, sd: single dose, tab: tablet

2.5 Model evaluation

2.5.1 Plasma concentration goodness-of-fit plots

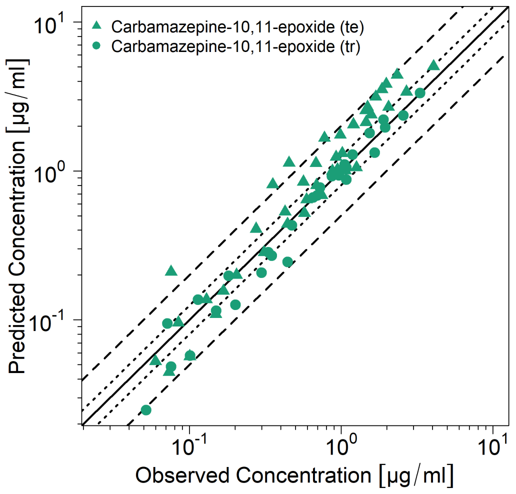

Figure S7: Predicted compared to observed carbamazepine-10,11-epoxide plasma concentrations after oral administration of carbamazepine-10,11-epoxide, predicted with the carbamazepine-10,11-epoxide PBPK model. The solid line marks the line of identity. Dotted lines indicate 1.25-fold, dashed lines indicate 2-fold deviation.

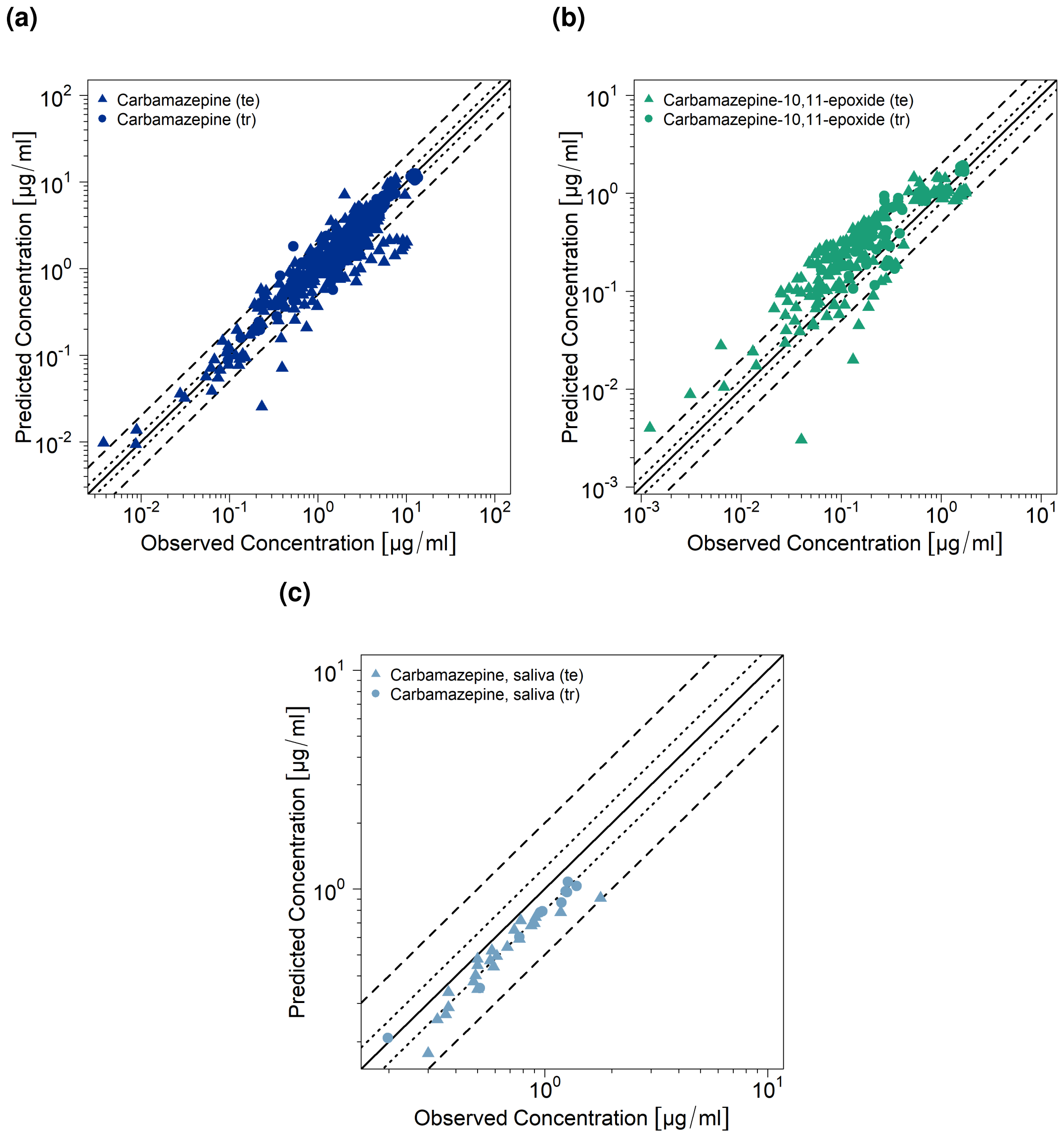

Figure S8: Predicted compared to observed (a) carbamazepine plasma concentrations (b) carbamazepine-10,11-epoxide plasma concentrations and (c) carbamazepine saliva concentrations after intravenous and oral administration of carbamazepine. The solid line marks the line of identity. Dotted lines indicate 1.25-fold, dashed lines indicate 2-fold deviation.

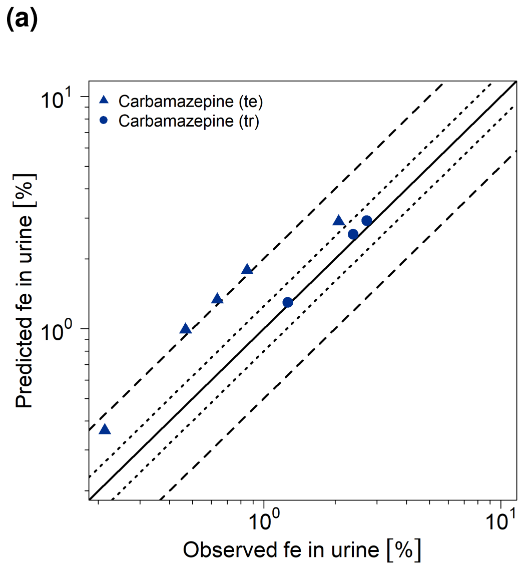

Figure S9: Predicted compared to observed fractions excreted unchanged in urine of (a) carbamazepine and (b) carbamazepine-10,11-epoxide after oral administration of carbamazepine. The solid line marks the line of identity. Dotted lines indicate 1.25-fold, dashed lines indicate 2-fold deviation

2.5.2 Mean relative deviation of predicted plasma concentrations

Table S3: Mean relative deviation values of predicted carbamazepine and carbamazepine-10,11-epoxide plasma and saliva concentrations

Dose [mg]	Route	CBZ MRD	CBZE MRD	CBZ (saliva) MRD	Reference
**Carbamazepine-10,11-epoxide administration**					
50	po (sol), sd	-	1.41	-	Tomson 1983 [5]
50	po (sol), sd	-	1.25	-	Tomson 1983 [5]
100	po (sol), sd	-	1.27	-	Tomson 1983 [5]
100	po (sol), sd	-	2.21	-	Tomson 1983 [5]
100	po (sol), sd	-	1.35	-	Tomson 1983 [5]
150	po (sol), sd	-	1.67	-	Sumi 1987 [17]
200	po (sol), sd	-	1.25	-	Tomson 1983 [5]
200	po (sol), sd	-	1.46	-	Tomson 1983 [5]
100	po (tab), sd	-	1.16	-	Pisani 1990 [18]
100	po (tab), sd	-	1.23	-	Pisani 1992 [19]
**mean MRD (range)**		**-**	**1.42 (1.16-2.21)**	**-**	
		**-**	**9/10 with MRD ≤ 2**	**-**	
**Carbamazepine administration**					
100/10	po (susp), sd; iv (2 h)	1.30	-	-	Gerardin 1990 [81]
100/10	po (susp), sd; iv (2 h)	1.55	-	-	Gerardin 1990 [81]
50	po (sol), sd	1.35	-	-	Rawlins 1975 [21]
100	po (sol), sd	1.39	-	-	Rawlins 1975 [21]
200	po (susp), sd	1.20	-	-	Eichelbaum 1985 [4]
200	po (sol), sd	1.26	-	-	Rawlins 1975 [21]
200	po (sol), sd	1.68	-	-	Sumi 1987 [17]
200	po (susp), sd	1.60	-	-	Tomson 1983 [5]
200	po (susp), sd	1.35	-	1.35	Wada 1978 [22]
100	po (tab), sd	1.12	-	-	Gerardin 1976 [23]
200	po (tab), sd	1.44	1.28	-	Bedada 2015 [24]
200	po (tab), sd	1.36	1.24	-	Bedada 2016 [25]
200	po (tab), sd	1.12	1.86	-	Eichelbaum 1975 [26]
200	po (tab), sd	1.17	-	-	Gerardin 1976 [23]
200	po (tab), sd	1.83	3.47	-	Kim 2005 [27]

bid: twice daily, cap: capsule, CBZ: carbamazepine, CBZE: carbamazepine-10,11-epoxide, D: day, iv: intravenous, MRD: mean relative deviation, po: oral, qd: once daily, sd: single dose, sol: solution, susp: suspension, tab: tablet, tid: three times daily, XR: extended release

Table S3: Mean relative deviation values of predicted carbamazepine and carbamazepine-10,11-epoxide plasma and saliva concentrations *(continued)*

Dose [mg]	Route	CBZ MRD	CBZE MRD	CBZ (saliva) MRD	Reference
200	po (tab), sd	1.27	-	-	Meyer 1992 [28]
200	po (tab), sd	1.39	-	-	Meyer 1998 [29]
200	po (tab), sd	3.98	-	-	Shahzadi 2011 [30]
200	po (tab), sd	1.27	-	-	Saint-Salvi 1987 [31]
200	po (tab), sd	1.32	-	1.31	Wada 1978 [22]
400	po (tab, fed), sd	1.35	2.41	-	Barzaghi 1987 [32]
400	po (tab, fed), sd	1.31	2.28	-	Bianchetti 1987 [33]
400	po (tab), sd	1.22	1.48	-	Faigle 1975 [8]
400	po (tab), sd	1.08	1.35	-	Faigle 1975 [8]
400	po (tab), sd	1.24	-	-	Kovacevic 2009 [34]
400	po (tab), sd	1.17	-	-	Morselli 1975 [35]
400	po (tab), sd	1.14	-	1.28	Pynnoenen 1977 [36]
400	po (tab), sd	1.40	-	-	Strandjord 1975 [37]
400	po (tab), sd	1.35	-	-	Wong 1983 [38]
420	po (tab), sd	1.26	-	-	Levy 1975 [15]
420	po (tab, fed), sd	1.30	-	-	Levy 1975 [15]
600	po (tab), sd	1.66	2.41	-	Dalton 1985 [39]
600	po (tab), sd	1.28	2.27	-	Dalton 1985a [40]
600	po (tab), sd	1.32	-	-	Gerardin 1976 [23]
800	po (tab, fed), sd	2.05	-	-	Cotter 1977 [41]
100/200/400	po (tab), bid/qd	1.11	1.63	-	Burstein 2000 [42]
100/200/400	po (tab), bid/qd	1.15	2.19	-	Moller 2001 [43]
200	po (tab), bid/tid	1.41	1.75	-	Eichelbaum 1975 [26]
200	po (tab), bid/tid	1.31	2.37	-	Eichelbaum 1975 [26]
200	po (tab), qd	1.15	-	-	Gerardin 1976 [23]
200/400	po (tab), bid/qd	1.15	1.2	-	Ji 2008 [44]
357	po (tab), qd	1.09	1.12	-	Miles 1989 [45]
600	po (tab), sd (D1,D5)	1.19	2.00	-	Bernus 1994 [46]
400	po (tab XR), sd	1.11	-	-	Graf 1990 [47]
400	po (tab XR), sd	1.55	2.72	-	Licht 2005 [48]
400	po (tab XR), sd	1.38	-	-	Kovacevic 2009 [34]
400	po (tab XR, fed), sd	2.08	-	-	Kshirsagar 2014 [49]

bid: twice daily, cap: capsule, CBZ: carbamazepine, CBZE: carbamazepine-10,11-epoxide, D: day, iv: intravenous, MRD: mean relative deviation, po: oral, qd: once daily, sd: single dose, sol: solution, susp: suspension, tab: tablet, tid: three times daily, XR: extended release

Table S3: Mean relative deviation values of predicted carbamazepine and carbamazepine-10,11-epoxide plasma and saliva concentrations *(continued)*

Dose [mg]	Route	CBZ MRD	CBZE MRD	CBZ (saliva) MRD	Reference
600	po (tab XR), sd	1.15	-	-	Licht 2005 [48]
400	po (tab XR), bid	1.15	1.04	-	Stevens 1998 [50]
300	po (cap XR), sd	1.57	3.24	-	Gande 2009 [51]
400	po (cap XR), sd	1.10	1.83	-	McLean 2001 [16]
400	po (cap XR, fed), sd	1.13	1.45	-	McLean 2001 [16]
400	po (cap XR), bid	1.03	1.16	-	Stevens 1998 [50]
**mean MRD (range)**		**1.38 (1.03-3.98)**	**1.90 (1.04-3.47)**	**1.31 (1.28-1.35)**	
		**52/55 with MRD ≤ 2**	**14/23 with MRD ≤ 2**	**3/3 with MRD ≤ 2**	

bid: twice daily, cap: capsule, CBZ: carbamazepine, CBZE: carbamazepine-10,11-epoxide, D: day, iv: intravenous, MRD: mean relative deviation, po: oral, qd: once daily, sd: single dose, sol: solution, susp: suspension, tab: tablet, tid: three times daily, XR: extended release

2.5.3 AUC_{last} and C_{max} goodness-of-fit plots

(a) (b)

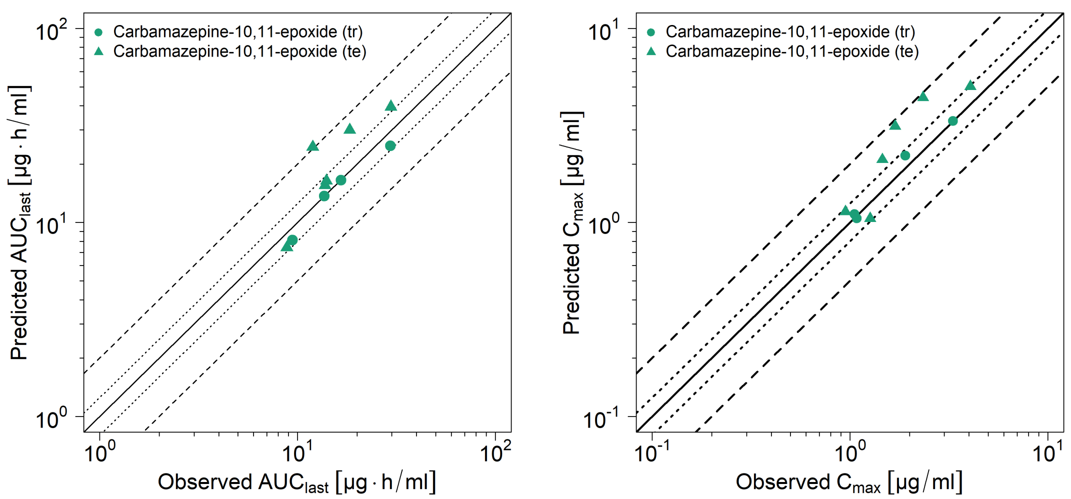

Figure S10: Predicted compared to observed carbamazepine-10,11-epoxide (a) AUC_{last} and (b) C_{max} values after oral administration of carbamazepine-10,11-epoxide. The solid line marks the line of identity. Dotted lines indicate 1.25-fold, dashed lines indicate 2-fold deviation. AUC_{last}: area under the plasma concentration-time curve from the time of drug administration to the last concentration measurement, C_{max}: maximum plasma concentration

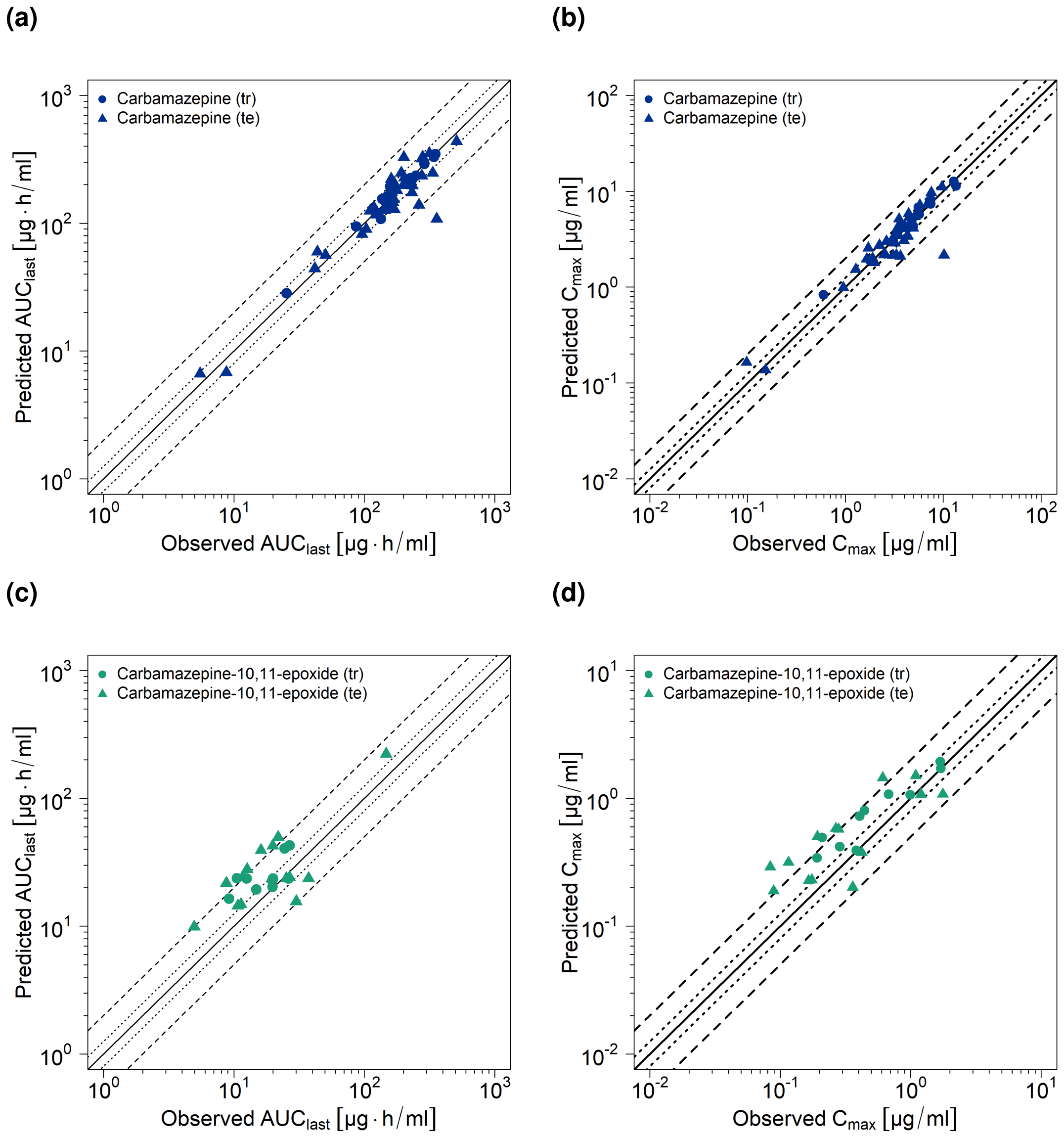

Figure S11: Predicted compared to observed (a,c) AUC_{last} and (b,d) C_{max} values of carbamazepine and carbamazepine-10,11-epoxide after intravenous and oral administration of carbamazepine. The solid line marks the line of identity. Dotted lines indicate 1.25-fold, dashed lines indicate 2-fold deviation. AUC_{last}: area under the plasma concentration-time curve from the time of drug administration to the last concentration measurement, C_{max}: maximum plasma concentration

2.5.4 Geometric mean fold error of predicted AUC_{last} and C_{max} values

Table S4: Predicted and observed AUC_{last} and C_{max} values with geometric mean fold errors of carbamazepine and carbamazepine-10,11-epoxide

		AUC_{last} [µg*h/ml]			C_{max} [µg/ml]			
**Dose [mg]**	**Route**	**Pred**	**Obs**	**Pred/Obs**	**Pred**	**Obs**	**Pred/Obs**	**Reference**
**Carbamazepine-10,11-epoxide administration**								
50	po (susp), sd	8.78	9.43	0.93	1.22	1.05	1.16	Tomson 1983 [5]
50	po (susp), sd	8.03	8.84	0.91	1.13	1.26	0.90	Tomson 1983 [5]
100	po (susp), sd	17.91	16.57	1.08	2.51	1.90	1.32	Tomson 1983 [5]
100	po (susp), sd	25.98	11.94	2.18	3.24	1.69	1.92	Tomson 1983 [5]
100	po (susp), sd	16.79	13.79	1.22	2.29	1.46	1.57	Tomson 1983 [5]
150	po (sol), sd	29.94	18.36	1.63	4.40	2.34	1.88	Sumi 1987 [17]
200	po (susp), sd	26.83	29.47	0.91	3.59	3.32	1.08	Tomson 1983 [5]
200	po (susp), sd	42.20	29.66	1.42	5.37	4.06	1.32	Tomson 1983 [5]
100	po (tab), sd	13.70	13.65	1.00	1.05	1.08	0.97	Pisani 1990 [18]
100	po (tab), sd	16.40	14.01	1.17	1.14	0.95	1.20	Pisani 1992 [19]
**mean GMFE (range)**				**1.29 (1.00-2.18)**			**1.35 (1.03-1.92)**	
				**9/10 with GMFE ≤ 2**			**10/10 with GMFE ≤ 2**	
**Carbamazepine administration**								
Carbamazepine								
10	iv (2 h), sd	6.64	5.51	1.21	0.16	0.10	1.68	Gerardin 1990 [81]
10	iv (2 h), sd	6.81	8.75	0.78	0.14	0.15	0.90	Gerardin 1990 [81]
50	po (sol), sd	28.32	25.33	1.12	0.83	0.60	1.39	Rawlins 1975 [21]
100	po (susp), sd	64.36	68.95	0.93	1.33	1.47	0.91	Gerardin 1990 [81]
100	po (susp), sd	66.14	78.08	0.85	1.38	1.49	0.93	Gerardin 1990 [81]
100	po (sol), sd	59.64	43.65	1.37	1.53	1.27	1.20	Rawlins 1975 [21]
200	po (susp), sd	128.67	157.26	0.82	3.06	3.95	0.77	Eichelbaum 1985 [4]
200	po (sol), sd	116.19	121.69	0.95	3.15	3.02	1.04	Rawlins 1975 [21]
200	po (sol), sd	108.22	134.16	0.81	4.22	4.53	0.93	Sumi 1987 [17]
200	po (susp), sd	133.02	118.44	1.12	3.47	4.32	0.80	Tomson 1983 [5]
200	po (susp), sd	89.8	103.48	0.87	3.49	3.49	1.00	Wada 1978 [22]
100	po (tab), sd	56.18	50.42	1.11	0.99	0.95	1.04	Gerardin 1976 [23]
200	po (tab), sd	128.13	170.03	0.75	2.17	3.32	0.65	Bedada 2015 [24]

bid: twice daily, cap: capsule, D: day,. GMFE: geometric mean fold error, iv: intravenous, po: oral, qd: once daily, sd: single dose, sol: solution, susp: suspension, tab: tablet, tid: three times daily, XR: extended release

Table S4: Predicted and observed AUC_{last} and C_{max} values with geometric mean fold errors of carbamazepine and carbamazepine-10,11-epoxide *(continued)*

		AUC_{last} [µg*h/ml]			C_{max} [µg/ml]			
**Dose [mg]**	**Route**	**Pred**	**Obs**	**Pred/Obs**	**Pred**	**Obs**	**Pred/Obs**	**Reference**
200	po (tab), sd	128.11	160.04	0.80	2.15	3.05	0.70	Bedada 2016 [25]
200	po (tab), sd	126.04	140.33	0.90	1.80	1.98	0.91	Eichelbaum 1975 [26]
200	po (tab), sd	124.99	110.69	1.13	1.96	1.65	1.19	Gerardin 1976 [23]
200	po (tab), sd	138.97	262.77	0.53	2.10	3.64	0.58	Kim 2005 [27]
200	po (tab), sd	119.95	122.94	0.98	1.91	1.77	1.08	Meyer 1992 [28]
200	po (tab), sd	44.2	41.66	1.06	2.00	1.89	1.05	Meyer 1998 [29]
200	po (tab), sd	107.98	359.4	0.30	2.15	10.16	0.21	Shahzadi 2011 [30]
200	po (tab), sd	126.94	143.96	0.88	2.18	2.56	0.85	Saint-Salvi 1987 [31]
200	po (tab), sd	82.24	96.00	0.86	2.17	2.47	0.88	Wada 1978 [22]
400	po (tab, fed), sd	234.54	276.37	0.85	5.44	5.12	1.06	Barzaghi 1987 [32]
400	po (tab, fed), sd	200.37	228.97	0.88	5.20	4.87	1.07	Bianchetti 1987 [33]
400	po (tab), sd	206.67	212.95	0.97	3.72	3.34	1.11	Faigle 1975 [8]
400	po (tab), sd	198.00	198.57	1.00	3.45	3.34	1.04	Faigle 1975 [8]
400	po (tab), sd	226.83	202.93	1.12	4.10	3.73	1.10	Kovacevic 2009 [34]
400	po (tab), sd	146.33	165.79	0.88	4.12	4.94	0.83	Morselli 1975 [35]
400	po (tab), sd	205.58	227.06	0.91	4.33	4.96	0.87	Pynnoenen 1977 [36]
400	po (tab), sd	207.00	168.63	1.23	4.42	3.54	1.25	Strandjord 1975 [37]
400	po (tab), sd	173.19	231.79	0.75	3.39	4.32	0.78	Wong 1983 [38]
420	po (tab), sd	211.66	216.88	0.98	4.48	4.64	0.97	Levy 1975 [15]
420	po (tab, fed), sd	221.80	233.35	0.95	5.74	5.69	1.01	Levy 1975 [15]
600	po (tab), sd	327.24	200.72	1.63	5.13	3.53	1.46	Dalton 1985 [39]
600	po (tab), sd	351.54	315.78	1.11	5.36	4.49	1.20	Dalton 1985a [40]
600	po (tab), sd	329.88	280.14	1.18	5.83	4.42	1.32	Gerardin 1976 [23]
800	po (tab, fed), sd	436.96	510.2	0.86	11.10	9.69	1.15	Cotter 1977 [41]
100/200/400	po (tab), bid/qd	155.41	142.38	1.09	7.42	7.09	1.05	Burstein 2000 [42]
100/200/400	po (tab), bid/qd	155.17	137.49	1.13	7.42	7.17	1.04	Moller 2001 [43]
200	po (tab), bid/tid	320.59	276.79	1.16	8.74	7.40	1.18	Eichelbaum 1975 [26]
200	po (tab), bid/tid	247.34	191.72	1.29	7.17	5.77	1.24	Eichelbaum 1975 [26]
200	po (tab), qd	94.53	86.57	1.09	1.90	1.83	1.04	Gerardin 1976 [23]
200	po (tab), qd	193.6	157.84	1.23	4.25	3.61	1.18	Gerardin 1976 [23]

bid: twice daily, cap: capsule, D: day,. GMFE: geometric mean fold error, iv: intravenous, po: oral, qd: once daily, sd: single dose, sol: solution, susp: suspension, tab: tablet, tid: three times daily, XR: extended release

Table S4: Predicted and observed AUC_{last} and C_{max} values with geometric mean fold errors of carbamazepine and carbamazepine-10,11-epoxide *(continued)*

		AUC_{last} [µg*h/ml]			C_{max} [µg/ml]			
**Dose [mg]**	**Route**	**Pred**	**Obs**	**Pred/Obs**	**Pred**	**Obs**	**Pred/Obs**	**Reference**
200/400	po (tab), bid/qd	154.51	142.44	1.08	7.35	7.47	0.98	Ji 2008 [44]
357	po (tab), qd	165.9	150.75	1.10	7.76	7.13	1.09	Miles 1989 [45]
600	po (tab), sd (D1)	330.92	341.99	0.97	6.66	5.64	1.18	Bernus 1994 [46]
600	po (tab), sd (D5)	346.93	350.82	0.99	6.79	5.59	1.22	Bernus 1994 [46]
400	po (tab XR), sd	181.03	177.3	1.02	3.00	2.63	1.14	Graf 1990 [47]
400	po (tab XR), sd	196.87	233.60	0.84	2.73	2.22	1.23	Licht 2005 [48]
400	po (tab XR), sd	154.75	167.70	0.92	2.89	3.21	0.90	Kovacevic 2009 [34]
400	po (tab XR, fed), sd	246.84	335.01	0.74	4.31	4.07	1.06	Kshirsagar 2014 [49]
600	po (tab XR), sd	291.55	288.8	1.01	4.07	3.55	1.15	Licht 2005 [48]
400	po (tab XR), bid	132.14	151.81	0.87	11.33	13.49	0.84	Stevens 1998 [50]
300	po (cap XR), sd	173.88	161.97	1.07	2.56	1.71	1.49	Gande 2009 [51]
400	po (cap XR), sd	225.08	225.12	1.00	3.02	3.07	0.98	McLean 2001 [16]
400	po (cap XR, fed), sd	236.09	249.01	0.95	4.27	4.09	1.05	McLean 2001 [16]
400	po (cap XR), bid	146.45	145.39	1.01	12.63	12.82	0.98	Stevens 1998 [50]
**mean GMFE (range)**				**1.20 (1.00-3.33)**			**1.24 (1.00-4.72)**	
				**58/59 with GMFE ≤ 2**			**58/59 with GMFE ≤ 2**	
Carbamazepine-10,11-epoxide								
200	po (tab), sd	14.43	10.72	1.35	0.23	0.16	1.37	Bedada 2015 [24]
200	po (tab), sd	14.83	11.32	1.31	0.23	0.17	1.31	Bedada 2016 [25]
200	po (tab), sd	9.89	4.97	1.99	0.19	0.09	2.12	Eichelbaum 1975 [26]
200	po (tab), sd	15.61	30.07	0.52	0.20	0.36	0.56	Kim 2005 [27]
600	po (tab), sd	39.26	16.11	2.44	0.57	0.28	2.05	Dalton 1985 [39]
600	po (tab), sd	42.57	19.85	2.14	0.58	0.27	2.18	Dalton 1985a [40]
400	po (tab, fed), sd	27.88	12.63	2.21	0.50	0.19	2.60	Barzaghi 1987 [32]
400	po (tab, fed), sd	23.76	10.54	2.25	0.49	0.21	2.35	Bianchetti 1987 [33]
400	po (tab), sd	23.67	26.17	0.90	0.39	0.38	1.02	Faigle 1975 [8]
400	po (tab), sd	23.63	25.20	0.94	0.38	0.42	0.89	Faigle 1975 [8]
100/200/400	po (tab), bid/qd	23.67	37.37	0.63	1.08	1.77	0.61	Burstein 2000 [42]
100/200/400	po (tab), bid/qd	23.62	12.51	1.89	1.08	0.68	1.59	Moller 2001 [43]
200	po (tab), bid/tid	222.76	147.11	1.51	1.51	1.09	1.38	Eichelbaum 1975 [26]

bid: twice daily, cap: capsule, D: day,. GMFE: geometric mean fold error, iv: intravenous, po: oral, qd: once daily, sd: single dose, sol: solution, susp: suspension, tab: tablet, tid: three times daily, XR: extended release

Table S4: Predicted and observed AUC_{last} and C_{max} values with geometric mean fold errors of carbamazepine and carbamazepine-10,11-epoxide *(continued)*

		AUC_{last} [µg*h/ml]			C_{max} [µg/ml]			
**Dose [mg]**	**Route**	**Pred**	**Obs**	**Pred/Obs**	**Pred**	**Obs**	**Pred/Obs**	**Reference**
200	po (tab), bid/tid	49.65	21.85	2.27	1.45	0.61	2.37	Eichelbaum 1975 [26]
200/ 400	po (tab), bid/qd	23.63	19.96	1.18	1.07	1.00	1.07	Ji 2008 [44]
357	po (tab), md	24.12	26.72	0.90	1.08	1.20	0.90	Miles 1989 [45]
600	po (tab), sd (D1)	40.6	24.41	1.66	0.73	0.41	1.79	Bernus 1994 [46]
600	po (tab), sd (D5)	43.02	26.72	1.61	0.80	0.44	1.81	Bernus 1994 [46]
400	po (tab XR), sd	24.15	11.68	2.07	0.32	0.12	2.73	Licht 2005 [48]
400	po (tab XR), bid	20.33	19.77	1.03	1.72	1.71	1.01	Stevens 1998 [50]
300	po (cap XR), sd	21.71	8.75	2.48	0.29	0.08	3.47	Gande 2009 [51]
400	po (cap XR), sd	16.44	9.18	1.79	0.34	0.19	1.78	McLean 2001 [16]
400	po (cap XR, fed), sd	19.43	14.85	1.31	0.42	0.29	1.46	McLean 2001 [16]
400	po (cap XR), bid	22.76	19.71	1.15	1.93	1.69	1.14	Stevens 1998 [50]
**mean GMFE (range)**				**1.68 (1.03-2.48)**			**1.76 (1.01-3.47)**	
				**17/24 with GMFE ≤ 2**			**16/24 with GMFE ≤ 2**	

bid: twice daily, cap: capsule, D: day,. GMFE: geometric mean fold error, iv: intravenous, po: oral, qd: once daily, sd: single dose, sol: solution, susp: suspension, tab: tablet, tid: three times daily, XR: extended release

2.5.5 Sensitivity analysis

Sensitivity of the final carbamazepine-10,11-epoxide PBPK model to single parameters (local sensitivity analysis) was calculated as the relative change of the predicted carbamazepine-10,11-epoxide $AUC_{0\text{-}24}$ following a single dose of 100 mg carbamazepine-10,11-epoxide as tablet. Sensitivity analysis was carried out using a relative parameter perturbation of 1000% (variation range 10.0, maximum number of 9 steps). Parameters were included into the analysis if they were optimized (lipophilicity, EPHX1 clearance, GFR fraction, intestinal permeability, dissolution shape, dissolution time) or if they might have a strong impact due to calculation methods used in the model (solubility, fraction unbound in plasma). Results of the sensitivity analysis are illustrated in Figure S12. The carbamazepine-10,11-epoxide model is mainly sensitive to the value fraction unbound in plasma (literature), and to the EPHX1 clearance (optimized).

Sensitivity of the carbamazepine parent-metabolite PBPK model to single parameters (local sensitivity analysis) was calculated as the relative change of the predicted carbamazepine and carbamazepine-10,11-epoxide AUC at steady-state (AUC_{ss}) of 400 mg carbamazepine three times daily as immediate release tablet. Sensitivity analysis was carried out using a relative parameter perturbation of 1000% (variation range 10.0, maximum number of 9 steps). Parameters were included into the analysis if they were optimized (lipophilicity, CYP3A4 k_{cat} values, carbamazepine hepatic clearance, EPHX1 clearance, GFR fraction, CYP3A4, CYP2B6 and EPHX1 E_{max} values, intestinal permeability (carbamazepine-10,11-epoxide), dissolution shape, dissolution time), if they are associated with optimized parameters (CYP3A4, CYP2C8, CYP2B6 and UGT2B7 K_m values, CYP2C8, CYP2B6 and UGT2B7 k_{cat} values, CYP3A4, CYP2B6 and EPHX1 EC_{50} values) or if they might have a strong impact due to calculation methods used in the model (solubility, fraction unbound in plasma, intestinal permeability (carbamazepine)). Results of the sensitivity analysis are illustrated in Figure S13. Sensitivity analysis of the carbamazepine parent-metabolite model revealed that the carbamazepine AUC_{ss} is mainly sensitive to the carbamazepine fraction unbound in plasma (literature), while the carbamazepine-10,11-epoxide AUC_{ss} is sensitive to carbamazepine-10,11-epoxide fraction unbound in plasma (literature), EPHX1 clearance of carbamazepine-10,11-epoxide (optimized), K_m and k_{cat} of carbamazepine CYP3A4 metabolism to carbamazepine-10,11-epoxide (literature and optimized, respectively) and carbamazepine EPHX1 E_{max} (optimized).

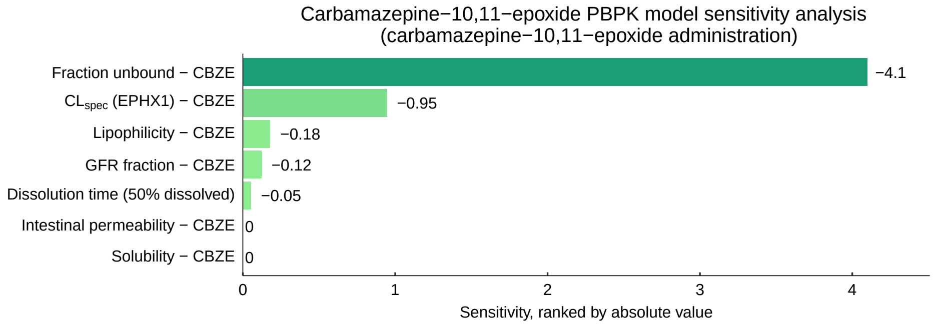

Figure S12: Carbamazepine-10,11-epoxide PBPK model sensitivity analysis. Sensitivity of the carbamazepine-10,11-epoxide model to single parameters, calculated as change of the simulated carbamazepine-10,11-epoxide $AUC_{0\text{-}24}$ following a single dose of 100 mg carbamazepine-10,11-epoxide as tablet. CBZE: carbamazepine-10,11-epoxide, CL_{spec}: specific clearance, EPHX1: epoxide hydroxylase 1, GFR: glomerular filtration rate

(a)

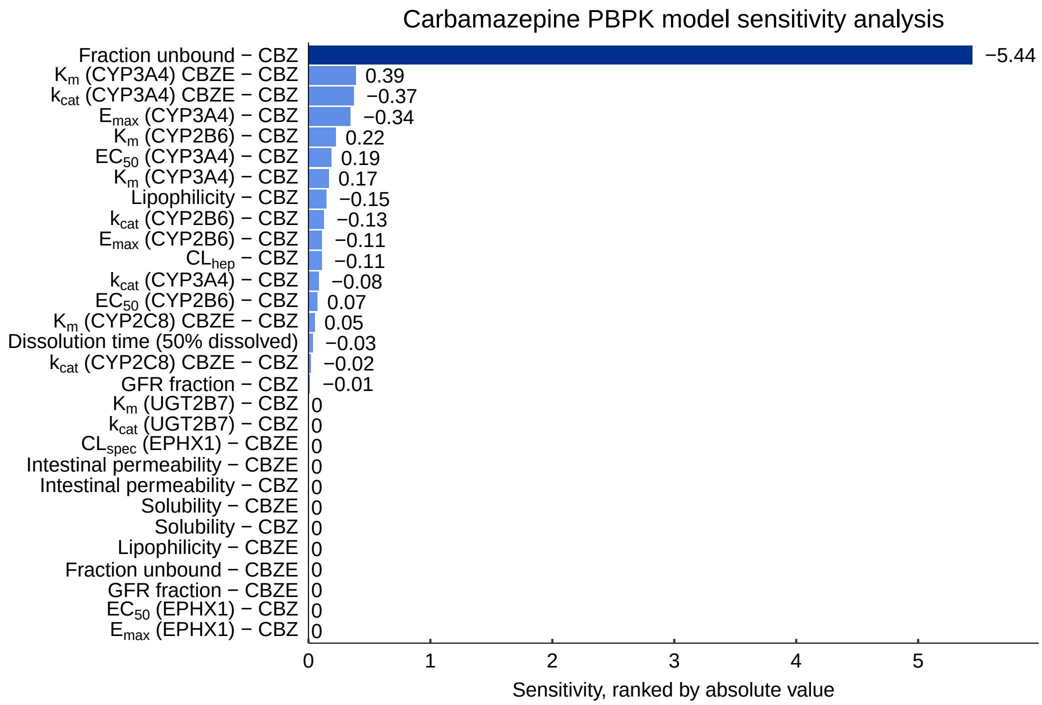

(b)

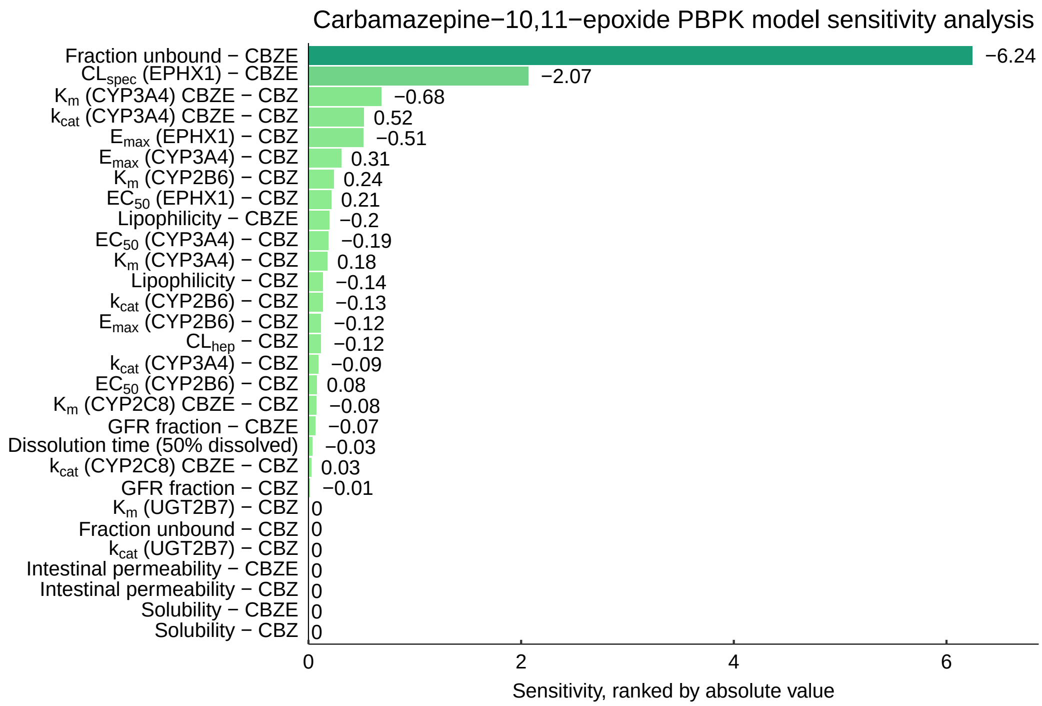

Figure S13: Carbamazepine parent-metabolite PBPK model sensitivity analysis. Sensitivity of the carbamazepine parent-metabolite PBPK model to single parameters, calculated as change of the simulated (a) carbamazepine AUC_{ss} and (b) carbamazepine-10,11-epoxide AUC_{ss} of 400 mg carbamazepine three times daily as immediate release tablet. CBZ: carbamazepine, CBZE: carbamazepine-10,11-epoxide, CL_{hep}: hepatic clearance, CL_{spec}: specific clearance, CYP: cytochrome P450, EC_{50}: half-maximal effective concentration, E_{max}: maximum effect, EPHX1: epoxide hydroxylase 1, GFR: glomerular filtration rate, k_{cat}: catalytic rate constant, K_m: Michaelis-Menten constant, UGT: UDP-glucuronosyltransferase

3 Efavirenz

3.1 PBPK model building

The antiretroviral drug efavirenz is a non-nucleoside reverse transcriptase inhibitor and used for the treatment of human immunodeficiency virus infections [82]. Its major metabolizing enzyme is CYP2B6, but CYP3A4, CYP3A5, CYP1A2 and CYP2A6 are also involved in efavienz metabolism [83, 84]. By activation of nuclear receptors (PXR and CAR), efavirenz increases the expression of CYP2B6 and CYP3A4. As a consequence, efavirenz induces its own metabolism and the metabolism of other drugs after multiple-dose administration [66, 85, 86]. The drug is classified by the FDA as moderate inducer (50-80% AUC decrease of the victim drug) of CYP2B6 and CYP3A4, and as moderately sensitive substrate of CYP2B6 [10].

CYP2B6 polymorphisms are a major determinant of clinical efavirenz disposition. CYP2B6 is highly polymorphic with more than 30 known alleles. The most frequent decreased function allele is CYP2B6*6. Heterozygosity (CYP2B6*1|*6, intermediate metabolizer (IM)) and homozygosity (CYP2B6*6|*6, poor metabolizer (PM)) for this allele are associated with elevated efavirenz plasma levels caused by decreased efavirenz metabolism. Little to no auto-induction was observed for CYP2B6*6|*6 [86].

The efavirenz PBPK model was downloaded from the Open Systems Pharmacology (OSP) GitHub model repository (https://github.com/Open-Systems-Pharmacology/Efavirenz-Model). This model includes (1) metabolism by CYP2B6 (2) metabolism via CYP1A2, CYP2A6, CYP3A4 and CYP3A5 and (3) glomerular filtration [87]. All metabolic processes are illustrated in Figure S14.

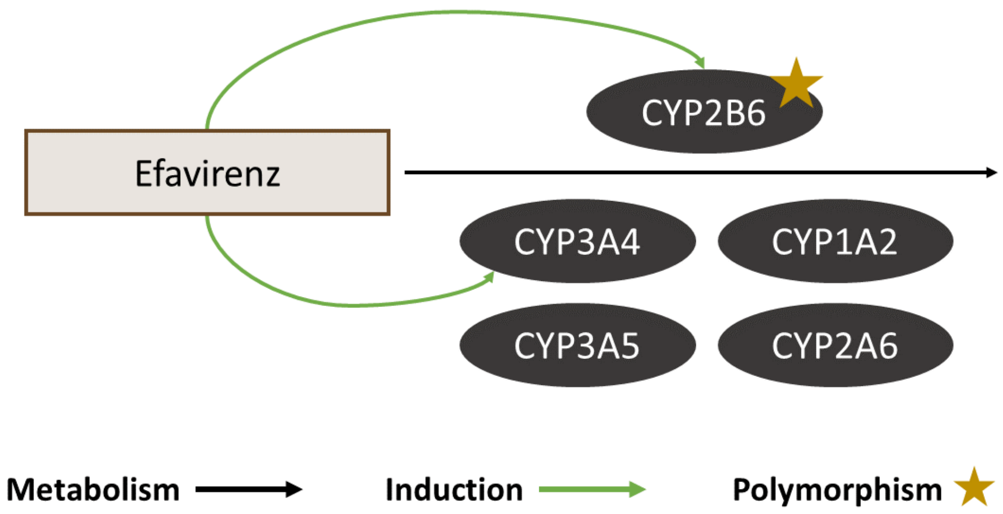

Figure S14: Metabolic pathways of efavirenz. Efavirenz is metabolized by CYP1A2, CYP2A6, CYP3A4, CYP3A5 and CYP2B6 (black arrow), while the latter is the main enzyme catalyzing efavirenz metabolism. Therefore, polymorphisms of CYP2B6 are a major determinant of efavirenz pharmacokinetics. Furthermore, efavirenz induces its own metabolism by induction of CYP2B6 and CYP3A4 (green arrows)

Prior to predicting the carbamazepine-efavirenz interaction, the DDI potential of efavirenz as a CYP2B6 substrate and inducer was evauated, using data of clinical DDI studies of efavirenz with rifampin, voriconazole (perpetrators) and bupropion (victim) as interaction partners. Based on this evaluation, the efavirenz model was updated using plasma concentration-time profiles of 13 publications, covering a dosing range of 50-600 mg administered in single- or multiple-dose regimens. All studies used are listed in Table S5.

All metabolic and inductive processes were retained, but literature values for Michaelis-Menten constants - used to describe enzymatic metabolism - and EC_{50} values - used to describe enzyme induction - were corrected for unspecific binding of efavirenz in microsomal preparations ($fu_{incubation}$). If no $fu_{incubation}$ was reported for the in vitro assay, it was calculated according to Equation S12 [55], using logP and microsomal protein concentration (C_{mic}). If no microsomal protein concentration was reported for the in vitro assay, a low protein concentration of 0.25 mg/ml was assumed.

$$fu_{incubation} = \frac{1}{C_{mic} * 10^{0.56*logP-1.41} + 1} \tag{S12}$$

where C_{mic} = microsomal protein concentration [mg/ml] and $logP$ = lipophilicity of the molecule.

Furthermore, a competitive inhibition of CYP2B6 and CYP3A4 was implemented in the model, according to literature [74, 88], using in vitro values that were also corrected for $fu_{incubation}$. Drug-dependent parameters of efavirenz used in the model, compared to literature values and the values used in the original model, are listed in Table S6.

The good descriptive and predictive performance of the updated efavirenz model is demonstrated in linear (Figure S15) as well as semi-logarithmic plots (Figure S16) of predicted compared to observed plasma concentration-time profiles of all clinical studies. Goodness-of-fit plots comparing all predicted to their corresponding observed plasma concentrations are presented in Figure S17 and corresponding MRD values for each study are given in Table S7. Furthermore, the correlation of predicted to observed AUC_{last} and C_{max} values is shown in Figure S18 and Table S8 lists the corresponding predicted and observed AUC_{last} and C_{max} values of all clinical studies including calculated GMFE values.

3.2 Efavirenz clinical studies

Table S5: Clinical studies used for the update of the efavirenz PBPK model

Dose [mg]	Route	Dataset	n	Healthy [%]	Females [%]	Age[a] [years]	Weight[a] [kg]	Height[a] [cm]	CYP2B6 genotype	Reference
50	po (tab), sd	training	16	100	0	23.8 (20-35)	23.5 (20.6-27.5)[b]	-	56% IM[c]	Derungs 2015 [89]
200	po (-), qd	training	8	100	4	29.9 (19-49) ± 8.7	-	-	-	Mouly 2002 [90]
400	po (cap), qd	test	34	100	0	(20-49)	(59-95)	-	-	Liu 2008 [91]
400	po (-), qd	training	8	100	4	29.9 (19-49) ± 8.7	-	-	-	Mouly 2002 [90]
600	po (-), sd	test	20	100	50	27.5 (19-44)	72.9 (57-88)	-	40% EM[c]<br>45% IM[c]<br>15% PM[c]	Cho 2016 [92]
600	po (-), sd	test	5	100	-	-	-	-	-	Ogburn 2010 [84]
600	po (-), sd	training	8	100	50	-	-	-	EM[c]	Xu 2013 [93]
600	po (-), sd	test	9	100	50	-	-	-	IM[c]	Xu 2013 [93]
600	po (-), sd	test	3	100	50	-	-	-	PM[c]	Xu 2013 [93]
600	po (-), qd	training	18	100	8	44[e] (19-62)	82.9[e] (57-119)	-	EM[d]	Dooley 2012 [94]
600	po (-), qd	training	12	100	8	44[e] (19-62)	82.9[e] (57-119)	-	IM[d]	Dooley 2012 [94]
600	po (-), qd	training	3	100	8	44[e] (19-62)	82.9[e] (57-119)	-	PM[d]	Dooley 2012 [94]
600	po (-), qd	test	16	100	0	29.6 (20-42)	24.3 (19-30)[b]	-	-	Damle 2008 [95]
600	po (tab), qd	training	21	100	4	24.5[e] (20-52)	26.3[e] (20.4-31.5)[b]	-	-	Garg 2013 [96]
600	po (-), qd	test	12	100	17	36 (24–53)	79.5 (64.7–92.5)	176 (165–192)	-	Huang 2012 [97]
600	po (-), qd	test	36	100	31	(20-45)	(54-92)	-	-	Ji 2008 [44]
600	po (-), qd	test	12	100	50	22 (18-29)	71 (57-96)	-	-	Kharasch 2012 [98]
600	po (-), qd	test	11	100	55	42.6 ± 7.4	76.9 ± 18.9	-	18% EM<br>55% IM<br>27% PM	Kwara 2011 [99]
600	po (tab), qd	test	18	100	29	43 (19-57)	-	-	-	Malvestutto 2014 [100]

-: not given, cap: capsule CYP2B6: cytochrome P450 2B6, EM: extensive metabolizer, IM: intermediate metabolizer, PM: poor metabolizer, po: oral, qd: once daily, sd: single dose, tab: tablet

[a] mean (range) ± standard deviation

[b] body mass index [kg/m^2]

[c] subjects were tested for the diminished-function allele CYP2B6*6

[d] subjects were tested for the diminished-function alleles CYP2B6*6 and CYP2B6*18

[e] median values

Table S5: Clinical studies used for the revision of the efavirenz PBPK model *(continued)*

Dose [mg]	Route	Dataset	n	Healthy [%]	Females [%]	Age[a] [years]	Weight[a] [kg]	Height[a] [cm]	CYP2B6 genotype	Reference
600	po (-), qd	test	12	100	41	(24-49)	(50-83)	-	-	Soon 2010 [101]

-: not given, cap: capsule CYP2B6: cytochrome P450 2B6, EM: extensive metabolizer, IM: intermediate metabolizer, PM: poor metabolizer, po: oral, qd: once daily, sd: single dose, tab: tablet

[a] mean (range) ± standard deviation

[b] body mass index [kg/m^2]

[c] subjects were tested for the diminished-function allele CYP2B6*6

[d] subjects were tested for the diminished-function alleles CYP2B6*6 and CYP2B6*18

[e] median values

3.3 Efavirenz drug-dependent parameters

Table S6: Drug-dependent parameters of the efavirenz PBPK model

Parameter	Unit	Updated model	Original model[a]	Literature[a]	Reference	Description
MW	g/mol	315.68 (Lit)	315.68 (Lit)	315.68	[102]	Molecular weight
logP	log Units	3.87 (Fit)	3.44 (Fit)	2.07, 4.6 (logP)	[102, 103]	Lipophilicity
				5.1 (logD)	[104]	
Solubility	mg/ml	0.10 (pH 7.0) (Fit)	0.039 (pH 0) (Fit)	0.011 (6.4), 0.06 (FaSSIF)	[105, 106]	Solubility
fu	%	0.60 (Lit)	0.60 (Lit)	0.60 (0.4-1.5)	[103]	Fraction unbound in plasma
pKa	-	10.1 (Lit)	10.1 (Lit)	10.1	[107]	Acid dissociation constant
K_m (CYP2B6)	μM	1.54^b (Lit)	6.4 (Lit)	6.4	[83]	CYP2B6 Michaelis-Menten constant
k_{cat} (CYP2B6*1|*1)	1/min	2.84 (Fit)	1.60 (Fit)	-	-	CYP2B6 catalytic rate constant for *1|*1 (EM) genotype
k_{cat} (CYP2B6*1|*6)	1/min	2.27 (Fit)	2.27 (Fit)	-	-	CYP2B6 catalytic rate constant for *1|*6 (IM) genotype
k_{cat} (CYP2B6*6|*6)	1/min	1.45 (Fit)	1.45 (Fit)	-	-	CYP2B6 catalytic rate constant for *6|*6 (PM) genotype
K_m (CYP1A2)	μM	1.99^b (Lit)	8.3 (Lit)	8.3	[83]	CYP1A2 Michaelis-Menten constant
k_{cat} (CYP1A2)	1/min	0.24 (Fit)	0.191 (Fit)	-	-	CYP1A2 catalytic rate constant
K_m (CYP2A6)	μM	7.70 (Lit)	7.70 (Lit)	7.70	[83]	CYP2A6 Michaelis-Menten constant
k_{cat} (CYP2A6)	1/min	0.28 (Fit)	0.318 (Fit)	-	-	CYP2A6 catalytic rate constant
K_m (CYP3A4)	μM	5.64^b (Lit)	23.5 (Lit)	23.5	[83]	CYP3A4 Michaelis-Menten constant
k_{cat} (CYP3A4)	1/min	0.03 (Fit)	0.05 (Fit)	-	-	CYP3A4 catalytic rate constant
K_m (CYP3A5)	μM	4.58^b (Lit)	19.1 (Lit)	19.1	[83]	CYP3A5 Michaelis-Menten constant
k_{cat} (CYP3A5)	1/min	0.11 (Fit)	0.19 (Fit)	-	-	CYP3A5 catalytic rate constant
GFR fraction	-	1	1	-	-	Fraction of filtered drug in the urine
EC_{50} (CYP2B6)	μM	0.23^c (Lit)	0.01 (Fit)	1.62, 1.20	[74, 85]	Concentration for half-maximal induction
E_{max} (CYP2B6)	-	8.13 (Fit)	5.20 (Fit)	6.20, 10.8	[74, 85]	Maximum induction effect
EC_{50} (CYP3A4)	μM	0.23^c (Asm)	0.07 (Fit)	4.59, 3.80, 2.18, 12.5	[66, 74, 85]	Concentration for half-maximal induction
E_{max} (CYP3A4)	-	12.00 (Fit)	5.21 (Fit)	7.27, 3.15, 19.6	[66, 74, 85]	Maximum induction effect
K_i (CYP2B6)	µmol/l	0.40^b (Lit)	-	2.7, 2.96, 0.39, 1.38	[74, 93]	Concentration for half-maximal inhibition
K_i (CYP3A4)	µmol/l	9.67^b (Lit)	-	40.33	[74, 93]	Concentration for half-maximal inhibition

-: not given, asm: assumption, calc: calculated, CYP: cytochrome P450, EM: extensive metabolizer (wildtype), FaSSIF: fasted state simulated intestinal fluid, fit: optimized during parameter identification, GFR: glomerular filtration rate, IM: intermediate metabolizer, lit: literature, PM: poor metabolizer

[a] adopted from [87]

[b] $fu_{incubation}$ = 0.24 was applied to literature value [83], calculated according to [55]

[c] $fu_{incubation}$ = 0.14 was applied to literature value [74], calculated according to [55]

Table S6: Drug-dependent parameters of efavirenz *(continued)*

Parameter	Unit	Updated Model	Original Model[a]	Literature[a]	Reference	Description
Intestinal permeability	cm/min	4.46E-05 (Fit)	2.972E-5 (Fit)	-	-	Transcellular intestinal permeability
Partition coefficients	-	Diverse	Diverse	Schmitt	[108]	Cell to plasma partition coefficients
Cellular permeability	cm/min	1.04	0.39	PK-Sim Standard	[79]	Permeability into the cellular space
Tablet Weibull time	min	44.15 (Fit)	60 (Fit)	-	-	Dissolution time (50% dissolved)
Tablet Weibull shape	-	0.31 (Fit)	0.272 (Fit)	-	-	Dissolution profile shape

-: not given, asm: assumption, calc: calculated, CYP: cytochrome P450, EM: extensive metabolizer (wildtype), FaSSIF: fasted state simulated intestinal fluid, fit: optimized during parameter identification, GFR: glomerular filtration rate, IM: intermediate metabolizer, lit: literature, PM: poor metabolizer

[a] adopted from [87]

[b] $fu_{incubation}$ = 0.24 was applied to literature value [83], calculated according to [55]

[c] $fu_{incubation}$ = 0.14 was applied to literature value [74], calculated according to [55]

3.4 Profiles

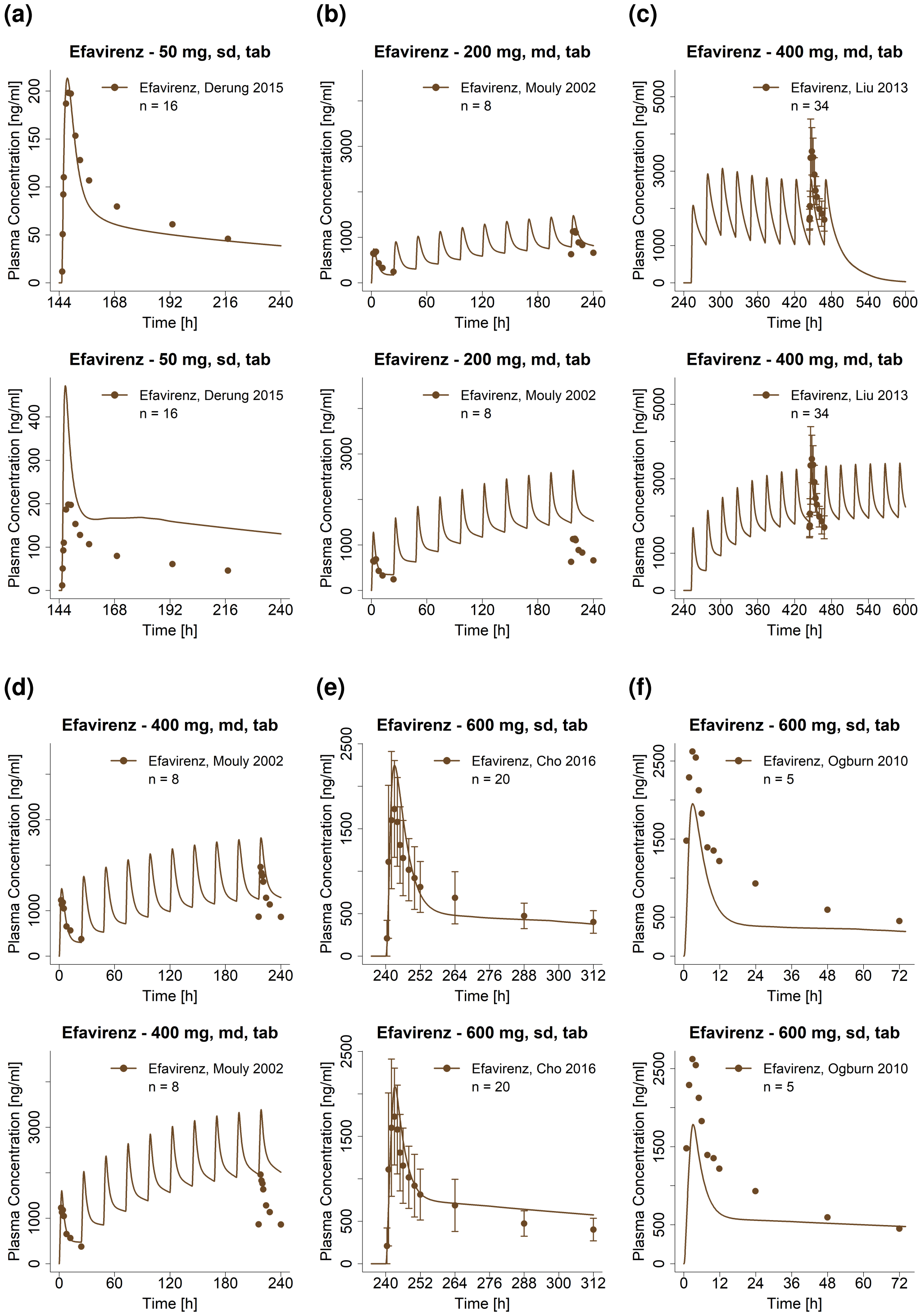

Figure S15: Predicted compared to observed efavirenz plasma concentration-time profiles (linear) predicted with the updated model (upper rows) or the original model (lower rows). Observed data are shown as dots ± standard deviation; model predictions are shown as solid lines. Details on dosing regimens, study populations and literature references are listed in Table S5.EM: extensive metabolizer, IM: intermediate metabolizer, md: multiple-dose, PM: poor metabolizer, tab: tablet, sd: single dose

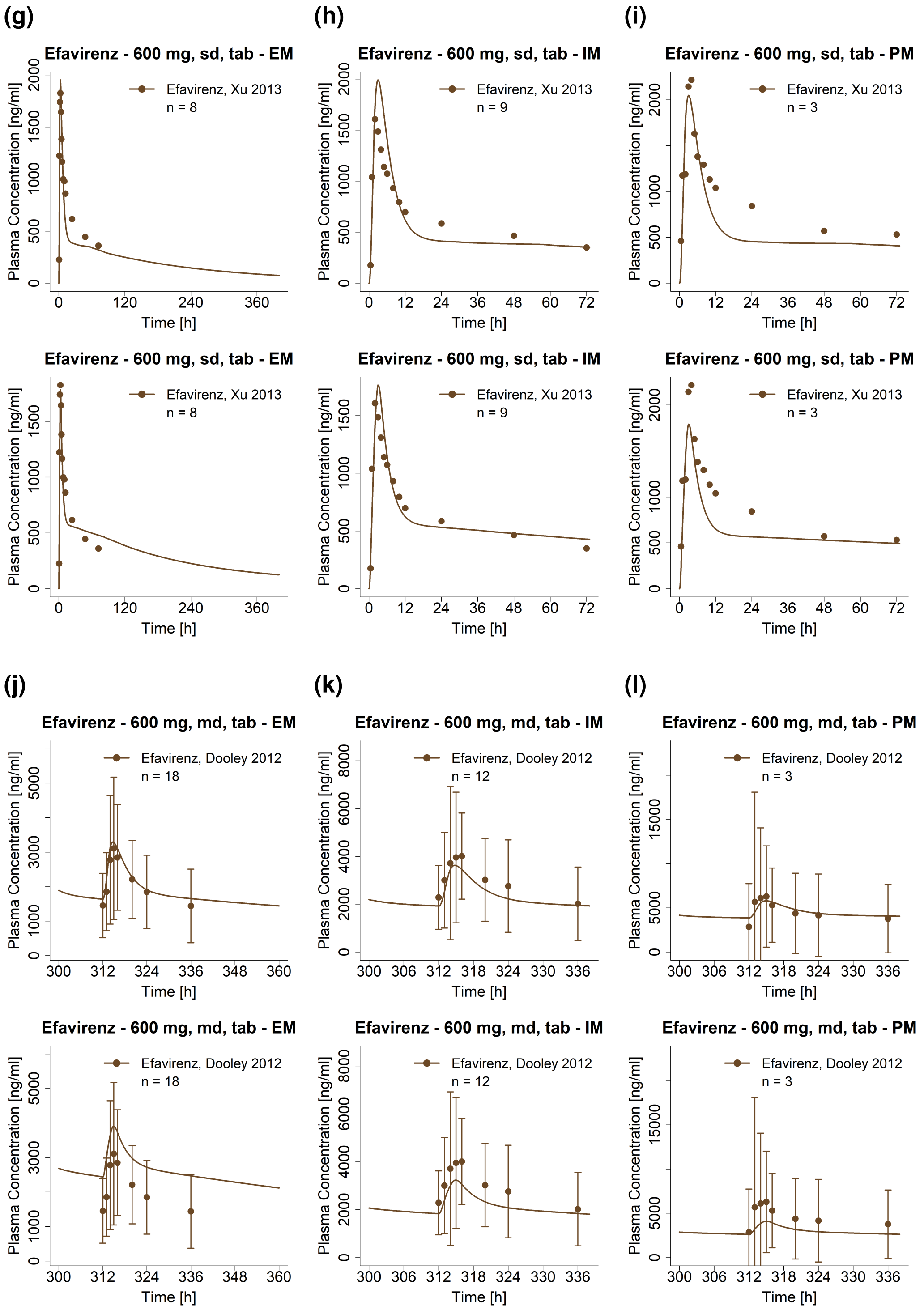

Figure S15: Predicted compared to observed efavirenz plasma concentration-time profiles (linear) predicted with the updated model (upper rows) or the original model (lower rows). Observed data are shown as dots ± standard deviation; model predictions are shown as solid lines. Details on dosing regimens, study populations and literature references are listed in Table S5. EM: extensive metabolizer, IM: intermediate metabolizer, md: multiple-dose, PM: poor metabolizer, tab: tablet, sd: single dose *(continued)*

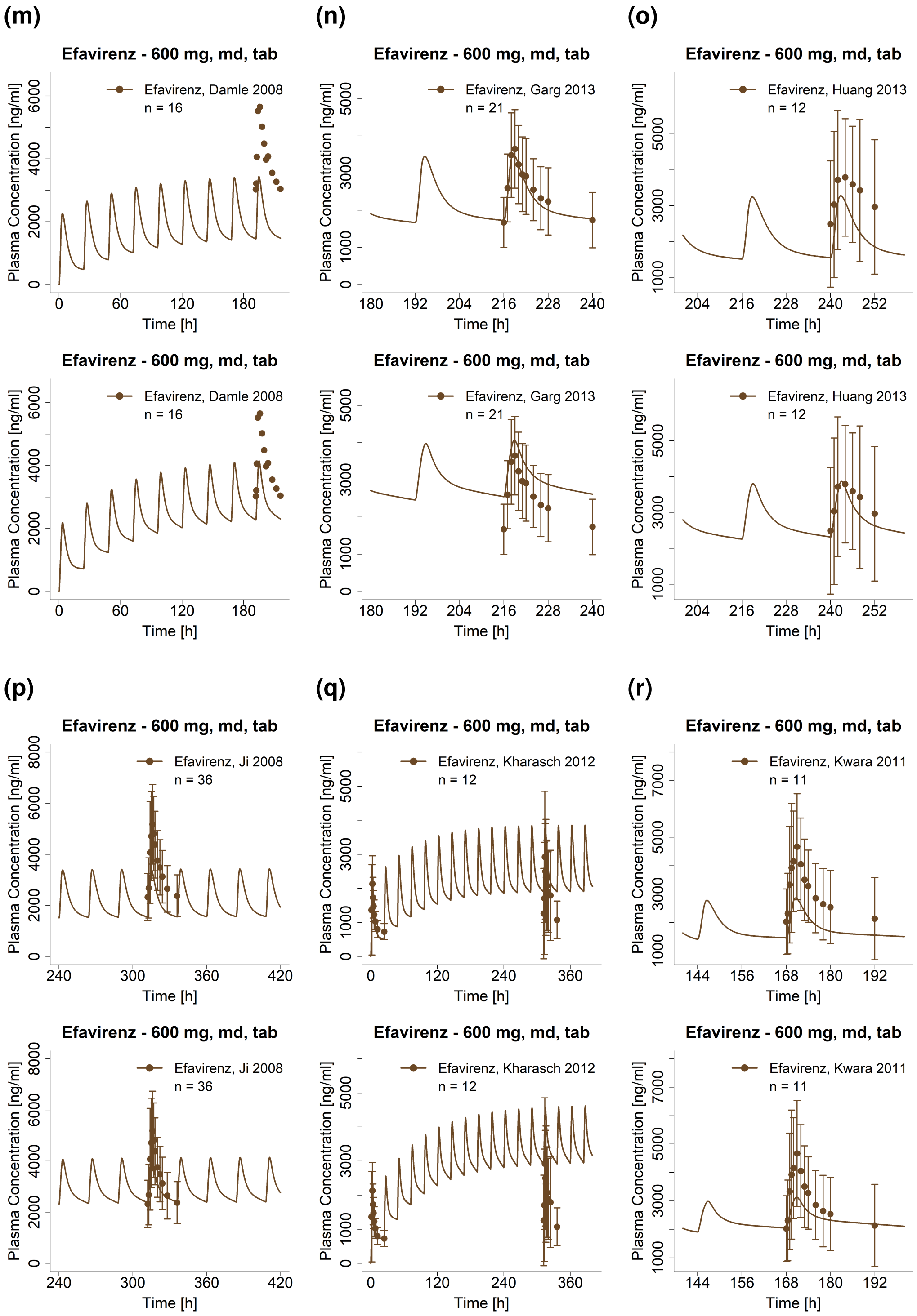

Figure S15: Predicted compared to observed efavirenz plasma concentration-time profiles (linear) predicted with the updated model (upper rows) or the original model (lower rows). Observed data are shown as dots ± standard deviation; model predictions are shown as solid lines. Details on dosing regimens, study populations and literature references are listed in Table S5. EM: extensive metabolizer, IM: intermediate metabolizer, md: multiple-dose, PM: poor metabolizer, tab: tablet, sd: single dose *(continued)*

(s) **(t)**

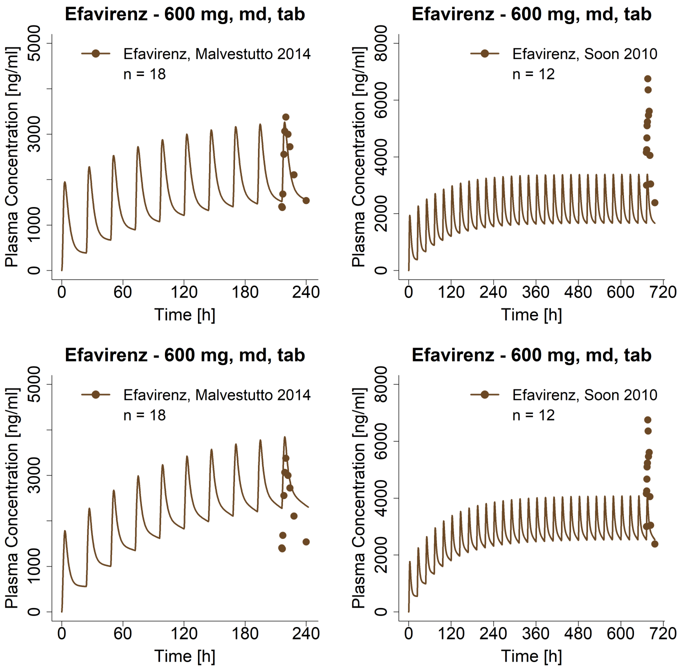

Figure S15: Predicted compared to observed efavirenz plasma concentration-time profiles (linear) predicted with the updated model (upper rows) or the original model (lower rows). Observed data are shown as dots ± standard deviation; model predictions are shown as solid lines. Details on dosing regimens, study populations and literature references are listed in Table S5. EM: extensive metabolizer, IM: intermediate metabolizer, md: multiple-dose, PM: poor metabolizer, tab: tablet, sd: single dose *(continued)*

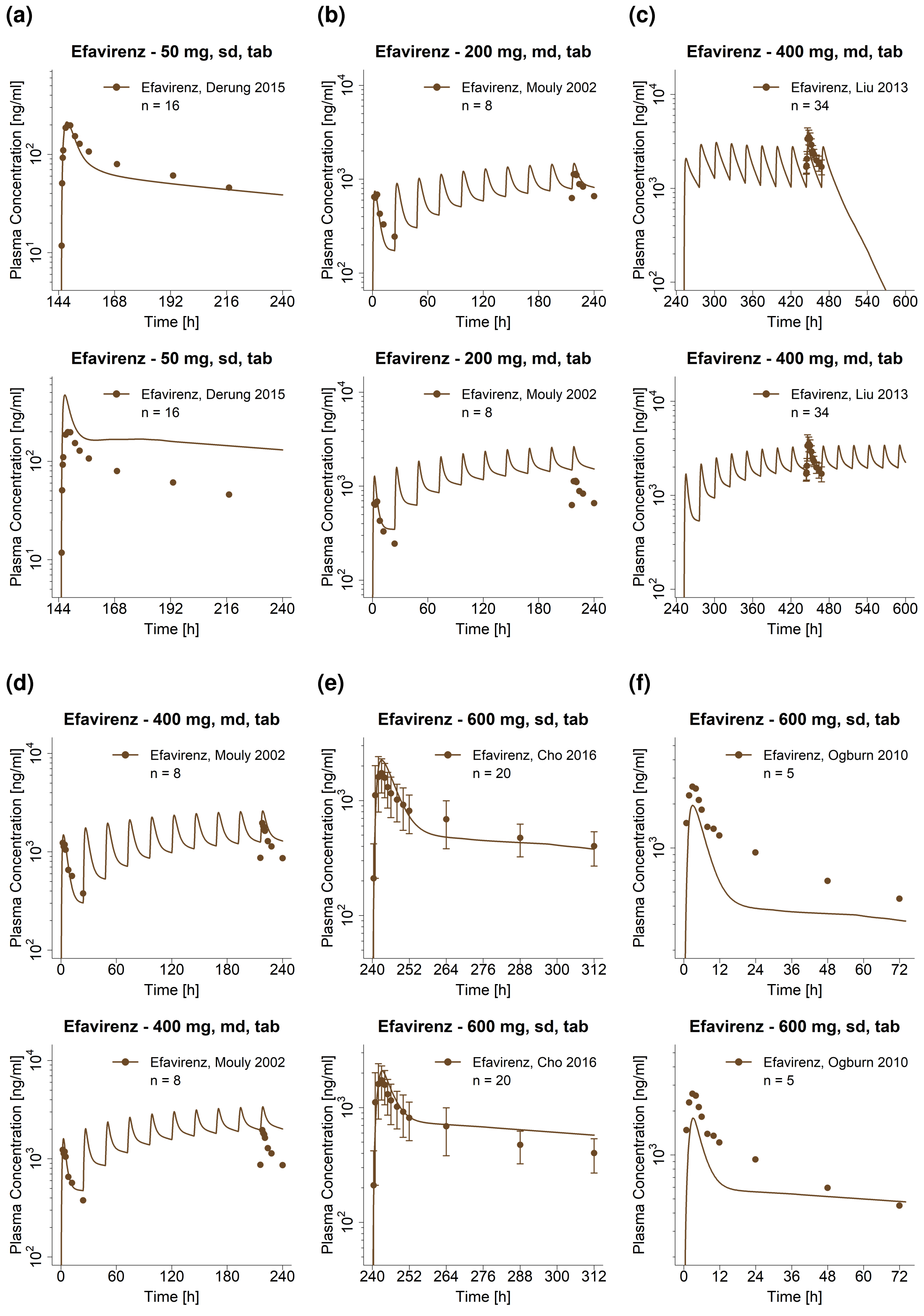

Figure S16: Predicted compared to observed efavirenz plasma concentration-time profiles (semi-logarithmic) predicted with the updated model (upper rows) or the original model (lower rows). Observed data are shown as dots ± standard deviation; model predictions are shown as solid lines. Details on dosing regimens, study populations and literature references are listed in Table S5. EM: extensive metabolizer, IM: intermediate metabolizer, md: multiple-dose, PM: poor metabolizer, tab: tablet, sd: single dose

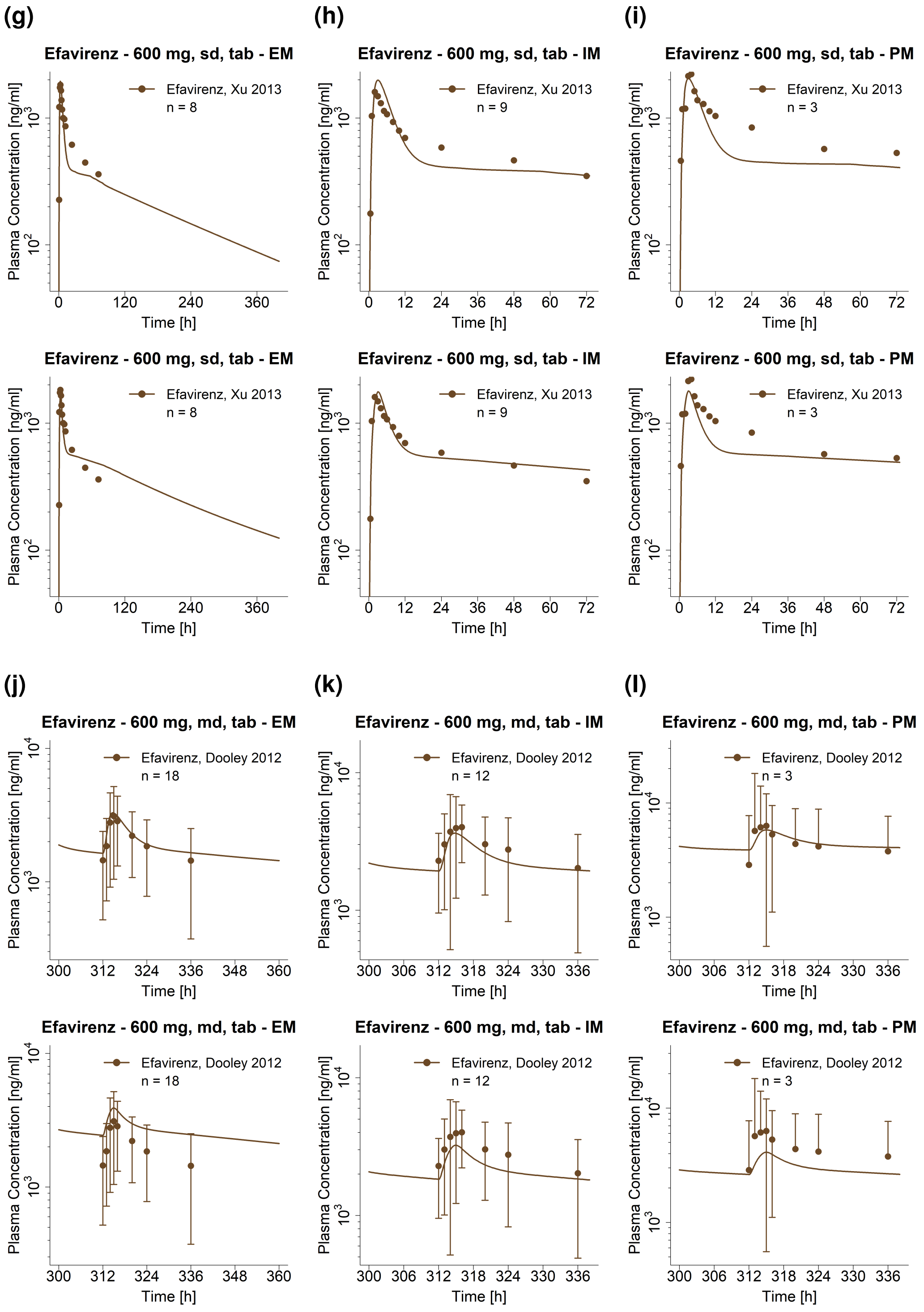

Figure S16: Predicted compared to observed efavirenz plasma concentration-time profiles (semi-logarithmic) predicted with the updated model (upper rows) or the original model (lower rows). Observed data are shown as dots ± standard deviation; model predictions are shown as solid lines. Details on dosing regimens, study populations and literature references are listed in Table S5. EM: extensive metabolizer, IM: intermediate metabolizer, md: multiple-dose, PM: poor metabolizer, tab: tablet, sd: single dose *(continued)*

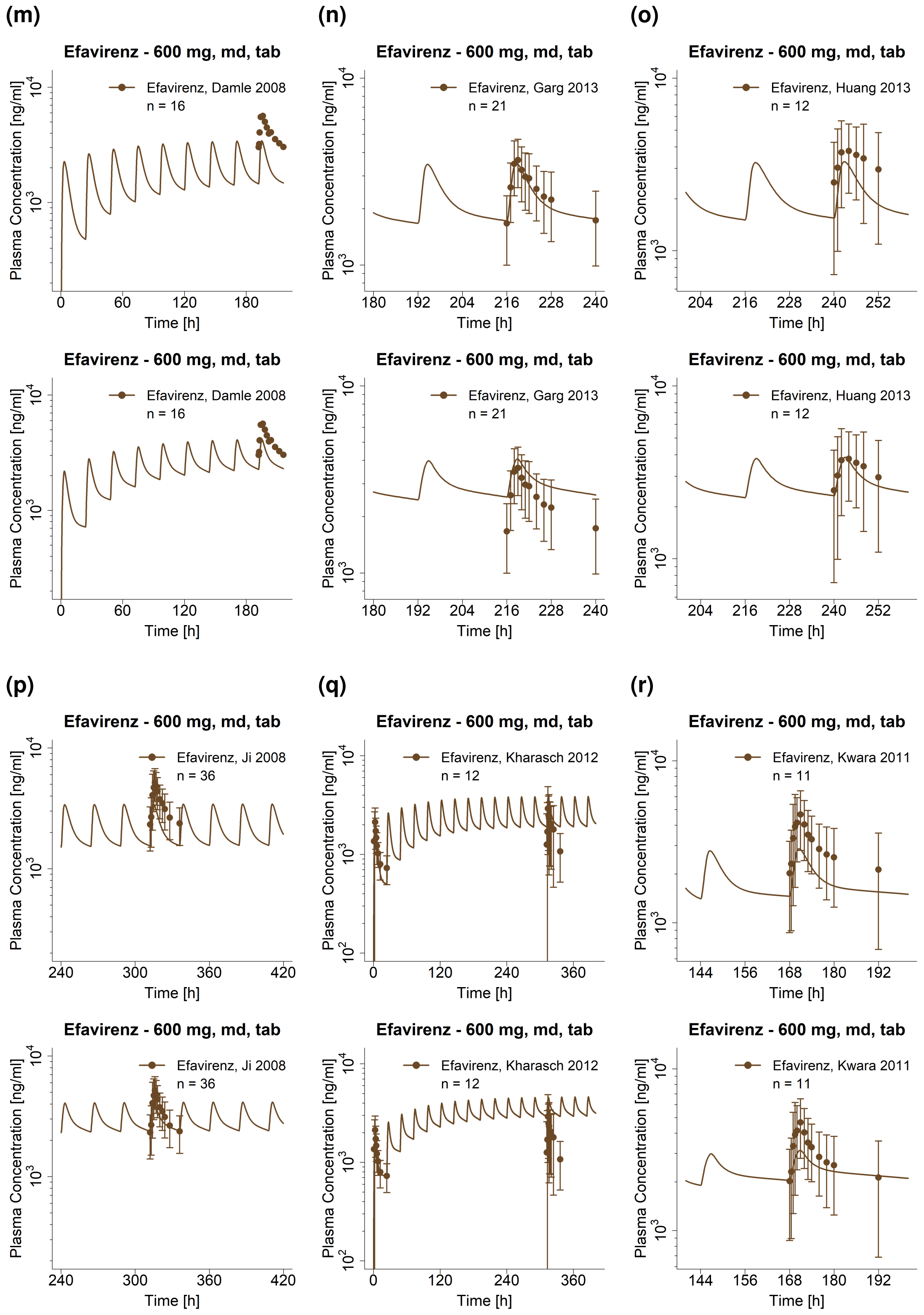

Figure S16: Predicted compared to observed efavirenz plasma concentration-time profiles (semi-logarithmic) predicted with the updated model (upper rows) or the original model (lower rows). Observed data are shown as dots ± standard deviation; model predictions are shown as solid lines. Details on dosing regimens, study populations and literature references are listed in Table S5. EM: extensive metabolizer, IM: intermediate metabolizer, md: multiple-dose, PM: poor metabolizer, tab: tablet, sd: single dose *(continued)*

(s)

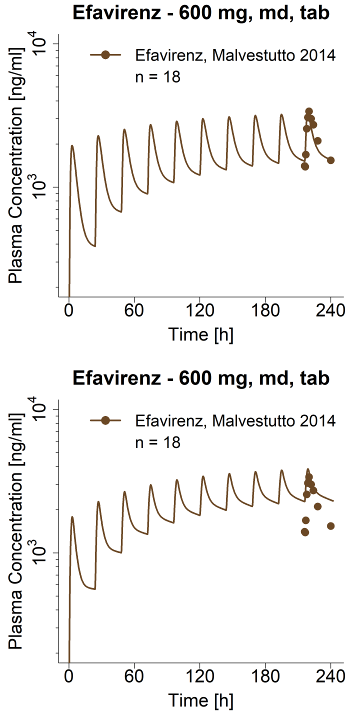

(t)

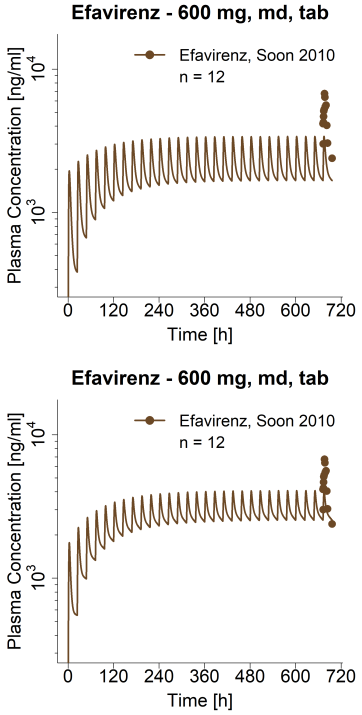

Figure S16: Predicted compared to observed efavirenz plasma concentration-time profiles (semi-logarithmic) predicted with the updated model (upper rows) or the original model (lower rows). Observed data are shown as dots ± standard deviation; model predictions are shown as solid lines. Details on dosing regimens, study populations and literature references are listed in Table S5. EM: extensive metabolizer, IM: intermediate metabolizer, md: multiple-dose, PM: poor metabolizer, tab: tablet, sd: single dose *(continued)*

3.5 Model evaluation

3.5.1 Plasma concentration goodness-of-fit plots

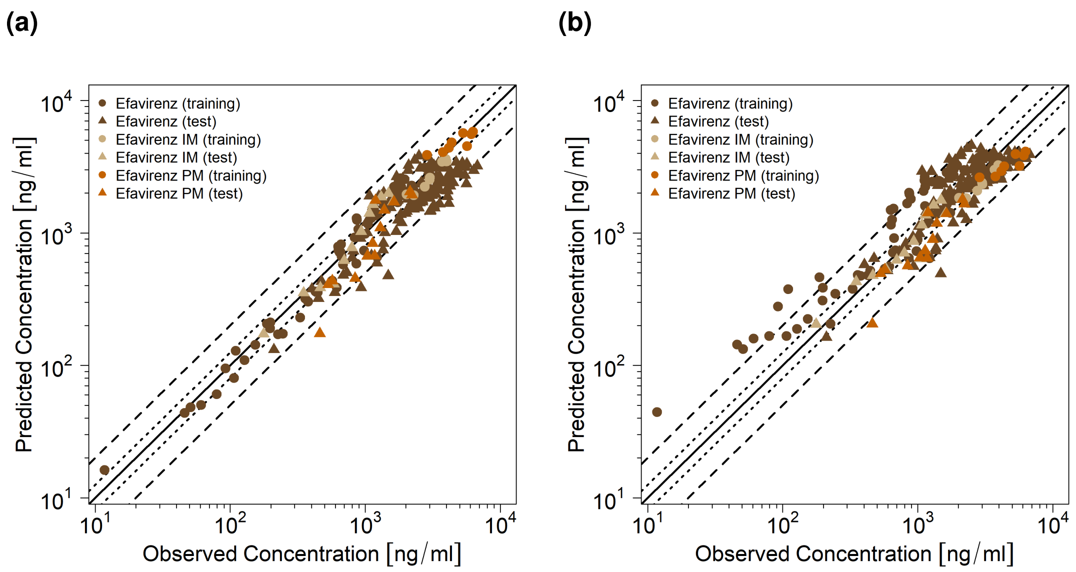

Figure S17: Predicted compared to observed efavirenz plasma concentrations of the training (triangles) and test (circles) datasets. (a) shows the updated model, (b) shows the original efavirenz model goodness-of-fit plot. The solid line marks the line of identity. Dotted lines indicate 1.25-fold, dashed lines indicate 2-fold deviation. IM: intermediate metabolizers (CYP2B6*1|*6), PM: poor metabolizers (CYP2B6*6|*6)

3.5.2 Mean relative deviation of predicted plasma concentrations

Table S7: Mean relative deviation values of predicted efavirenz plasma concentrations of the updated model in comparison to the original model

Route	Dose [mg]	MRD updated	MRD original	Reference
po, sd	50	1.19	2.42	Derungs 2015 [89]
po, qd (D1)	200	1.25	1.47	Mouly 2002 [90]
po, qd (D10)	200	1.23	2.14	Mouly 2002 [90]
po, qd	400	1.39	1.13	Liu 2013 [91]
po, qd (D1)	400	1.20	1.19	Mouly 2002 [90]
po, qd (D10)	400	1.39	1.94	Mouly 2002 [90]
po, sd	600	1.33	1.27	Cho 2016 [92]
po, sd	600	1.78	1.72	Ogburn 2010 [84]
po, sd	600	1.33	1.27	Xu 2013 (EM) [93]
po, sd	600	1.28	1.21	Xu 2013 (IM) [93]
po, sd	600	1.52	1.47	Xu 2013 (PM) [93]
po, qd	600	1.11	1.48	Dooley 2012 (EM) [94]
po, qd	600	1.15	1.26	Dooley 2012 (IM) [94]
po, qd	600	1.16	1.47	Dooley 2012 (PM) [94]
po, qd	600	1.96	1.40	Damle 2008 [95]
po, qd	600	1.06	1.28	Garg 2013 [96]
po, qd	600	1.44	1.09	Huang 2012 [97]
po, qd	600	1.51	1.18	Ji 2008 [44]
po, qd (D1)	600	1.30	1.22	Kharasch 2012 [98]
po, qd (D14)	600	1.48	1.99	Kharasch 2012 [98]
po, qd	600	1.51	1.26	Kwara 2011 [99]
po, qd	600	1.16	1.42	Malvestutto 2014 [100]
po, qd	600	1.89	1.46	Soon 2010 [101]
**mean MRD (range)**		**1.38 (1.06-1.96)**	**1.47 (1.09-2.42)**	
		**23/23 with MRD ≤ 2**	**21/23 with MRD ≤ 2**	

D: day, EM: extensive metabolizer, IM: intermediate metabolizer, MRD: mean relative deviation, po: oral, PM: poor metabolizer, qd: once daily, sd: single dose

3.5.3 AUC_{last} and C_{max} goodness-of-fit plots

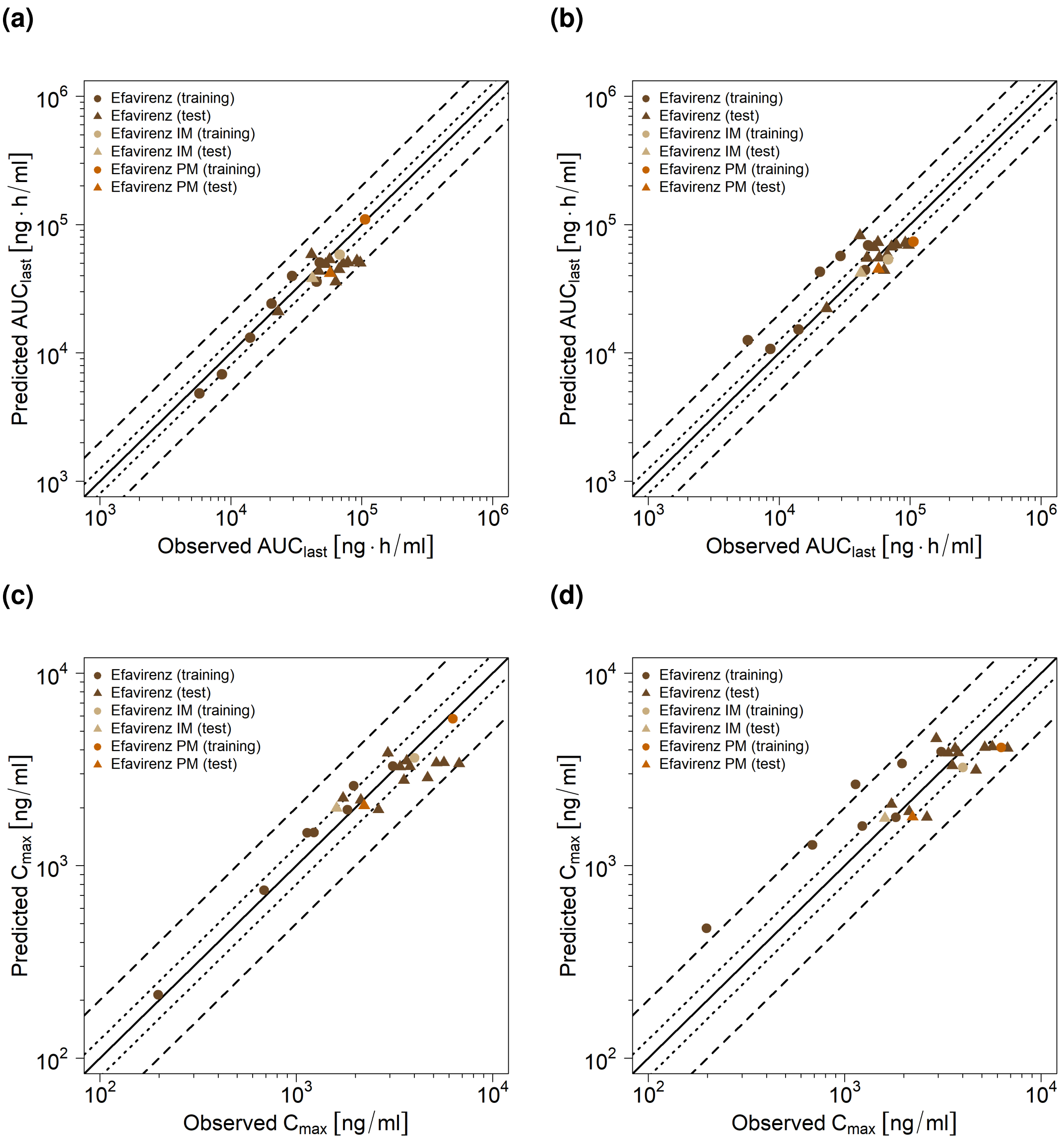

Figure S18: Predicted compared to observed (a,b) efavirenz AUC_{last} and (c,d) C_{max} values of the training (triangles) and test (circles) datasets. (a,c) show the updated efavirenz model, (b,d) show the original model. The solid line marks the line of identity. Dotted lines indicate 1.25-fold, dashed lines indicate 2-fold deviation. AUC_{last}: area under the plasma concentration-time curve from the time of drug administration to the last concentration measurement, C_{max}: maximum plasma concentration, IM: intermediate metabolizers (CYP2B6*1|*6), PM: poor metabolizers (CYP2B6*6|*6)

3.5.4 Geometric mean fold error of predicted AUC_{last} and C_{max} values

Table S8: Predicted and observed efavirenz AUC_{last} and C_{max} values with geometric mean fold errors of the updated model in comparison to the original model

		**AUC_{last} [ng*h/ml]**					**C_{max} [ng/ml]**					
			**updated**		**original**			**updated**		**original**		
**Route**	**Dose [mg]**	**Obs**	**Pred**	**Pred/Obs**	**Pred**	**Pred/Obs**	**Obs**	**Pred**	**Pred/Obs**	**Pred**	**Pred/Obs**	**Reference**
po, sd	50	5754.87	4838.42	0.84	12595.07	2.19	198.20	213.44	1.08	471.99	2.38	Derungs 2015 [89]
po, qd (D1)	200	8549.99	6814.78	0.80	10762.34	1.26	686.44	744.67	1.08	1279.23	1.86	Mouly 2002 [90]
po, qd (D10)	200	20499.16	24229.34	1.18	42920.81	2.09	1137.10	1478.74	1.30	2641.49	2.32	Mouly 2002 [90]
po, qd	400	58122.94	41716.36	0.72	55023.35	0.95	3534.24	2774.52	0.79	3308.00	0.94	Liu 2013 [91]
po, qd (D1)	400	14010.97	13152.89	0.94	15204.11	1.09	1232.47	148.96	1.21	1606.67	1.30	Mouly 2002 [90]
po, qd (D10)	400	29345.59	39868.54	1.36	57080.77	1.95	1960.69	2601.95	1.33	3388.03	1.73	Mouly 2002 [90]
po, sd	600	46884.25	43472.37	0.93	54901.73	1.17	1732.67	2245.91	1.30	2086.42	1.20	Cho 2016 [92]
po, qd	600	63363.54	35800.32	0.56	44097.59	0.70	2621.60	1952.53	0.74	1784.62	0.68	Ogburn 2010 [84]
po, sd	600	42014.64	38187.36	0.91	41888.82	1.00	1605.82	1991.59	1.24	1763.64	1.10	Xu 2013 (IM) [93]
po, sd	600	57284.66	41735.85	0.73	44946.79	0.78	2216.72	2048.71	0.92	1789.51	0.81	Xu 2013 (PM) [93]
po, sd	600	45168.30	35992.96	0.80	44340.57	0.98	1825.76	1952.54	1.07	1784.66	0.98	Xu 2013 (EM) [93]
po, qd	600	47653.02	50815.19	1.07	69063.36	1.45	3110.08	3291.76	1.06	3906.06	1.26	Dooley 2012 (EM) [94]
po, qd	600	67827.78	58427.85	0.86	53655.34	0.79	4013.47	3628.21	0.90	3237.77	0.81	Dooley 2012 (IM) [94]
po, qd	600	106157.40	109939.29	1.04	73853.54	0.70	6287.26	5800.13	0.92	4110.63	0.65	Dooley 2012 (PM) [94]
po, qd	600	97456.28	50278.98	0.52	69013.33	0.71	5651.38	3435.26	0.61	4146.26	0.73	Damle 2008 [95]
po, qd	600	56807.83	53486.71	0.94	72666.54	1.28	3650.55	3503.60	0.96	4064.81	1.11	Garg 2013 [96]
po, qd	600	72191.85	49600.72	0.69	67066.50	0.93	3790.09	3278.26	0.86	3863.82	1.02	Huang 2012 [97]
po, qd	600	78208.11	50642.86	0.65	69413.52	0.89	5176.12	3415.81	0.66	4121.84	0.80	Ji 2008 [44]
po, qd (D1)	600	22997.58	21008.78	0.91	22235.47	0.97	2130.00	2193.17	1.03	1912.06	0.90	Kharasch 2012 [98]
po, qd (D14)	600	41374.61	58497.10	1.41	82034.77	1.98	2926.26	3846.35	1.31	4574.00	1.56	Kharasch 2012 [98]
po, qd	600	66987.63	44762.00	0.67	58089.72	0.87	4665.93	2862.26	0.61	3130.82	0.67	Kwara 2011 [99]
po, qd	600	53003.19	49042.46	0.93	66377.21	1.25	3378.21	3262.64	0.97	3845.35	1.14	Malvestutto 2014 [100]
po, qd	600	92045.45	52013.69	0.57	71848.96	0.78	6751.46	3383.10	0.50	4077.99	0.60	Soon 2010 [101]
**mean GMFE (range)**				**1.31 (1.04-1.94)**		**1.36 (1.00-2.19)**			**1.26 (1.03-1.99)**		**1.41 (1.02-2.38)**	
				**23/23 with GMFE ≤ 2**		**21/23 with GMFE ≤ 2**			**23/23 with GMFE ≤ 2**		**21/23 with GMFE ≤ 2**	

AUC_{last}: area under the plasma concentration-time curve from the time of drug administration to the last concentration measurement, C_{max}: maximum plasma concentration, D: day, EM: extensive metabolizer, GMFE: geometric mean fold error, IM: intermediate metabolizer, obs: observed, po: oral, PM: poor metabolizer, pred: predicted, qd: once daily, sd: single dose

3.5.5 Sensitivity analysis

Sensitivity of the updated and original efavirenz PBPK models to single parameters (local sensitivity analysis) was calculated as the relative change of the predicted efavirenz AUC_{ss} at steady-state of an oral administration of 600 mg efavirenz once daily as tablet. Sensitivity analysis was carried out using a relative parameter perturbation of 1000% (variation range 10.0, maximum number of 9 steps). Parameters were included into the analysis if they were optimized (lipophilicity, solubility, CYP2B6, CYP1A2, CYP2A6, CYP3A4, CYP3A5 k_{cat} values, CYP3A4, CYP2B6 E_{max} values, intestinal permeability, Weibull tablet dissolution shape and time), if they are associated with optimized parameters (CYP2B6, CYP1A2, CYP2A6, CYP3A4, CYP3A5 K_m values, CYP3A4 and CYP2B6 EC_{50} values) or if they might have a strong impact due to calculation methods used in the model (fraction unbound in plasma, pKa, GFR fraction).

(a)

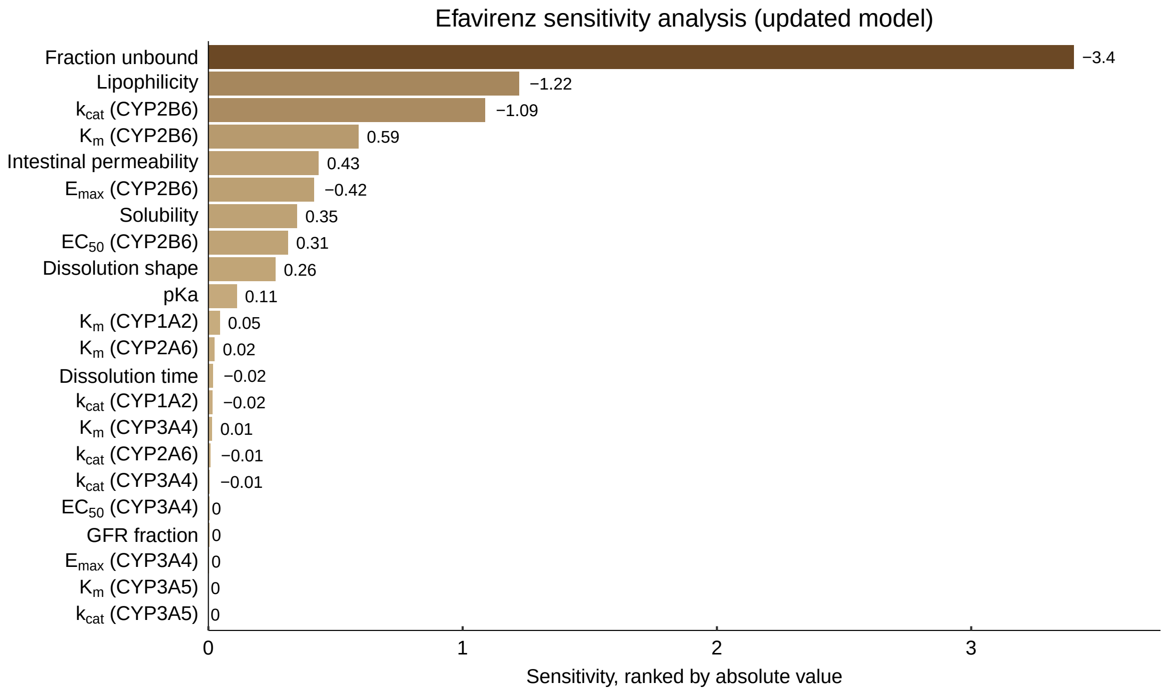

Figure S19: Efavirenz PBPK model sensitivity analysis. Sensitivity of the updated model to single parameters, calculated as change of the simulated efavirenz AUC_{ss} at steady-state of an oral administration of 600 mg efavirenz once daily as tablet. CYP: cytochrome P450, EC_{50}: half-maximal effective concentration, E_{max}: maximum effect, GFR: glomerular filtration rate, k_{cat}: catalytic rate constant, K_m: Michaelis-Menten constant

(a)

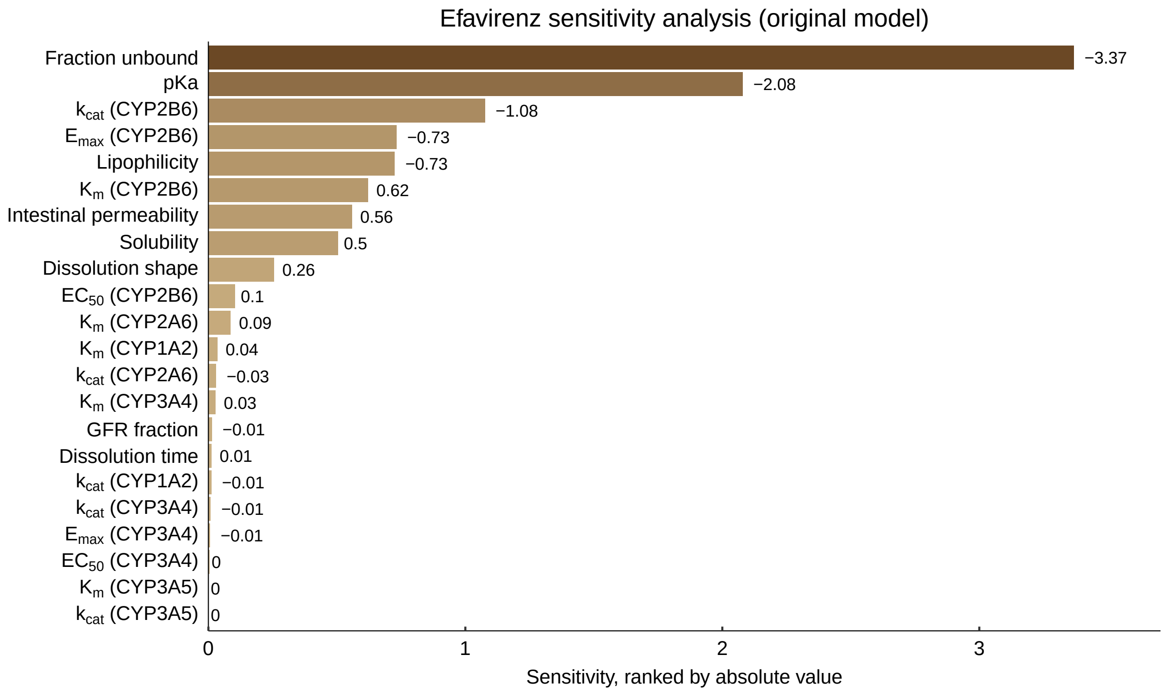

Figure S20: Efavirenz PBPK model sensitivity analysis. Sensitivity of the original model to single parameters, calculated as change of the simulated efavirenz AUC_{ss} at steady-state of an oral administration of 600 mg efavirenz once daily as tablet. CYP: cytochrome P450, EC_{50}: half-maximal effective concentration, E_{max}: maximum effect, GFR: glomerular filtration rate, k_{cat}: catalytic rate constant, K_m: Michaelis-Menten constant

4 Efavirenz drug-gene interactions (DGI)

4.1 DGI modeling - general

As CYP2B6 polymorphisms are a major determinant of efavirenz metabolism, the CYP2B6*6 polymorphism was integrated in the efavirenz PBPK model. Carriers of the CYP2B6*6 allele (either heterozygous or homozygous) show higher plasma concentrations of efavirenz than CYP2B6*1|*1 (wild type) carriers, due to decreased drug metabolism by CYP2B6 [86]. Parametrization of CYP2B6 metabolism in the efavirenz PBPK model describes efavirenz pharmacokinetics for the wild type. Intermediate metabolizers (CYP2B6*1|*6) and poor metabolizers (CYP2B6*6|*6) were described in the model by adjusting k_{cat}, assuming the same literature value vor K_m. Additionally, for CYP2B6 poor metabolizers no CYP2B6 auto-induction was assumed, as described in literature [86]. K_m and k_{cat} values used for DGI modeling are included in Table S6. Details on the modeled clinical studies investigating the efavirenz-CYP2B6*6 DGI are given in Table S9. Predicted efavirenz plasma concentration-time profiles for different CYP2B6 genotypes in comparison to their respective observed data are presented in Figures S21 (linear) and S22 (semi-logarithmic). The correlation of predicted to observed DGI AUC_{last} and C_{max} ratios is shown in Figure S23. Tables S10 and S11 list the corresponding predicted and observed DGI AUC_{last} ratios, DGI C_{max} ratios as well as model GMFE values of the updated model and the original model, respectively.

4.2 Efavirenz clinical DGI studies

Table S9: Clinical studies used for the establishment of the efavirenz DGI parameters

Dose [mg]	Route	Dataset	n	Healthy [%]	Females [%]	Age[a] [years]	Weight[a] [kg]	Height[a] [cm]	CYP2B6 genotype	Reference
600	po (-), sd	training	8	100	50	-	-	-	EM[b]	Xu 2013 [93]
600	po (-), sd	test	9	100	50	-	-	-	IM[b]	Xu 2013 [93]
600	po (-), sd	test	3	100	50	-	-	-	PM[b]	Xu 2013 [93]
600	po (-), qd	training	18	100	8	44[d] (19-62)	82.9[d] (57-119)	-	EM[c]	Dooley 2012 [94]
600	po (-), qd	training	12	100	8	44[d] (19-62)	82.9[d] (57-119)	-	IM[c]	Dooley 2012 [94]
600	po (-), qd	training	3	100	8	44[d] (19-62)	82.9[d] (57-119)	-	PM[c]	Dooley 2012 [94]

-: not given, CYP2B6: cytochrome P450 2B6, EM: extensive metabolizer, IM: intermediate metabolizer, PM: poor metabolizer, qd: once daily, sd: single dose

[a] mean (range)

[b] subjects were tested for the diminished-function allele CYP2B6*6

[c] subjects were tested for the diminished-function alleles CYP2B6*6 and CYP2B6*18

[d] median values

4.3 Profiles

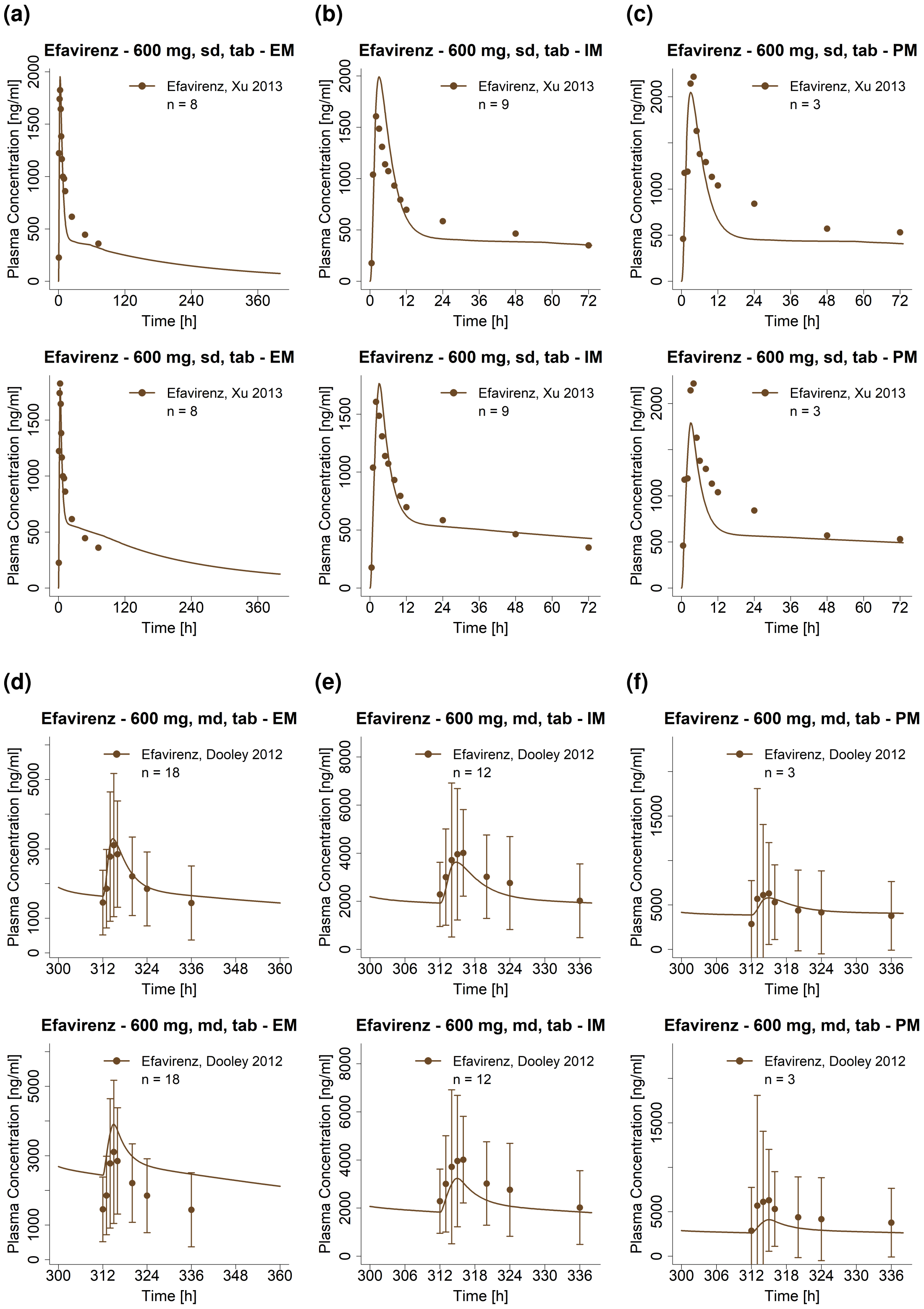

Figure S21: Predicted compared to observed efavirenz plasma concentration-time profiles (linear) of different CYP2B6 genotypes predicted with the updated model (upper rows) or the original model (lower rows). Observed data are shown as dots ± standard deviation; model predictions are shown as solid lines. Details on dosing regimens, study populations and literature references are listed in Table S5. md: multiple-dose, tab: tablet, sd: single dose

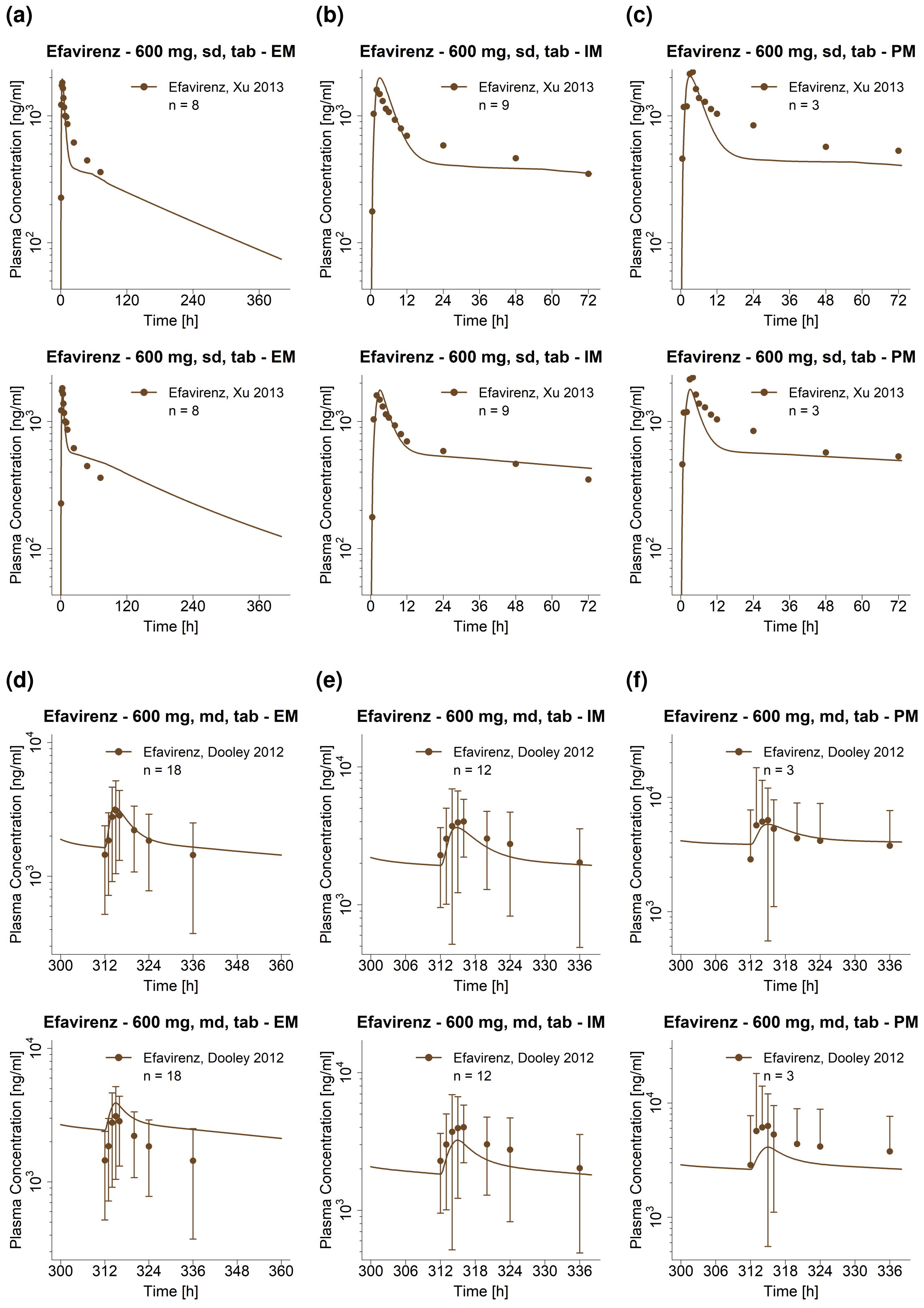

Figure S22: Predicted compared to observed efavirenz plasma concentration-time profiles (semi-logarithmic) of different CYP2B6 genotypes predicted with the updated model (upper rows) or the original model (lower rows). Observed data are shown as dots ± standard deviation; model predictions are shown as solid lines. Details on dosing regimens, study populations and literature references are listed in Table S5. md: multiple-dose, tab: tablet, sd: single dose

4.4 DGI AUC_{last} and C_{max} ratio goodness-of-fit plots

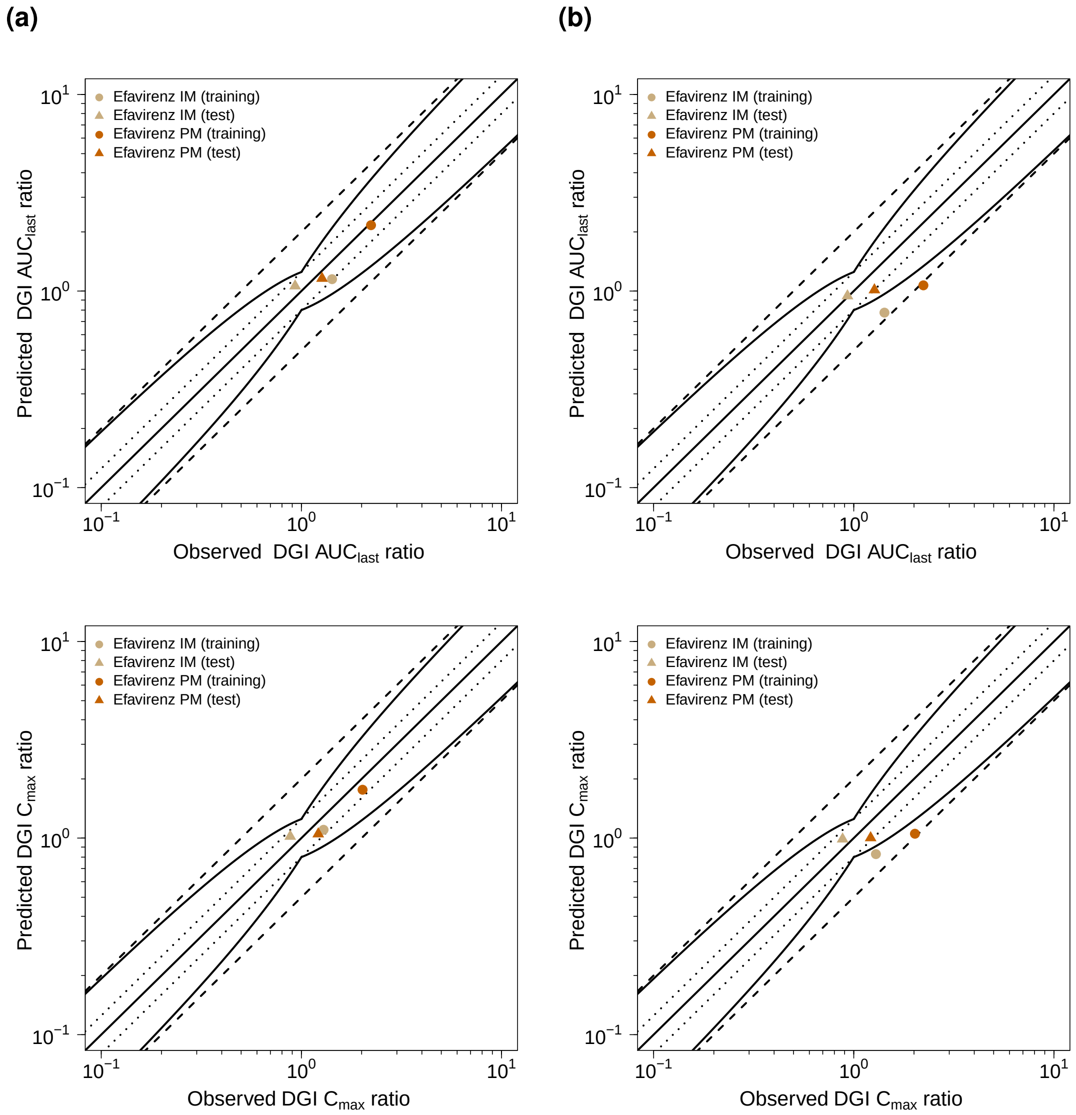

Figure S23: Predicted compared to observed efavirenz (a,b) DGI AUC_{last} and (c,d) DGI C_{max} ratios predicted with the updated efavirenz PBPK model (left) or the original PBPK model (right). The straight solid line marks the line of identity, the curved solid lines show the prediction success limits proposed by Guest et al. allowing for 1.25-fold variability of the DGI ratio [109]. Dotted lines indicate 1.25-fold, dashed lines indicate 2-fold deviation. AUC_{last}: area under the plasma concentration-time curve from the time of drug administration to the last concentration measurement, C_{max} : maximum plasma concentration, DGI: drug-gene interaction, IM: intermediate metabolizers (CYP2B6*1|*6), PM: poor metabolizers (CYP2B6*6|*6)

4.5 Geometric mean fold error of predicted DGI AUC_{last} and C_{max} ratios

Table S10: Predicted and observed DGI AUC_{last} and C_{max} ratios with geometric mean fold errors of the updated model

		DGI AUC_{last} ratio			DGI C_{max} ratio			
**Administration**	**Genotype**	**Pred**	**Obs**	**Pred/Obs**	**Pred**	**Obs**	**Pred/Obs**	**Reference**
600 mg, po, sd	CYP2B6*1|*6	1.06	0.93	1.14	1.02	0.88	1.16	Xu 2013 [93]
600 mg, po, sd	CYP2B6*6|*6	1.16	1.27	0.91	1.05	1.21	0.86	Xu 2013 [93]
600 mg, po, qd	CYP2B6*1|*6	1.14	1.42	0.81	1.10	1.29	0.85	Dooley 2012 [94]
600 mg, po, qd	CYP2B6*6|*6	2.16	2.23	0.97	1.76	2.02	0.87	Dooley 2012 [94]
**mean GMFE (range)**				**1.12 (1.03-1.24)**			**1.16 (1.14-1.17)**	
				**4/4 with GMFE ≤ 2**			**4/4 with GMFE ≤ 2**	

AUC_{last}: area under the plasma concentration-time curve from the time of drug administration to the last concentration measurement, C_{max}: maximum plasma concentration, CYP2B6: cytochrome P450 2B6, DGI: drug-gene interaction, GMFE: geometric mean fold error, obs: observed, po: oral, pred: predicted, qd: once daily, sd: single dose

Table S11: Predicted and observed DGI AUC_{last} and C_{max} ratios with geometric mean fold errors of the original model

		DGI AUC_{last} ratio			DGI C_{max} ratio			
**Administration**	**Genotype**	**Pred**	**Obs**	**Pred/Obs**	**Pred**	**Obs**	**Pred/Obs**	**Reference**
600 mg, po, sd	CYP2B6*1|*6	0.94	0.93	1.02	0.99	0.88	1.12	Xu 2013 [93]
600 mg, po, sd	CYP2B6*6|*6	1.01	1.27	0.80	1.00	1.21	0.83	Xu 2013 [93]
600 mg, po, qd	CYP2B6*1|*6	0.78	1.42	0.55	0.83	1.29	0.64	Dooley 2012 [94]
600 mg, po, qd	CYP2B6*6|*6	1.07	2.23	0.48	1.05	2.02	0.52	Dooley 2012 [94]
**mean GMFE (range)**				**1.54 (1.01-2.08)**			**1.45 (1.12-1.92)**	
				**3/4 with GMFE ≤ 2**			**4/4 with GMFE ≤ 2**	

AUC_{last}: area under the plasma concentration-time curve from the time of drug administration to the last concentration measurement, C_{max}: maximum plasma concentration, CYP2B6: cytochrome P450 2B6, DGI: drug-gene interaction, GMFE: geometric mean fold error, obs: observed, po: oral, pred: predicted, qd: once daily, sd: single dose

5 Carbamazepine drug-drug interactions (DDI)

5.1 DDI modeling - general

The accurate prediction of DDIs indicates that the perpetrator model adequately describes the drug concentrations at the site(s) of interaction and that the victim drug model sufficiently describes the amount of drug eliminated via the affected pathway. Therefore, DDI predictions are considered as additional evaluation of both models.

A total number of 7 DDI studies, providing 8 victim drug plasma concentration-time profiles and 7 metabolite plasma concentration-time profiles, was utilized to evaluate the DDI performance of the carbamazepine parent-metabolite PBPK model, including studies with a CYP3A4 inhibitor (erythromycin), CYP3A4 victim drugs (alprazolam and simvastatin), a CYP2B6 victim drug (bupropion) as well as a CYP3A4 and CYP2B6 victim and perpetrator drug (efavirenz). The carbamazepine DDI network is illustrated in Figure S24.

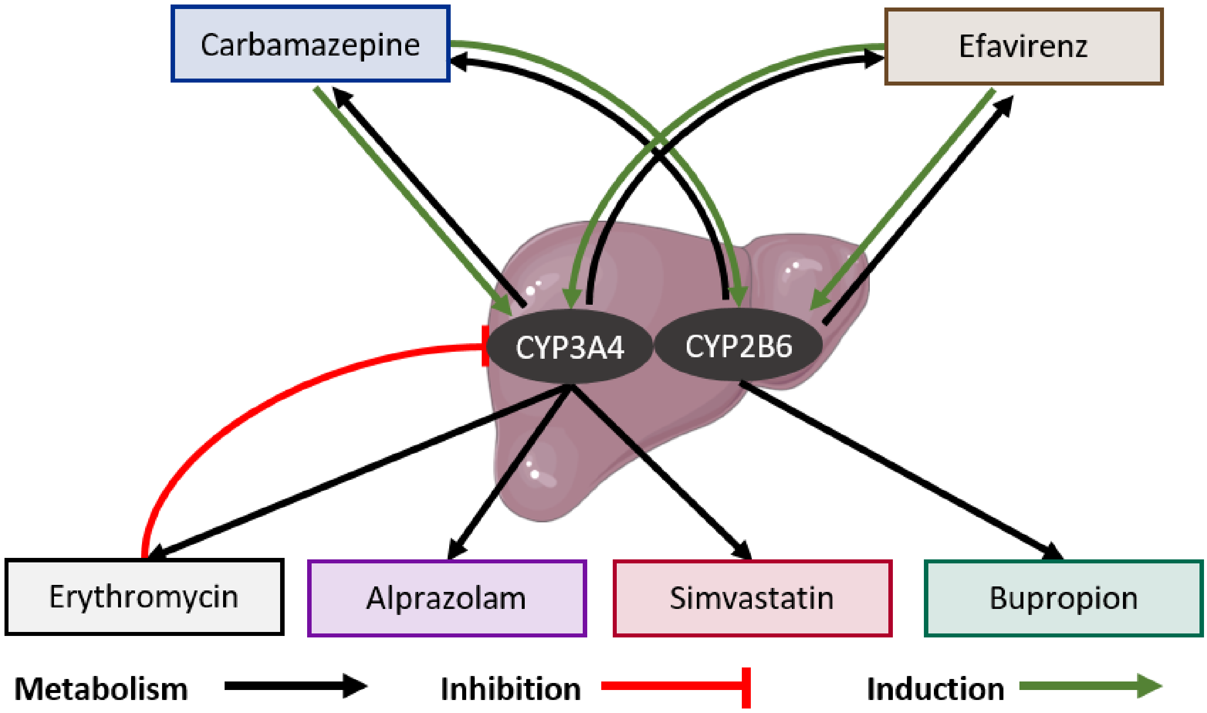

Figure S24: Carbamazepine drug-drug interaction network. Black arrows represent metabolism of the compounds, red and green arrows represent inhibition or induction of the CYP enzymes, respectively

The parameters describing the induction of CYP3A4 and CYP2B6 by carbamazepine were already introduced during carbamazepine model building, as the compound induces its own metabolism. While the carbamazepine-alprazolam DDI study was used in the training dataset to inform the parametrization of the carbamazepine CYP3A4 induction, all other DDIs were purely predicted. The implementation of these DDIs is described in more detail in the following sections.

5.2 Erythromycin-carbamazepine DDI

The erythromycin-carbamazepine DDI was modeled using a previously developed whole-body PBPK model of erythromycin, available in the OSP GitHub model repository (https://github.com/Open-Systems-Pharmacology/Erythromycin-Model). Erythromycin is a substrate and mechanism-based inhibitor of CYP3A4. The drug-dependent parameters of the erythromycin model are reproduced in Table S12.

The erythromycin-carbamazepine DDI was modeled as mechanism-based inhibition of carbamazepine CYP3A4 metabolism by erythromycin. K_i and K_{inact} values, describing the mechanism-based CYP3A4 inhibition by erythromycin, were qualified previously in different DDI preditions [110, 111]. Induction of erythromycin CYP3A4 metabolism by carbamazepine was also implemented, as significant CYP3A4 induction can be assumed after multiple-dose administration of carbamazepine, although the effect of carbamazepine on erythromycin metabolism has not been investigated in clinical studies, yet. CYP3A4 induction by carbamazepine was described using EC_{50} = 20.0 µmol/l from literature, the carbamazepine-alprazolam DDI study was used in the training dataset to inform the parametrization of the E_{max} = 6.0.

Details on the modeled clinical DDI studies are given in Table S13. Model predictions of carbamazepine and carbamazepine-10,11-epoxide plasma concentration-time profiles before and during erythromycin co-administration, compared to observed data, are shown in Figures S25 (linear) and S26 (semi-logarithmic). The correlation of predicted to observed DDI AUC_{last} and C_{max} ratios is shown in Figure S27. Table S14 lists the corresponding predicted and observed DDI AUC_{last} ratios, DDI C_{max} ratios, as well as GMFE values.

5.2.1 Erythromycin drug-dependent parameters

Table S12: Drug-dependent parameters of the erythromycin PBPK model (adopted from [110])

Parameter	Unit	Model	Literature	Reference	Description
MW	g/mol	733.9 (Lit)	733.9	[112]	Molecular weight
logP	Log Units	2.82 (Lit)	2.82 (2.48-3.06)[a]	[113–115]	Lipophilicity
Solubility (pH)	mg/ml	200.0 (7.0) (lactobionate) (Lit),	200.0 (7.0) (lactobionate),	[116]	Solubility
		0.028 (7.0) (stearate) (Fit),	0.182 (7.0) (stearate),	[117]	
		0.50 (7.0) (base pellets) (Fit),	2.10 (7.0) (base)	[118]	
		0.0084 (7.0) (base tablet) (Fit)			
fu	%	30.5 (Lit)	27.0, 28.0, 30.5, 32.6	[119–122]	Fraction unbound in plasma
pKa (base)	-	8.88 (Lit)	8.88	[115]	Acid dissociation constant
K_m (CYP3A4)	µmol/l	70.0 (Lit)	70 (44.0-88.0)[a]	[123, 124]	CYP3A4 Michaelis-Menten constant
k_{cat} (CYP3A4)	1/min	8.50 (Fit)	-	-	CYP3A4 catalytic rate constant
K_m (OATP1B1)	µmol/l	0.74 (Fit)	13.2	[125]	OATP1B1 Michaelis-Menten constant
k_{cat} (OATP1B1)	1/min	2.02 (Fit)	-	-	OATP1B1 transport rate constant
CL_{hep}	1/min	4.15 (Fit)	-	-	Hepatic plasma clearance
GFR fraction	-	1.16 (Fit)	-	-	Fraction of filtered drug in the urine
K_i (CYP3A4)	µmol/l	7.60 (Fit)	18.4 (0.76-109.0)[b]	[122, 126–136]	Concentration for half-maximal inactivation
k_{inact} (CYP3A4)	1/min	0.03 (Fit)	0.06 (0.01-0.30)[b]	[122, 126–136]	Maximum inactivation rate constant
Intestinal permeability	cm/min	3.87E-04 (Fit)	-	-	Transcellular intestinal permeability
Partition coefficients	-	Diverse	Rogers and Rowland	[77, 78]	Cell to plasma partition coefficients
Cellular permeability	cm/min	1.22E-4 (Calc)	Charge-dependent Schmitt	[2]	Permeability into the cellular space
Coated pellets Weibull time	min	1.75 (Fit)	-	-	Dissolution time (50% dissolved)
Coated pellets Weibull lag time	min	54.35 (Fit)	-	-	Dissolution lag time
Coated pellets Weibull shape	-	1.06 (Fit)	-	-	Dissolution profile shape
Coated tablet Weibull time	min	79.63 (Fit)	-	-	Dissolution time (50% dissolved)
Coated tablet Weibull lag time	min	78.79 (Fit)	-	-	Dissolution lag time
Coated tablet Weibull shape	-	1.08 (Fit)	-	-	Dissolution profile shape
Film tablet Weibull time	min	1.70 (Fit)	-	-	Dissolution time (50% dissolved)
Film tablet Weibull shape	-	1.10 (Fit)	-	-	Dissolution profile shape

-: not given, calc: calculated, CYP3A4: cytochrome P450 3A4, fit: optimized during parameter identification, GFR: glomerular filtration rate, lit: literature, OATP1B1: organic anion transporting polypeptide 1B1

[a] mean (range)

5.2.2 Erythromycin-carbamazepine clinical DDI studies

Table S13: Clinical studies investigating the erythromycin-carbamazepine DDI

Erythromycin administration		Carbamazepine administration		n	Healthy [%]	Females [%]	Age[a] [years]	Weight[a] [kg]	Height [cm]	Reference
Dose [mg]	Route	Dose [mg]	Route							
250	po (cap), qid (D15-D17)	357[b]	po (tab), qd (D1-D17)	7	100	0	25.4 (22-27)	78.8 (70.8-90.9)	-	Miles 1989 [45]
500	po (-), tid (D1-D10)	400	po (tab*), sd (D7)	7	-	-	(21-27)	(70-90)	-	Barzaghi 1987 [32]
250	po (-), qid (D1-D8)	400	po (tab), sd (D6)	8	100	0	(24-36)	(72.3-96.4)	-	Wong 1983 [38]

-: not given, cap: capsule, D: day, po: oral, qd: once daily, qid: four times daily, sd: single dose, tab: tablet, tab*: tablet with concomitant food intake, tid: three times daily

[a] mean (range)

[b] mean administered dose (range: 300-400 mg)

5.2.3 Profiles

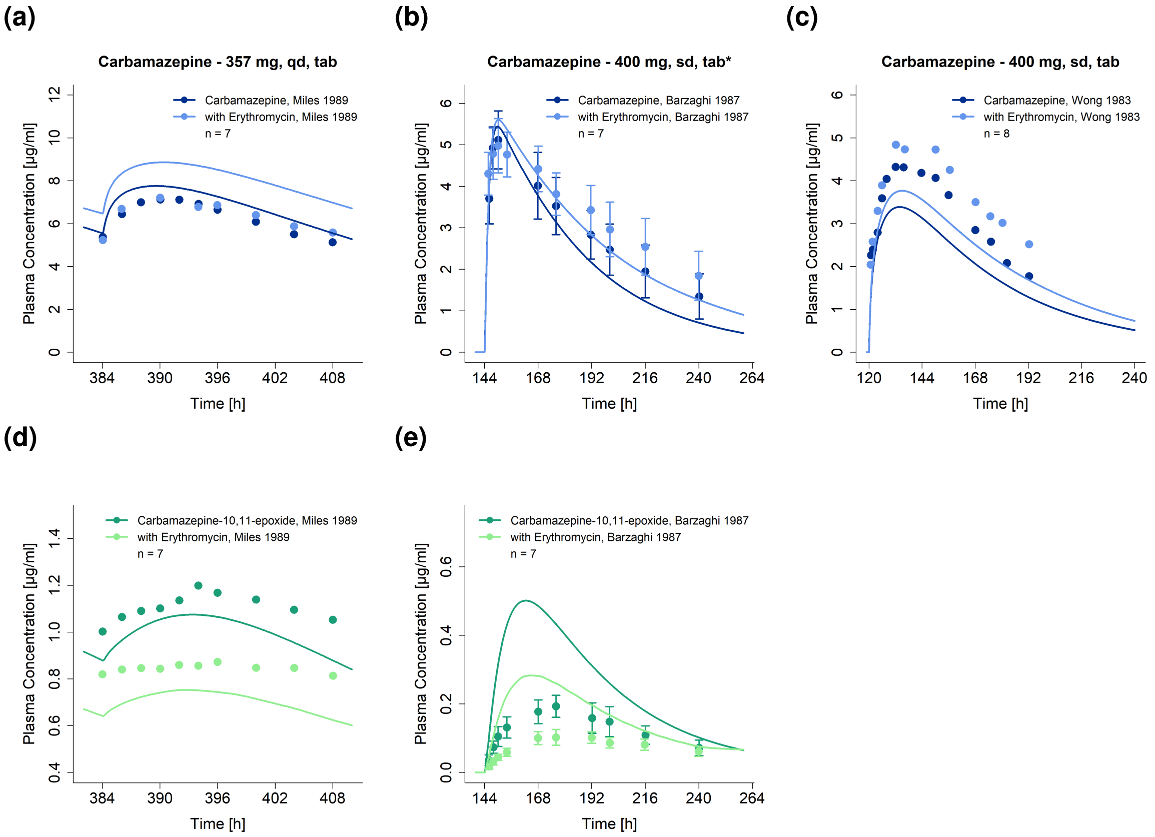

Figure S25: Predicted compared to observed carbamazepine (upper row) and carbamazepine-10,11-epoxide (lower row) plasma concentration-time profiles (linear) before and during erythromycin co-administration. Observed data are shown as dots ± standard deviation; model predictions are shown as solid lines. Details on dosing regimens, study populations and literature references are listed in Table S13. qd: once daily, sd: single dose, tab: tablet, tab*: tablet with concomitant food intake

(a)

(b)

(c)

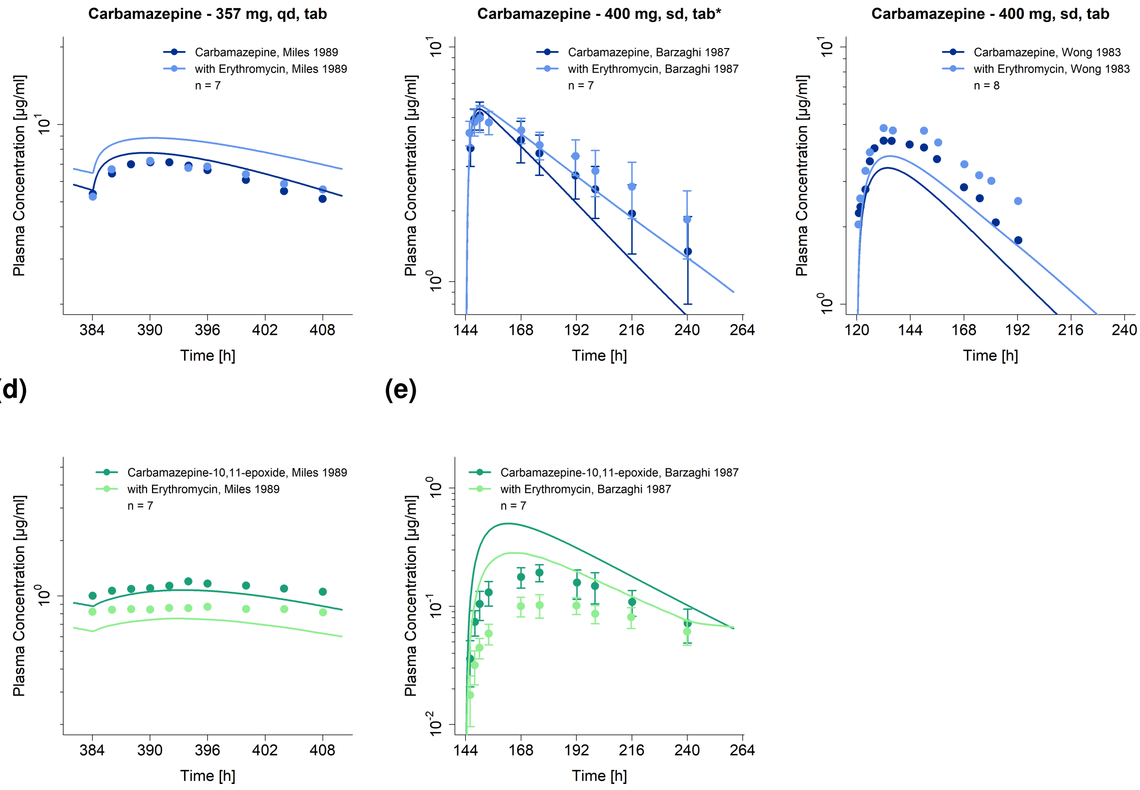

(d)

(e)

Figure S26: Predicted compared to observed carbamazepine (upper row) and carbamazepine-10,11-epoxide (lower row) plasma concentration-time profiles (semi-logarithmic) before and during erythromycin co-administration. Observed data are shown as dots ± standard deviation; model predictions are shown as solid lines. Details on dosing regimens, study populations and literature references are listed in Table S13. qd: once daily, sd: single dose, tab: tablet, tab*: tablet with concomitant food intake

5.2.4 DDI AUC_{last} and C_{max} ratio goodness-of-fit plots

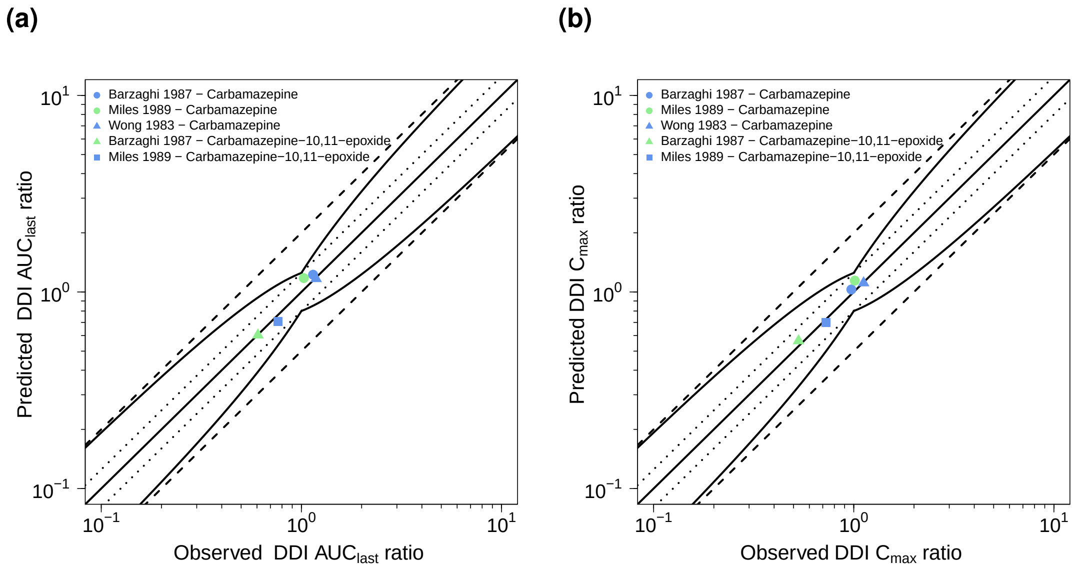

Figure S27: Predicted compared to observed erythromycin-carbamazepine (a) DDI AUC_{last} and (b) DDI C_{max} ratios. The straight solid line marks the line of identity, the curved solid lines show the prediction success limits proposed by Guest et al. allowing for 1.25-fold variability of the DDI ratio [109]. Dotted lines indicate 1.25-fold, dashed lines indicate 2-fold deviation. AUC_{last}: area under the plasma concentration-time curve from the time of drug administration to the last concentration measurement, C_{max}: maximum plasma concentration, DDI: drug-drug interaction

5.2.5 Geometric mean fold error of predicted DDI AUC_{last} and C_{max} ratios

Table S14: Predicted and observed erythromycin-carbamazepine DDI AUC_{last} and C_{max} ratios with geometric mean fold errors

**Perpetrator**	**Victim**				**DDI AUC_{last} ratio**			**DDI C_{max} ratio**			
Erythromycin	Carbamazepine	**Compound**	**Dose gap [h]**	**n**	**Pred**	**Obs**	**Pred/Obs**	**Pred**	**Obs**	**Pred/Obs**	**Reference**
250 mg, po, qid	357 mg[a], po, qd	Carbamazepine	-	7	1.18	1.03	1.14	1.14	1.01	1.13	Miles 1989 [45]
250 mg, po, qid	357 mg[a], po, qd	Carbamazepine-10,11-epoxide	-	7	0.71	0.76	0.93	0.70	0.73	0.96	Miles 1989 [45]
500 mg, po, tid	400 mg, po, sd	Carbamazepine	-	7	1.22	1.14	1.05	1.03	0.97	1.06	Barzaghi 1987 [32]
500 mg, po, tid	400 mg, po, sd	Carbamazepine-10,11-epoxide	-	7	0.60	0.61	1.00	0.56	0.53	1.06	Barzaghi 1987 [32]
250 mg, po, qid	400 mg, po, sd	Carbamazepine	-	8	1.17	1.18	0.99	1.11	1.12	0.99	Wong 1983 [38]
**mean GMFE (range) (Carbamazepine)**							**1.08 (1.01-1.14)**			**1.06 (1.01-1.13)**	
							**3/3 with GMFE ≤ 2**			**3/3 with GMFE ≤ 2**	
**mean GMFE (range) (Carbamazepine-10,11-epoxide)**							**1.04 (1.00-1.08)**			**1.05 (1.04-1.06)**	
							**2/2 with GMFE ≤ 2**			**2/2 with GMFE ≤ 2**	

-: not given, AUC_{last}: area under the plasma concentration-time curve from the time of drug administration to the last concentration measurement, C_{max}: maximum plasma concentration, DDI: drug-drug interaction, GMFE: geometric mean fold error, obs: observed, po: oral, pred: predicted, qd: once daily, qid: four times daily, sd: single dose, tid: three times daily

[a] Mean administered carbamazepine dose

5.3 Carbamazepine-alprazolam DDI

The carbamazepine-alprazolam DDI was modeled using a previously developed whole-body PBPK model of alprazolam, available in the OSP GitHub model repository (https://github.com/Open-Systems-Pharmacology/Alprazolam-Model). The metabolism of the CYP3A4 substrate alprazolam is described using Michaelis-Menten kinetics [137]. The drug-dependent parameters of the alprazolam model are reproduced in Table S15.

The carbamazepine-alprazolam DDI was modeled as induction of alprazolam CYP3A4 metabolism by carbamazepine. CYP3A4 induction by carbamazepine was described using EC_{50} = 20.0 µmol/l from literature, the carbamazepine-alprazolam DDI study was used in the training dataset to inform the parametrization of the E_{max} = 6.0.

Details on the modeled clinical DDI study are given in Table S16. Model predictions of alprazolam plasma concentration-time profiles before and during carbamazepine co-administration, compared to observed data, are shown in Figures S28 (linear) and S29 (semi-logarithmic). The correlation of predicted to observed DDI AUC_{last} and C_{max} ratios is shown in Figure S30. Table S17 lists the corresponding predicted and observed DDI AUC_{last} ratios, DDI C_{max} ratios, as well as GMFE values.

5.3.1 Alprazolam drug-dependent parameters

Table S15: Drug-dependent parameters of the alprazolam PBPK model (adopted from [137])

Parameter	Unit	Model	Literature	Reference	Description
MW	g/mol	308.77 (Lit)	308.77	[138]	Molecular weight
logP	Log Units	2.05 (Fit)	1.26 (logD)	[139]	Lipophilicity
			2.19 (logP)	[140]	
Solubility (pH)	mg/ml	0.04 (7.0) (Lit)	0.012 (1.2),	[138]	Solubility
			0.04 (7.0),	[138]	
			0.08 (7.0)	[141]	
fu	%	23.3 (Lit)	20.0, 23.3, 31.1	[142–144]	Fraction unbound in plasma
pKa (base)	-	2.40 (Lit)	2.40, 2.48	[145–147]	Acid dissociation constant
K_m (CYP3A4) α-hydroxy	µmol/l	269.0 (Lit)	269.0	[148]	CYP3A4 Michaelis-Menten constant for α-hydroxy-alprazolam formation
k_{cat} (CYP3A4) α-hydroxy	1/min	0.73 (Fit)	-	-	CYP3A4 catalytic rate constant for α-hydroxy-alprazolam formation
K_m (CYP3A4) 4-hydroxy	µmol/l	704.0 (Lit)	704.0	[148]	CYP3A4 Michaelis-Menten constant for 4-hydroxy-alprazolam formation
k_{cat} (CYP3A4) 4-hydroxy	1/min	12.44 (Fit)	-	-	CYP3A4 catalytic rate constant for 4-hydroxy-alprazolam formation
GFR fraction	-	0.52 (Fit)	-	-	Fraction of filtered drug in the urine
Intestinal permeability	cm/min	0.65 (Fit)	-	-	Transcellular intestinal permeability
Partition coefficients	-	Diverse	Rogers and Rowland	[77, 78]	Cell to plasma partition coefficients
Cellular permeability	cm/min	5.74E-3 (Calc)	PK-Sim Standard	[79]	Permeability into the cellular space
Tablet (Solanax®) Weibull time	min	35.72 (Fit)	-	-	Dissolution time (50% dissolved)
Tablet (Solanax®) Weibull shape	-	0.72 (Fit)	-	-	Dissolution profile shape

-: not given, calc: calculated, CYP: cytochrome P450, fit: optimized during parameter identification, GFR: glomerular filtration rate, lit: literature

5.3.2 Carbamazepine-alprazolam clinical DDI studies

Table S16: Clinical studies investigating the carbamazepine-alprazolam DDI

Carbamazepine administration		Alprazolam administration		n	Healthy [%]	Females [%]	Age[a] [years]	Weight[a] [kg]	Height [cm]	Reference
**Dose [mg]**	**Route**	**Dose [mg]**	**Route**							
100	po (tab), tid (D1-D10)	0.8	po (tab), sd (D8)	7	100	0	32.7	60.9	-	Furukori 1998 [149]

-: not given, D: day, po: oral, sd: single dose, tab: tablet, tid: three times daily

[a] Mean values

5.3.3 Profiles

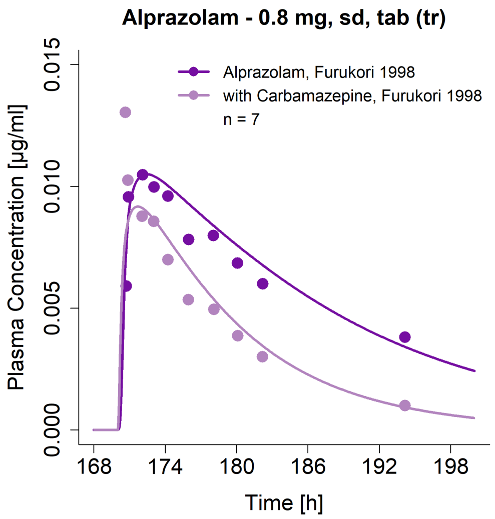

Figure S28: Predicted compared to observed alprazolam plasma concentration-time profiles (linear) before and during carbamazepine co-administration. Observed data are shown as dots; model predictions are shown as solid lines. Details on dosing regimens, study population and literature reference are listed in Table S16. sd: single dose, tab: tablet

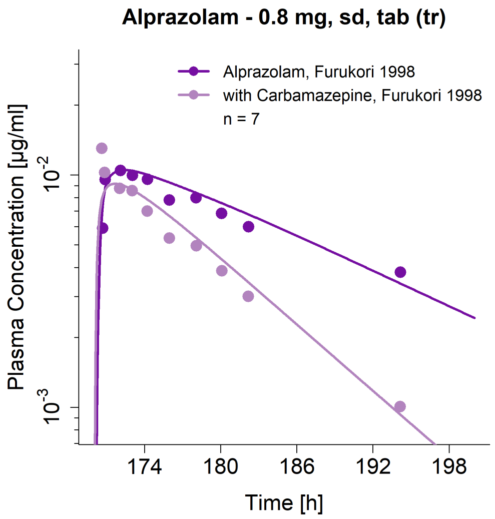

Figure S29: Predicted compared to observed alprazolam plasma concentration-time profiles (semi-logarithmic) before and during carbamazepine co-administration. Observed data are shown as dots; model predictions are shown as solid lines. Details on dosing regimens, study population and literature reference are listed in Table S16. sd: single dose, tab: tablet

5.3.4 DDI AUC_{last} and C_{max} ratio goodness-of-fit plots

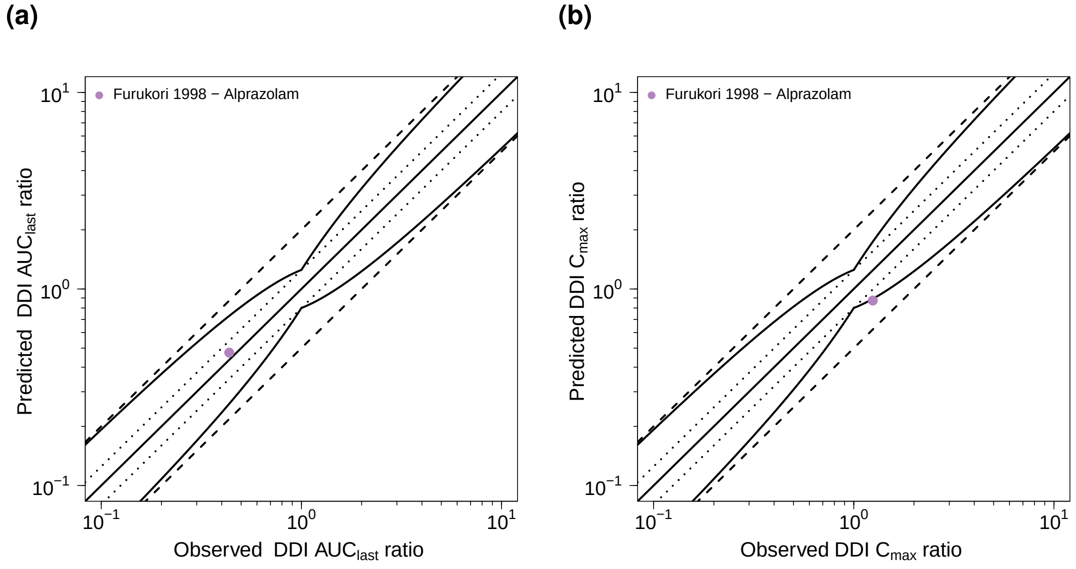

Figure S30: Predicted compared to observed carbamazepine-alprazolam (a) DDI AUC_{last} and (b) DDI C_{max} ratios. The straight solid line marks the line of identity, the curved solid lines show the prediction success limits proposed by Guest et al. allowing for 1.25-fold variability of the DDI ratio [109]. Dotted lines indicate 1.25-fold, dashed lines indicate 2-fold deviation. AUC_{last}: area under the plasma concentration-time curve from the time of drug administration to the last concentration measurement, C_{max}: maximum plasma concentration, DDI: drug-drug interaction

5.3.5 Geometric mean fold error of predicted DDI AUC_{last} and C_{max} ratios

Table S17: Predicted and observed carbamazepine-alprazolam DDI AUC_{last} and C_{max} ratios with geometric mean fold errors

**Perpetrator**	**Victim**			**DDI AUC_{last} ratio**			**DDI C_{max} ratio**			
Carbamazepine	Alprazolam	**Dose gap [h]**	**n**	**Pred**	**Obs**	**Pred/Obs**	**Pred**	**Obs**	**Pred/Obs**	**Reference**
100 mg, po, tid	0.8 mg, po, sd	2	7	0.47	0.44	1.09	0.87	1.24	0.70	Furukori 1998 [149]
**mean GMFE**						**1.09**			**1.43**	
						**1/1 with GMFE ≤ 2**			**1/1 with GMFE ≤ 2**	

AUC_{last}: area under the plasma concentration-time curve from the time of drug administration to the last concentration measurement, C_{max}: maximum plasma concentration, DDI: drug-drug interaction, GMFE: geometric mean fold error, obs: observed, po: oral, pred: predicted, sd: single dose, tid: three times daily

5.4 Carbamazepine-simvastatin DDI

The carbamazepine-simvastatin DDI was modeled using a previously established whole-body parent-metabolite PBPK model of simvastatin [150]. Simvastatin is metabolized by CYP3A4, with simvastatin acid as the main metabolite. Simvastatin acid is also a substrate of CYP3A4. Metabolism of simvastatin and simvastatin acid by CYP3A4 is described in the model using Michaelis-Menten kinetics. Both compounds are also competitive inhibitors of CYP3A4 as well as CYP2C8. The drug-dependent parameters of the simvastatin model are reproduced in Table S18.

The carbamazepine-simvastatin DDI was modeled as induction of simvastatin and simvastatin acid CYP3A4 metabolism by carbamazepine. CYP3A4 induction by carbamazepine was described using EC_{50} = 20.0 µmol/l from literature, E_{max} = 6.0 was identified during the carbamazepine parameter identification. Competitive inhibition of carbamazepine CYP3A4 and CYP2C8 metabolism by simvastatin and simvastatin acid was also implemented, although the effect of simvastatin and simvastatin acid on carbamazepine metabolism has not been investigated in clinical studies, yet. K_i values, describing the competitive CYP3A4 and CYP2C8 inhibition by simvastatin and simvastatin acid, were qualified previously in different DDI preditions [150].

Details on the modeled clinical DDI study are given in Table S19. Model predictions of simvastatin and simvastatin acid plasma concentration-time profiles before and during carbamazepine co-administration, compared to observed data, are shown in Figures S31 (linear) and S32 (semi-logarithmic). The correlation of predicted to observed DDI AUC_{last} and C_{max} ratios is shown in Figure S33. Table S20 lists the corresponding predicted and observed DDI AUC_{last} ratios, DDI C_{max} ratios, as well as GMFE values.

5.4.1 Simvastatin drug-dependent parameters

Table S18: Drug-dependent parameters of the simvastatin PBPK model (adopted from [150])

Parameter	Unit	Model	Literature[a]	Reference	Description
**Simvastatin**					
MW	g/mol	418.57 (Lit)	418.57	[151]	Molecular weight
logP	Log Units	4.68 (Lit)	4.60 (2.06-5.19)	[152, 153]	Lipophilicity
Solubility (pH)	mg/ml	0.016 (5.0) (Lit)	0.016 (1.40E-3-0.061) (5.0)	[151, 152, 154–157]	Solubility
fu	%	1.34 (Lit)	2.37 (1.09-6.00)	[152, 158, 159]	Fraction unbound in plasma
K_m (CYP3A4)	µmol/l	21.0 (Lit)	2.55 (0.46-30.0)	[160–162]	CYP3A4 Michaelis-Menten constant
k_{cat} (CYP3A4)	1/min	5194.04 (Fit)	-	-	CYP3A4 catalytic rate constant
K_m (CYP3A5)	µmol/l	39.1 (Fit)	88.0 (62.0-91.0)	[160]	CYP3A5 Michaelis-Menten constant
k_{cat} (CYP3A5)	1/min	162281.57 (Fit)	-	-	CYP3A5 catalytic rate constant
K_m (PON3)	µmol/l	840.0 (Lit)	840.0	[163]	PON3 Michaelis-Menten constant
k_{cat} (PON3)	1/min	4952.08 (Fit)	-	-	PON3 catalytic rate constant
Chemical hydrolysis rate	l/µmol/min	9.80E-4 (Lit)	8.21E-4 (1.67E-06-1.96E-2)	[151, 164, 165]	Chemical hydrolysis rate (ubiquitous)
Plasma hydrolysis rate	l/µmol/min	6.03E-2 (Lit)	6.03E-2	[166]	Plasma hydrolysis rate
K_m (BCRP)	µmol/l	5.00[b] (Asm)	(1.20-10.8)	[167–172]	BCRP Michaelis-Menten constant
k_{cat} (BCRP)	1/min	32.61 (Fit)	-	-	BCRP catalytic rate constant
GFR fraction	-	1 (Asm)	-	-	Fraction of filtered drug in the urine
K_i (CYP2C8)	µmol/l	1.10 (Lit)	5.7 (1.10-12.3)	[173]	Concentration for half-maximal inhibition
K_i (CYP3A4)	µmol/l	0.16 (Lit)	2.1 (0.16-35.0)	[160, 174–177]	Concentration for half-maximal inhibition
K_i (OATP1B1)	µmol/l	5.00 (Lit)	7.85 (5.00-12.5)	[178, 179]	Concentration for half-maximal inhibition
K_i (Pgp)	µmol/l	4.60 (Lit)	37.7 (4.60-209.0)	[180, 180–186]	Concentration for half-maximal inhibition
Intestinal permeability	cm/min	1.08E-3 (Fit)	0.258	[151]	Transcellular intestinal permeability
Partition coefficients	-	Diverse	Berezhkovskiy	[187]	Cell to plasma partition coefficients
Cellular permeability	cm/min	0.26 (Calc)	PK-Sim Standard	[79]	Permeability into the cellular space
Tablet Weibull time	min	86.38 (Fit)	-	-	Dissolution time (50% dissolved)
Tablet Weibull shape	-	1.30 (Fit)	-	-	Dissolution profile shape

-: not given, asm: assumption, BCRP: breast cancer resistance protein, calc: calculated, CYP: cytochrome P450, fit: optimized during parameter identification, GFR: glomerular filtration rate, lit: literature, OATP: organic anion transporting polypeptide, Pgp: P-glycoprotein, PON3: paraoxonase 3, UGT: UDP-glucuronosyltransferase

[a] median (range)

[b] assumed from other statins

[c] calculated from V_{max}

[d] calculated from liver S9

Table S18: Drug-dependent parameters of the simvastatin PBPK model (adopted from [150]) *(continued)*

Parameter	Unit	Model	Literature[a]	Reference	Description
**Simvastatin acid**					
MW	g/mol	436.58 (Lit)	436.58	[188]	Molecular weight
logP	Log Units	1.45 (Lit)	3.82 (1.45-4.70)	[151, 152, 154, 188, 189]	Lipophilicity
Solubility (pH)	mg/ml	13.09 (6.8) (Lit)	45.1 (0.13-51.5) (6.8)	[154, 157, 188]	Solubility
fu	%	5.68 (Lit)	6.26 (5.48-9.61)	[159]	Fraction unbound in plasma
pKa (acidic)	-	4.21 (Lit)	4.20 (4.18-5.5)	[152, 188–190]	Acid dissociation constant
K_m (CYP3A4)	µmol/l	26.0 (Lit)	26.0 (21.0-29.0)	[191]	CYP3A4 Michaelis-Menten constant
k_{cat} (CYP3A4)	1/min	31.0[c] (Lit)	-	-	CYP3A4 catalytic rate constant
K_m (CYP2C8)	µmol/l	38.6 (Lit)	38.6 (16.0-88.0)	[191, 192]	CYP2C8 Michaelis-Menten constant
k_{cat} (CYP2C8)	1/min	52.3[c] (Lit)	-	-	CYP2C8 catalytic rate constant
K_m (UGT1A1)	µmol/l	349.0 (Lit)	349.0	[193]	UGT1A1 Michaelis-Menten constant
k_{cat} (UGT1A1)	1/min	6.5[c] (Lit)	-	-	UGT1A1 catalytic rate constant
K_m (UGT1A3)	µmol/l	349.0 (Lit)	349.0	[193]	UGT1A3 Michaelis-Menten constant
k_{cat} (UGT1A3)	1/min	6.5[c] (Lit)	-	-	UGT1A3 catalytic rate constant
Liver lactonization rate	l/µmol/min	2.43E-3 (Lit)	2.43E-3[d]	[151]	Liver lactonization rate
K_m (Pgp)	µmol/l	10.0 (Asm)	-	-	Pgp Michaelis-Menten constant
k_{cat} (Pgp)	1/min	50.0 (Fit)	-	-	Pgp transport rate constant
K_m (OATP1B1)	µmol/l	2.00 (Lit)	1.99 (1.17-2.53)	[194]	OATP1B1 Michaelis-Menten constant
k_{cat} (OATP1B1)	1/min	10.25 (Fit)	-	-	OATP1B1 transport rate constant
K_m (OATP1B3)	µmol/l	2.0 (Asm)	-	-	OATP1B3 Michaelis-Menten constant
k_{cat} (OATP1B3)	1/min	2.15 (Fit)	-	-	OATP1B3 transport rate constant
GFR fraction	-	1 (Asm)	-	-	Fraction of filtered drug in the urine
K_i (CYP2C8)	µmol/l	41.1 (Lit)	41.1	[192]	Concentration for half-maximal inhibition
K_i (CYP3A4)	µmol/l	69.6 (Lit)	56.1 (42.6-69.6)	[176, 195]	Concentration for half-maximal inhibition
K_i (BCRP)	µmol/l	18 (Lit)	18	[169]	Concentration for half-maximal inhibition
K_i (OATP1B1)	µmol/l	3.6 (Lit)	3.6	[179]	Concentration for half-maximal inhibition
Intestinal permeability	cm/min	5.92E-07 (Calc)	-	-	Transcellular intestinal permeability
Partition coefficients	-	Diverse	Schmitt	[108]	Cell to plasma partition coefficients

-: not given, asm: assumption, BCRP: breast cancer resistance protein, calc: calculated, CYP: cytochrome P450, fit: optimized during parameter identification, GFR: glomerular filtration rate, lit: literature, OATP: organic anion transporting polypeptide, Pgp: P-glycoprotein, PON3: paraoxonase 3, UGT: UDP-glucuronosyltransferase

[a] median (range)

[b] assumed from other statins

[c] calculated from V_{max}

[d] calculated from liver S9

Table S18: Drug-dependent parameters of the simvastatin PBPK model (adopted from [150]) *(continued)*

Parameter	Unit	Model	Literature[a]	Reference	Description
Cellular permeability	cm/min	1.17E-4 (Calc)	Charge-dependent Schmitt normalized to PK-Sim	[2]	Permeability into the cellular space

-: not given, asm: assumption, BCRP: breast cancer resistance protein, calc: calculated, CYP: cytochrome P450, fit: optimized during parameter identification, GFR: glomerular filtration rate, lit: literature, OATP: organic anion transporting polypeptide, Pgp: P-glycoprotein, PON3: paraoxonase 3, UGT: UDP-glucuronosyltransferase

[a] median (range)

[b] assumed from other statins

[c] calculated from V_{max}

[d] calculated from liver S9

5.4.2 Carbamazepine-simvastatin clinical DDI studies

Table S19: Clinical studies investigating the carbamazepine-simvastatin DDI

Carbamazepine administration		Simvastatin administration		n	Healthy [%]	Females [%]	Age[a] [years]	Weight[a] [kg]	Height [cm]	Reference
**Dose [mg]**	**Route**	**Dose [mg]**	**Route**							
200/300	po (tab), 200 mg bid (D1-D2), 300 mg tid (D3-D14)	80	po (tab), sd (D15)	12	100	0	22-31	66-93	-	Ucar 2004 [196]

-: not given, bid: twice daily, D: day, po: oral, sd: single dose, tab: tablet, tid: three times daily

[a] range

5.4.3 Profiles

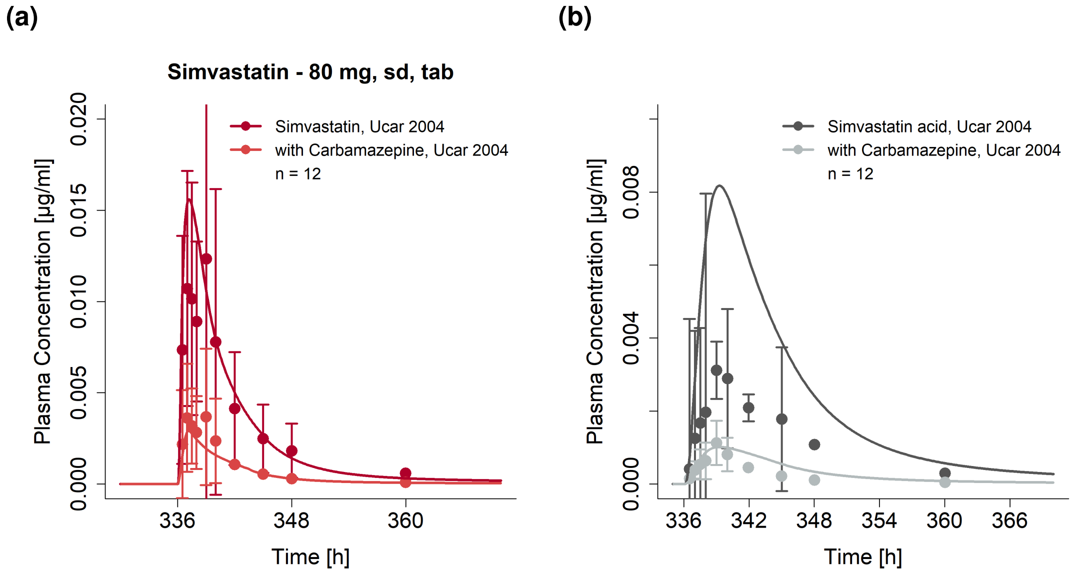

Figure S31: Predicted compared to observed (a) simvastatin and (b) simvastatin acid plasma concentration-time profiles (linear) before and during carbamazepine co-administration. Observed data are shown as dots ± standard deviation; model predictions are shown as solid lines. Details on dosing regimens, study population and literature reference are listed in Table S19. sd: single dose, tab: tablet

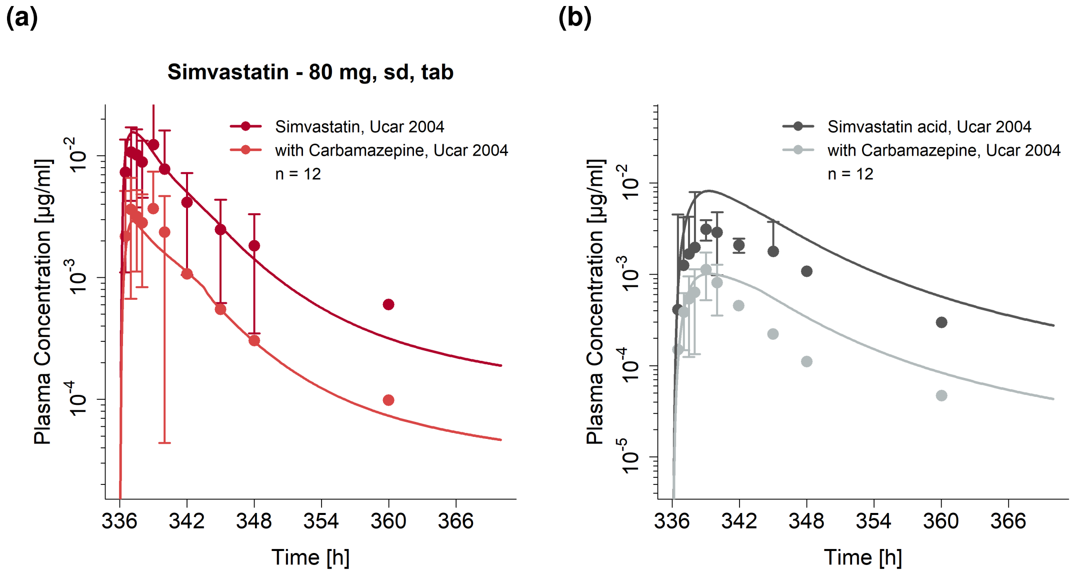

Figure S32: Predicted compared to observed (a) simvastatin and (b) simvastatin acid plasma concentration-time profiles (semi-logarithmic) before and during carbamazepine co-administration. Observed data are shown as dots ± standard deviation; model predictions are shown as solid lines. Details on dosing regimens, study population and literature reference are listed in Table S19. sd: single dose, tab: tablet

5.4.4 DDI AUC_{last} and C_{max} ratio goodness-of-fit plots

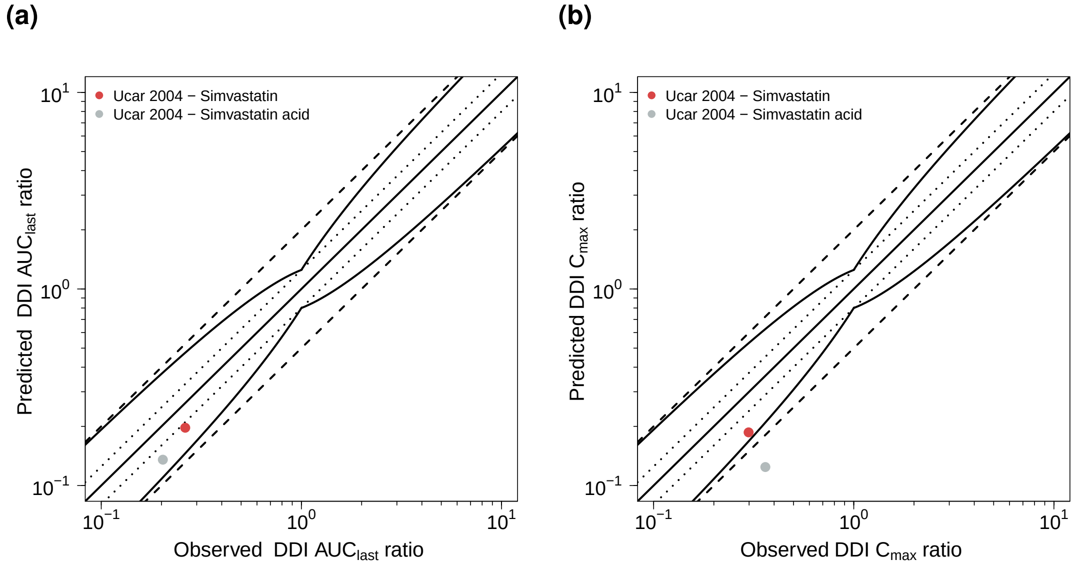

Figure S33: Predicted compared to observed carbamazepine-simvastatin (a) DDI AUC_{last} and (b) DDI C_{max} ratios. The straight solid line marks the line of identity, the curved solid lines show the prediction success limits proposed by Guest et al. allowing for 1.25-fold variability of the DDI ratio [109]. Dotted lines indicate 1.25-fold, dashed lines indicate 2-fold deviation. AUC_{last}: area under the plasma concentration-time curve from the time of drug administration to the last concentration measurement, C_{max}: maximum plasma concentration, DDI: drug-drug interaction

5.4.5 Geometric mean fold error of predicted DDI AUC_{last} and C_{max} ratios

Table S20: Predicted and observed carbamazepine-simvastatin DDI AUC_{last} and C_{max} ratios with geometric mean fold errors

**Perpetrator**	**Victim**				**DDI AUC_{last} ratio**			**DDI C_{max} ratio**			
Carbamazepine	Simvastatin	**Compound**	**Dose gap [h]**	**n**	**Pred**	**Obs**	**Pred/Obs**	**Pred**	**Obs**	**Pred/Obs**	**Reference**
200/300 mg, po, bid/tid	80 mg, po, sd	Simvastatin	12	12	0.20	0.26	0.75	0.19	0.30	0.63	Ucar 2004 [196]
200/300 mg, po, bid/tid	80 mg, po, sd	Simvastatin acid	12	12	0.14	0.20	0.67	0.12	0.36	0.34	Ucar 2004 [196]
**mean GMFE (Simvastatin)**							**1.34**			**1.60**	
							**1/1 with GMFE ≤ 2**			**1/1 with GMFE ≤ 2**	
**mean GMFE (Simvastatin acid)**							**1.50**			**2.91**	
							**1/1 with GMFE ≤ 2**			**0/1 with GMFE ≤ 2**	

AUC_{last}: area under the plasma concentration-time curve from the time of drug administration to the last concentration measurement, bid: twice daily, C_{max}: maximum plasma concentration, DDI: drug-drug interaction, GMFE: geometric mean fold error, obs: observed, po: oral, pred: predicted, sd: single dose, tid: three times daily

5.5 Carbamazepine-bupropion DDI

The carbamazepine-bupropion interaction was modeled using a previously established whole-body parent-metabolite PBPK model of bupropion [197]. Bupropion is a sensitive CYP2B6 substrate, with hydroxybupropion as the main metabolite. CYP2B6 metabolism is described in the model using Michaelis-Menten kinetics. The drug-dependent parameters of the bupropion model are reproduced in Table S21. The k_{cat} for bupropion CYP2B6 metabolism was slightly adjusted (k_{cat} = 40.0 1/min), assuming variability of the CYP2B6 expression in the relatively small DDI study population, to better match the plasma concentration-time profile. The adjusted k_{cat} was then applied for the control and the DDI simulations of this study.

The carbamazepine-bupropion DDI was modeled as induction of bupropion CYP2B6 metabolism by carbamazepine. Induction of CYP2B6 by carbamazepine was described using EC_{50} = 20.0 µmol/l from literature and E_{max} = 17.0 identified during the carbamazepine parameter identification.

Details on the modeled clinical DDI study are given in Table S22. Model predictions of bupropion, hydroxybupropion, erythrohydrobupropion and threohydrobupropion plasma concentration-time profiles before and during carbamazepine co-administration, compared to observed data, are shown in Figures S34 (linear) and S35 (semi-logarithmic). The correlation of predicted to observed DDI AUC_{last} and C_{max} ratios is shown in Figure S36. Table S23 lists the corresponding predicted and observed DDI AUC_{last} ratios, DDI C_{max} ratios, as well as GMFE values.

5.5.1 Bupropion drug-dependent parameters

Table S21: Drug-dependent parameters of the bupropion PBPK model (adopted from [197])

Parameter	Unit	Model	Literature	Reference	Description
**Bupropion**					
MW	g/mol	239.74 (Lit)	239.74	[154]	Molecular weight
logP	-	2.70 (Fit)	3.27	[154]	Lipophilicity
Solubility (pH)	mg/ml	364.56 (7.4) (Lit)	364.56 (7.4)	[198]	Solubility
fu	%	16.0 (Lit)	16.0	[199]	Fraction unbound in plasma
pKa (base)	-	8.75 (Lit)	8.75	[200]	Acid dissociation constant
K_m (CYP2B6)	µmol/l	25.80 (Lit)	25.80[a]	[88]	CYP2B6 Michaelis-Menten constant
k_{cat} (CYP2B6)	1/min	21.74 (Fit)	-	-	CYP2B6 catalytic rate constant for wildtype
K_m (CYP2C19)	µmol/l	8.30 (Lit)	8.30	[201]	CYP2C19 Michaelis-Menten constant
k_{cat} (CYP2C19)	1/min	2.59 (Fit)	-	-	CYP2C19 catalytic rate constant
K_m (11β-HSD1) EBUP	µmol/l	39.1 (Lit)	39.1	[202]	11β-HSD Michaelis-Menten constant for EBUP formation
k_{cat} (11β-HSD1) EBUP	1/min	2.15 (Fit)	-	-	11β-HSD catalytic rate constant for EBUP formation
K_m (11β-HSD1) TBUP	µmol/l	39.1 (Lit)	39.1	[202]	11β-HSD Michaelis-Menten constant for TBUP formation
k_{cat} (11β-HSD1) TBUP	1/min	8.18 (Fit)	-	-	11β-HSD catalytic rate constant for TBUP formation
K_D (NAT1)	µmol/l	0.44 (Lit)	0.44	[203]	NAT1 dissociation constant
k_{off} (NAT1)	1/min	0.05 (Fit)	-	-	NAT1 dissociation rate constant
GFR fraction	-	1 (Asm)	-	-	Fraction of filtered drug in the urine
Intestinal permeability	cm/min	3.30E-05 (Fit)	-	-	Transcellular intestinal permeability
Partition coefficients	-	Diverse	PK-Sim Standard	[79]	Cell to plasma partition coefficients
Cellular permeability	cm/min	0.14 (Calc)	Charge-dependent Schmitt	[2]	Permeability into the cellular space
IR tablet Weibull time	min	3.12 (Fit)	-	-	Dissolution time (50% dissolved)
IR tablet Weibull shape	-	0.75 (Fit)	-	-	Dissolution profile shape
**Hydroxybupropion**					
MW	g/mol	255.74 (Lit)	255.74	[154]	Molecular weight
logP	-	1.90 (Fit)	2.20	[204]	Lipophilicity

-: not given, 11β-HSD1: 11β-hydroxysteroid dehydrogenase 1, asm: assumption, calc: calculated, CYP: cytochrome P450, EBUP: erythrohydrobupropion, fit: optimized during parameter identification, IR: immediate release tablet formulation, GFR: glomerular filtration rate, lit: literature, NAT1: norepinephrine transporter 1, TBUP: threohydrobupropion, UGT: UDP-glucuronosyltransferase

[a] $fu_{incubation}$ was applied to in vitro literature value

Table S21: Drug-dependent parameters of the bupropion PBPK model (adopted from [197]) *(continued)*

Parameter	Unit	Model	Literature	Reference	Description
Solubility (pH)	mg/ml	0.91 (7.4) (Lit)	0.91 (7.4)	[154]	Solubility
fu	%	23.0 (Lit)	23.0	[199]	Fraction unbound in plasma
pKa (base)	-	7.65 (Lit)	7.65	[154]	Acid dissociation constant
K_m (UGT2B7)	µmol/l	14.64 (Lit)	14.64[a]	[205]	UGT2B7 Michaelis-Menten constant
k_{cat} (UGT2B7)	1/min	1.38 (Fit)	-	[205]	UGT2B7 catalytic rate constant
GFR fraction	-	1 (Asm)	-	-	Fraction of filtered drug in the urine
Partition coefficients	-	Diverse	Berezhkovskiy	[187]	Cell to plasma partition coefficients
Cellular permeability	cm/min	0.01 (Calc)	Charge-dependent Schmitt	[2]	Permeability into the cellular space
**Erythrohydrobupropion**					
MW	g/mol	241.76 (Lit)	241.76	[154]	Molecular weight
logP	-	1.76 (Fit)	2.88	[206]	Lipophilicity
Solubility (pH)	mg/ml	82.98 (7.4) (Lit)	82.98 (7.4)	[154]	Solubility
fu	%	58.0 (Lit)	58.0	[199]	Fraction unbound in plasma
pKa (base)	-	9.71 (Lit)	9.71	[154]	Acid dissociation constant
K_m (UGT2B7)	µmol/l	9.33 (Lit)	9.33[a]	[205]	UGT2B7 Michaelis-Menten constant
k_{cat} (UGT2B7)	1/min	0.38 (Fit)	-	-	UGT2B7 catalytic rate constant
GFR fraction	-	1 (Asm)	-	-	Fraction of filtered drug in the urine
Partition coefficients	-	Diverse	Berezhkovskiy	[187]	Cell to plasma partition coefficients
Cellular permeability	cm/min	0.01 (Calc)	Charge-dependent Schmitt	[2]	Permeability into the cellular space
**Threohydrobupropion**					
MW	g/mol	241.76 (Lit)	241.76	[154]	Molecular weight
logP	-	1.76 (Fit)	2.88	[206]	Lipophilicity
Solubility (pH)	mg/ml	82.98 (7.4) (Lit)	82.98 (7.4)	[154]	Solubility
fu	%	58.0 (Lit)	58.0	[199]	Fraction unbound in plasma
pKa (base)	-	9.71 (Lit)	9.71	[154]	Acid dissociation constant
K_m (UGT2B7)	µmol/l	6.22 (Lit)	6.22[a]	[205]	UGT2B7 Michaelis-Menten constant
k_{cat} (UGT2B7)	1/min	0.10 (Fit)	-	-	UGT2B7 catalytic rate constant

-: not given, 11β-HSD1: 11β-hydroxysteroid dehydrogenase 1, asm: assumption, calc: calculated, CYP: cytochrome P450, EBUP: erythrohydrobupropion, fit: optimized during parameter identification, IR: immediate release tablet formulation, GFR: glomerular filtration rate, lit: literature, NAT1: norepinephrine transporter 1, TBUP: threohydrobupropion, UGT: UDP-glucuronosyltransferase

[a] $fu_{incubation}$ was applied to in vitro literature value

Table S21: Drug-dependent parameters of the bupropion PBPK model (adopted from [197]) *(continued)*

Parameter	Unit	Model	Literature	Reference	Description
GFR fraction	-	1 (Asm)	-	-	Fraction of filtered drug in the urine
Partition coefficients	-	Diverse	Berezhkovskiy	[187]	Cell to plasma partition coefficients
Cellular permeability	cm/min	0.01 (Calc)	Charge-dependent Schmitt	[2]	Permeability into the cellular space

-: not given, 11β-HSD1: 11β-hydroxysteroid dehydrogenase 1, asm: assumption, calc: calculated, CYP: cytochrome P450, EBUP: erythrohydrobupropion, fit: optimized during parameter identification, IR: immediate release tablet formulation, GFR: glomerular filtration rate, lit: literature, NAT1: norepinephrine transporter 1, TBUP: threohydrobupropion, UGT: UDP-glucuronosyltransferase

[a] $fu_{incubation}$ was applied to in vitro literature value

5.5.2 Carbamazepine-bupropion clinical DDI studies

Table S22: Clinical studies investigating the carbamazepine-bupropion DDI

Carbamazepine administration		Bupropion administration		n	Healthy [%]	Females [%]	Age[a] [years]	Weight [kg]	Height [cm]	Reference
Dose [mg]	Route	Dose [mg]	Route							
-	-	150	po (tab), sd	17	100	53	38.5	-	-	Ketter 1995[d] [207]
314[b]	po (-), tid[b]	150	po (tab), sd	12	0[c]	40	35.4	-	-	Ketter 1995[e] [207]

-: not given, po: oral, sd: single dose, tab: tablet, tid: three times daily, qid: four times daily

[a] mean

[b] patients received a mean dose of 942 mg per day, in three to four doses

[c] patients with major affective disorders on chronic carbamazepine treatment for at least 3 weeks before bupropion intake

[d] control arm of the study

[e] DDI arm of the study

5.5.3 Profiles

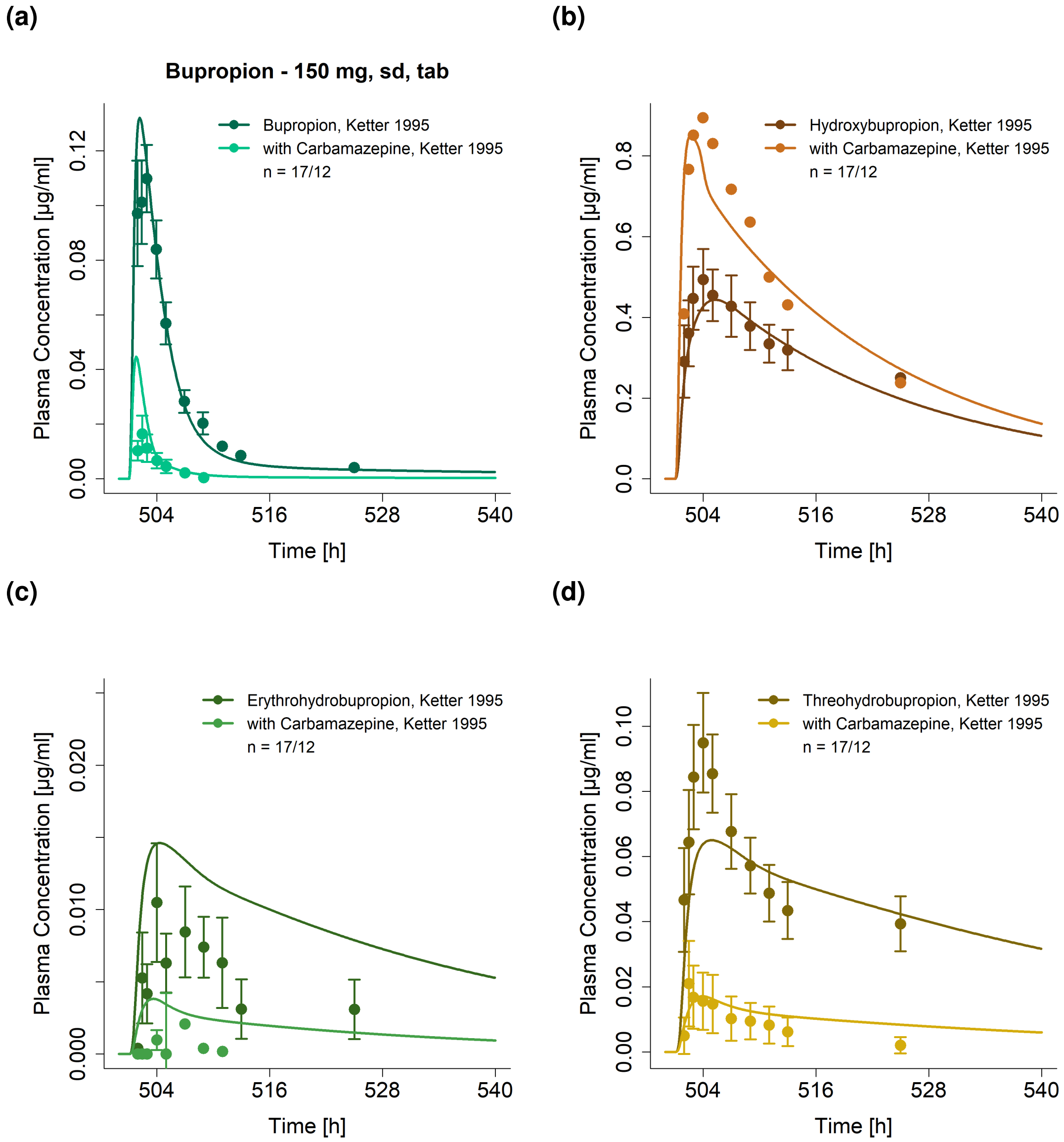

Figure S34: Predicted compared to observed (a) bupropion, (b) hydroxybupropion, (c) erythrohydrobupropion and (d) threohydrobupropion plasma concentration-time profiles (linear) before and during carbamazepine co-administration. Observed data are shown as dots ± standard deviation; model predictions are shown as solid lines. Details on dosing regimens, study populations and literature reference are listed in Table S22. sd: single dose, tab: tablet

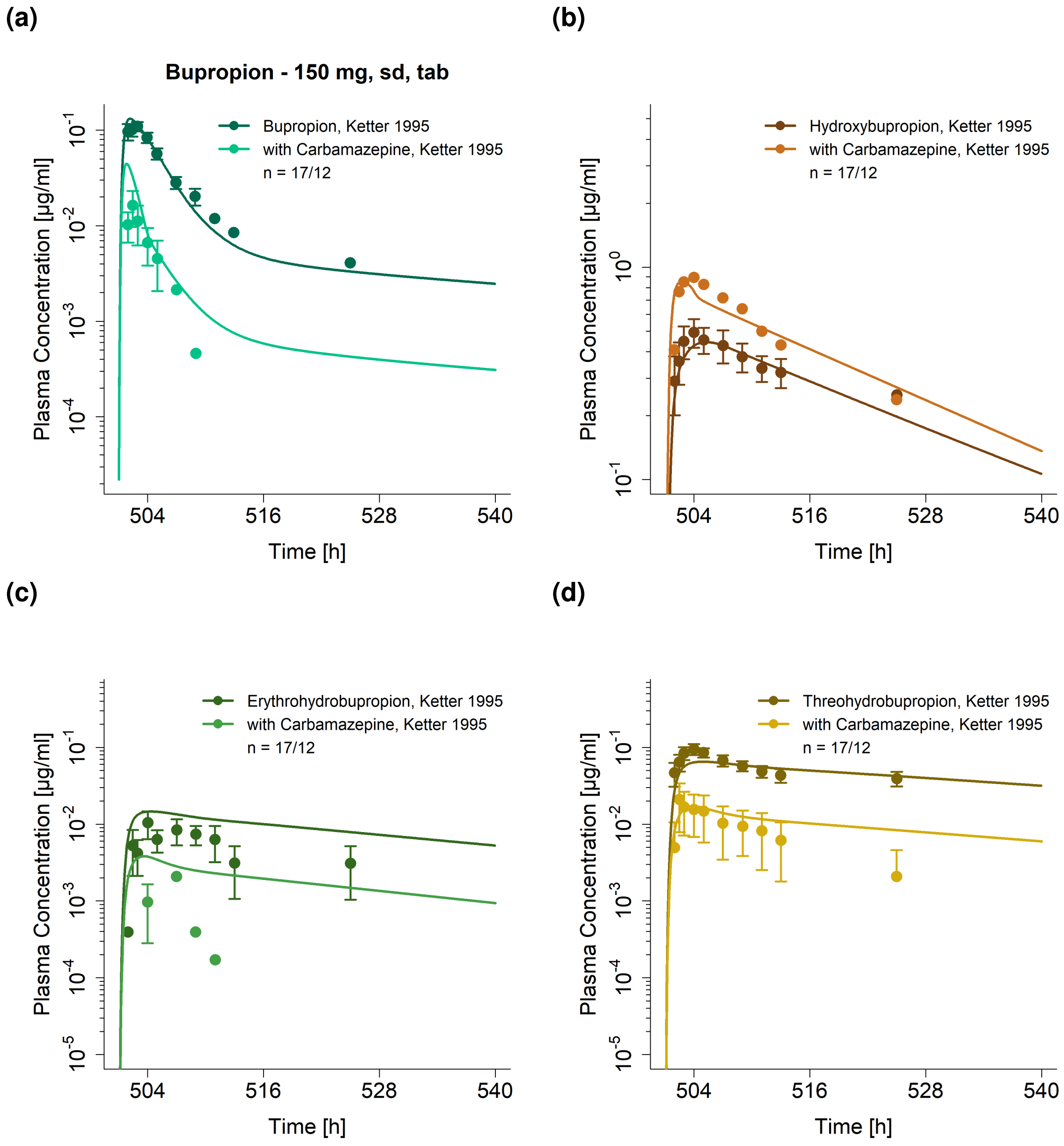

Figure S35: Predicted compared to observed (a) bupropion, (b) hydroxybupropion, (c) erythrohydrobupropion (d) and threohydrobupropion plasma concentration-time profiles (semi-logarithmic) before and during carbamazepine co-administration. Observed data are shown as dots ± standard deviation; model predictions are shown as solid lines. Details on dosing regimens, study populations and literature reference are listed in Table S22. sd: single dose, tab: tablet

5.5.4 DDI AUC_{last} and C_{max} ratio goodness-of-fit plots

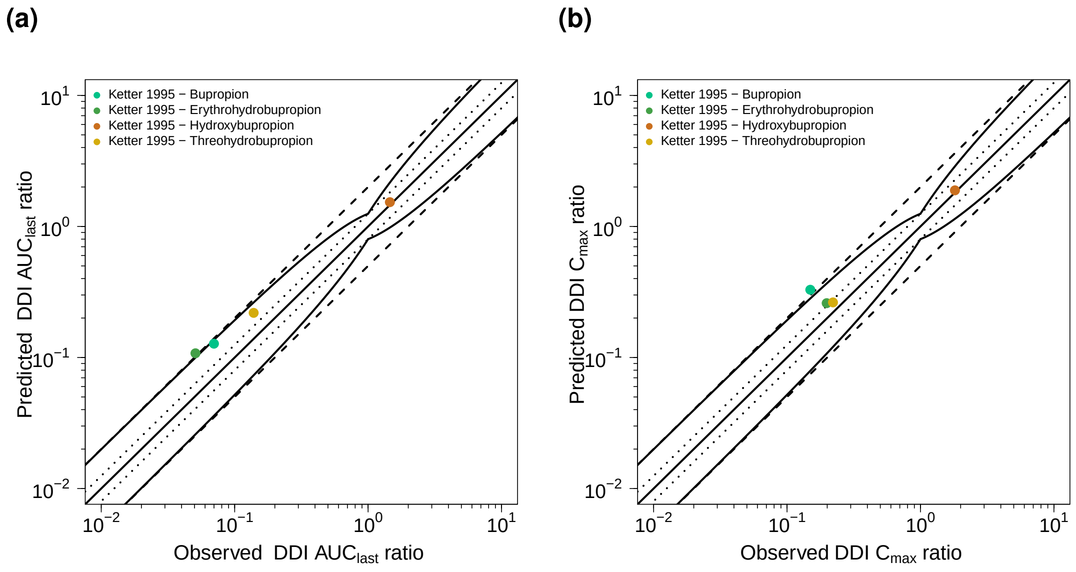

Figure S36: Predicted compared to observed carbamazepine-bupropion (a) DDI AUC_{last} and (b) DDI C_{max} ratios. The straight solid line marks the line of identity, the curved solid lines show the prediction success limits proposed by Guest et al. allowing for 1.25-fold variability of the DDI ratio [109]. Dotted lines indicate 1.25-fold, dashed lines indicate 2-fold deviation. AUC_{last}: area under the plasma concentration-time curve from the time of drug administration to the last concentration measurement, C_{max}: maximum plasma concentration, DDI: drug-drug interaction

5.5.5 Geometric mean fold error of predicted DDI AUC_{last} and C_{max} ratios

Table S23: Predicted and observed carbamazepine-bupropion DDI AUC_{last} and C_{max} ratios with geometric mean fold errors

**Perpetrator**	**Victim**				**DDI AUC_{last} ratio**			**DDI C_{max} ratio**			
Carbamazepine	Bupropion	**Compound**	**Dose gap [h]**	**n**	**Pred**	**Obs**	**Pred/Obs**	**Pred**	**Obs**	**Pred/Obs**	**Reference**
314 mg, po, tid	150 mg, po, sd	Bupropion	3	17/12	0.13	0.07	1.82	0.33	0.15	2.20	Ketter 1995 [207]
314 mg, po, tid	150 mg, po, sd	Hydroxybupropion	3	17/12	1.53	1.46	1.05	1.89	1.81	1.04	Ketter 1995 [207]
314 mg, po, tid	150 mg, po, sd	Erythrohydrobupropion	3	17/12	0.11	0.05	2.12	0.26	0.20	1.31	Ketter 1995 [207]
314 mg, po, tid	150 mg, po, sd	Threohydrobupropion	3	17/12	0.22	0.14	1.58	0.26	0.22	1.19	Ketter 1995 [207]
**mean GMFE (Bupropion)**							**1.82**			**2.20**	
							**1/1 with GMFE ≤ 2**			**0/1 with GMFE ≤ 2**	
**mean GMFE (Hydroxybupropion)**							**1.05**			**1.04**	
							**1/1 with GMFE ≤ 2**			**1/1 with GMFE ≤ 2**	
**mean GMFE (Erythrohydrobupropion)**							**2.12**			**1.31**	
							**0/1 with GMFE ≤ 2**			**1/1 with GMFE ≤ 2**	
**mean GMFE (Threohydrobupropion)**							**1.58**			**1.19**	
							**1/1 with GMFE ≤ 2**			**1/1 with GMFE ≤ 2**	

AUC_{last}: area under the plasma concentration-time curve from the time of drug administration to the last concentration measurement, C_{max}: maximum plasma concentration, DDI: drug-drug interaction, GMFE: geometric mean fold error, obs: observed, po: oral, pred: predicted, sd: single dose, tid: three times daily

5.6 Efavirenz-carbamazepine DDI

The efavirenz-carbamazepine DDI was modeled using a previously developed whole-body PBPK model of efavirenz, available in the OSP GitHub model repository (https://github.com/Open-Systems-Pharmacology/Efavirenz-Model), which was updated prior to DDI modeling with carbamazepine as described in Sections 3 and 6. Efavirenz is substrate and inducer of CYP3A4 and CYP2B6. The drug-dependent parameters of the updated efavirenz model are shown in Table S6.

The efavirenz-carbamazepine DDI was modeled as induction and simultaneous competitive inhibition of carbamazepine CYP3A4 and CYP2B6 metabolism by efavirenz and as induction of efavirenz CYP3A4 and CYP2B6 metabolism by carbamazepine. K_i = 9.67 µmol/l and K_i = 0.40 µmol/l, describing the competitive CYP3A4 and CYP2B6 inhibition by efavirenz, respectively, were taken from literature and corrected for binding in the in vitro assay, as described in Section 3. EC_{50} = 0.23 µmol/l to describe the CYP3A4 and CYP2B6 induction by efavirenz was taken from literature and corrected for binding in the in vitro assay, E_{max} = 12.0 (CYP3A4) and E_{max} = 8.13 (CYP2B6), were identified during parameter identification. EC_{50} = 20.0 µmol/l describing CYP3A4 and CYP2B6 induction by carbamazepine was taken from literature. E_{max} = 6.0 (CYP3A4) and E_{max} = 17.0 (CYP2B6), were optimized during parameter identification.

Details on the modeled clinical DDI study are given in Table S24. Model predictions of carbamazepine, carbamazepine-10-11-epoxide and efavirenz plasma concentration-time profiles before and during co-administration, compared to observed data, are shown in Figures S37 (linear) and S38 (semi-logarithmic). The correlation of predicted to observed DDI AUC_{last} and C_{max} ratios is shown in Figure S39. Table S25 lists the corresponding predicted and observed DDI AUC_{last} ratios, DDI C_{max} ratios, as well as GMFE values.

5.6.1 Efavirenz-carbamazepine clinical DDI studies

Table S24: Clinical studies investigating the efavirenz-carbamazepine DDI

Efavirenz administration		Carbamazepine administration		n	Healthy [%]	Females [%]	Age[a] [years]	Weight[a] [kg]	Height [cm]	Reference
**Dose [mg]**	**Route**	**Dose [mg]**	**Route**							
600	po (tab), qd (D22-D35)	200/400	po (tab), 200 mg, qd (D1-D3),<br>mg, bid (D4-D6),<br>400 mg, qd (D7-D35)	36	100	31	20-45	54-92	-	Ji 2008[b] [44]
600	po (tab), qd (D1-D35)	200/400	po (tab), 200 mg, qd (D15-D17),<br>mg, bid (D18-D20),<br>400 mg, qd (D21-D35)	36	100	31	20-45	54-92	-	Ji 2008[c] [44]

-: not given, bid: twice daily, D: day, po: oral, qd: once daily, tab: tablet

[a] range

[b] Carbamazepine as victim drug

[c] Efavirenz as victim drug

5.6.2 Profiles

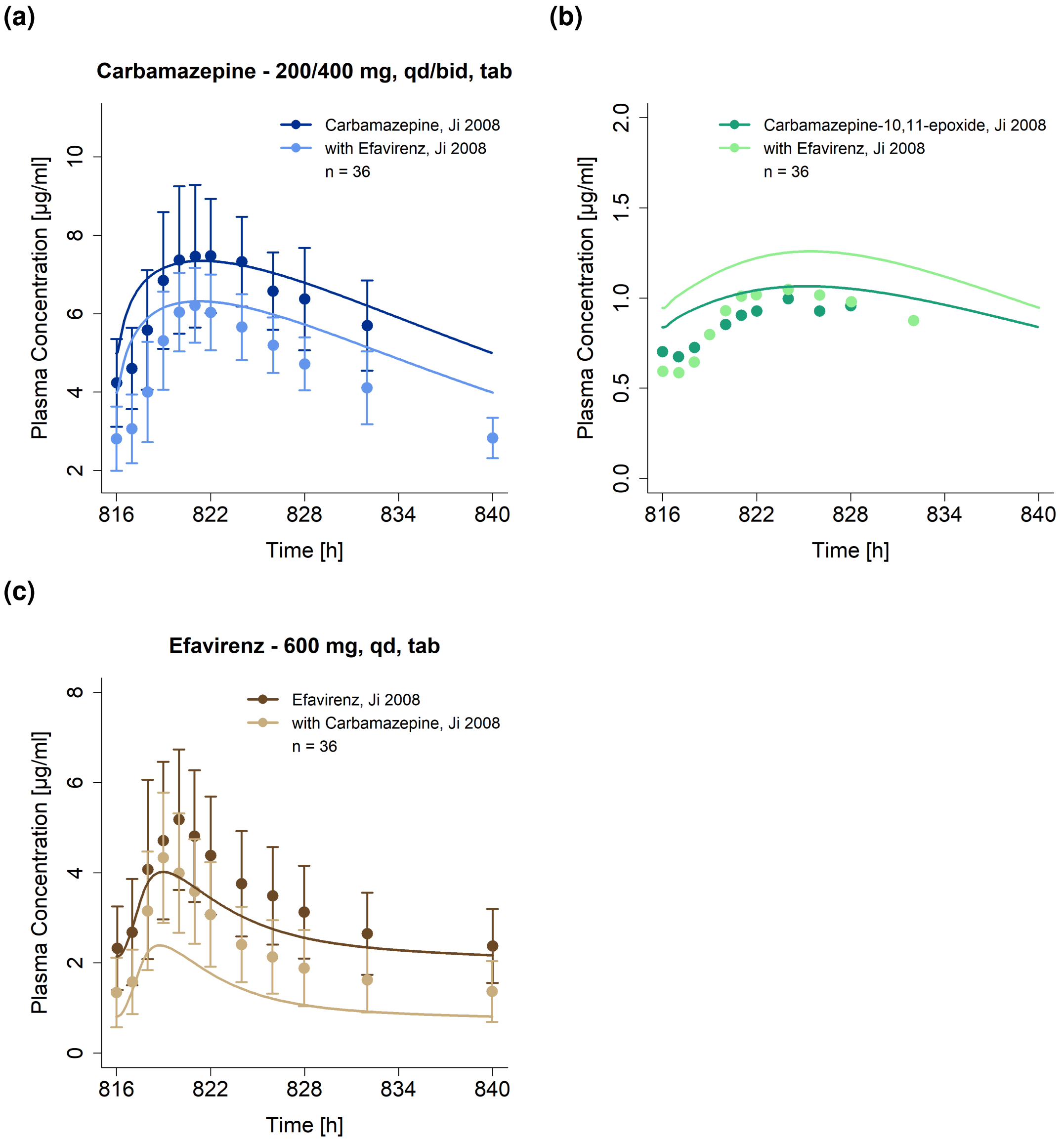

Figure S37: Predicted compared to observed (a) carbamazepine, (b) carbamazepine-10-11-epoxide and (c) efavirenz plasma concentration-time profiles (linear) before and during co-administration. Observed data are shown as dots ± standard deviation; model predictions are shown as solid lines. Details on dosing regimens, study population and literature reference are listed in Table S24. bid: twice daily, qd: once daily, tab: tablet

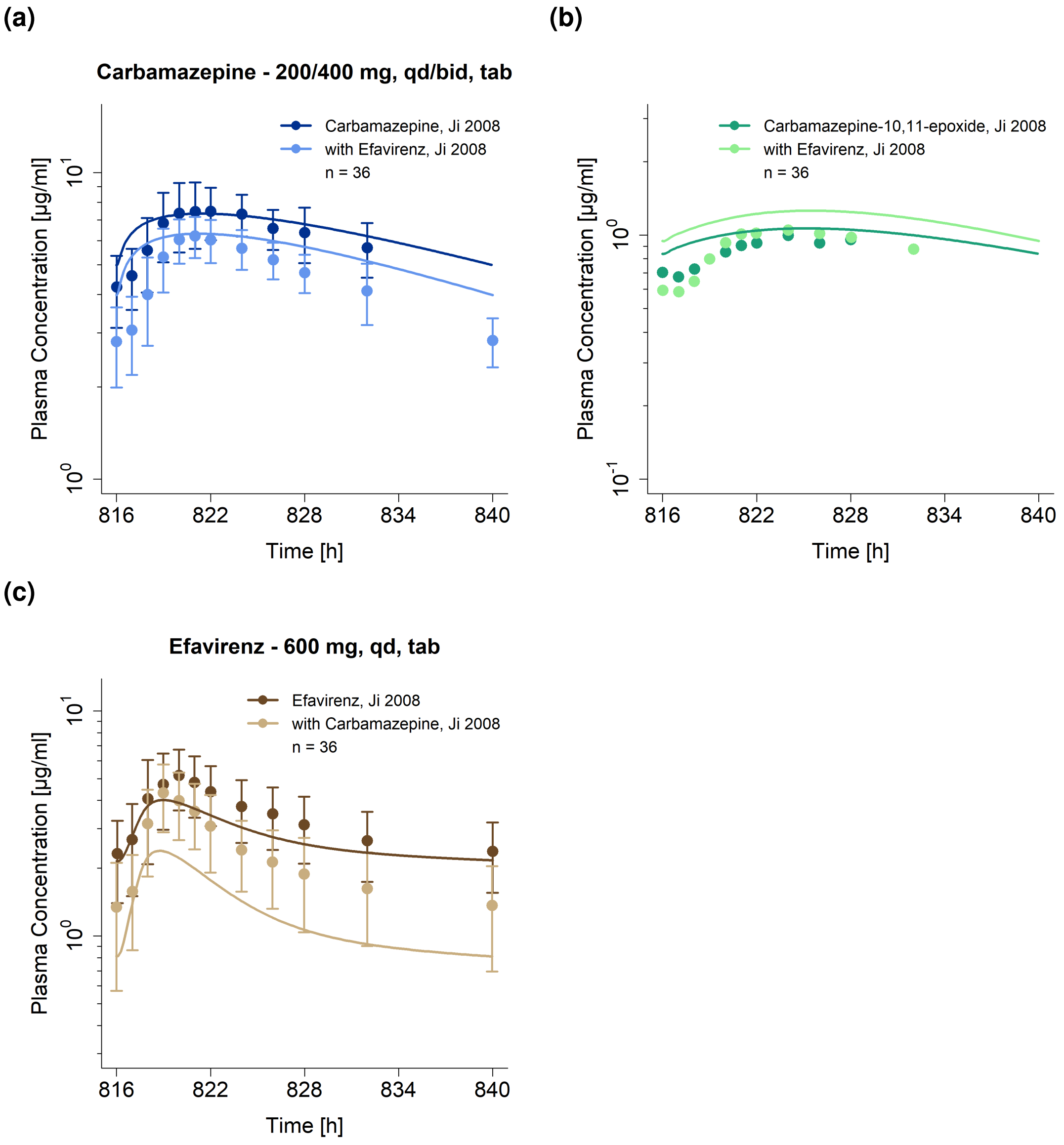

Figure S38: Predicted compared to observed (a) carbamazepine, (b) carbamazepine-10-11-epoxide and (c) efavirenz plasma concentration-time profiles (semi-logarithmic) before and during co-administration. Observed data are shown as dots ± standard deviation; model predictions are shown as solid lines. Details on dosing regimens, study population and literature reference are listed in Table S24. bid: twice daily, qd: once daily, tab: tablet

5.6.3 DDI AUC_{last} and C_{max} ratio goodness-of-fit plots

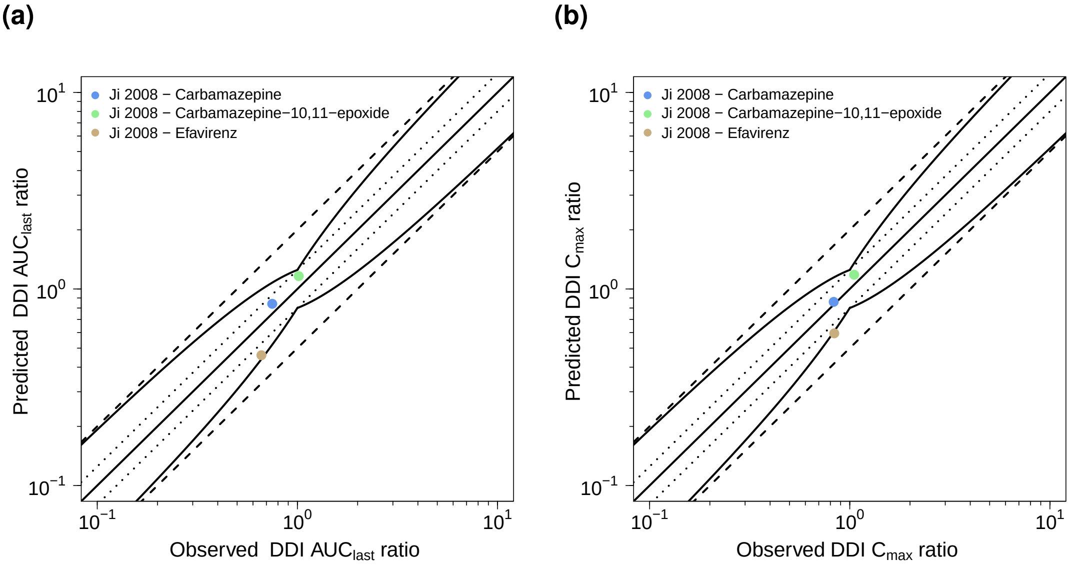

Figure S39: Predicted compared to observed efavirenz-carbamazepine (a) DDI AUC_{last} and (b) DDI C_{max} ratios. The straight solid line marks the line of identity, the curved solid lines show the prediction success limits proposed by Guest et al. allowing for 1.25-fold variability of the DDI ratio [109]. Dotted lines indicate 1.25-fold, dashed lines indicate 2-fold deviation. AUC_{last}: area under the plasma concentration-time curve from the time of drug administration to the last concentration measurement, C_{max}: maximum plasma concentration, DDI: drug-drug interaction

5.6.4 Geometric mean fold error of predicted DDI AUC_{last} and C_{max} ratios

Table S25: Predicted and observed efavirenz-carbamazepine DDI AUC_{last} and C_{max} ratios with geometric mean fold errors

					DDI AUC_{last} ratio			DDI C_{max} ratio			
**Perpetrator**	**Victim**	**Compound**	**Dose gap [h]**	**n**	**Pred**	**Obs**	**Pred/Obs**	**Pred**	**Obs**	**Pred/Obs**	**Reference**
Efavirenz	*Carbamazepine*										
600 mg, po, qd	200/400 mg, po, bid/qd	Carbamazepine	0	36	0.84	0.75	1.12	0.86	0.83	1.03	Ji 2008 [44]
600 mg, po, qd	200/400 mg, po, bid/qd	Carbamazepine-10,11-epoxide	0	36	1.16	1.02	1.14	1.18	1.05	1.12	Ji 2008 [44]
Carbamazepine	*Efavirenz*										
200/400 mg, po, bid/qd	600 mg, po, qd	Efavirenz	0	36	0.46	0.66	0.70	0.59	0.84	0.71	Ji 2008 [44]
**mean GMFE (Carbamazepine)**							**1.12**			**1.03**	
							**1/1 with GMFE ≤ 2**			**1/1 with GMFE ≤ 2**	
**mean GMFE (Carbamazepine-10,11-epoxide)**							**1.14**			**1.12**	
							**1/1 with GMFE ≤ 2**			**1/1 with GMFE ≤ 2**	
**mean GMFE (Efavirenz)**							**1.44**			**1.41**	
							**1/1 with GMFE ≤ 2**			**1/1 with GMFE ≤ 2**	

AUC_{last}: area under the plasma concentration-time curve from the time of drug administration to the last concentration measurement, bid: twice daily, C_{max}: maximum plasma concentration, DDI: drug-drug interaction, GMFE: geometric mean fold error, md: multiple-dose, obs: observed, po: oral, pred: predicted, qd: once daily

6 Efavirenz drug-drug interactions (DDI)

6.1 DDI modeling - general

A total number of 9 different clinical DDI studies was utilized to evaluate the DDI performance of the efavirenz PBPK model, including studies with CYP3A4 victim drugs (midazolam and alfentanil), a CYP2B6 victim drug (bupropion), a CYP3A4 and CYP2B6 perpetrator (rifampin) and a simultaneous CYP3A4 substrate and inhibitor and CYP2B6 inhibitor (voriconazole). The efavirenz DDI network is illustrated in Figure S40. The implementation of the DDIs is described in more detail in the following sections.

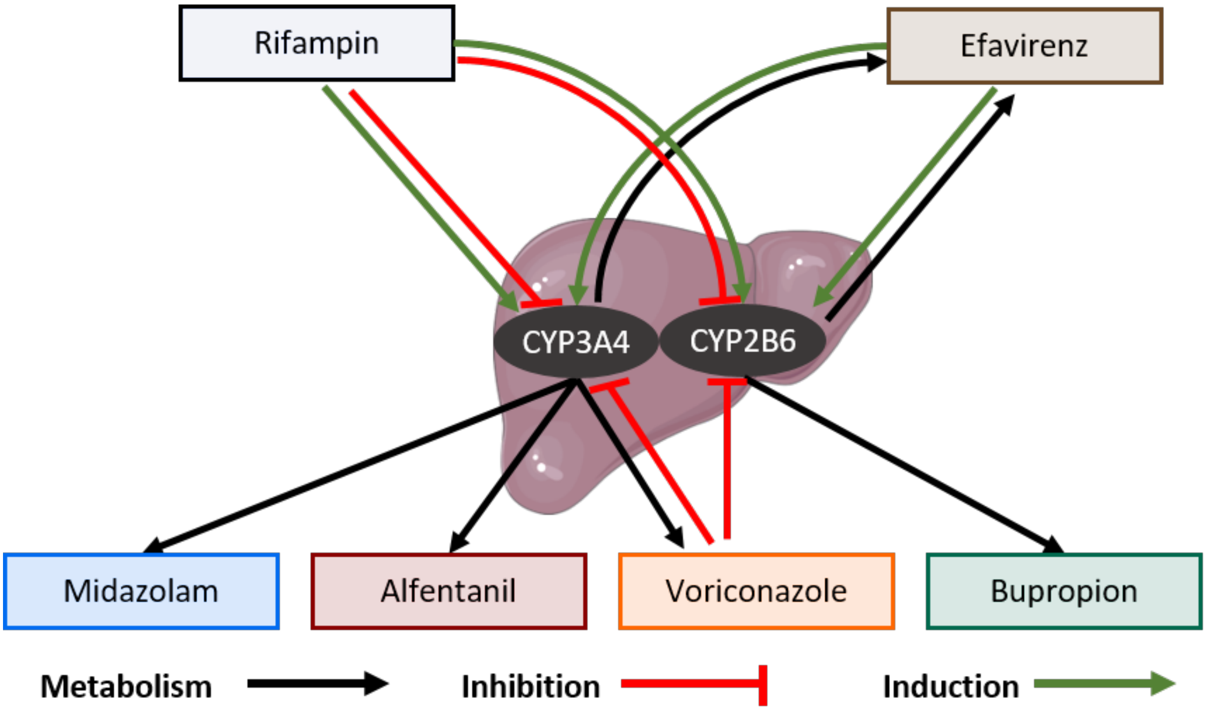

Figure S40: Efavirenz drug-drug interaction network. Black arrows represent metabolism of the compounds, red and green arrows represent inhibition or induction of the CYP enzymes, respectively

The parameters describing the induction of CYP3A4 and CYP2B6 by efavirenz were already introduced during efavirenz model building, as the compound induces its own metabolism. While the efavirenz-midazolam DDI study by Mikus et al. 2017 [208] was used in the training dataset to inform the parametrization of the efavirenz CYP3A4 induction, all other DDIs were purely predictive.

6.2 Efavirenz-midazolam DDI

The ability of the efavirenz model to predict the available clinical efavirenz-midazolam DDI data was already evaluated with the original efavirenz PBPK model [87], using a previously developed whole-body PBPK model of midazolam, available in the OSP GitHub model repository (https://github.com/Open-Systems-Pharmacology/Midazolam-Model). The metabolism of the sensitive CYP3A4 substrate midazolam is described using Michaelis-Menten kinetics [209]. The drug-dependent parameters of the midazolam model are reproduced in Table S26.

The efavirenz-midazolam DDI was modeled as induction with simultaneous competitive inhibition of midazolam CYP3A4 metabolism by efavirenz. The inhibition was described using K_i = 9.67 µmol/l from literature, corrected for binding in the in vitro assay, as described in Section 3. The induction was described using EC_{50} = 0.23 µmol/l (taken from literature and corrected for $fu_{incubation}$). The efavirenz-midazolam DDI study by Mikus et al. [208] was used in the training dataset to inform the parametrization of CYP3A4 E_{max} = 12.0.

Details on the modeled clinical DDI studies are given in Table S27. Model predictions of midazolam plasma concentration-time profiles before and during efavirenz co-administration, compared to observed data, are shown in Figures S41 (linear) and S42 (semi-logarithmic). The correlation of predicted to observed DDI AUC_{last} and C_{max} ratios is shown in Figure S43. Tables S28 and S29 list the corresponding predicted and observed DDI AUC_{last} ratios, DDI C_{max} ratios, as well as GMFE values for the updated and the original efavirenz PBPK model, respectively.

6.2.1 Midazolam drug-dependent parameters

Table S26: Drug-dependent parameters of the midazolam PBPK model (adopted from [209] and [210])

Parameter	Unit	Model	Literature	Reference	Description
MW	g/mol	325.77 (Lit)	325.77	[211]	Molecular weight
logP	log Units	2.89 (Fit)	3.00, 3.10, 3.37, 3.53	[212–215]	Lipophilicity
Solubility (pH)	mg/ml	0.05 (6.5) (Lit)	0.09 (5.0), 0.13 (5.0), 0.05 (6.5),	[216]	Solubility
fu	%	3.10 (Lit)	2.20, 3.10, 3.10	[158, 217–219]	Fraction unbound in plasma
pKa (base)	-	6.20 (Lit)	6.20	[212]	Acid dissociation constant
pKa (acid)	-	10.95 (Lit)	10.95	[212]	Acid dissociation constant
K_m (CYP3A4)	µmol/l	4.00 (Lit)	2.16, 2.69, 3.80, 3.90, 4.00	[212, 214, 220–222]	CYP3A4 Michaelis-Menten constant
k_{cat} (CYP3A4)	1/min	8.76 (Fit)	-	-	CYP3A4 catalytic rate constant
K_m (UGT1A4)	µmol/l	37.8 (Lit)	37.8	[223]	UGT1A4 Michaelis-Menten constant
k_{cat} (UGT1A4)	1/min	3.59 (Fit)	-	-	UGT1A4 catalytic rate constant
K_D (GABRG2)	nmol/l	1.80 (Lit)	1.8	[224]	GABRG2 dissociation constant
GFR fraction	-	0.64 (Fit)	-	-	Fraction of filtered drug in the urine
k_{off} (GABRG2)	1/min	1 (Asm)	-	-	GABRG2 dissociation rate constant
Intestinal permeability	cm/min	1.55E-04 (Fit)	-	-	Transcellular intestinal permeability
Partition coefficients	-	diverse	Rodgers and Rowland	[77, 78]	Cell to plasma partition coefficients
Cellular permeability	cm/min	0.04 (Calc)	PK-Sim Standard	[2]	Permeability into the cellular space
Tablet Weibull time	min	0.01 (Fit)	-	-	Dissolution time (50% dissolved)
Tablet Weibull shape	-	4.38 (Fit)	-	-	Dissolution profile shape

-: not given, asm: assumption, calc: calculated, CYP: cytochrome P450, UGT: UDP-glucuronosyltransferase, fit: optimized during parameter optimization, GABRG2: gamma-aminobutyric acid receptor subunit gamma 2, GFR: glomerular filtration rate, lit: literature

6.2.2 Efavirenz-midazolam clinical DDI studies

Table S27: Clinical studies investigating the efavirenz-midazolam DDI

Efavirenz administration		Midazolam administration		n	Healthy [%]	Females [%]	Age [years]	Weight [kg]	Height [cm]	Reference
**Dose [mg]**	**Route**	**Dose [mg]**	**Route**							
400	po (tab), qd (D1-D14)	3	po (sol), (D13)	12	-	-	-	-	-	Katzenmaier 2010 [225]
400	po (tab), sd (D1)	4	po (sol), sd (D2)	12	100	50	21-34	-	-	Mikus 2017 [208]
		2	iv, sd (D2, 6 h after po)							

-: not given, D: day, iv: intravenous, po: oral, qd: once daily, sd: single dose, sol: solution, tab: tablet

6.2.3 Profiles

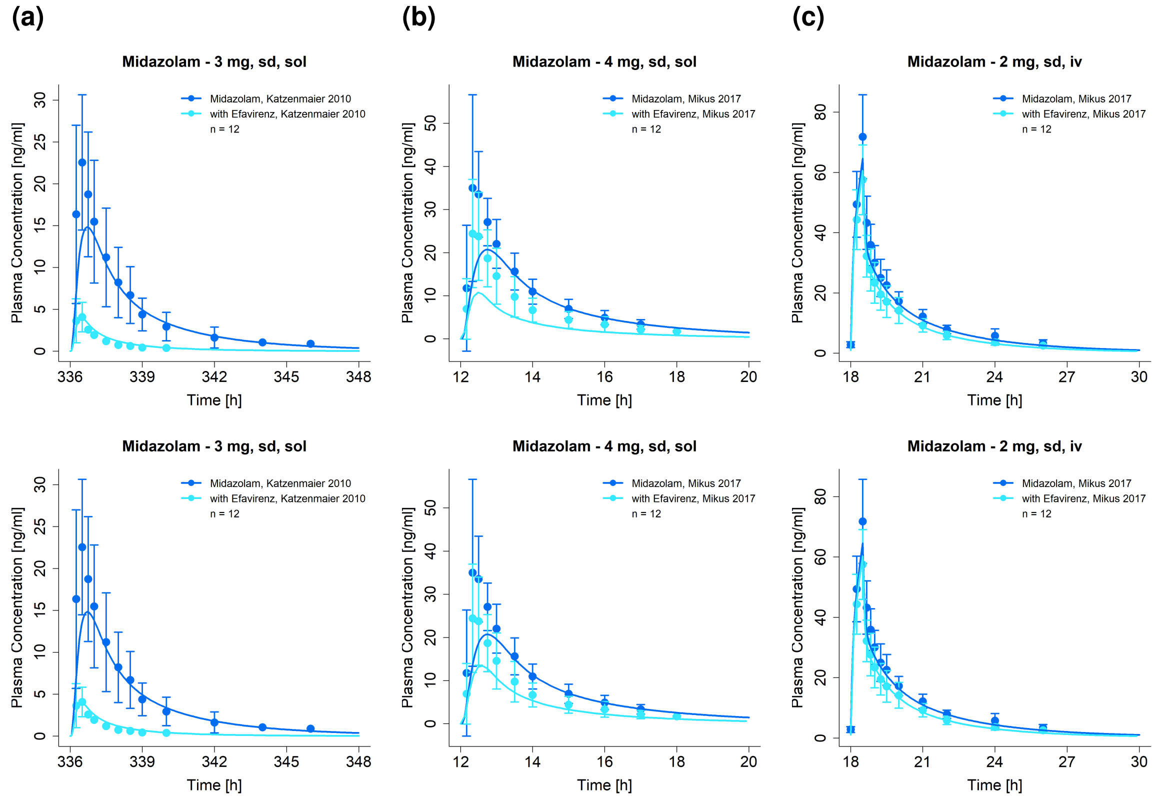

Figure S41: Predicted compared to observed midazolam plasma concentration-time profiles (linear) before and during efavirenz co-administration, predicted with the updated efavirenz model (upper row) or the original efavirenz model (lower row). Observed data are shown as dots ± standard deviation; model predictions are shown as solid lines. Details on dosing regimens, study populations and literature references are listed in Table S27. iv: intravenous, sd: single dose, sol: solution

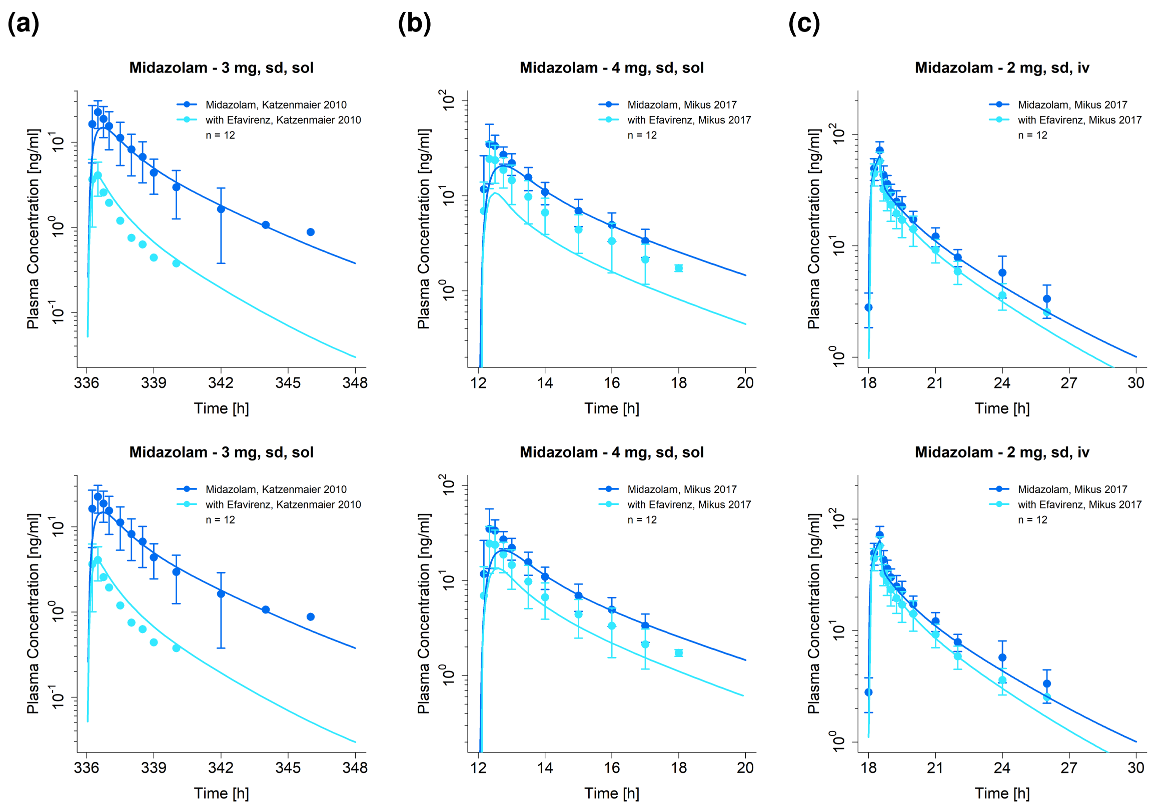

Figure S42: Predicted compared to observed midazolam plasma concentration-time profiles (semi-logarithmic) before and during efavirenz co-administration, predicted with the updated efavirenz model (upper row) or the original efavirenz model (lower row). Observed data are shown as dots ± standard deviation; model predictions are shown as solid lines. Details on dosing regimens, study populations and literature references are listed in Table S27. iv: intravenous, sd: single dose, sol: solution

6.2.4 DDI AUC_{last} and C_{max} ratio goodness-of-fit plots

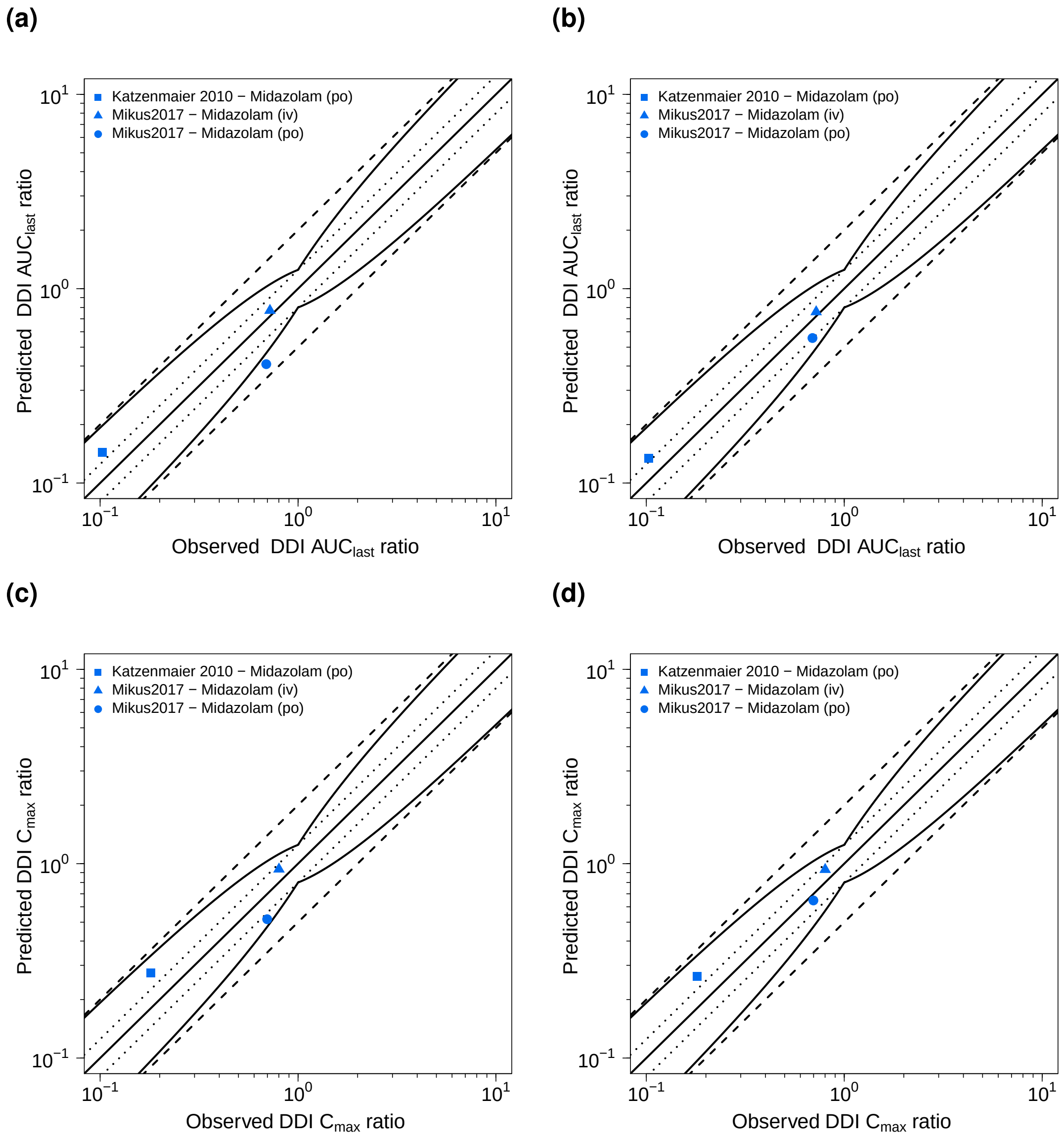

Figure S43: Predicted compared to observed efavirenz-midazolam (a,b) DDI AUC_{last} and (c,d) DDI C_{max} ratios, predicted with the updated efavirenz PBPK model (left) or the original PBPK model (right). The straight solid line marks the line of identity, the curved solid lines show the prediction success limits proposed by Guest et al. allowing for 1.25-fold variability of the DDI ratio [109]. Dotted lines indicate 1.25-fold, dashed lines indicate 2-fold deviation. AUC_{last}: area under the plasma concentration-time curve from the time of drug administration to the last concentration measurement, C_{max}: maximum plasma concentration, DDI: drug-drug interaction, iv: intravenous, po: oral

6.2.5 Geometric mean fold error of predicted DDI AUC_{last} and C_{max} ratios

Table S28: Predicted and observed efavirenz-midazolam DDI AUC_{last} and C_{max} ratios with geometric mean fold errors of the updated model

**Perpetrator**	**Victim**			**DDI AUC_{last} ratio**			**DDI C_{max} ratio**			
Efavirenz	Midazolam	**Dose gap [h]**	**n**	**Pred**	**Obs**	**Pred/Obs**	**Pred**	**Obs**	**Pred/Obs**	**Reference**
400 mg, po, qd	3 mg, po, sd	-	12	0.15	0.10	1.46	0.28	0.18	1.57	Katzenmaier 2010 [225]
400 mg, po, sd	4 mg, po, sd	12	12	0.42	0.69	0.61	0.53	0.70	0.75	Mikus 2017 [208]
400 mg, po, sd	2 mg, iv, sd	18	12	0.78	0.72	1.08	0.94	0.80	1.17	Mikus 2017 [208]
**mean GMFE (range)**						**1.40 (1.08-1.65)**			**1.36 (1.17-1.57)**	
						**3/3 with GMFE ≤ 2**			**3/3 with GMFE ≤ 2**	

-: not given, AUC_{last}: area under the plasma concentration-time curve from the time of drug administration to the last concentration measurement, C_{max}: maximum plasma concentration, DDI: drug-drug interaction, GMFE: geometric mean fold error, iv: intravenous, obs: observed, po: oral, pred: predicted, qd: once daily, sd: single dose

Table S29: Predicted and observed efavirenz-midazolam DDI AUC_{last} and C_{max} ratios with geometric mean fold errors of the original model

**Perpetrator**	**Victim**			**DDI AUC_{last} ratio**			**DDI C_{max} ratio**			
Efavirenz	Midazolam	**Dose gap [h]**	**n**	**Pred**	**Obs**	**Pred/Obs**	**Pred**	**Obs**	**Pred/Obs**	**Reference**
400 mg, po, qd	3 mg, po, sd	-	12	0.13	0.10	1.31	0.26	0.18	1.46	Katzenmaier 2010 et al. [225]
400 mg, po, sd	4 mg, po, sd	12	12	0.56	0.69	0.81	0.65	0.70	0.93	Mikus 2017 et al. [208]
400 mg, po, sd	2 mg, iv, sd	18	12	0.76	0.72	1.05	0.93	0.80	1.16	Mikus 2017 et al. [208]
**mean GMFE (range)**						**1.20 (1.05-1.31)**			**1.23 (1.08-1.46)**	
						**3/3 with GMFE ≤ 2**			**3/3 with GMFE ≤ 2**	

-: not given, AUC_{last}: area under the plasma concentration-time curve from the time of drug administration to the last concentration measurement, C_{max}: maximum plasma concentration, DDI: drug-drug interaction, GMFE: geometric mean fold error, iv: intravenous, obs: observed, po: oral, pred: predicted, qd: once daily, sd: single dose

6.3 Efavirenz-alfentanil DDI

The ability of the efavirenz model to predict the available clinical efavirenz-alfentanil DDI data was already evaluated with the original efavirenz PBPK model [87], using a previously developed whole-body PBPK model of alfentanil [210, 226] available in the OSP GitHub model repository (https://github.com/Open-Systems-Pharmacology/Alfentanil-Model). The metabolism of the sensitive CYP3A4 substrate is described in the model by a specific first-order CYP3A4 clearance (CL_{spec}). The drug-dependent parameters of the alfentanil model are reproduced in Table S30.

The efavirenz-alfentanil DDI was modeled as induction with simultaneous competitive inhibition of alfentanil CYP3A4 metabolism by efavirenz. The inhibition was described using K_i = 9.67 µmol/l from literature, corrected for binding in the in vitro assay, as described in Section 3. The induction was described using EC_{50} = 0.23 µmol/l (taken from literature and corrected for $fu_{incubation}$).

Details on the modeled clinical DDI studies are given in Table S31. Model predictions of alfentanil plasma concentration-time profiles before and during efavirenz co-administration, compared to observed data, are shown in Figures S44 (linear) and S45 (semi-logarithmic). The correlation of predicted to observed DDI AUC_{last} and C_{max} ratios is shown in Figure S52. Tables S32 and S33 list the corresponding predicted and observed DDI AUC_{last} ratios, DDI C_{max} ratios, as well as GMFE values for the updated and the original efavirenz PBPK model, respectively.

6.3.1 Alfentanil drug-dependent parameters

Table S30: Drug-dependent parameters of alfentanil (adopted from [226] and [210])

Parameter	Unit	Model	Literature	Reference	Description
MW	g/mol	416.52 (Lit)	416.52	[227]	Molecular weight
logP	Log Units	1.85 (Fit)	2.10, 2.20 (logD)	[228, 229]	Lipophilicity
Solubility (pH)	mg/ml	0.992 (6.5) (Lit)	0.992 (6.5)	[228]	Solubility
fu	%	10.0 (Lit)	8.6, 10.0, 12.0	[158, 230, 231]	Fraction unbound in plasma
pKa (base)	-	6.50 (Lit)	6.50	[229]	Acid dissociation constant
CL_{spec} (CYP3A4)	l/min	0.527 (Fit)	-	-	CYP3A4 clearance
GFR fraction	-	0.06 (Fit)	-	-	Fraction of filtered drug in the urine
Basolateral mucosa permeability	cm/min	5.42E-4 (Fit)	-	-	Basolateral mucosa permeability<br>P(interstitial → intracellular), P(intracellular → interstitial)
Intestinal permeability	cm/min	5.74E-4 (Fit)	-	-	Transcellular intestinal permeability
Partition coefficients	-	Diverse	Rogers and Rowland	[77, 78]	Cell to plasma partition coefficients
Cellular permeability	cm/min	6.88E-3 (Fit)	PK-Sim Standard	[79]	Permeability into the cellular space

-: not given, CL_{spec}: specific clearance, CYP: cytochrome P450, fit: optimized during parameter identification, lit: literature

6.3.2 Efavirenz-alfentanil clinical DDI studies

Table S31: Clinical studies investigating the efavirenz-alfentanil DDI

Efavirenz administration		Alfentanil administration		n	Healthy [%]	Females [%]	Age[a] [years]	Weight[a] [kg]	Height [cm]	Reference
**Dose [mg]**	**Route**	**Dose [mg]**	**Route**							
600	po (tab), qd (D1-D14)	3.05	po (sol), sd (D14)	12	100	50	22 (18-29)	71 (57-96)	-	Kharasch 2012 [98]
		1.06	iv, sd (D15)							

-: not given, D: day, iv: intravenous, po: oral, qd: once daily, sd: single dose, sol: solution tab: tablet

[a] mean (range)

6.3.3 Profiles

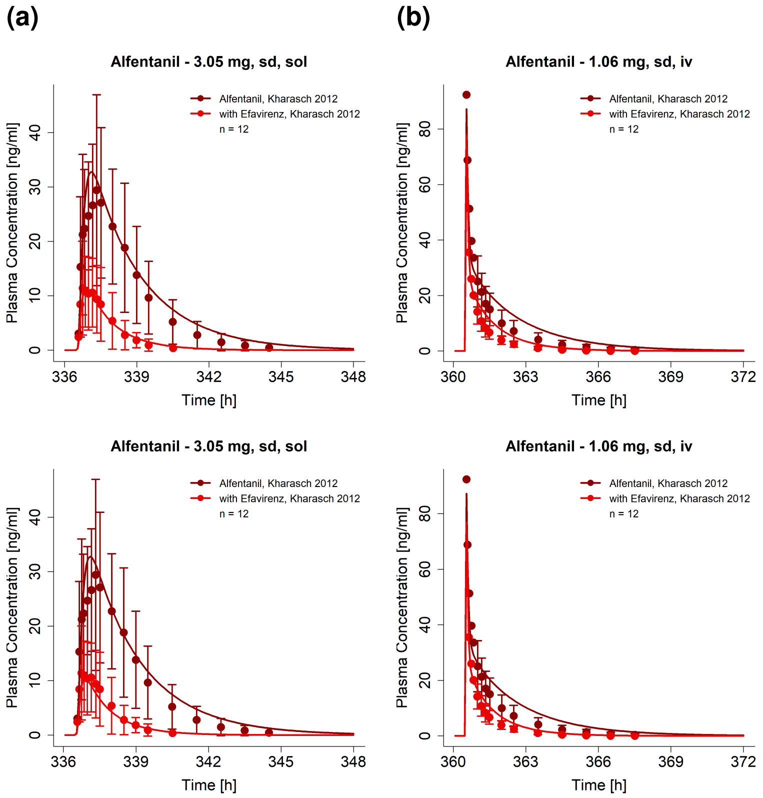

Figure S44: Predicted compared to observed alfentanil plasma concentration-time profiles (linear) before and during efavirenz co-administration, predicted with the updated efavirenz model (upper row) or the original efavirenz model (lower row). Observed data are shown as dots ± standard deviation; model predictions are shown as solid lines. Details on dosing regimens, study populations and literature references are listed in Table S31. iv: intravenous, sd: single dose, sol: solution

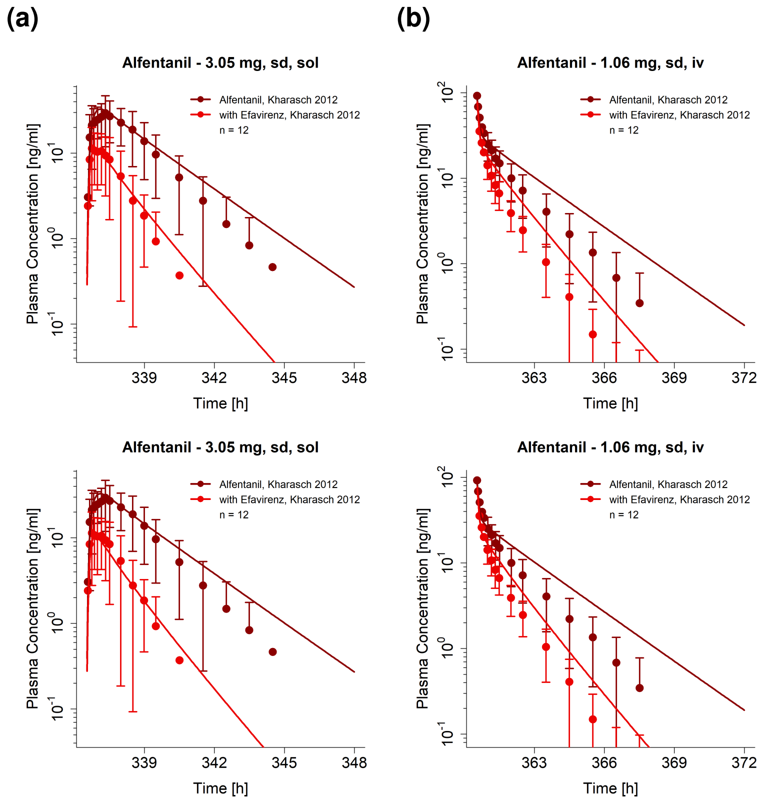

Figure S45: Predicted compared to observed alfentanil plasma concentration-time profiles (semi-logarithmic) before and during efavirenz co-administration, predicted with the updated efavirenz model (upper row) or the original efavirenz model (lower row). Observed data are shown as dots ± standard deviation; model predictions are shown as solid lines. Details on dosing regimens, study populations and literature references are listed in Table S31. iv: intravenous, sd: single dose, sol: solution

6.3.4 DDI AUC_{last} and C_{max} ratio goodness-of-fit plots

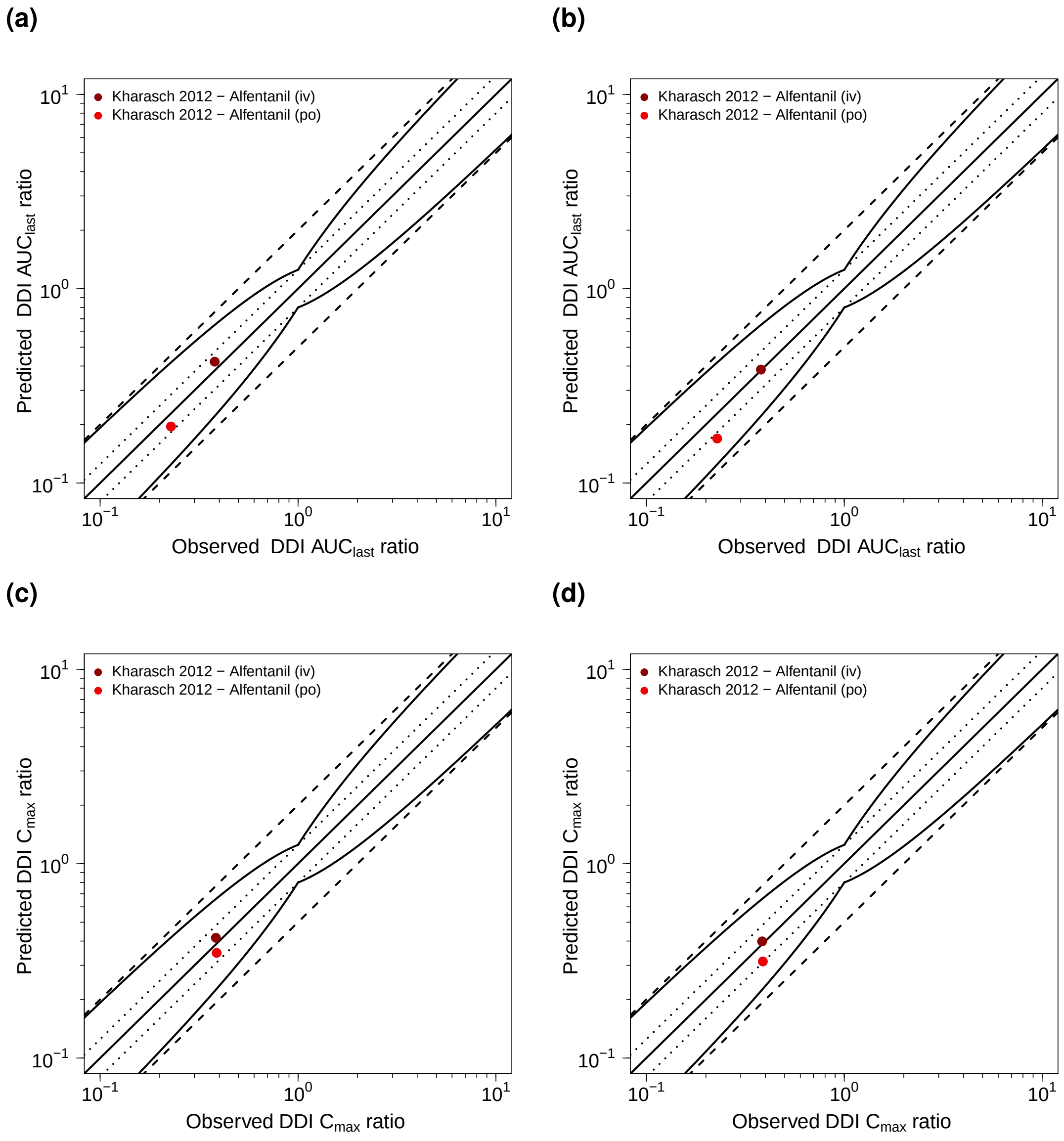

Figure S46: Predicted compared to observed efavirenz-alfentanil (a,b) DDI AUC_{last} and (c,d) DDI C_{max} ratios, predicted with the updated efavirenz PBPK model (left) or the original PBPK model (right). The straight solid line marks the line of identity, the curved solid lines show the prediction success limits proposed by Guest et al. allowing for 1.25-fold variability of the DDI ratio [109]. Dotted lines indicate 1.25-fold, dashed lines indicate 2-fold deviation. AUC_{last}: area under the plasma concentration-time curve from the time of drug administration to the last concentration measurement, C_{max}: maximum plasma concentration, DDI: drug-drug interaction, iv: intravenous, po: oral

6.3.5 Geometric mean fold error of predicted DDI AUC_{last} and C_{max} ratios

Table S32: Predicted and observed efavirenz-alfentanil DDI AUC_{last} and C_{max} ratios with geometric mean fold errors of the updated model

**Perpetrator**	**Victim**			**DDI AUC_{last} ratio**			**DDI C_{max} ratio**			
Efavirenz	Alfentanil	**Dose gap [h]**	**n**	**Pred**	**Obs**	**Pred/Obs**	**Pred**	**Obs**	**Pred/Obs**	**Reference**
600 mg, po, qd	3.05 mg, po, sd	-	12	0.20	0.23	0.89	0.36	0.39	0.93	Kharasch 2012 et al.[98]
600 mg, po, qd	1.06 mg, iv, sd	-	12	0.43	0.38	1.13	0.42	0.38	1.09	Kharasch 2012 et al.[98]
**mean GMFE (range)**						**1.13 (1.12-1.13)**			**1.09 (1.08-1.09)**	
						**2/2 with GMFE ≤ 2**			**2/2 with GMFE ≤ 2**	

-: not given, AUC_{last}: area under the plasma concentration-time curve from the time of drug administration to the last concentration measurement, C_{max}: maximum plasma concentration, DDI: drug-drug interaction, GMFE: geometric mean fold error, iv: intravenous, obs: observed, po: oral, pred: predicted, qd: once daily, sd: single dose

Table S33: Predicted and observed efavirenz-alfentanil DDI AUC_{last} and C_{max} ratios with geometric mean fold errors of the original model

**Perpetrator**	**Victim**			**DDI AUC_{last} ratio**			**DDI C_{max} ratio**			
Efavirenz	Alfentanil	**Dose gap [h]**	**n**	**Pred**	**Obs**	**Pred/Obs**	**Pred**	**Obs**	**Pred/Obs**	**Reference**
600 mg, po, qd	3.05 mg, po, sd	-	12	0.17	0.23	0.74	0.31	0.39	0.81	Kharasch 2012 [98]
600 mg, po, qd	1.06 mg, iv, sd	-	12	0.38	0.38	1.01	0.40	0.38	1.04	Kharasch 2012 [98]
**mean GMFE (range)**						**1.18 (1.01-1.35)**			**1.14 (1.04-1.24)**	
						**2/2 with GMFE ≤ 2**			**2/2 with GMFE ≤ 2**	

-: not given, AUC_{last}: area under the plasma concentration-time curve from the time of drug administration to the last concentration measurement, C_{max}: maximum plasma concentration, DDI: drug-drug interaction, GMFE: geometric mean fold error, iv: intravenous, obs: observed, po: oral, pred: predicted, qd: once daily, sd: single dose

6.4 Efavirenz-bupropion DDI

The efavirenz-bupropion DDI was modeled using a previously established whole-body PBPK model of bupropion [197]. Bupropion is a sensitive CYP2B6 substrate, with hydroxybupropion as the main metabolite. CYP2B6 metabolism is described in the model using Michaelis-Menten kinetics. The drug-dependent parameters of the bupropion model are reproduced in Table S21.

The efavirenz-bupropion DDI was modeled as induction with simultaneous competitive inhibition of bupropion CYP2B6 metabolism by efavirenz. Inhibition was described using K_i = 0.4 µmol/l from literature. The value was corrected for binding in the in vitro assay, as described in section 3.1. Induction of CYP2B6 by efavirenz was described using an EC_{50} = 0.23 µmol/l and E_{max} = 8.13. EC_{50} was taken from literature and corrected for $fu_{incubation}$, E_{max} was optimized during the efavirenz parameter identification.

Details on the modeled clinical DDI studies are given in Table S34. Model predictions of bupropion plasma concentration-time profiles before and during efavirenz co-administration, compared to observed data, are shown in Figures S47 (linear) and S48 (semi-logarithmic). The correlation of predicted to observed DDI AUC_{last} and C_{max} ratios is shown in Figure S49. Tables S35 and S36 list the corresponding predicted and observed DDI AUC_{last} ratios, DDI C_{max} ratios, as well as GMFE values for the updated and the original efavirenz PBPK model, respectively.

6.4.1 Efavirenz-bupropion clinical DDI studies

Table S34: Clinical studies investigating the efavirenz-bupropion DDI

Efavirenz administration		Bupropion administration		n	Healthy [%]	Females [%]	Age[a] [years]	Weight[a] [kg]	Height [cm]	Reference
**Dose [mg]**	**Route**	**Dose [mg]**	**Route**							
600	po (tab), qd (D1-D14)	150	po (tab), sd (D14)	13	100	23	39 (21-54)	86	-	Robertson 2008[b] [232]

-: not given, D: day, po: oral, qd: once daily, sd: single dose, tab: tablet

[a] mean (range)

[b] 56% intermediate metabolizers (subjects were tested for the diminished-function allele CYP2B6*6)

6.4.2 Profiles

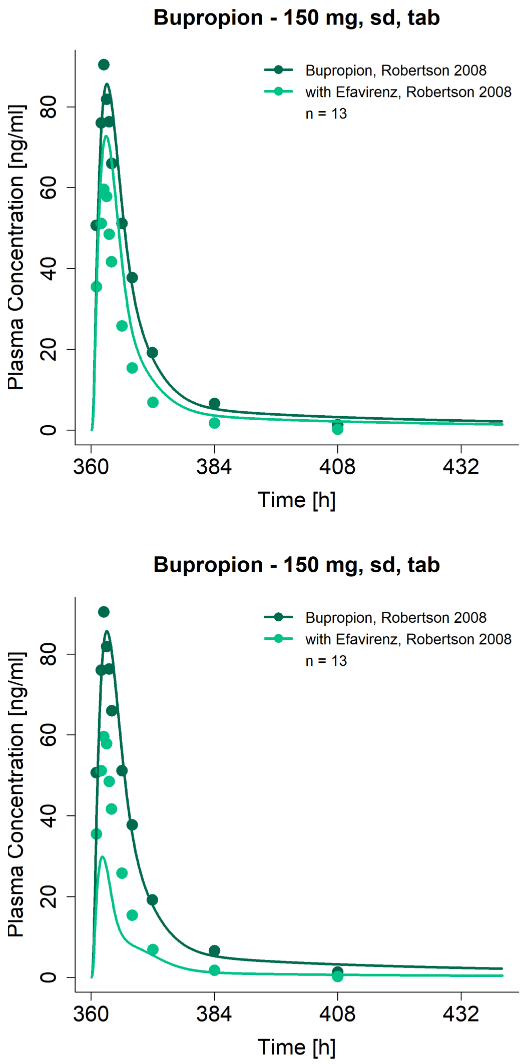

Figure S47: Predicted compared to observed bupropion plasma concentration-time profiles (linear) before and during efavirenz co-administration, predicted with the updated efavirenz model (upper row) or the original efavirenz model (lower row). Observed data are shown as dots $\pm$ standard deviation; model predictions are shown as solid lines. Details on dosing regimens, study population and literature reference are listed in Table S34. sd: single dose, tab: tablet

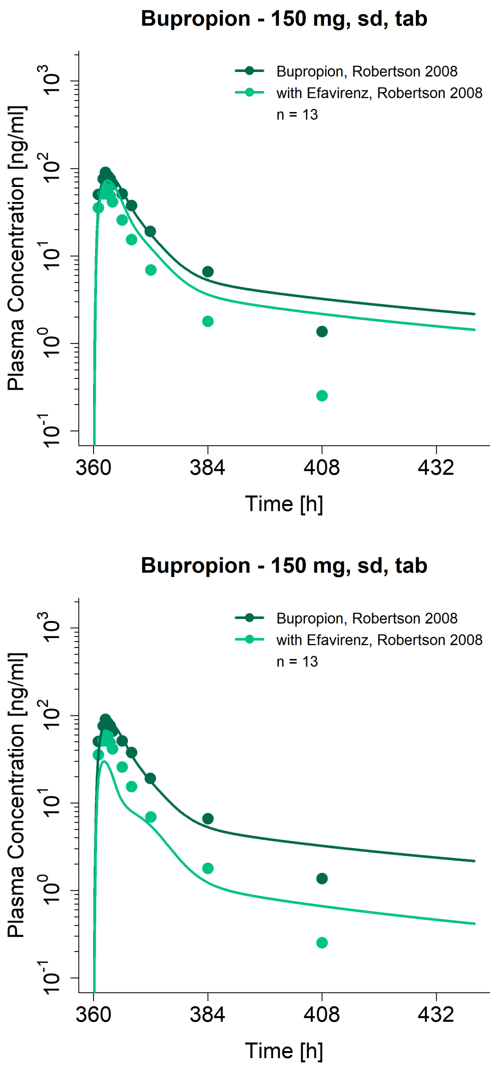

Figure S48: Predicted compared to observed bupropion plasma concentration-time profiles (semi-logarithmic) before and during efavirenz co-administration, predicted with the updated efavirenz model (upper row) or the original efavirenz model (lower row). Observed data are shown as dots ± standard deviation; model predictions are shown as solid lines. Details on dosing regimens, study population and literature reference are listed in Table S34. sd: single dose, tab: tablet

6.4.3 DDI AUC_{last} and C_{max} ratio goodness-of-fit plots

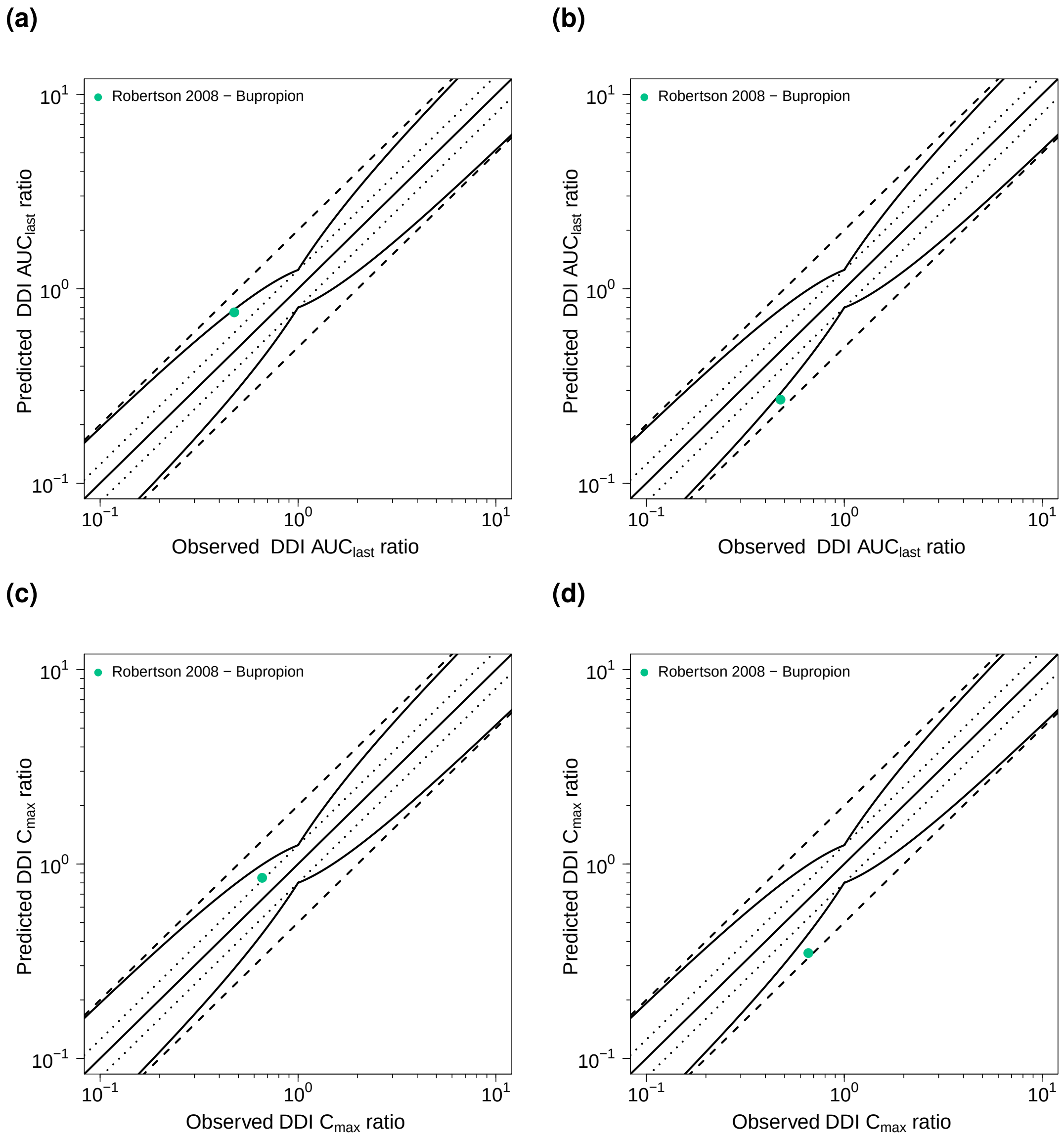

Figure S49: Predicted compared to observed efavirenz-bupropion (a,b) DDI AUC_{last} and (c,d) DDI C_{max} ratios, predicted with the updated efavirenz PBPK model (left) or the original PBPK model (right). The straight solid line marks the line of identity, the curved solid lines show the prediction success limits proposed by Guest et al. allowing for 1.25-fold variability of the DDI ratio [109]. Dotted lines indicate 1.25-fold, dashed lines indicate 2-fold deviation. AUC_{last}: area under the plasma concentration-time curve from the time of drug administration to the last concentration measurement, C_{max}: maximum plasma concentration, DDI: drug-drug interaction

6.4.4 Geometric mean fold error of predicted DDI AUC_{last} and C_{max} ratios

Table S35: Predicted and observed efavirenz-bupropion DDI AUC_{last} and C_{max} ratios with geometric mean fold errors of the updated model

**Perpetrator**	**Victim**			**DDI AUC_{last} ratio**			**DDI C_{max} ratio**			
Efavirenz	Bupropion	**Dose gap [h]**	**n**	**Pred**	**Obs**	**Pred/Obs**	**Pred**	**Obs**	**Pred/Obs**	**Reference**
600 mg, po, qd	150 mg, po, sd	0	13	0.76	0.48	1.59	0.85	0.66	1.29	Robertson 2008 [232]
**mean GMFE**						**1.59**			**1.29**	
						**1/1 with GMFE ≤ 2**			**1/1 with GMFE ≤ 2**	

AUC_{last}: area under the plasma concentration-time curve from the time of drug administration to the last concentration measurement, C_{max}: maximum plasma concentration, DDI: drug-drug interaction, GMFE: geometric mean fold error, obs: observed, po: oral, pred: predicted, qd: once daily, sd: single dose

Table S36: Predicted and observed efavirenz-bupropion DDI AUC_{last} and C_{max} ratios with geometric mean fold errors of the original model

**Perpetrator**	**Victim**			**DDI AUC_{last} ratio**			**DDI C_{max} ratio**			
Efavirenz	Bupropion	**Dose gap [h]**	**n**	**Pred**	**Obs**	**Pred/Obs**	**Pred**	**Obs**	**Pred/Obs**	**Reference**
600 mg, po, qd	150 mg, po, sd	0	13	0.27	0.48	0.57	0.35	0.66	0.53	Robertson 2008 [232]
**mean GMFE**						**1.77**			**1.89**	
						**1/1 with GMFE ≤ 2**			**1/1 with GMFE ≤ 2**	

AUC_{last}: area under the plasma concentration-time curve from the time of drug administration to the last concentration measurement, C_{max}: maximum plasma concentration, DDI: drug-drug interaction, GMFE: geometric mean fold error, obs: observed, po: oral, pred: predicted, qd: once daily, sd: single dose

6.5 Rifampin-efavirenz DDI

The rifampin-efavirenz DDI was modeled using a previously developed whole-body PBPK model of rifampin [210]. Rifampin induces the expression of CYP3A4 and CYP2B6. The drug-dependent parameters of the rifampin model are reproduced in Table S37.

The rifampin-efavirenz DDI was modeled as induction with simultaneous competitive inhibition of efavirenz CYP3A4 and CYP2B6 metabolism by rifampin. Parameters describing the CYP3A4 induction and inhibition were previously implemented and have been qualified in several different DDI predictions [210]. The K_i = 118.5 µmol/l describing the competitive CYP2B6 inhibition and E_{max} = 3.6 describing the CYP2B6 induction by rifampin were taken from literature. EC_{50} = 0.34 µmol/l was adopted from induction processes already implemented in the rifampin PBPK model (see Table S37), as induction of 3A4 and 2B6 by rifampin is both mediated via activation of the nuclear receptor PXR [210].

Details on the modeled clinical DDI studies are given in Table S38. Model predictions of efavirenz plasma concentration-time profiles before and during rifampin co-administration, compared to observed data, are shown in Figures S50 (linear) and S51 (semi-logarithmic). The correlation of predicted to observed DDI AUC_{last} and C_{max} ratios is shown in Figure S46. Tables S39 and S40 list the corresponding predicted and observed DDI AUC_{last} ratios, DDI C_{max} ratios, as well as GMFE values for the updated and the original efavirenz PBPK model, respectively.

6.5.1 Rifampin drug-dependent parameters

Table S37: Drug-dependent parameters of the rifampin PBPK model (adopted from [210])

Parameter	Unit	Model	Literature	Reference	Description
MW	g/mol	822.94 (Lit)	822.94	[233]	Molecular weight
logP	-	2.50 (Fit)	1.30, 2.70	[228, 233]	Lipophilicity
Solubility (pH)	g/l	2.8 (7.5) (Lit)	2.8 (7.5)	[234]	Solubility
fu	%	17.0 (Lit)	17.0	[235]	Fraction unbound in plasma
pKa (acid)	-	1.7 (Lit)	1.7	[236]	First acid dissociation constant
pKa (base)	-	7.9 (Lit)	7.9	[236]	Second acid dissociation constant
B/P ratio	-	0.89 (Calc)	0.90	[237]	Blood/plasma ratio
K_m (OATP1B1)	µmol/l	1.5 (Lit)	1.5	[238]	OATP1B1 Michaelis-Menten constant
k_{cat} (OATP1B1)	1/min	7.8 (Fit)	-	-	OATP1B1 transport rate constant
K_m (AADAC)	µmol/l	195.1 (Lit)	195.1	[239]	AADAC Michaelis-Menten constant
k_{cat} (AADAC)	1/min	9.87 (Fit)	-	-	AADAC catalytic rate constant
K_m (Pgp)	µmol/l	55.0 (Lit)	55.0	[240]	Pgp Michaelis-Menten constant
k_{cat} (Pgp)	1/min	0.61 (Fit)	-	-	Pgp transport rate constant
GFR fraction	-	1 (Asm)	-	-	Fraction of filtered drug in the urine
Induction EC_{50}	µmol/l	0.34 (Lit)	0.42 [a]	[66, 235]	Conc. for half-maximal induction
E_{max} (OATP1B1)	-	0.38 (Fit)	-	-	Maximum OATP1B1 induction effect
E_{max} (AADAC)	-	0.99 (Fit)	-	-	Maximum AADAC induction effect
E_{max} (Pgp)	-	2.5 (Lit)	2.5	[241]	Maximum Pgp induction effect
E_{max} (CYP3A4)	-	9.0 (Lit)	9.0	[235]	Maximum CYP3A4 induction effect
E_{max} (CYP2B6)	-	3.6 (Lit)	3.6	[242]	Maximum CYP2B6 induction effect
K_i (OATP1B1)	µmol/l	0.48 (Lit)	0.48	[243]	Concentration for half-maximal OATP1B1 inhibition
K_i (Pgp)	µmol/l	169.0 (Lit)	169.0	[244]	Concentration for half-maximal Pgp inhibition
K_i (CYP3A4)	µmol/l	18.5 (Lit)	18.5	[245]	Concentration for half-maximal CYP3A4 inhibition
K_i (CYP2B6)	µmol/l	118.5(Lit)	118.5	[246]	Concentration for half-maximal CYP2B6 inhibition
Intestinal permeability	cm/min	1.24E-5 (Fit)	3.84E-07	Calculated	Transcellular intestinal permeability
Partition coefficients	-	Diverse	Rodgers and Rowland	[77, 78]	Cell to plasma partition coefficients
Cellular permeability	cm/min	2.93E-5 (Calc)	PK-Sim Standard	[79]	Permeability into the cellular space

-: not given, AADAC: arylacetamide deacetylase, calc: calculated, CYP: cytochrome P450, GFR: glomerular filtration rate, OATP1B1: organic anion transporting polypeptide 1B1, Pgp: P-glycoprotein

[a] $fu_{incubation}$ = 0.80 was applied to in vitro literature value

6.5.2 Rifampin-efavirenz clinical DDI studies

Table S38: Clinical studies investigating the rifampin-efavirenz DDI

Rifampin administration		Efavirenz administration		n	Healthy [%]	Females [%]	Age[a] [years]	Weight[a] [kg]	Height [cm]	Reference
**Dose [mg]**	**Route**	**Dose [mg]**	**Route**							
600	po (tab), qd (D1-D7)	50	po (tab), sd (D7)	16	100	0	23.8 (20-35)	23.5 (20.6-27.5)[b]	-	Derungs2016[c] [89]
600	po (tab), qd (D1-D11)	600	po (-), sd (D11)	20	100	50	27.5 (19-44)	72.9 (57-88)	-	Cho 2016 [92]
600	po (-), qd (D1-D8)	600	po (-), qd (D1-D8)	11	100	55	42.6	76.9	-	Kwara 2011[d] [99]

-: not given, D: day, po: oral, qd: once daily, sd: single dose, tab: tablet

[a] mean (range)

[b] Body mass index

[c] CYP2B6 status: 9 intermediate metabolizers, 0 poor metabolizers; subjects were tested for the diminished-function allele CYP2B6*6

[d] CYP2B6 status: 2 extensive metabolizers, 6 intermediate metabolizers, 3 poor metabolizers; subjects were tested for the diminished-function allele CYP2B6*6 and CYP2B6*16

6.5.3 Profiles

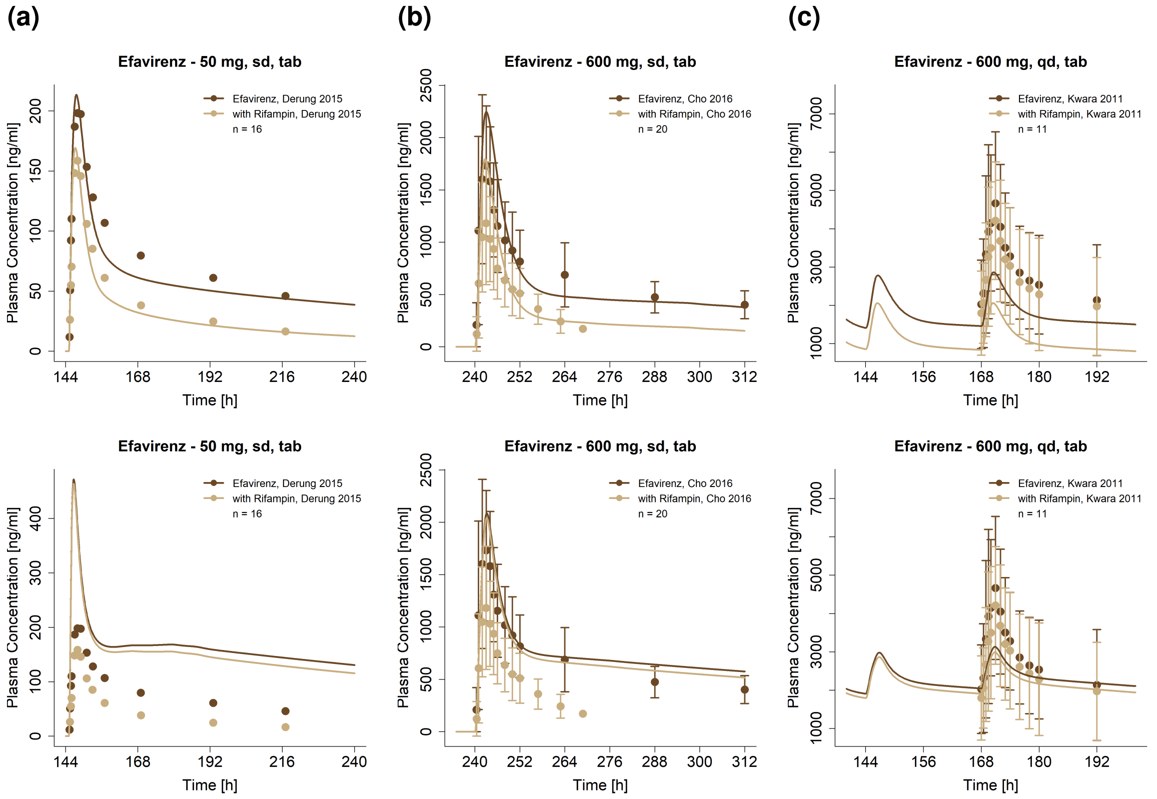

Figure S50: Predicted compared to observed efavirenz plasma concentration-time profiles (linear) before and during rifampin co-administration, predicted with the updated efavirenz model (upper row) or the original efavirenz model (lower row). Observed data are shown as dots ± standard deviation; model predictions are shown as solid lines. Details on dosing regimens, study populations and literature references are listed in Table S38. qd: once daily, sd: single dose, sol: solution, tab: tablet

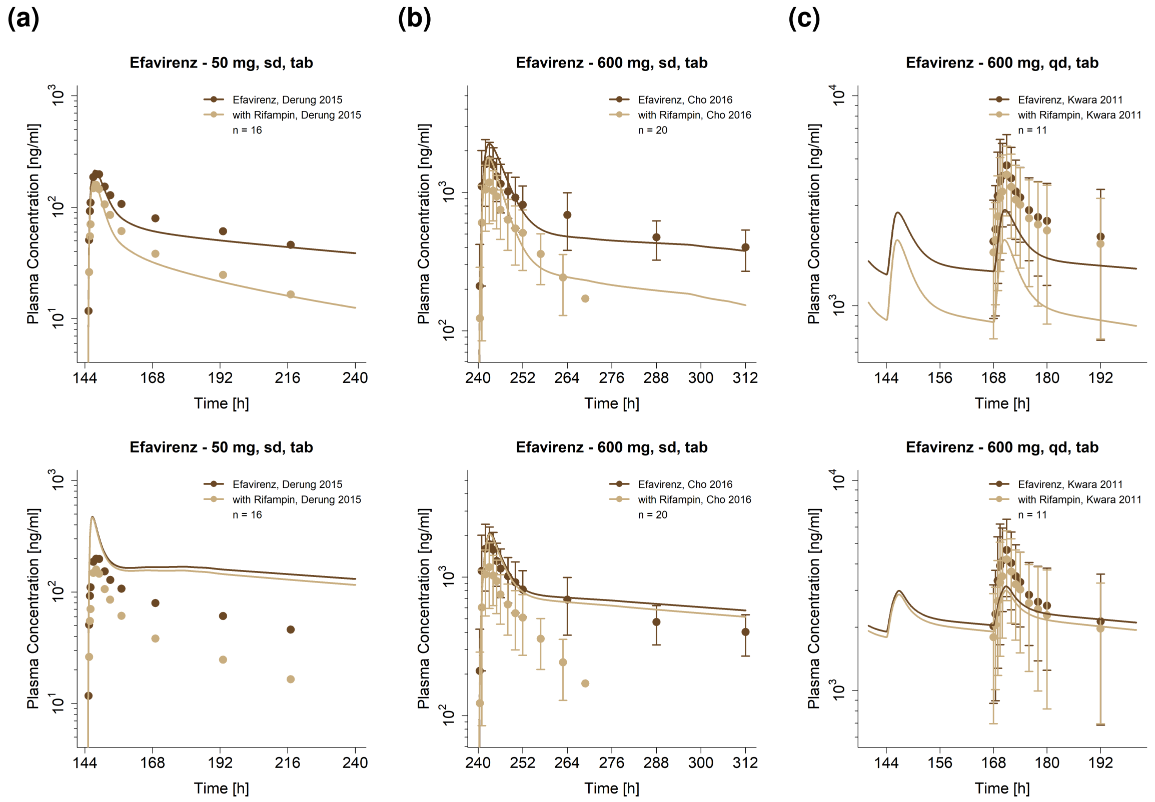

Figure S51: Predicted compared to observed efavirenz plasma concentration-time profiles (semi-logarithmic) before and during rifampin co-administration, predicted with the updated efavirenz model (upper row) or the original efavirenz model (lower row). Observed data are shown as dots ± standard deviation; model predictions are shown as solid lines. Details on dosing regimens, study populations and literature references are listed in Table S38. qd: once daily, sd: single dose, sol: solution, tab: tablet

6.5.4 DDI AUC_{last} and C_{max} ratio goodness-of-fit plots

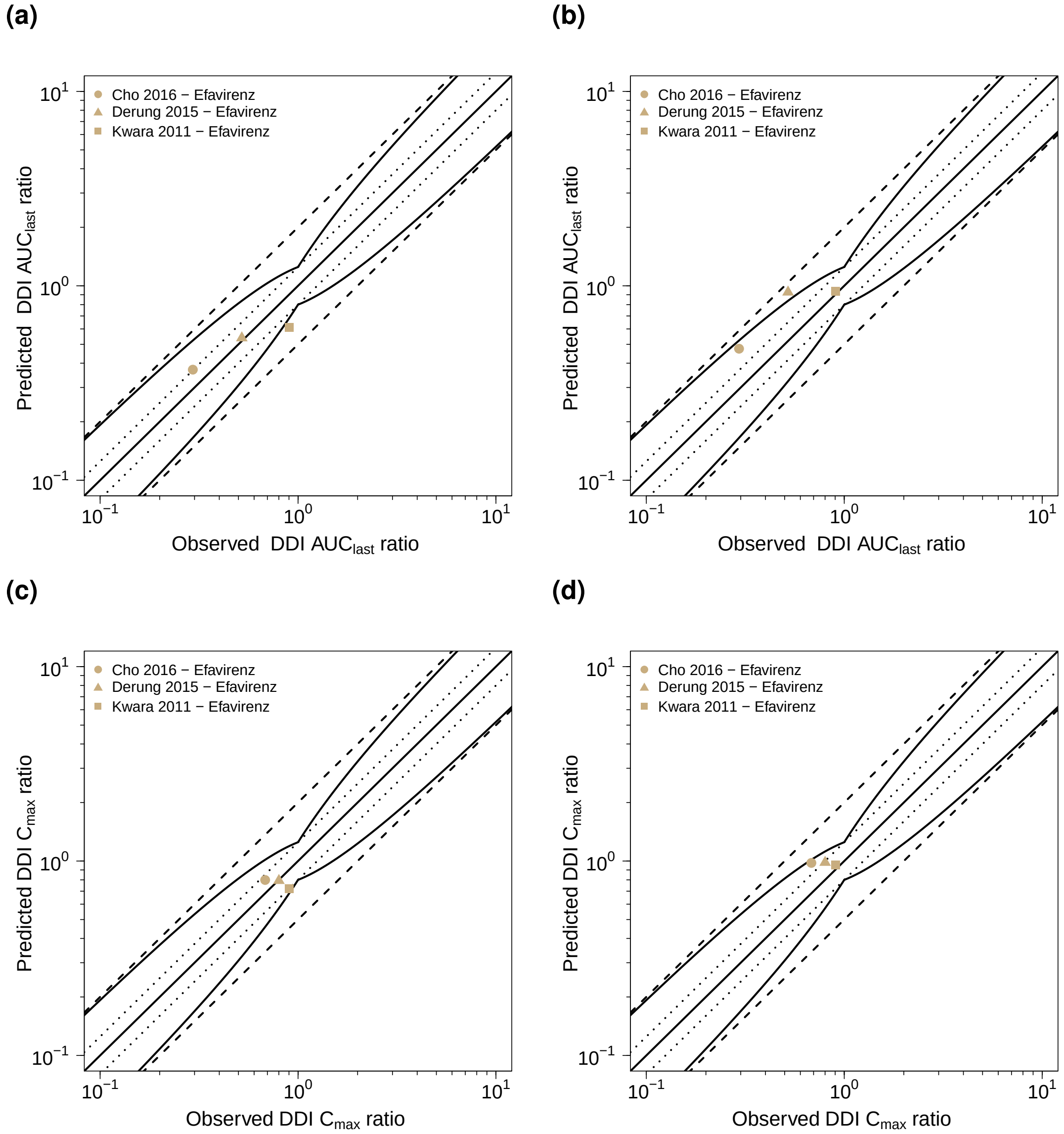

Figure S52: Predicted compared to observed rifampin-efavirenz (a,b) DDI AUC_{last} and (c,d) DDI C_{max} ratios, predicted with the updated efavirenz PBPK model (left) or the original PBPK model (right). The straight solid line marks the line of identity, the curved solid lines show the prediction success limits proposed by Guest et al. allowing for 1.25-fold variability of the DDI ratio [109]. Dotted lines indicate 1.25-fold, dashed lines indicate 2-fold deviation. AUC_{last}: area under the plasma concentration-time curve from the time of drug administration to the last concentration measurement, C_{max}: maximum plasma concentration, DDI: drug-drug interaction

6.5.5 Geometric mean fold error of predicted DDI AUC_{last} and C_{max} ratios

Table S39: Predicted and observed rifampin-efavirenz DDI AUC_{last} and C_{max} ratios with geometric mean fold errors of the updated model

**Perpetrator**	**Victim**			**DDI AUC_{last} ratio**			**DDI C_{max} ratio**			
Rifampin	Efavirenz	**Dose gap [h]**	**n**	**Pred**	**Obs**	**Pred/Obs**	**Pred**	**Obs**	**Pred/Obs**	**Reference**
600 mg, po, qd	50 mg, po, sd	-	16	0.54	0.52	1.04	0.79	0.80	0.99	Derungs 2015 [89]
600 mg, po, qd	600 mg, po, sd	0	20	0.37	0.29	1.26	0.80	0.68	1.17	Cho 2016 [92]
600 mg, po, qd	600 mg, po, qd	-	11	0.61	0.90	0.68	0.72	0.90	0.80	Kwara 2011 [99]
**mean GMFE (range)**						**1.26 (1.04-1.48)**			**1.15 (1.01-1.26)**	
						**3/3 with GMFE ≤ 2**			**3/3 with GMFE ≤ 2**	

-: not given, AUC_{last}: area under the plasma concentration-time curve from the time of drug administration to the last concentration measurement, C_{max}: maximum plasma concentration, DDI: drug-drug interaction, GMFE: geometric mean fold error, obs: observed, po: oral, pred: predicted, qd: once daily, sd: single dose

Table S40: Predicted and observed rifampin-efavirenz DDI AUC_{last} and C_{max} ratios with geometric mean fold errors of the original model

**Perpetrator**	**Victim**			**DDI AUC_{last} ratio**			**DDI C_{max} ratio**			
Rifampin	Efavirenz	**Dose gap [h]**	**n**	**Pred**	**Obs**	**Pred/Obs**	**Pred**	**Obs**	**Pred/Obs**	**Reference**
600 mg, po, qd	50 mg, po, sd	-	16	0.93	0.52	1.78	0.98	0.80	1.23	Derungs 2015 [89]
600 mg, po, qd	600 mg, po, sd	0	20	0.47	0.29	1.61	0.98	0.68	1.43	Cho 2016 [92]
600 mg, po, qd	600 mg, po, sd	-	11	0.94	0.90	1.04	0.95	0.90	1.06	Kwara 2011 [99]
**mean GMFE (range)**						**1.48 (1.04-1.78)**			**1.24 (1.06-1.43)**	
						**3/3 with GMFE ≤ 2**			**3/3 with GMFE ≤ 2**	

-: not given, AUC_{last}: area under the plasma concentration-time curve from the time of drug administration to the last concentration measurement, C_{max}: maximum plasma concentration, DDI: drug-drug interaction, GMFE: geometric mean fold error, obs: observed, po: oral, pred: predicted, qd: once daily, sd: single dose

6.6 Efavirenz-voriconazole DDI

The efavirenz-voriconazole DDI was modeled using a previously established whole-body PBPK model of voriconazole [247]. The metabolism of the CYP2C19 and CYP3A4 substrate voriconazole is described in the model using Michaelis-Menten kinetics. The drug-dependent parameters of the voriconazole model are reproduced in Table S41.

To adequately describe the voriconazole plasma concentration-time profiles, the CYP2C19 k_{cat} was adjusted (k_{cat} = 0.20 1/min) to match the control group of the modeled DDI study, assuming variability of the CYP2C19 expression in the small DDI study population.

The efavirenz-voriconazole DDI was modeled as induction with simultaneous competitive inhibition of voriconazole CYP3A4 metabolism by efavirenz and as competitive inhibition of efavirenz CYP2B6 metabolism by voriconazole. Inhibition of CYP3A4 by efavirenz was described using K_i = 9.67 µmol/l from literature, corrected for binding in the in vitro assay, as described in Section 3. The induction was described using EC_{50} = 0.23 µmol/l (taken from literature and corrected for binding in the in vitro assay). Inhibition of CYP2B6 by voriconazole was described using a K_i = 0.3 µmol/l from literature, corrected for binding in the in vitro assay.

Details on the modeled clinical DDI studies are given in Table S42. Model predictions of voriconazole and efavirenz plasma concentration-time profiles before and during co-administration, compared to observed data, are shown in Figure S53 (linear) and S54 (semi-logarithmic). The correlation of predicted to observed DDI AUC_{last} and C_{max} ratios is shown in Figure S55. Tables S43 and S44 list the corresponding predicted and observed DDI AUC_{last} ratios, DDI C_{max} ratios, as well as GMFE values for the updated and the original efavirenz PBPK model, respectively.

6.6.1 Voriconazole drug-dependent parameters

Table S41: Drug-dependent parameters of the voriconazole PBPK model (adopted from [247])

Parameter	Unit	Model	Literature	Reference	Description
MW	g/mol	349.3 (Lit)	349.3	[248]	Molecular weight
logP	log Units	1.80 (Lit)	1.65, 1.75, 1.80, 2.56	[248–252]	Lipophilicity
Solubility (pH)	mg/ml	3.2 (1.0), 2.7 (1.2), 0.1 (7.0)	3.2 (1.0), 2.7 (1.2), 0.1 (7.0)	[248, 250, 253, 254]	Solubility
fu	%	42.0 (Lit)	42.0	[250–252, 255]	Fraction unbound in plasma
pKa (base)	-	1.60 (Lit)	1.60, 1.76, 2.27	[248, 250, 251, 253, 256]	Acid dissociation constant
K_m (CYP3A4)	µmol/l	15.0 (Lit)	11.0, 15.0, 16.0, 235.0, 834.7	[250, 252, 257, 258]	CYP3A4 Michaelis-Menten constant
k_{cat} (CYP3A4)	1/min	2.12 (Fit)	0.05, 0.10, 0.14, 0.31, 32.2	[250, 252, 257, 258]	CYP3A4 catalytic rate constant
K_m (CYP2C19)	µmol/l	3.5 (Lit)	3.5, 9.3, 14.0	[250, 252, 257, 258]	CYP2C19 Michaelis-Menten constant
k_{cat} (CYP2C19)	1/min	1.19 (Lit)	0.22, 0.39, 1.19, 40.0	[250, 252, 257, 258]	CYP2C19 catalytic rate constant
GFR fraction	-	1 (Asm)	-	-	Fraction of filtered drug in the urine
K_i (CYP3A4)	µmol/l	9.33 (Lit)	9.33	[247]	Concentration for half-maximal inhibition
k_{inact} (CYP3A4)	1/min	0.015 (Fit)	0.04	[247]	Maximum inactivation rate constant
Intestinal permeability	cm/s	1.62E-2 (Fit)	1.68E-3	[252]	Transcellular intestinal permeability
Partition coefficients	-	Diverse	Poulin and Theil	[259–262]	Cell to plasma partition coefficients
Cellular permeability	cm/min	2.58E-3 (Calc)	PK-Sim Standard	[79]	Permeability into the cellular space
Tablet Weibull time	min	30.0 (Fit)	-	-	Dissolution time (50% dissolved)
Tablet Weibull shape	-	1.29 (Fit)	-	-	Dissolution profile shape

-: not given, asm: assumption, calc: calculated, CYP: cytochrome P450, fit: optimized during parameter optimization, GFR: glomerular filtration rate, lit: literature

6.6.2 Efavirenz-voriconazole clinical DDI studies

Table S42: Clinical studies investigating the efavirenz-voriconazole DDI

**Efavirenz administration**		**Voriconazole administration**		**n**	**Healthy [%]**	**Females [%]**	**Age[a] [years]**	**Weight[a] [kg]**	**Height [cm]**	**Reference**
**Dose [mg]**	**Route**	**Dose [mg]**	**Route**							
400	po (cap), qd (D1-D19)	200[b]	po (tab), bid (D11-D19)	27	100	0	34 (20-48)	79 (59-92)	-	Liu 2008 [91]

-: not given, bid: twice daily, cap: capsule, D: day, po: oral, qd: once daily, tab: tablet

[a] mean (range)

[b] Two 400 mg loading doses (bid) on day 1

6.6.3 Profiles

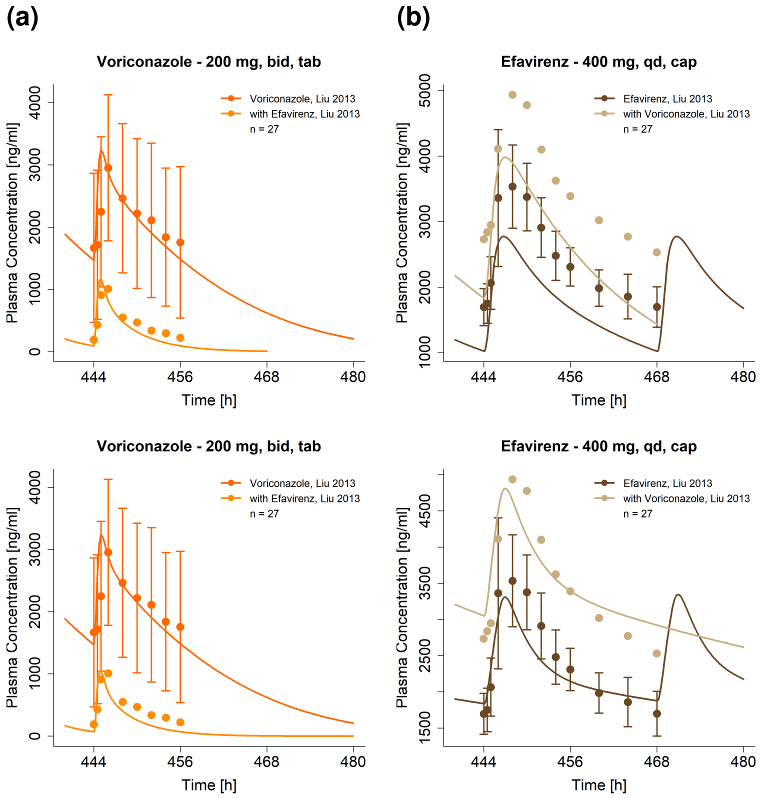

Figure S53: Predicted compared to observed voriconazole and efavirenz plasma concentration-time profiles (linear) before and during co-administration, predicted with the updated efavirenz model (upper row) or the original efavirenz model (lower row). Observed data are shown as dots ± standard deviation; model predictions are shown as solid lines. Details on dosing regimens, study population and literature reference are listed in Table S42. bid: twice daily, cap: capsule, qd: once daily, tab: tablet

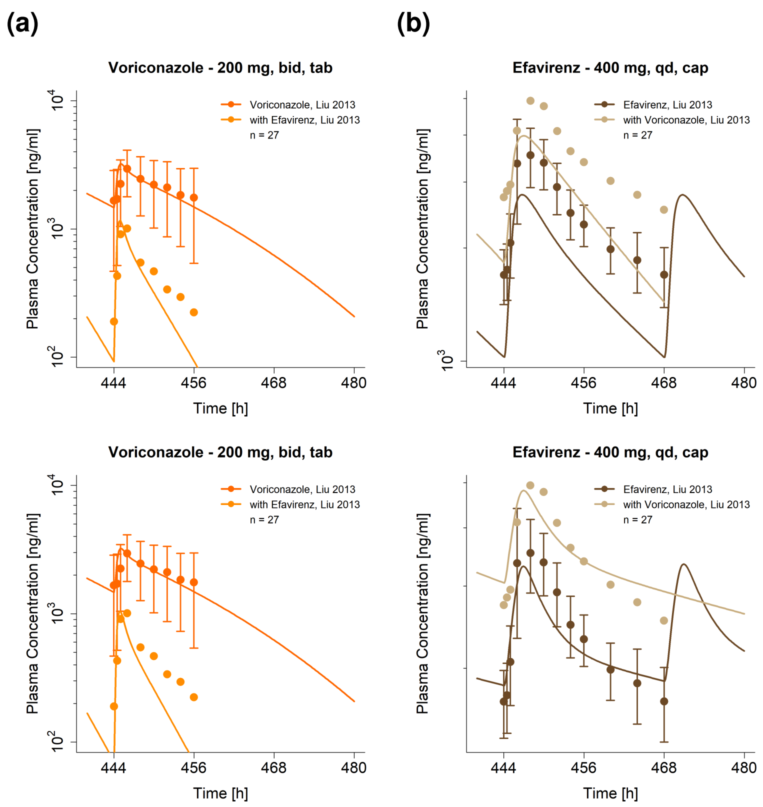

Figure S54: Predicted compared to observed efavirenz and voriconazole plasma concentration-time profiles (semi-logarithmic) before and during efavirenz and voriconazole co-administration, predicted with the updated efavirenz model (upper row) or the original efavirenz model (lower row). Observed data are shown as dots ± standard deviation; model predictions are shown as solid lines. Details on dosing regimens, study population and literature reference are listed in Table S42. bid: twice daily, cap: capsule, qd: once daily, tab: tablet

6.6.4 DDI AUC_{last} and C_{max} ratio goodness-of-fit plots

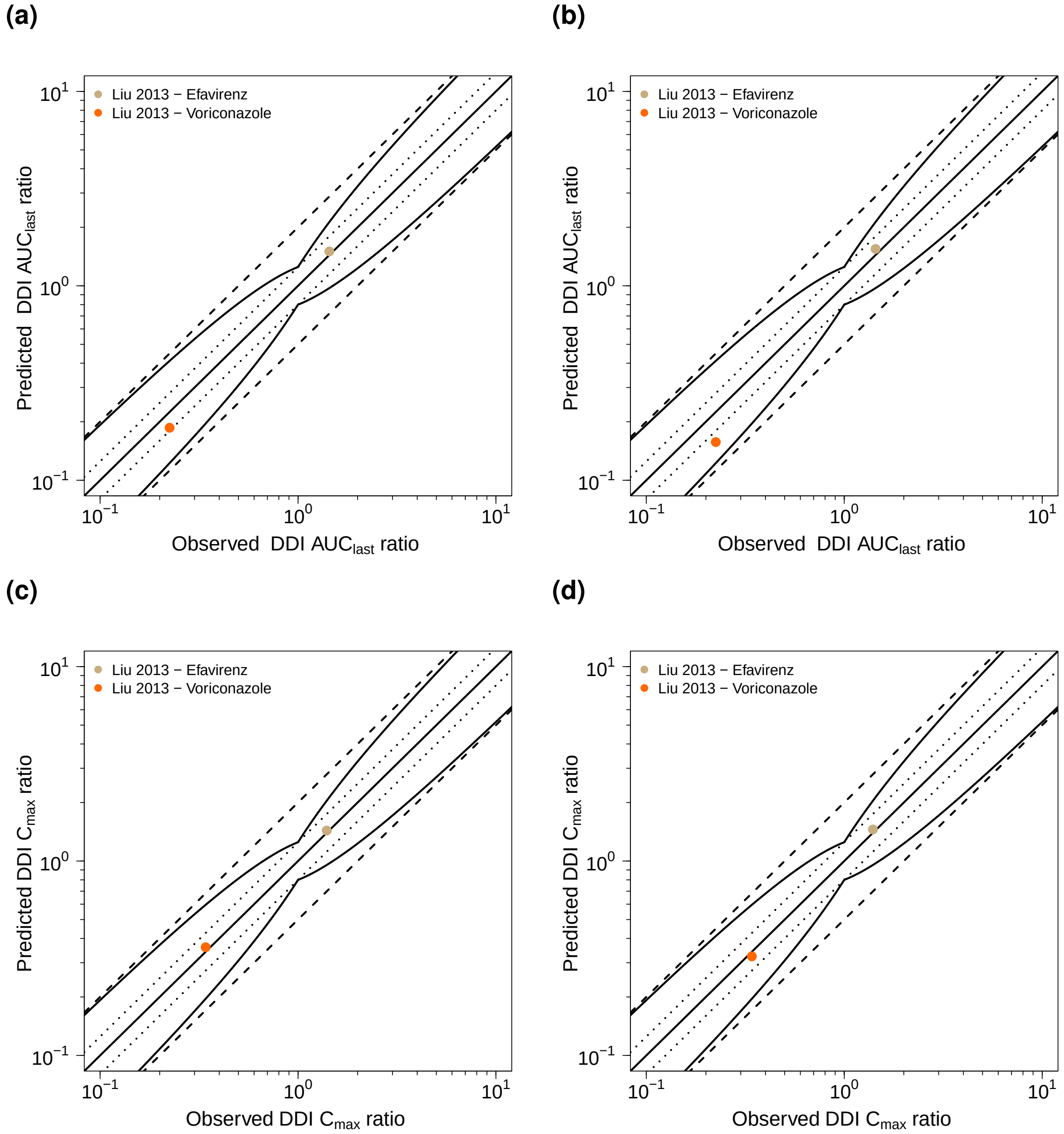

Figure S55: Predicted compared to observed efavirenz-voriconazole (a,b) DDI AUC_{last} and (c,d) DDI C_{max} ratios predicted with the updated efavirenz PBPK model (left) or the original PBPK model (right). The straight solid line marks the line of identity, the curved solid lines show the prediction success limits proposed by Guest et al. allowing for 1.25-fold variability of the DDI ratio [109]. Dotted lines indicate 1.25-fold, dashed lines indicate 2-fold deviation. AUC_{last}: area under the plasma concentration-time curve from the time of drug administration to the last concentration measurement, C_{max}: maximum plasma concentration, DDI: drug-drug interaction

6.6.5 Geometric mean fold error of predicted DDI AUC_{last} and C_{max} ratios

Table S43: Predicted and observed efavirenz-voriconazole DDI AUC_{last} and C_{max} ratios with geometric mean fold errors of the updated model

				DDI AUC_{last} ratio			DDI C_{max} ratio			
**Perpetrator**	**Victim**	**Dose gap [h]**	**n**	**Pred**	**Obs**	**Pred/Obs**	**Pred**	**Obs**	**Pred/Obs**	**Reference**
Voriconazole	*Efavirenz*									
200 mg, po, bid	400 mg, po, qd	0	27	1.53	1.44	1.06	1.46	1.40	1.04	Liu 2013 [91]
Efavirenz	*Voriconazole*									
200 mg, po, bid	400 mg, po, qd	0	27	0.19	0.22	0.87	0.37	0.34	1.09	Liu 2013 [91]
**mean GMFE (range)**						**1.11 (1.06-1.15)**			**1.06 (1.04-1.09)**	
						**2/2 with GMFE ≤ 2**			**2/2 with GMFE ≤ 2**	

AUC_{last}: area under the plasma concentration-time curve from the time of drug administration to the last concentration measurement, bid: twice daily, C_{max}: maximum plasma concentration, DDI: drug-drug interaction, GMFE: geometric mean fold error, obs: observed, po: oral, pred: predicted, qd: once daily

Table S44: Predicted and observed efavirenz-voriconazole DDI AUC_{last} and C_{max} ratios with geometric mean fold errors of the original model

				DDI AUC_{last} ratio			DDI C_{max} ratio			
**Perpetrator**	**Victim**	**Dose gap [h]**	**n**	**Pred**	**Obs**	**Pred/Obs**	**Pred**	**Obs**	**Pred/Obs**	**Reference**
Voriconazole	Efavirenz									
200 mg, po, bid	400 mg, po, qd	0	27	1.55	1.44	1.08	1.45	1.40	1.04	Liu 2013 [91]
Efavirenz	Voriconazole									
200 mg, po, bid	400 mg, po, qd	0	27	0.16	0.22	0.70	0.32	0.34	0.95	Liu 2013 [91]
**mean GMFE (range)**						**1.25 (1.08-1.43)**			**1.05 (1.04-1.06)**	
						**2/2 with GMFE ≤ 2**			**2/2 with GMFE ≤ 2**	

AUC_{last}: area under the plasma concentration-time curve from the time of drug administration to the last concentration measurement, bid: twice daily, C_{max}: maximum plasma concentration, DDI: drug-drug interaction, GMFE: geometric mean fold error, obs: observed, po: oral, pred: predicted, qd: once daily

7 System-dependent parameters

Details on the expression of metabolizing enzymes, transport proteins and protein binding partners implemented to model the pharmacokinetics of carbamazepine, carbamazepine-10,11-epoxide, efavirenz, alfentanil, alprazolam, bupropion, erythromycin, rifampin, simvastatin and voriconazole are summarized in Table S45. As enterohepatic circulation is active under physiological conditions, the parameter EHC continuous fraction was set to 1 in all individuals.

Table S45: System-dependent parameters

	**Reference concentration**				**Half-life**	
**Enzyme/ Transporter Binding partner**	**Mean[a] [µmol/l]**	**Relative expression[b]**	**Localization**	**Direction**	**Liver [h]**	**Intestine [h]**
11β-HSD	1.00[c] [263]	Array [265]	Intracellular	-	36	23
AADAC	1.00[c] [263]	RT-PCR [266]	Intracellular	-	36	23
CYP1A2	1.80 [267]	RT-PCR [268]	Intracellular	-	39	23
CYP2A6	2.72 [267]	RT-PCR [268]	Intracellular	-	26	23
CYP3A4	4.32 [267]	RT-PCR [268]	Intracellular	-	36 [269]	23 [139]
CYP3A5	0.04 [267]	RT-PCR [268]	Intracellular	-	36	23
CYP2B6	1.56 [267]	RT-PCR [268]	Intracellular	-	32	23
CYP2C8	2.56 [267]	RT-PCR [268]	Intracellular	-	23	23
CYP2C19	0.76 [267]	RT-PCR [268]	Intracellular	-	26	23
EPHX1	1.00[c] [263]	RT-PCR [266]	Intracellular	-	36	23
PON3	1.00[c] [263]	Array [265]	Intracellular	-	36	23
UGT1A1	1.00[c] [263]	RT-PCR [266]	Intracellular	-	36	23
UGT1A3	1.00[c] [263]	RT-PCR [266]	Intracellular	-	36	23
UGT1A4	2.32 [53]	liver only [209]	Intracellular	-	36	-
UGT2B7	2.78 [270]	EST [271]	Intracellular	-	36	23
BCRP	1.00[c] [263]	RT-PCR[d] [272]	Apical	Efflux	36	23
OATP1B1	1.00[c] [263]	RT-PCR [272]	Basolateral	Influx	36	-
Pgp	1.41 [210]	RT-PCR[e] [272]	Apical	Efflux	36	23
GABRG2	1.30 [209]	brain only [209]	Extracellular membrane	-	-	-
NAT1	1.00[c] [263]	EST [271]	Extracellular membrane	-	36	23

-: not given, 11β-HSD1: 11β-hydroxysteroid dehydrogenase 1, AADAC: arylacetamide deacetylase, array: genome expression arrays from ArrayExpress, BCRP: breast cancer resistance protein, CYP: cytochrome P450, EPHX1: epoxidehydroxylase 1, EST: expressed sequence tags expression profile, GABRG2: gamma-aminobutyric acid receptor subunit gamma 2, NAT1: norepinephrine transporter 1, OATP1B1: organic anion transporting polypeptide 1B1, Pgp: P-glycoprotein, PON3: paraoxonase 3, RT-PCR: reverse transcription-polymerase chain reaction profile, UGT: UDP-glucuronosyltransferase

[a] µmol/lol protein/l in the tissue of highest expression

[b] in the different organs (PK-Sim® expression database profile)

[c] if no information was available, the mean reference concentration was set to 1.00 µmol/l and the catalytic rate constant (kcat) was optimized [263]

[d] with the relative expression in the blood cells set to 0.3046 [264]

[e] with the relative expression in the intestinal mucosa increased by factor 3.57 [210]

List of Figures

S1 Metabolic pathways of carbamazepine 10
S2 Predicted compared to observed carbamazepine-10,11-epoxide plasma concentration-time profiles (linear) 16
S3 Predicted compared to observed carbamazepine-10,11-epoxide plasma concentration-time profiles (semi-logarithmic) 17
S4 Predicted compared to observed carbamazepine and carbamazepine-10,11-epoxide plasma (and saliva) concentration-time profiles (linear) 18
S5 Predicted compared to observed carbamazepine and carbamazepine-10,11-epoxide plasma (and saliva) concentration-time profiles (semi-logarithmic) 24
S6 Predicted compared to observed carbamazepine and carbamazepine-10,11-epoxide fraction excreted unchanged in urine profiles (linear) 30
S7 Predicted compared to observed carbamazepine-10,11-epoxide plasma concentrations 31
S8 Predicted compared to observed carbamazepine and carbamazepine-10,11-epoxide concentrations 32
S9 Predicted compared to observed fractions excreted unchanged in urine 33
S10 Predicted compared to observed carbamazepine-10,11-epoxide AUC_{last} and C_{max} values 37
S11 Predicted compared to observed carbamazepine and carbamazepine-10,11-epoxide AUC_{last} and C_{max} values 38
S12 Carbamazepine-10,11-epoxide PBPK model sensitivity analysis (carbamazepine-10,11-epoxide administration) 43
S13 Carbamazepine parent-metabolite PBPK model sensitivity analysis 44

S14 Metabolic pathways of efavirenz 45
S15 Predicted compared to observed efavirenz plasma concentration-time profiles (linear) 51
S16 Predicted compared to observed efavirenz plasma concentration-time profiles (semi-logarithmic) 55
S17 Predicted compared to observed efavirenz plasma concentrations 59
S18 Predicted compared to observed efavirenz AUC_{last} and C_{max} values 61
S19 Updated efavirenz PBPK model sensitivity analysis 63
S20 Original efavirenz PBPK model sensitivity analysis 64

S21 Predicted compared to observed efavirenz plasma concentration-time profiles (linear) for different CYP2B6 genotypes 67
S22 Predicted compared to observed efavirenz plasma concentration-time profiles (semi-logarithmic) for different CYP2B6 genotypes 68
S23 Predicted compared to observed efavirenz DGI AUC_{last} and DGI C_{max} ratios 69

S24 Carbamazepine drug-drug interaction network 71
S25 Erythromycin-carbamazepine DDI - linear 75
S26 Erythromycin-carbamazepine DDI - semi-logarithmic 76
S27 Predicted compared to observed erythromycin-carbamazepine DDI AUC_{last} and DDI C_{max} ratios 77
S28 Carbamazepine-alprazolam DDI -linear 82
S29 Carbamazepine-alprazolam DDI - semi-logarithmic 82
S30 Predicted compared to observed carbamazepine-alprazolam DDI AUC_{last} and DDI C_{max} ratios 83

S31 Carbamazepine-simvstatin DDI - linear 90
S32 Carbamazepine-simvstatin DDI - semi-logarithmic 90
S33 Predicted compared to observed carbamazepine-simvastatin DDI AUC_{last} and DDI C_{max} ratios 91
S34 Carbamazepine-bupropion DDI - linear 98
S35 Carbamazepine-bupropion DDI - semi-logarithmic 99
S36 Predicted compared to observed carbamazepine-bupropion DDI AUC_{last} and DDI C_{max} ratios 100
S37 Efavirenz-carbamazepine DDI - linear 104
S38 Efavirenz-carbamazepine DDI - semi-logarithmic 105
S39 Predicted compared to observed efavirenz-carbamazepine DDI AUC_{last} and DDI C_{max} ratios 106

S40 Efavirenz drug-drug interaction network 108
S41 Efavirenz-midazolam DDI - linear 112
S42 Efavirenz-midazolam DDI - semi-logarithmic 113
S43 Predicted compared to observed efavirenz-midazolam DDI AUC_{last} and DDI C_{max} ratios 114
S44 Efavirenz-alfentanil DDI - linear 119
S45 Efavirenz-alfentanil DDI - semi-logarithmic 120
S46 Predicted compared to observed efavirenz-alfentanil DDI AUC_{last} and DDI C_{max} ratios 121
S47 Efavirenz-bupropion DDI - linear 125
S48 Efavirenz-bupropion DDI - semi-logarithmic 126
S49 Predicted compared to observed efavirenz-bupropion DDI AUC_{last} and DDI C_{max} ratios 127
S50 Rifampin-efavirenz DDI - linear 132
S51 Rifampin-efavirenz DDI - semi-logarithmic 133
S52 Predicted compared to observed rifampin-efavirenz DDI AUC_{last} and DDI C_{max} ratios 134
S53 Voriconazole-efavirenz DDI - linear 139
S54 Voriconazole-efavirenz DDI - semi-logarithmic 140
S55 Predicted compared to observed efavirenz-voriconazole DDI AUC_{last} and DDI C_{max} ratios 141

List of Tables

S1 Clinical studies used for the development of the carbamazepine parent-metabolite PBPK model . . . 11
S2 Drug-dependent parameters of the carbamazepine and carbamazepine-10,11-epoxide PBPK model . . . 14
S3 Mean relative deviation values of predicted carbamazepine and carbamazepine-10,11-epoxide plasma and saliva concentrations . . . 34
S4 Predicted and observed AUC_{last} and C_{max} values with geometric mean fold errors of carbamazepine and carbamazepine-10,11-epoxide . . . 39

S5 Clinical studies used for the update of the efavirenz PBPK model . . . 47
S6 Drug-dependent parameters of the efavirenz PBPK model . . . 49
S7 Mean relative deviation values of predicted efavirenz plasma concentrations of the updated model in comparison to the original model . . . 60
S8 Predicted and observed efavirenz AUC_{last} and C_{max} values with geometric mean fold errors of the updated model in comparison to the original model . . . 62

S9 Clinical studies used for the establishment of the efavirenz DGI parameters . . . 66
S10 Predicted and observed DGI AUC_{last} and C_{max} ratios with geometric mean fold errors of the updated model . . . 70
S11 Predicted and observed DGI AUC_{last} and C_{max} ratios with geometric mean fold errors of the original model . . . 70

S12 Drug-dependent parameters of the erythromycin PBPK model (adopted from [110]) 73
S13 Clinical studies investigating the erythromycin-carbamazepine DDI . . . 74
S14 Predicted and observed erythromycin-carbamazepine DDI AUC_{last} and C_{max} ratios with geometric mean fold errors . . . 78
S15 Drug-dependent parameters of the alprazolam PBPK model (adopted from [137]) 80
S16 Clinical studies investigating the carbamazepine-alprazolam DDI . . . 81
S17 Predicted and observed carbamazepine-alprazolam DDI AUC_{last} and C_{max} ratios with geometric mean fold errors . . . 84
S18 Drug-dependent parameters of the simvastatin PBPK model (adopted from [150]) 86
S19 Clinical studies investigating the carbamazepine-simvastatin DDI . . . 89
S20 Predicted and observed carbamazepine-simvastatin DDI AUC_{last} and C_{max} ratios with geometric mean fold errors . . . 92
S21 Drug-dependent parameters of the bupropion PBPK model (adopted from [197]) 94
S22 Clinical studies investigating the carbamazepine-bupropion DDI . . . 97
S23 Predicted and observed carbamazepine-bupropion DDI AUC_{last} and C_{max} ratios with geometric mean fold errors . . . 101
S24 Clinical studies investigating the efavirenz-carbamazepine DDI . . . 103
S25 Predicted and observed efavirenz-carbamazepine DDI AUC_{last} and C_{max} ratios with geometric mean fold errors . . . 107

S26 Drug-dependent parameters of the midazolam PBPK model (adopted from [209] and [210]) . . . 110
S27 Clinical studies investigating the efavirenz-midazolam DDI . . . 111
S28 Predicted and observed efavirenz-midazolam DDI AUC_{last} and C_{max} ratios with geometric mean fold errors of the updated model . . . 115
S29 Predicted and observed efavirenz-midazolam DDI AUC_{last} and C_{max} ratios with geometric mean fold errors of the original model . . . 115
S30 Drug-dependent parameters of alfentanil (adopted from [226] and [210]) . . . 117

S31 Clinical studies investigating the efavirenz-alfentanil DDI 118
S32 Predicted and observed efavirenz-alfentanil DDI AUC_{last} and C_{max} ratios with geometric mean fold errors of the updated model 122
S33 Predicted and observed efavirenz-alfentanil DDI AUC_{last} and C_{max} ratios with geometric mean fold errors of the original model 122
S34 Clinical studies investigating the efavirenz-bupropion DDI 124
S35 Predicted and observed efavirenz-bupropion DDI AUC_{last} and C_{max} ratios with geometric mean fold errors of the updated model 128
S36 Predicted and observed efavirenz-bupropion DDI AUC_{last} and C_{max} ratios with geometric mean fold errors of the original model 128
S37 Drug-dependent parameters of the rifampin PBPK model (adopted from [210]) . 130
S38 Clinical studies investigating the rifampin-efavirenz DDI 131
S39 Predicted and observed rifampin-efavirenz DDI AUC_{last} and C_{max} ratios with geometric mean fold errors of the updated model 135
S40 Predicted and observed rifampin-efavirenz DDI AUC_{last} and C_{max} ratios with geometric mean fold errors of the original model 135
S41 Drug-dependent parameters of the voriconazole PBPK model (adopted from [247])137
S42 Clinical studies investigating the efavirenz-voriconazole DDI 138
S43 Predicted and observed efavirenz-voriconazole DDI AUC_{last} and C_{max} ratios with geometric mean fold errors of the updated model 142
S44 Predicted and observed efavirenz-voriconazole DDI AUC_{last} and C_{max} ratios with geometric mean fold errors of the original model 142

S45 System-dependent parameters 143

Abbreviations

**11β-HSD1**	11β-hydroxysteroid dehydrogenase 1
**AADAC**	Arylacetamide deacetylase
**ADME**	Absorption, distribution, metabolism and excretion
**AUC**	Area under the concentration-time curve
**AUC_{last}**	AUC values calculated from the time of drug administration to the time of the last concentration measurement
**asm**	Assumption
**BCRP**	Breast cancer resistance protein
**bid**	Twice daily
**CBZ**	Carbamazepine
**CBZE**	Carbamazepine-10,11-epoxide
**calc**	Calculated
**cap**	Capsule
**CAR**	Constitutive androstane receptor
**CL_{hep}**	Hepatic clearance
**C_{mic}**	Microsomal protein concentration
**CL_{spec}**	Secific clearance
**C_{max}**	Maximum plasma concentration
**CYP**	Cytochrome P450
**D**	Day
**DDI**	Drug-drug interaction
**DGI**	Drug-gene interaction
**EC50**	Concentration for half maximal induction in vivo
**EBUP**	Erythrohydrobupropion
**EHC**	Enterohepatic circulation
**EM**	Extensive metabolizer
**E_{max}**	Maximal induction effect in vivo
**EPHX1**	Epoxide hydroxylase 1
**EST**	Expressed sequence tag
**FaHIF**	Fasted human intestinal fluid

**fit**	Optimized during parameter identification
**fu**	Fraction unbound in plasma
**$fu_{incubation}$**	Fraction unbound in the incubation
**GABRG2**	Gamma-aminobutyric acid receptor subunit gamma 2
**GFR**	Glomerular filtration rate
**GMFE**	Geometric mean fold error
**IC_{50}**	Half maximal inhibitory concentration
**IM**	Intermediate metabolizer
**IR**	Immediate release
**iv**	Intravenous
**k_{cat}**	Transport or catalytic rate constant
**k_{deg}**	Degradation rate constant
**$k_{deg, app}$**	Degradation rate constant in the presence of a mechanism based inhibitor
**K_i**	Dissociation constant of the inhibitor-transporter/ -enzyme complex
**K_{inact}**	Maximum inactivation rate
**K_M**	Michaelis-Menten constant
**$K_{M,app}$**	Michaelis-Menten constant in the presence of inhibitor
**logP**	Lipophilicity
**lit**	Literature
**MRD**	Mean relative deviation
**MW**	Molecular weight
**NAT1**	Norepinephrine transporter 1
**OATP**	Organic-anion-transporting polypeptide
**obs**	Observed
**OSP**	Open Systems Pharmacology
**Pgp**	P-glycoprotein
**PBPK**	Physiologically based pharmacokinetic
**pKa**	Acid dissociation constant
**PM**	Poor metabolizer
**po**	Oral
**PON3**	Paraoxonase 3
**pred**	Predicted

**PXR**	Pregnane X receptor
**qid**	Four times daily
**qd**	Once daily
R_{syn}	Rate of transporter or enzyme synthesis
$R_{syn,app}$	Rate of transporter or enzyme synthesis in the presence of inducer
**RT-PCR**	Reverse transcription-polymerase chain reaction
**sd**	Single dose
**sol**	Solution
**susp**	Suspension
**tab**	Tablet
**TBUP**	Threohydrobupropion
**tid**	Three times daily
**UGT**	UDP-glucuronosyltransferase
**v**	Reaction velocity
v_{max}	Maximum reaction velocity
**XR**	Extended release

Bibliography

[1] Open Systems Pharmacology Suite Community. PK-Sim® Ontogeny Database Documentation, Version 7.3, 2018. URL https://github.com/Open-Systems-Pharmacology/OSPSuite.Documentation/blob/master/PK-Sim%20Ontogeny%20Database%20Version%207.3.pdf. accessed: 14 Dec 2020.

[2] Open Systems Pharmacology Suite Community. Open Systems Pharmacology Suite Manual, 2018. URL https://docs.open-systems-pharmacology.org/. accessed: 14 Dec 2020.

[3] Novartis. Tegretol ® label, 2009. URL https://www.accessdata.fda.gov/drugsatfda_docs/label/2009/016608s101,018281s048lbl.pdf. accessed: 14 Dec 2020.

[4] M. Eichelbaum, T. Tomson, G. Tybring, and L. Bertilsson. Carbamazepine metabolism in man. Induction and pharmacogenetic aspects. *Clinical pharmacokinetics*, 10(1):80–90, 1985.

[5] T. Tomson, G. Tybring, and L. Bertilsson. Single-dose kinetics and metabolism of carbamazepine-10,11-epoxide. *Clinical Pharmacology and Therapeutics*, 33(1):58–65, jan 1983.

[6] B. M. Kerr, K. E. Thummel, C. J. Wurden, S. M. Klein, D. L. Kroetz, F. J. Gonzalez, and R. H. Levy. Human liver carbamazepine metabolism. Role of CYP3A4 and CYP2C8 in 10,11-epoxide formation. *Biochemical pharmacology*, 47(11):1969–79, jun 1994.

[7] R. E. Pearce, G. R. Vakkalagadda, and J. Steven Leeder. Pathways of carbamazepine bioactivation in vitro I. Characterization of human cytochromes P450 responsible for the formation of 2- and 3-hydroxylated metabolites. *Drug Metabolism and Disposition*, 30 (11):1170–1179, 2002.

[8] J. W. Faigle and K. F. Feldmann. *Pharmacokinetic data of carbamazepine and its major metabolites in man*. Springer Berlin Heidelberg, Berlin, Heidelberg, 1975. ISBN 978-3-642-85923-6.

[9] B. Terhaag, K. Richter, and H. Diettrich. Concentration behavior of carbamazepine in bile and plasma of man. *International journal of clinical pharmacology and biopharmacy*, 16 (12):607–9, 1978.

[10] U.S. Food and Drug Administration. Drug development and drug interactions, 2016. URL https://www.fda.gov/drugs/drug-interactions-labeling/drug-development-and-drug-interactions. accessed: 14 Dec 2020.

[11] B. M. Kerr, A. E. Rettie, A. C. Eddy, P. Loiseau, M. Guyot, A. J. Wilensky, and R. H. Levy. Inhibition of human liver microsomal epoxide hydrolase by valproate and valpromide: in vitro/in vivo correlation. *Clinical pharmacology and therapeutics*, 46(1):82–93, jul 1989.

[12] T. B. Vree, T. J. Janssen, Y. A. Hekster, E. F. Termond, A. C. van de Dries, and W. J. Wijnands. Clinical pharmacokinetics of carbamazepine and its epoxy and hydroxy metabolites in humans after an overdose. *Therapeutic drug monitoring*, 8(3):297–304, 1986.

[13] N. R. Kitteringham, C. Davis, N. Howard, M. Pirmohamed, and B. K. Park. Interindividual and interspecies variation in hepatic microsomal epoxide hydrolase activity: Studies with cis-stilbene oxide, carbamazepine 10,11- epoxide and naphthalene. *Journal of Pharmacology and Experimental Therapeutics*, 278(3):1018–1027, 1996.

[14] S. R. Faucette, T. C. Zhang, R. Moore, T. Sueyoshi, C. J. Omiecinski, E. L. LeCluyse, M. Negishi, and H. Wang. Relative activation of human pregnane X receptor versus constitutive androstane receptor defines distinct classes of CYP2B6 and CYP3A4 inducers. *Journal of Pharmacology and Experimental Therapeutics*, 320(1):72–80, 2007.

[15] R. H. Levy, W. H. Pitlick, A. S. Troupin, J. R. Green, and J. M. Neal. Pharmacokinetics of carbamazepine in normal man. *Clinical Pharmacology & Therapeutics*, 17(6):657–668, jun 1975.

[16] A. McLean, S. Browne, Y. Zhang, E. Slaughter, C. Halstenson, and R. Couch. The influence of food on the bioavailability of a twice-daily controlled release carbamazepine formulation. *Journal of Clinical Pharmacology*, 41(2):183–186, 2001.

[17] M. Sumi, N. Watari, O. Umezawa, and N. Kaneniwa. Pharmacokinetic study of carbamazepine and its epoxide metabolite in humans. *Journal of Pharmacobio-Dynamics*, 10(11):652–661, 1987.

[18] F. Pisani, M. Caputo, A. Fazio, G. Oteri, M. Russo, E. Spina, E. Perucca, and L. Bertilsson. Interaction of carbamazepine-10,11-epoxide, an active metabolite of carbamazepine, with valproate: A pharmacokinetic study. *Epilepsia*, 31(3):339–342, 1990.

[19] F. Pisani, A. Fazio, C. Artesi, G. Oteri, E. Spina, T. Tomson, and E. Perucca. Impairment of carbamazepine-10, 11-epoxide elimination by valnoctamide, a valpromide isomer, in healthy subjects. *British Journal of Clinical Pharmacology*, 34(1):85–87, 1992.

[20] A. Gérardin, J. P. Dubois, J. Moppert, and L. Geller. Absolute bioavailability of carbamazepine after oral administration of a 2% syrup. *Epilepsia*, 31(3):334–338, 1990.

[21] M. D. Rawlins, P. Collste, L. Bertilsson, and L. Palmér. Distribution and elimination kinetics of carbamazepine in man. *European journal of clinical pharmacology*, 8(2):91–6, feb 1975.

[22] J. A. Wada, A. S. Troupin, P. Friel, R. Remick, K. Leal, and J. Pearmain. Pharmacokinetic comparison of tablet and suspension dosage forms of carbamazepine. *Epilepsia*, 19(3): 251–5, 1978.

[23] A. P. Gérardin, F. V. Abadie, J. A. Campestrini, and W. Theobald. Pharmacokinetics of carbamazepine in normal humans after single and repeated oral doses. *Journal of Pharmacokinetics and Biopharmaceutics*, 4(6):521–535, 1976.

[24] S. K. Bedada, N. R. Yellu, and P. Neerati. Effect of resveratrol treatment on the pharmacokinetics of diclofenac in healthy human volunteers. *Phytotherapy Research*, 30(3): 397–401, 2016.

[25] S. K. Bedada, R. Appani, and P. K. Boga. Effect of piperine on the metabolism and pharmacokinetics of carbamazepine in healthy volunteers. *Drug research*, 67(1):46–51, jan 2017.

[26] M. Eichelbaum, K. Ekbom, L. Bertilsson, V. A. Ringberger, and A. Rane. Plasma kinetics of carbamazepine and its epoxide metabolite in man after single and multiple doses. *European Journal of Clinical Pharmacology*, 8(5):337–341, 1975.

[27] K. A. Kim, O. O. Sae, P. W. Park, and J. Y. Park. Effect of probenecid on the pharmacokinetics of carbamazepine in healthy subjects. *European Journal of Clinical Pharmacology*, 61(4):275–280, 2005.

[28] M. C. Meyer, A. B. Straughn, E. J. Jarvi, G. C. Wood, F. R. Pelsor, and V. P. Shah. The bioinequivalence of carbamazepine tablets with a history of clinical failures. *Pharmaceutical research*, 9(12):1612–6, 1992.

[29] M. C. Meyer, A. B. Straughn, R. M. Mhatre, V. P. Shah, R. L. Williams, and L. J. Lesko. The relative bioavailability and in vivo-in vitro correlations for four marketed carbamazepine tablets. *Pharmaceutical research*, 15(11):1787–91, 1998.

[30] A. Shahzadi, I. Javed, B. Aslam, F. Muhammad, M. R. Asi, M. Y. Ashraf, and Zia-Ur-Rahman. Therapeutic effects of ciprofloxacin on the pharmacokinetics of carbamazepine in healthy adult male volunteers. *Pakistan Journal of Pharmaceutical Sciences*, 24(1): 63–68, 2011.

[31] B. Saint-Salvi, D. Tremblay, A. Surjus, and M. A. Lefebvre. A study of the interaction of roxithromycin with theophylline and carbamazepine. *The Journal of antimicrobial chemotherapy*, 20 Suppl B:121–9, 1987.

[32] N. Barzaghi, G. Gatti, F. Crema, M. Monteleone, C. Amione, L. Leone, and E. Perucca. Inhibition by erythromycin of the conversion of carbamazepine to its active 10,11-epoxide metabolite. *British journal of clinical pharmacology*, 24(6):836–8, dec 1987.

[33] G. Bianchetti, P. Padovani, J. P. Thénot, J. F. Thiercelin, P. L. Morselli, J. P. Thenot, J. F. Thiercelin, and P. L. Morselli. Pharmacokinetic interactions of progabide with other antiepileptic drugs. *Epilepsia*, 28(1):68–73, 1987.

[34] I. Kovacević, J. Parojcić, I. Homsek, M. Tubić-Grozdanis, and P. Langguth. Justification of biowaiver for carbamazepine, a low soluble high permeable compound, in solid dosage forms based on IVIVC and gastrointestinal simulation. *Molecular pharmaceutics*, 6(1): 40–7, 2009.

[35] P. L. Morselli, M. Gerna, D. de Maio, G. Zanda, F. Viani, and S. Garattini. *Pharmacokinetic studies on carbamazepine in volunteers and in epileptic patients*. Springer Berlin Heidelberg, Berlin, Heidelberg, 1975. ISBN 978-3-642-85923-6. URL http://link.springer.com/10.1007/978-3-642-85921-2.

[36] S. Pynnönen. The Pharmacokinetics of Carbamazepine in Plasma and Saliva of Man. *Acta Pharmacologica et Toxicologica*, 41(5):465–471, 1977.

[37] R. E. Strandjord and S. I. Johannessen. *Clinical Pharmacology of Anti-Epileptic Drugs*. Springer Berlin Heidelberg, Berlin, Heidelberg, 1975. ISBN 978-3-642-85923-6. URL http://link.springer.com/10.1007/978-3-642-85921-2.

[38] Y. Y. Wong, T. M. Ludden, and R. D. Bell. Effect of erythromycin on carbamazepine kinetics. *Clinical pharmacology and therapeutics*, 33(4):460–4, 1983.

[39] M. J. Dalton, J. R. Powell, and J. A. Messenheimer. The influence of cimetidine on single-dose carbamazepine pharmacokinetics. *Epilepsia*, 26(2):127–130, 1985.

[40] J. Michael J. Dalton, J. Robert Powell, and John A. Messenheimer. *Drug Intelligence and Clinical Pharmacy*.

[41] L. M. Cotter, M. J. Eadie, W. D. Hooper, C. M. Lander, G. A. Smith, and J. H. Tyrer. The pharmacokinetics of carbamazepine. *European Journal of Clinical Pharmacology*, 12(6): 451–456, 1977.

[42] A. H. Burstein, R. L. Horton, T. Dunn, R. M. Alfaro, S. C. Piscitelli, and W. Theodore. Lack of effect of St John's Wort on carbamazepine pharmacokinetics in healthy volunteers. *Clinical Pharmacology and Therapeutics*, 68(6):605–612, 2000.

[43] S. E. Møller, F. Larsen, A. Z. Khan, and P. E. Rolan. Lack of effect of citalopram on the steady-state pharmacokinetics of carbamazepine in healthy male subjects. *Journal of Clinical Psychopharmacology*, 21(5):493–499, 2001.

[44] P. Ji, B. Damle, J. Xie, S. E. Unger, D. M. Grasela, and S. Kaul. Pharmacokinetic interaction between efavirenz and carbamazepine after multiple-dose administration in healthy subjects. *Journal of Clinical Pharmacology*, 48(8):948–956, 2008.

[45] M. V. Miles and M. B. Tennison. Erythromycin effects on multiple-dose carbamazepine kinetics. *Therapeutic Drug Monitoring*, 11(1):47–52, 1989.

[46] I. Bernus, R. G. Dickinson, W. D. Hooper, and M. J. Eadie. Early stage autoinduction of carbamazepine metabolism in humans. *European journal of clinical pharmacology*, 47 (4):355–60, 1994.

[47] E. Graf. *Bioäquivalenz; Qualitätsbewertung wirkstoffgleicher Fertigarzneimittel. Hsg. von H. Blume und E. Mutschler unter Mitarb. von G. Wendt, G. Stenzhorn, M. Siewert und M. Schäfer-Korting.* Govi-Verlag, 1990. URL http://doi.wiley.com/10.1002/pauz.19900190516.

[48] D. Licht, M. Zholkovsky, R. Kaplan, M. Friedman, A. Yacobi, Y. Golander, D. Moros, and B. Levitt. European patent specification. Sustained release carbamazepine formulation. EP 1044 681 B1, 2005.

[49] R. Kshirsagar, G. Shinde, and A. Kandikurwar. United States patent application publication. Extended release pharmaceutical compositions containing carbamazepine, Pub. No.: US 2014/0302138A1. URL https://patents.google.com/patent/US20140331942A1/en.

[50] R. E. Stevens, T. Limsakun, G. Evans, and D. H. Mason. Controlled, multidose, pharmacokinetic evaluation of two extended-release carbamazepine formulations (Carbatrol and Tegretol-XR). *Journal of pharmaceutical sciences*, 87(12):1531–4, dec 1998.

[51] M. Gande, R. Gondalia, M. Kothapalli, N. M. Velishala, and V. Koppuri. United States patent application publication - carbamazepine extended release dosage form, Pub. No.: US 2009/01696.19 A1. 2009.

[52] A. G. Staines, M. W. Coughtrie, and B. Burchell. N-glucuronidation of carbamazepine in human tissues is mediated by UGT2B7. *Journal of Pharmacology and Experimental Therapeutics*, 311(3):1131–1137, 2004.

[53] B. Achour, M. R. Russell, J. Barber, and A. Rostami-Hodjegan. Simultaneous quantification of the abundance of several cytochrome P450 and uridine 5-diphospho-glucuronosyltransferase enzymes in human liver microsomes using multiplexed targeted proteomics. *Drug Metabolism and Disposition*, 42(4):500–510, 2014.

[54] Drugbank. Carbamazepine, 2018. URL https://www.drugbank.ca/drugs/DB00564. accessed: 14 Dec 2020.

[55] R. P. Austin, P. Barton, S. L. Cockroft, M. C. Wenlock, and R. J. Riley. The influence of nonspecific microsomal binding on apparent intrinsic clearance, and its prediction from physicochemical properties. *Drug Metabolism and Disposition*, 30(12):1497–1503, 2002.

[56] A. Avdeef. *Absorption and Drug Development*. John Wiley & Sons, Inc., Hoboken, NJ, USA, 2012. ISBN 9781118286067.

[57] P. Annaert, J. Brouwers, A. Bijnens, F. Lammert, J. Tack, and P. Augustijns. Ex vivo permeability experiments in excised rat intestinal tissue and in vitro solubility measurements in aspirated human intestinal fluids support age-dependent oral drug absorption. *European journal of pharmaceutical sciences : official journal of the European Federation for Pharmaceutical Sciences*, 39(1-3):15–22, jan 2010.

[58] S. Clarysse, J. Brouwers, J. Tack, P. Annaert, and P. Augustijns. Intestinal drug solubility estimation based on simulated intestinal fluids: Comparison with solubility in human intestinal fluids. *European Journal of Pharmaceutical Sciences*, 43(4):260–269, 2011.

[59] T. Heikkilä, M. Karjalainen, K. Ojala, K. Partola, F. Lammert, P. Augustijns, A. Urtti, M. Yliperttula, L. Peltonen, and J. Hirvonen. Equilibrium drug solubility measurements in 96-well plates reveal similar drug solubilities in phosphate buffer pH 6.8 and human intestinal fluid. *International Journal of Pharmaceutics*, 405(1-2):132–136, 2011.

[60] E. Söderlind, E. Karlsson, A. Carlsson, R. Kong, A. Lenz, S. Lindborg, and J. J. Sheng. Simulating fasted human intestinal fluids: understanding the roles of lecithin and bile acids. *Molecular pharmaceutics*, 7(5):1498–507, oct 2010.

[61] Heumann Pharma GmbH & Co. Generica KG. Fachinformation - Carbamazepin 200/400 Heumann, 2014. URL https://www.fachinfo.de/suche/fi/006667. accessed: 11 Nov 2020.

[62] L. Bertilsson. Clinical pharmacokinetics of carbamazepine. *Clinical Pharmacokinetics*, 3:128–1473, 1978.

[63] J. Henshall, A. Galetin, A. Harrison, and J. B. Houston. Comparative analysis of CYP3A heteroactivation by steroid hormones and flavonoids in different in vitro systems and potential in vivo implications. *Drug Metabolism and Disposition*, 36(7):1332–1340, 2008.

[64] N. Cazali, A. Tran, J. M. Treluyer, E. Rey, P. Athis, J. Vincent, and G. Pons. Inhibitory effect of stiripentol on carbamazepine and saquinavir metabolism in human. *British Journal of Clinical Pharmacology*, 56(5):526, 2003.

[65] W. Huang, Y. S. Lin, D. J. McConn, J. C. Calamia, R. A. Totah, N. Isoherranen, M. Glodowski, and K. E. Thummel. Evidence of significant contribution from CYP3A5 to hepatic drug metabolism. *Drug Metabolism and Disposition*, 32(12):1434–1445, 2004.

[66] M. Shou, M. Hayashi, Y. Pan, Y. Xu, K. Morrissey, L. Xu, and G. L. Skiles. Modeling, prediction, and in vitro in vivo correlation of CYP3A4 induction. *Drug Metabolism and Disposition*, 36(11):2355–2370, 2008.

[67] D. F. McGinnity, G. Zhang, J. R. Kenny, G. A. Hamilton, S. Otmani, K. R. Stams, S. Haney, P. Brassil, D. M. Stresser, and R. J. Riley. Evaluation of multiple in vitro systems for assessment of CYP3A4 induction in drug discovery: human hepatocytes, pregnane X receptor reporter gene, and Fa2N-4 and HepaRG cells. *Drug metabolism and disposition: the biological fate of chemicals*, 37(6):1259–68, jun 2009.

[68] O. A. Fahmi, J. L. Raucy, E. Ponce, S. Hassanali, and J. M. Lasker. Utility of DPX2 cells for predicting CYP3A induction-mediated drug-drug interactions and associated structure-activity relationships. *Drug metabolism and disposition: the biological fate of chemicals*, 40(11):2204–11, nov 2012.

[69] J. G. Zhang, T. Ho, A. L. Callendrello, R. J. Clark, E. A. Santone, S. Kinsman, D. Xiao, L. G. Fox, H. J. Einolf, and D. M. Stresser. Evaluation of calibration curve-based approaches to predict clinical inducers and noninducers of CYP3A4 with plated human hepatocytes. *Drug Metabolism and Disposition*, 42(9):1379–1391, 2014.

[70] A. Moore, P. P. Chothe, H. Tsao, and N. Hariparsad. Evaluation of the interplay between uptake transport and CYP3A4 induction in micropatterned cocultured hepatocytes. *Drug Metabolism and Disposition*, 44(12):1910–1919, 2016.

[71] O. A. Fahmi, M. Kish, S. Boldt, and R. Scott Obach. Cytochrome P450 3A4 mRNA is a more reliable marker than CYP3A4 activity for detecting pregnane X receptor-activated induction of drug-metabolizing enzymes. *Drug Metabolism and Disposition*, 38(9):1605–1611, 2010.

[72] R. Zuo, F. Li, S. Parikh, L. Cao, K. L. Cooper, Y. Hong, J. Liu, R. A. Faris, D. Li, and H. Wang. Evaluation of a novel renewable hepatic cell model for prediction of clinical CYP3A4 induction using a correlation-based relative induction score approach. *Drug Metabolism and Disposition*, 45(2):198–207, 2017.

[73] J. G. Zhang, R. Patel, R. J. Clark, T. Ho, S. K. Trisdale, Y. Fang, and D. M. Stresser. Effect of Fifteen CYP3A4 in vitro Inducers on the Induction of Hepatocytes : A Trend Analysis. Poster presented at: 20th North American ISSX Meeting; 2015 18-22 Oct; Orlando Florida.

[74] O. A. Fahmi, M. Shebley, J. Palamanda, M. W. Sinz, D. Ramsden, H. J. Einolf, L. Chen, and H. Wang. Evaluation of CYP2B6 induction and prediction of clinical drug-drug interactions: Considerations from the IQ consortium induction working group - An industry perspective. *Drug Metabolism and Disposition*, 44(10):1720–1730, 2016.

[75] L. J. Dickmann and N. Isoherranen. Quantitative prediction of CYP2B6 induction by estradiol during pregnancy: Potential explanation for increased methadone clearance during pregnancy. *Drug Metabolism and Disposition*, 41(2):270–274, 2013.

[76] H. Lennernäs. Intestinal permeability and its relevance for absorption and elimination. *Xenobiotica*, 37(10-11):1015–1051, nov 2007.

[77] T. Rodgers, D. Leahy, and M. Rowland. Physiologically based pharmacokinetic modeling 1: predicting the tissue distribution of moderate-to-strong bases. *Journal of pharmaceutical sciences*, 94(6):1259–76, jun 2005.

[78] M. J. Taylor, S. Tanna, and T. Sahota. In vivo study of a polymeric glucose-sensitive insulin delivery system using a rat model. *Journal of pharmaceutical sciences*, 99(10): 4215–27, 2010.

[79] R. Kawai, M. Lemaire, J. L. Steimer, A. Bruelisauer, W. Niederberger, and M. Rowland. Physiologically based pharmacokinetic study on a cyclosporin derivative, SDZ IMM 125. *Journal of pharmacokinetics and biopharmaceutics*, 22(5):327–65, oct 1994.

[80] Drugbank. Metabolite 10,11-Epoxycarbamazepine, 2020. URL https://www.drugbank.ca/metabolites/DBMET00291. accessed: 14 Dec 2020.

[81] A. Gérardin, J. P. Dubois, J. Moppert, and L. Geller. Absolute bioavailability of carbamazepine after oral administration of a 2% syrup. *Epilepsia*, 31(3):334–8, 1990.

[82] U.S. Food and Drug Administration. SUSTIVA (efavirenz) label, 2011. URL https://www.accessdata.fda.gov/drugsatfda_docs/label/2011/020972s038lbl.pdf. accessed: 14 Dec 2020.

[83] B. A. Ward, J. C. Gorski, D. R. Jones, S. D. Hall, D. A. Flockhart, and Z. Desta. The cytochrome P450 2B6 (CYP2B6) is the main catalyst of efavirenz primary and secondary metabolism: Implication for HIV/AIDS therapy and utility of efavirenz as a substrate marker of CYP2B6 catalytic activity. *Journal of Pharmacology and Experimental Therapeutics*, 306(1):287–300, 2003.

[84] E. T. Ogburn, D. R. Jones, A. R. Masters, C. Xu, Y. Guo, and Z. Desta. Efavirenz primary and secondary metabolism in vitro and in vivo: Identification of novel metabolic pathways and cytochrome P450 2A6 as the principal catalyst of efavirenz 7-hydroxylation. *Drug Metabolism and Disposition*, 38(7):1218–1229, 2010.

[85] A. Ke, Z. Barter, K. Rowland-Yeo, and L. Almond. Towards a best practice approach in PBPK modeling: Case example of developing a unified efavirenz model accounting for induction of CYPs 3A4 and 2B6. *CPT: Pharmacometrics and Systems Pharmacology*, 5 (7):367–376, 2016.

[86] Z. Desta, R. S. Gammal, L. Gong, M. Whirl-Carrillo, A. H. Gaur, C. Sukasem, J. Hockings, A. Myers, M. Swart, R. F. Tyndale, C. Masimirembwa, O. F. Iwuchukwu, S. Chirwa, J. Lennox, A. Gaedigk, T. E. Klein, and D. W. Haas. Clinical Pharmacogenetics Implementation Consortium (CPIC) guideline for CYP2B6 and efavirenz-containing antiretroviral therapy. *Clinical Pharmacology and Therapeutics*, 106(4):726–733, 2019.

[87] S. Frechen, T. Wendel, and J. Solodenko. Building and evaluation of a PBPK model for efavirenz in healthy adults, 2020. URL https://github.com/Open-Systems-Pharmacology/OSP-PBPK-Model-Library/blob/v9.1/Efavirenz/efavirenz_evaluation_report.pdf. accessed: 13 Nov 2020.

[88] C. Xu, E. T. Ogburn, Y. Guo, and Z. Desta. Effects of the CYP2B6*6 allele on catalytic properties and inhibition of CYP2B6 in vitro: Implication for the mechanism of reduced efavirenz metabolism and other CYP2B6 substrates in vivo. *Drug Metabolism and Disposition*, 40(4):717–725, 2012.

[89] A. Derungs, M. Donzelli, B. Berger, C. Noppen, S. Krähenbühl, and M. Haschke. Effects of cytochrome P450 inhibition and induction on the phenotyping metrics of the Basel Cocktail: A randomized crossover study. *Clinical Pharmacokinetics*, 55(1):79–91, 2016.

[90] S. Mouly, K. S. Lown, D. Kornhauser, J. L. Joseph, W. D. Fiske, I. H. Benedek, and P. B. Watkins. Hepatic but not intestinal CYP3A4 displays dose-dependent induction by efavirenz in humans. *Clinical Pharmacology and Therapeutics*, 72(1):1–9, 2002.

[91] Ping Liu, G. Foster, R. R. Labadie, M. J. Gutierrez, and A. Sharma. Pharmacokinetic interaction between voriconazole and efavirenz at steady state in healthy male subjects. *Journal of Clinical Pharmacology*, 48(1):73–84, 2008.

[92] D. Y. Cho, J. H. Shen, S. M. Lemler, T. C. Skaar, L. Li, J. Blievernicht, U. M. Zanger, K. B. Kim, J. G. Shin, D. A. Flockhart, and Z. Desta. Rifampin enhances cytochrome P450 (CYP) 2B6-mediated efavirenz 8-hydroxylation in healthy volunteers. *Drug Metabolism and Pharmacokinetics*, 31(2):107–116, 2016.

[93] C. Xu, S. K. Quinney, Y. Guo, S. D. Hall, L. Li, and Z. Desta. CYP2B6 pharmacogenetics-based in vitro-in vivo extrapolation of efavirenz clearance by physiologically based pharmacokinetic modeling. *Drug Metabolism and Disposition*, 41(12):2004–2011, 2013.

[94] K. E. Dooley, J. G. Park, S. Swindells, R. Allen, D. W. Haas, Y. Cramer, F. Aweeka, I. Wiggins, A. Gupta, P. Lizak, S. Qasba, R. Van Heeswijk, and C. Flexner. Safety, tolerability, and pharmacokinetic interactions of the antituberculous agent TMC207 (Bedaquiline)

with efavirenz in healthy volunteers: AIDS clinical trials group study A5267. *Journal of Acquired Immune Deficiency Syndromes*, 59(5):455–462, 2012.

[95] B. Damle, R. LaBadie, P. Crownover, and P. Glue. Pharmacokinetic interactions of efavirenz and voriconazole in healthy volunteers. *British Journal of Clinical Pharmacology*, 65(4):523–530, 2008.

[96] V. Garg, G. Chandorkar, Y. Yang, N. Adda, L. Mcnair, K. Alves, F. Smith, and R. P. van Heeswijk. The effect of CYP3A inhibitors and inducers on the pharmacokinetics of telaprevir in healthy volunteers. *British Journal of Clinical Pharmacology*, 75(2):431–439, 2013.

[97] L. Huang, S. Parikh, P. J. Rosenthal, P. Lizak, F. Marzan, G. Dorsey, D. Havlir, and F. T. Aweeka. Concomitant efavirenz reduces pharmacokinetic exposure to the antimalarial drug artemether–lumefantrine in healthy volunteers. *JAIDS Journal of Acquired Immune Deficiency Syndromes*, 61(3):310–316, nov 2012.

[98] E. D. Kharasch, D. Whittington, D. Ensign, C. Hoffer, P. S. Bedynek, S. Campbell, K. Stubbert, A. Crafford, A. London, and T. Kim. Mechanism of efavirenz influence on methadone pharmacokinetics and pharmacodynamics. *Clinical Pharmacology and Therapeutics*, 91 (4):673–684, 2012.

[99] A. Kwara, K. T. Tashima, J. B. Dumond, P. Poethke, J. Kurpewski, A. D. Kashuba, M. H. Court, and D. J. Greenblatt. Modest but variable effect of rifampin on steady-state plasma pharmacokinetics of efavirenz in healthy African-American and Caucasian volunteers. *Antimicrobial Agents and Chemotherapy*, 55(7):3527–3533, 2011.

[100] C. D. Malvestutto, Q. Ma, G. D. Morse, J. A. Underberg, and J. A. Aberg. Lack of pharmacokinetic interactions between pitavastatin and efavirenz or darunavir/ritonavir. *Journal of Acquired Immune Deficiency Syndromes*, 67(4):390–396, 2014.

[101] G. H. Soon, P. Shen, E. L. Yong, P. Pham, C. Flexner, and L. Lee. Pharmacokinetics of darunavir at 900 milligrams and ritonavir at 100 milligrams once daily when coadministered with efavirenz at 600 milligrams once daily in healthy volunteers. *Antimicrobial Agents and Chemotherapy*, 54(7):2775–2780, 2010.

[102] Drugbank. Efavirenz, 2020. URL https://go.drugbank.com/drugs/DB00625. accessed: 14 Dec 2020.

[103] L. M. Almond, P. G. Hoggard, D. Edirisinghe, S. H. Khoo, and D. J. Back. Intracellular and plasma pharmacokinetics of efavirenz in HIV-infected individuals. *Journal of Antimicrobial Chemotherapy*, 56(4):738–744, 2005.

[104] O. Janneh, B. Chandler, R. Hartkoorn, W. S. Kwan, C. Jenkinson, S. Evans, D. J. Back, A. Owen, and S. H. Khoo. Intracellular accumulation of efavirenz and nevirapine is independent of P-glycoprotein activity in cultured CD4 T cells and primary human lymphocytes. *Journal of Antimicrobial Chemotherapy*, 64(5):1002–1007, nov 2009.

[105] R. Cristofoletti, A. Nair, B. Abrahamsson, D. Groot, S. Kopp, P. Langguth, J. E. Polli, V. P. Shah, and J. B. Dressman. Biowaiver monographs for immediate release solid oral dosage forms: Efavirenz. *Journal of Pharmaceutical Sciences*, 102(2):318–329, feb 2013.

[106] J. Gao, M. Hussain, R. Motheram, D. Gray, I. Benedek, W. Fiske, W. Doll, E. Sandefer, R. Page, and G. Digenis. Investigation of human pharmacoscintigraphic behavior of two tablets and a capsule formulation of a high dose, poorly water soluble/highly permeable drug (efavirenz). *Journal of Pharmaceutical Sciences*, 96(11):2970–2977, nov 2007.

[107] S. R. Rabel, M. B. Maurin, S. M. Rowe, and M. Hussain. Determination of the pKa and pH-solubility behavior of an ionizable cyclic carbamate, (S)-6-Chloro-4-(cyclopropylethynyl)-1,4-dihydro-4-(trifluoromethyl)-2H-3,1-benzoxazin-2-one (DMP 266). *Pharmaceutical Development and Technology*, 1(1):91–95, jan 1996.

[108] W. Schmitt. General approach for the calculation of tissue to plasma partition coefficients. *Toxicology in vitro : an international journal published in association with BIBRA*, 22(2): 457–67, mar 2008.

[109] E. J. Guest, L. Aarons, J. B. Houston, A. Rostami-Hodjegan, and A. Galetin. Critique of the two-fold measure of prediction success for ratios: application for the assessment of drug-drug interactions. *Drug metabolism and disposition: the biological fate of chemicals*, 39(2):170–3, feb 2011.

[110] S. Frechen and A. Dallmann. Building and evaluation of a PBPK model for erythromycin in healthy adults, 2020. URL https://github.com/Open-Systems-Pharmacology/OSP-PBPK-Model-Library/blob/v9.1/Erythromycin/Erythromycin_evaluation_report.pdf. accessed: 13 Nov 2020.

[111] S. Frechen. CYP3A4 DDI Qualification, 2020. URL https://github.com/Open-Systems-Pharmacology/OSP-Qualification-Reports/blob/v9.1/DDI_Qualification_CYP3A4/report.pdf. accessed: 14 Dec 2020.

[112] Drugbank. Erythromycin, 2020. URL https://go.drugbank.com/drugs/DB00199. accessed: 14 Dec 2020.

[113] E. Lien, J. Kuwahara, and R. Koda. Diffusion of drugs into prostatic fluid and milk. *Drug intelligence & Clinical Pharmacy*, 8(8):470–475, 1974.

[114] J. O. Capobianco and R. C. Goldman. Macrolide transport in Escherichia coli strains having normal and altered OmpC and/or OmpF porins. *International journal of antimicrobial agents*, 4(3):183–9, 1994.

[115] J. W. McFarland, C. M. Berger, S. A. Froshauer, S. F. Hayashi, S. J. Hecker, B. H. Jaynes, M. R. Jefson, B. J. Kamicker, C. A. Lipinski, K. M. Lundy, C. P. Reese, and C. B. Vu. Quantitative structure-activity relationships among macrolide antibacterial agents: In vitro and in vivo potency against Pasteurella multocida. *Journal of Medicinal Chemistry*, 40 (9):1340–1346, 1997.

[116] C. Hoffhine. Aqueous soluble salts of erythromycin. Issues September 4. Nr. 2,761,859, 1956. URL https://patents.google.com/patent/US2761859A/en. accessed: 14 Dec 2020.

[117] P. H. Jones, E. K. Rowley, A. L. Weiss, D. L. Bishop, and A. H. Chun. Insoluble erythromycin salts. *Journal of Pharmaceutical Sciences*, 58(3):337–339, 1969.

[118] P. K. Manna and S. K. Basu. Preparation and evaluation of erythromycin fumarate - A new derivative of erythromycin. *Drug Development and Industrial Pharmacy*, 24(9): 879–882, 1998.

[119] H. Sun, L. A. Frassetto, Y. Huang, and L. Z. Benet. Hepatic clearance, but not gut availability, of erythromycin is altered in patients with end-stage renal disease. *Clinical Pharmacology & Therapeutics*, 87(4):465–472, 2010.

[120] A. Iliopoulou, M. Aldhous, A. Johnston, and P. Turner. Pharmacokinetic interaction between theophylline and erythromycin. *British Journal of Clinical Pharmacology*, 14 (4):495–499, 1982.

[121] J. Barre, A. Mallat, J. Rosenbaum, L. Deforges, G. Houin, D. Dhumeaux, and J. Tillement. Pharmacokinetics of erythromycin in patients with severe cirrhosis. Respective influence of decreased serum binding and impaired liver metabolic capacity. *British Journal of Clinical Pharmacology*, 23(6):753–757, 1987.

[122] L. Xu, Y. Chen, Y. Pan, G. L. Skiles, and M. Shou. Prediction of human drug-drug interactions from time-dependent inactivation of CYP3A4 in primary hepatocytes using a population-based simulator. *Drug Metabolism and Disposition*, 37(12):2330–2339, 2009.

[123] R. W. Wang, D. J. Newton, T. D. Scheri, and A. Y. Lu. Human cytochrome P450 3A4-catalyzed testosterone 6 beta-hydroxylation and erythromycin N-demethylation. Competition during catalysis. *Drug metabolism and disposition: the biological fate of chemicals*, 25(4):502–7, 1997.

[124] R. J. Riley and D. Howbrook. In vitro analysis of the activity of the major human hepatic CYP enzyme (CYP3A4) using [N-methyl-14C]-erythromycin. *Journal of Pharmacological and Toxicological Methods*, 38(4):189–193, dec 1997.

[125] C. S. Lancaster, G. H. Bruun, C. J. Peer, T. S. Mikkelsen, T. J. Corydon, A. A. Gibson, S. Hu, S. J. Orwick, R. H. J. Mathijssen, W. D. Figg, S. D. Baker, and A. Sparreboom. OATP1B1 polymorphism as a determinant of erythromycin disposition. *Clinical Pharmacology & Therapeutics*, 92(5):642–650, nov 2012.

[126] T. Akiyoshi, M. Ito, S. Murase, M. Miyazaki, F. Peter Guengerich, K. Nakamura, K. Yamamoto, and H. Ohtani. Mechanism-based inhibition profiles of erythromycin and Cclarithromycin with cytochrome P450 3A4 genetic variants. *Drug Metabolism and Pharmacokinetics*, 28(5):411–415, 2013.

[127] A. Atkinson, J. R. Kenny, and K. Grime. Automated assessment of time-dependent inhibition of human cytochrome P450 enzymes using liquid chromatography-tandem mass spectrometry analysis. *Drug Metabolism and Disposition*, 33(11):1637–1647, nov 2005.

[128] S. Aueviriyavit, K. Kobayashi, and K. Chiba. Species differences in mechanism-based inactivation of CYP3A in humans, rats and mice. *Drug Metabolism and Pharmacokinetics*, 25(1):93–100, 2010.

[129] W. K. Chan and A. B. Delucchi. Resveratrol, a red wine constituent, is a mechanism-based inactivator of cytochrome P450 3A4. *Life Sciences*, 67(25):3103–3112, nov 2000.

[130] Y. Chen, L. Liu, M. Monshouwer, and A. J. Fretland. Determination of time-dependent inactivation of CYP3A4 in cryopreserved human hepatocytes and assessment of human drug-drug interactions. *Drug Metabolism and Disposition*, 39(11):2085–2092, nov 2011.

[131] Y. Ishikawa, T. Akiyoshi, A. Imaoka, and H. Ohtani. Inactivation kinetics and residual activity of CYP3A4 after treatment with erythromycin. *Biopharmaceutics & Drug Disposition*, 38(7):420–425, 2017.

[132] S. Kanamitsu, I. K, G. CE, C. Tyson, N. Shimada, and Y. Sugiyama. Prediction of in vivo interaction between triazolam and erythromycin based on in vitro studies using human liver microsomes and recombinant human CYP3A4. *Pharmaceutical Research*, 17(4): 419–426, 2000.

[133] K. Kozakai, Y. Yamada, M. Oshikata, T. Kawase, E. Suzuki, Y. Haramaki, and H. Taniguchi. Cocktail-substrate approach-based high-throughput assay for evaluation of direct and time-dependent inhibition of multiple cytochrome P450 isoforms. *Drug Metabolism and Pharmacokinetics*, 29(2):198–207, 2014.

[134] J. Mao, S. Tay, C. S. Khojasteh, Y. Chen, C. E. C. A. Hop, and J. R. Kenny. Evaluation of time dependent inhibition assays for marketed oncology drugs: Comparison of human hepatocytes and liver microsomes in the presence and absence of human plasma. *Pharmaceutical Research*, 33(5):1204–1219, 2016.

[135] D. J. McConn, Y. S. Lin, K. Allen, K. L. Kunze, and K. E. Thummel. Differences in the inhibition of cytochromes P450 3A4 and 3A5 by metabolite-inhibitor complex-forming drugs. *Drug Metabolism and Disposition*, 32(10):1083–1091, 2004.

[136] X. Zhang, D. R. Jones, and S. D. Hall. Prediction of the effect of erythromycin, diltiazem, and their metabolites, alone and in combination, on CYP3A4 inhibition. *Drug Metabolism and Disposition*, 37(1):150–160, 2009.

[137] S. Frechen and A. Dallmann. Building and evaluation of a PBPK model for alprazolam in healthy adults, 2020. URL https://github.com/Open-Systems-Pharmacology/OSP-PBPK-Model-Library/blob/v9.1/Alprazolam/Alprazolam_evaluation_report.pdf. accessed: 13 Nov 2020.

[138] Drugbank. Alprazolam, 2020. URL https://go.drugbank.com/drugs/DB00404. accessed: 14 Dec 2020.

[139] D. J. Greenblatt, L. L. Von Moltke, J. S. Harmatz, G. Chen, J. L. Weemhoff, C. Jen, C. J. Kelley, B. W. LeDuc, and M. A. Zinny. Time course of recovery of cytochrome P450 3A function after single doses of grapefruit juice. *Clinical Pharmacology and Therapeutics*, 74(2):121–29, 2003.

[140] S. G. Machatha and S. H. Yalkowsky. Estimation of the ethanol/water solubility profile from the octanol/water partition coefficient. *International Journal of Pharmaceutics*, 286 (1-2):111–115, nov 2004.

[141] T. Loftsson and D. Hreinsdôttir. Determination of aqueous solubility by heating and equilibration: A technical note. *AAPS PharmSciTech*, 7(1):E29–E32, mar 2006.

[142] F. S. Eberts, Y. Philopoulos, L. M. Reineke, and R. W. Vliek. Disposition of 14-C-alprazolam, a new anxiolytic-antidepressant, in man. *Pharmacologist*, 22(3):279, 1980.

[143] V. D. Schmith, B. Piraino, R. B. Smith, and P. D. Kroboth. Alprazolam in end-stage renal disease: I. Pharmacokinetics. *The Journal of Clinical Pharmacology*, 31(6):571–579, jun 1991.

[144] H. R. Ochs, D. J. Greenblatt, L. Labedzki, and R. B. Smith. Alprazolam kinetics in patients with renal insufficiency. *Journal of clinical psychopharmacology*, 6(5):292–294, 1986.

[145] M. Cho, T. Scahill, and J. Hester. Kinetics and equilibrium of the reversible alprazolam ring-opening reaction. *Journal of Pharmaceutical Sciences*, 72(4):356–362, apr 1983.

[146] G. G. Raymond and J. L. Born. An updated pKa listing of medicinal compounds. *Drug Intelligence & Clinical Pharmacy*, 20(9):683–686, sep 1986.

[147] K. R. Manchester, P. D. Maskell, and L. Waters. Experimental versus theoretical log D7.4, pKa and plasma protein binding values for benzodiazepines appearing as new psychoactive substances. *Drug Testing and Analysis*, 10(8):1258–1269, 2018.

[148] N. Hirota, K. Ito, T. Iwatsubo, C. E. Green, C. A. Tyson, N. Shimada, H. Suzuki, and Y. Sugiyama. In Vitro/in Vivo scaling of alprazolam metabolism by CYP3A4 and CYP3A5 in humans. *Biopharmaceutics & Drug Disposition*, 22(2):53–71, mar 2001.

[149] H. Furukori, M.D. Effect of carbamazepine on the single oral dose pharmacokinetics of alprazolam. *Neuropsychopharmacology*, 18(5):364–369, may 1998.

[150] J. Wojtyniak, H. Britz, D. Selzer, M. Schwab, and T. Lehr. Data Digitizing: Accurate and Precise Data Extraction for Quantitative Systems Pharmacology and Physiologically-Based Pharmacokinetic Modeling. *CPT: Pharmacometrics & Systems Pharmacology*, 9 (6):322–331, jun 2020.

[151] N. Tsamandouras, G. Dickinson, Y. Guo, S. Hall, A. Rostami-Hodjegan, A. Galetin, and L. Aarons. Development and application of a mechanistic pharmacokinetic model for simvastatin and its active metabolite simvastatin acid using an integrated population PBPK approach. *Pharmaceutical Research*, 32(6):1864–1883, jun 2015.

[152] H. Lennernäs and G. Fager. Pharmacodynamics and pharmacokinetics of the HMG-CoA reductase inhibitors. *Clinical Pharmacokinetics*, 32(5):403–425, may 1997.

[153] A. H. Rageh, N. N. Atia, and H. M. Abdel-Rahman. Lipophilicity estimation of statins as a decisive physicochemical parameter for their hepato-selectivity using reversed-phase thin layer chromatography. *Journal of Pharmaceutical and Biomedical Analysis*, 142: 7–14, aug 2017.

[154] Chemaxon. Chemicalize - instant cheminformatics solutions, 2020. URL https://chemicalize.com/. accessed: 14 Dec 2020.

[155] M. Rao, Y. Mandage, K. Thanki, and S. Bhise. Dissolution improvement of simvastatin by surface solid dispersion technology. *Dissolution Technologies*, 17(2):27–34, 2010.

[156] Merck Canada Inc. Product monograph: Zocor. 2006.

[157] S. Geboers, J. Stappaerts, J. Tack, P. Annaert, and P. Augustijns. In vitro and in vivo investigation of the gastrointestinal behavior of simvastatin. *International Journal of Pharmaceutics*, 510(1):296–303, aug 2016.

[158] M. Gertz, A. Harrison, J. B. Houston, and A. Galetin. Prediction of human intestinal first-pass metabolism of 25 CYP3A substrates from in vitro clearance and permeability data. *Drug Metabolism and Disposition*, 38(7):1147–1158, jul 2010.

[159] S. Vickers, C. A. Duncan, I. W. Chen, A. Rosegay, and D. E. Duggan. Metabolic disposition studies on simvastatin, a cholesterol-lowering prodrug. *Drug Metabolism and Disposition*, 18(2):138–145, 1990.

[160] T. Prueksaritanont, L. M. Gorham, B. Ma, L. Liu, X. Yu, J. J. Zhao, D. E. Slaughter, B. H. Arison, and K. P. Vyas. In vitro metabolism of simvastatin in humans [SBT] identification of metabolizing enzymes and effect of the drug on hepatic P450s. *Drug metabolism and disposition: the biological fate of chemicals*, 25(10):1191–1199, 1997.

[161] N. Le Goff, J. C. Koffel, S. Vandenschrieck, L. Jung, and G. Ubeaud. Comparison of in vitro hepatic models for the prediction of metabolic interaction between simvastatin and naringenin. *European Journal of Drug Metabolism and Pharmacokinetics*, 27(4): 233–241, dec 2002.

[162] M. Ishigam, M. Uchiyama, T. Kondo, H. Iwabuchi, S. I. Inoue, W. Takasaki, T. Ikeda, T. Komai, K. Ito, and Y. Sugiyama. Inhibition of in vitro metabolism of simvastatin by itraconazole in humans and prediction of in vivo drug-drug interactions. *Pharmaceutical Research*, 18(5):622–631, 2001.

[163] H. Lu, J. Zhu, Y. Zang, Y. Ze, and J. Qin. Cloning, high level expression of human paraoxonase-3 in Sf9 cells and pharmacological characterization of its product. *Biochemical Pharmacology*, 70(7):1019–1025, oct 2005.

[164] D. A. Taha, C. H. D. Moor, D. A. Barrett, J. B. Lee, R. D. Gandhi, C. W. Hoo, and P. Gershkovich. The role of acid-base imbalance in statin-induced myotoxicity. *Translational research: the journal of laboratory and clinical medicine,*, 174:140–160, 2016.

[165] A. Á. Lueje, C. Valenzuela, J. A. Squella, and N.-V. Joaquin Luis. Stability study of simvastatin under hydrolytic conditions assessed by liquid chromatography. *Journal of AOAC International*, 88(6):1631–1636, 2005.

[166] T. Prueksaritanont, Y. Qiu, L. Mu, K. Michel, J. Brunner, K. M. Richards, and J. H. Lin. Interconversion pharmacokinetics of simvastatin and its hydroxy acid in dogs: Effects of gemfibrozil. *Pharmaceutical Research*, 22(7):1101–1109, jul 2005.

[167] L. Huang, Y. Wang, and S. Grimm. ATP-dependent transport of rosuvastatin in membrane vesicles expressing brast cancer resistance protein. *Drug Metabolism and Disposition*, 34(5):738–742, may 2006.

[168] J. W. Deng, J.-H. Shon, H.-J. Shin, S.-J. Park, C.-W. Yeo, H.-H. Zhou, I.-S. Song, and J.-G. Shin. Effect of silymarin supplement on the pharmacokinetics of rosuvastatin. *Pharmaceutical Research*, 25(8):1807–1814, aug 2008.

[169] M. Hirano, K. Maeda, S. Matsushima, Y. Nozaki, H. Kusuhara, and Y. Sugiyama. Involvement of BCRP (ABCG2) in the biliary excretion of pitavastatin. *Molecular Pharmacology*, 68(3):800–807, sep 2005.

[170] M. Afrouzian, R. Al-Lahham, S. Patrikeeva, M. Xu, V. Fokina, W. G. Fischer, S. Z. Abdel-Rahman, M. Costantine, M. S. Ahmed, and T. Nanovskaya. Role of the efflux transporters BCRP and MRP1 in human placental bio-disposition of pravastatin. *Biochemical Pharmacology*, 156:467–478, oct 2018.

[171] S. Kitamura, K. Maeda, Y. Wang, and Y. Sugiyama. Involvement of multiple transporters in the hepatobiliary transport of rosuvastatin. *Drug Metabolism and Disposition*, 36(10): 2014–2023, oct 2008.

[172] A. Vildhede, A. Mateus, E. K. Khan, Y. Lai, M. Karlgren, P. Artursson, and M. C. Kjellsson. Mechanistic modeling of pitavastatin disposition in sandwich-cultured human hepatocytes: A proteomics-informed bottom-up approach. *Drug Metabolism and Disposition*, 44(4):505–516, feb 2016.

[173] B. M. VandenBrink, R. S. Foti, D. A. Rock, L. C. Wienkers, and J. L. Wahlstrom. Evaluation of CYP2C8 inhibition in vitro: Utility of montelukast as a selective CYP2C8 probe substrate. *Drug Metabolism and Disposition*, 39(9):1546–1554, sep 2011.

[174] R. S. Foti, D. A. Rock, L. C. Wienkers, and J. L. Wahlstrom. Selection of alternative CYP3A4 probe substrates for clinical drug interaction studies using in vitro data and in vivo simulation. *Drug Metabolism and Disposition*, 38(6):981–987, jun 2010.

[175] C. S. Cook, L. M. Berry, and E. Burton. Prediction of in vivo drug interactions with eplerenone in man from in vitro metabolic inhibition data. *Xenobiotica*, 34(3):215–228, mar 2004.

[176] M. Ishigami, T. Honda, W. Takasaki, T. Ikeda, T. Komai, K. Ito, and Y. Sugiyama. A comparison of the effects of 3-hydroxy-3-methylglutaryl-coenzyme a (HMG-CoA) reductase

inhibitors on the CYP3A4-dependent oxidation of mexazolam in vitro. *Drug metabolism and disposition: the biological fate of chemicals*, 29(3):282–8, mar 2001.

[177] H. Fujino, I. Yamada, S. Shimada, T. Nagao, and M. Yoneda. Metabolic fate of pitavastatin (NK-104), a new inhibitor of 3-hydroxy-3-methyllutaryl coenzyme A reductase. *Arzneimittelforschung*, 52(10):745–753, dec 2011.

[178] M. G. Soars, P. Barton, M. Ismair, R. Jupp, and R. J. Riley. The development, characterization, and application of an OATP1B1 inhibition assay in drug discovery. *Drug Metabolism and Disposition*, 40(8):1641–1648, aug 2012.

[179] C. Chen, R. J. Mireles, S. D. Campbell, J. Lin, J. B. Mills, J. J. Xu, and T. A. Smolarek. Differential interaction of 3-hydroxy-3-methylglutaryl-CoA reductase inhibitors with ABCB1, ABCC2 and OATP1B1. *Drug Metabolism and Disposition*, 33(4):537–546, apr 2005.

[180] E. J. Wang, C. N. Casciano, R. P. Clement, and W. W. Johnson. HMG-CoA reductase inhibitors (statins) characterized as direct inhibitors of P-glycoprotein. *Pharmaceutical Research1*, 18(6):800–806, 2001.

[181] M. Werner, B. Atil, E. Sieczkowski, P. Chiba, and M. Hohenegger. Simvastatin-induced compartmentalisation of doxorubicin sharpens up nuclear topoisomerase II inhibition in human rhabdomyosarcoma cells. *Naunyn-Schmiedeberg's Archives of Pharmacology*, 386(7):605–617, jul 2013.

[182] E.-j. Wang, C. N. Casciano, R. P. Clement, and W. W. Johnson. Active transport of fluorescent P-glycoprotein substrates: Evaluation as markers and interaction with inhibitors. *Biochemical and Biophysical Research Communications*, 289(2):580–585, nov 2001.

[183] H. Sugimoto, S.-I. Matsumoto, M. Tachibana, S.-I. Niwa, H. Hirabayashi, N. Amano, and T. Moriwaki. Establishment of in vitro P-glycoprotein inhibition assay and its exclusion criteria to assess the risk Of drug–drug interaction at the drug discovery stage. *Journal of Pharmaceutical Sciences*, 100(9):4013–4023, sep 2011.

[184] A. Poirier, A.-C. Cascais, U. Bader, R. Portmann, M.-E. Brun, I. Walter, A. Hillebrecht, M. Ullah, and C. Funk. Calibration of in vitro multidrug resistance protein 1 substrate and inhibition assays as a basis to support the prediction of clinically relevant interactions in vivo. *Drug Metabolism and Disposition*, 42(9):1411–1422, sep 2014.

[185] J. P. Keogh and J. R. Kunta. Development, validation and utility of an in vitro technique for assessment of potential clinical drug–drug interactions involving P-glycoprotein. *European Journal of Pharmaceutical Sciences*, 27(5):543–554, apr 2006.

[186] T. Sakaeda, H. Fujino, C. Komoto, M. Kakumoto, J. S. Jin, K. Iwaki, K. Nishiguchi, T. Nakamura, N. Okamura, and K. Okumura. Effects of acid and lactone forms of eight HMG-CoA reductase inhibitors on CYP-mediated metabolism and MDR1-mediated transport. *Pharmaceutical Research*, 23(3):506–512, 2006.

[187] L. M. Berezhkovskiy. Volume of distribution at steady state for a linear pharmacokinetic system with peripheral elimination. *Journal of pharmaceutical sciences*, 93(6):1628–40, jun 2004.

[188] Drugbank. Simvastatin hydroxy acid, 2020. URL https://go.drugbank.com/metabolites/DBMET00388. accessed: 14 Dec 2020.

[189] C. W. Fong. Statins in therapy: Cellular transport, side effects, drug-drug interactions and cytotoxicity -the unrecognized role of lactones. 2016.

[190] M. Yoshinari, K. Matsuzaka, S. Hashimoto, K. Ishihara, T. Inoue, Y. Oda, T. Ide, and T. Tanaka. Controlled release of simvastatin acid using cyclodextrin inclusion system. *Dental Materials Journal*, 26(3):451–456, 2007.

[191] T. Prueksaritanont, B. Ma, and N. Yu. The human hepatic metabolism of simvastatin hydroxy acid is mediated primarily by CYP3A, and not CYP2D6. *British Journal of Clinical Pharmacology*, 56(1):120–124, jul 2003.

[192] A. Tornio, M. K. Pasanen, J. Laitila, P. J. Neuvonen, and J. T. Backman. Comparison of 3-hydroxy-3-methylglutaryl Coenzyme A (HMG-CoA) reductase inhibitors (statins) as inhibitors of cytochrome P450 2C8. *Basic & Clinical Pharmacology & Toxicology*, 97(2): 104–108, aug 2005.

[193] T. Prueksaritanont, R. Subramanian, X. Fang, B. Ma, Y. Qiu, J. H. Lin, P. G. Pearson, and T. A. Baillie. Glucuronidation of statins in animals and humans: A novel mechanism of statin lactonization. *Drug Metabolism and Disposition*, 30(5):505–512, may 2002.

[194] H. Huang. *Characterization of in vitro systems for transporter studies*. Phd thesis, Uppsala University, 2010.

[195] H. Schelleman, X. Han, C. M. Brensinger, S. K. Quinney, W. B. Bilker, D. A. Flockhart, L. Li, and S. Hennessy. Pharmacoepidemiologic and in vitro evaluation of potential drug-drug interactions of sulfonylureas with fibrates and statins. *British Journal of Clinical Pharmacology*, 78(3):639–648, sep 2014.

[196] M. Ucar, M. Neuvonen, H. Luurila, R. Dahlqvist, P. J. Neuvonen, and T. Mjörndal. Carbamazepine markedly reduces serum concentrations of simvastatin and simvastatin acid. *European journal of clinical pharmacology*, 59(12):879–82, feb 2004.

[197] F. Z. Marok, L. Fuhr, N. Hanke, D. Selzer, and T. Lehr. Physiologically Based Pharmacokinetic Modeling of Bupropion and its Metabolites in a CYP2B6 Drug-Drug-Gene Interaction Network. *Pharmaceutics*, in submission, 2020.

[198] P. Muralidhar, E. Bhargav, and B. Srinath. Formulation and optimization of bupropion HCl in microspongesby 2^3 factorial design. *International Journal of Pharmaceutical Sciences and Research*, 8(3):1134–1144, 2017.

[199] M. J. Reese, R. M. Wurm, K. T. Muir, G. T. Generaux, L. St. John-Williams, and D. J. Mcconn. An in vitro mechanistic study to elucidate the desipramine/bupropion clinical drug-drug interaction. *Drug Metabolism and Disposition*, 36(7):1198–1201, jul 2008.

[200] T. Takayanagi, D. Itoh, and H. Mizugushi. Analysis of acid dissociation equilibrium of bupropion by capillary zone electrophoresis after the heat-degradation. *Chromatography*, 37(3):105–109, 2016.

[201] Y. Chen, J. Mao, and C. E. Hop. Physiologically based pharmacokinetic modeling to predict drug-drug interactions involving inhibitory metabolite: A case study of amiodarone. *Drug Metabolism and Disposition*, 43(2):182–189, 2015.

[202] X. Wang, D. R. Abdelrahman, O. L. Zharikova, S. L. Patrikeeva, G. D. Hankins, M. S. Ahmed, and T. N. Nanovskaya. Bupropion metabolism by human placenta. *Biochemical Pharmacology*, 79(11):1684–1690, jun 2010.

[203] F. I. Carroll, B. E. Blough, P. Abraham, A. C. Mills, J. A. Holleman, S. A. Wolckenhauer, A. M. Decker, A. Landavazo, K. T. McElroy, H. A. Navarro, M. B. Gatch, and M. J. Forster. Synthesis and biological evaluation of bupropion analogues as potential pharmacotherapies for cocaine addiction. *Journal of Medicinal Chemistry*, 52(21):6768–6781, 2009.

[204] PubChem Hydroxybupropion (27.11.2020). URL https://pubchem.ncbi.nlm.nih.gov/compound/446.

[205] B. T. Gufford, J. B. L. Lu, I. F. Metzger, D. R. Jones, and Z. Desta. Stereoselective glucuronidation of bupropion metabolites in vitro and in vivo. *Drug Metabolism and Disposition*, 44(4):544–553, 2016.

[206] C. Xue, X. Zhang, and W. Cai. Prediction of drug-drug interactions with bupropion and its metabolites as CYP2D6 inhibitors using a physiologically-based pharmacokinetic model. *Pharmaceutics*, 10(1):1, dec 2017.

[207] T. A. Ketter, J. B. Jenkins, D. H. Schroeder, P. J. Pazzaglia, L. B. Marangell, M. S. George, A. M. Callahan, M. L. Hinton, J. Chao, and R. M. Post. Carbamazepine but not valproate induces bupropion metabolism. *Journal of clinical psychopharmacology*, 15(5):327–33, oct 1995.

[208] G. Mikus, T. Heinrich, J. Bödigheimer, C. Röder, A. K. Matthee, J. Weiss, J. Burhenne, and W. E. Haefeli. Semisimultaneous midazolam administration to evaluate the time course of CYP3A activation by a single oral dose of efavirenz. *Journal of Clinical Pharmacology*, 57(7):899–905, 2017.

[209] S. Frechen and A. Dallmann. Building and evaluation of a PBPK model for midazolam in healthy adults, 2020. URL https://github.com/Open-Systems-Pharmacology/OSP-PBPK-Model-Library/blob/v9.1/Erythromycin/Erythromycin_evaluation_report.pdf. accessed: 14 Dec 2020.

[210] N. Hanke, S. Frechen, D. Moj, H. Britz, T. Eissing, T. Wendl, and T. Lehr. PBPK models for CYP3A4 and P-gp DDI prediction: A modeling network of rifampicin, itraconazole, clarithromycin, midazolam, alfentanil, and digoxin. *CPT: Pharmacometrics & Systems Pharmacology*, 7(10):647–659, oct 2018.

[211] Drugbank. Midazolam, 2020. URL https://go.drugbank.com/drugs/DB00683. accessed: 14 Dec 2020.

[212] Y. Wang, D. Chen, G. Hartmann, C. R. Cho, and K. Menzel. PBPK modeling strategy for predicting complex drug interactions of letermovir as a perpetrator in support of product labeling. *Clinical Pharmacology & Therapeutics*, 105(2):515–523, feb 2019.

[213] C. Dagenais, A. Avdeef, O. Tsinman, A. Dudley, and R. Beliveau. P-glycoprotein deficient mouse in situ blood–brain barrier permeability and its prediction using an in combo PAMPA model. *European Journal of Pharmaceutical Sciences*, 38(2):121–137, sep 2009.

[214] M. Bolger. Physiologically-based pharmacokinetics (PBPK) linked to pharmacodynamics: In silico and in vitro parameterization; presented at Globalization of Pharmaceutics Education Network (GPEN), 2006, Kansas.

[215] T. Rodgers and M. Rowland. Physiologically based pharmacokinetic modelling 2: predicting the tissue distribution of acids, very weak bases, neutrals and zwitterions. *Journal of pharmaceutical sciences*, 95(6):1238–57, jun 2006.

[216] A. T. Heikkinen, G. Baneyx, A. Caruso, and N. Parrott. Application of PBPK modeling to predict human intestinal metabolism of CYP3A substrates – An evaluation and case study using GastroPlus™. *European Journal of Pharmaceutical Sciences*, 47(2):375–386, sep 2012.

[217] P. F. Wang, A. Neiner, and E. D. Kharasch. Efavirenz metabolism: Influence of polymorphic CYP2B6 variants and stereochemistry. *Drug Metabolism and Disposition*, 47 (10):1195–1205, 2019.

[218] K. S. Lown, K. E. Thummel, P. E. Benedict, D. D. Shen, D. K. Turgeon, S. Berent, and P. B. Watkins. The erythromycin breath test predicts the clearance of midazolam. *Clinical Pharmacology & Therapeutics*, 57(1):16–24, jan 1995.

[219] S. Björkman, D. Wada, B. Berling, and G. Benoni. Prediction of the disposition of midazolam in surgical patients by a physiologically based pharmacokinetic model. *Journal of Pharmaceutical Sciences*, 90(9):1226–1241, sep 2001.

[220] A. Galetin, C. Brown, D. Hallifax, K. Ito, and J. B. Houston. Utility of recombinant enzyme kinetics in prediction of human clearance: Impact of variability, CYP3A5, and CYP2C19 on CYP3A4 probe substrates. *Drug Metabolism and Disposition*, 32(12):1411–1420, dec 2004.

[221] K. Ito, K. Ogihara, S.-i. Kanamitsu, and T. Itoh. Prediction of the in vivo interaction between midazolam and macrolides based on in vitro studies using human liver microsomes. *Drug Metabolism and Disposition*, 31(7):945–954, jul 2003.

[222] K. C. Patki, L. L. von Moltke, and D. J. Greenblatt. In vitro metabolism of midazolam, triazolam, nifedipine, and testosterone by human liver microsomes and recombinant cytochromes P440: Role of CYP3A4 and CYP3A5. *Drug Metabolism and Disposition*, 31(7): 938–944, jul 2003.

[223] S. Klieber, S. Hugla, R. Ngo, C. Arabeyre-Fabre, V. Meunier, F. Sadoun, O. Fedeli, M. Rival, M. Bourrie, F. Guillou, P. Maurel, and G. Fabre. Contribution of the N - glucuronidation pathway to the overall in vitro metabolic clearance of midazolam in humans. *Drug Metabolism and Disposition*, 36(5):851–862, may 2008.

[224] A. Buhr, R. Baur, and E. Sigel. Subtle changes in residue 77 of the γ subunit of $\alpha 1\beta 2\gamma 2$ GABA A receptors drastically alter the affinity for ligands of the benzodiazepine binding site. *Journal of Biological Chemistry*, 272(18):11799–11804, may 1997.

[225] S. Katzenmaier, C. Markert, and G. Mikus. Proposal of a new limited sampling strategy to predict CYP3A activity using a partial AUC of midazolam. *European Journal of Clinical Pharmacology*, 66(11):1137–1141, 2010.

[226] S. Frechen, A. Dallmann, and J. Solodenko. Building and evaluation of a PBPK model for alfentanil in healthy adults, 2020. URL https://github.com/Open-Systems-Pharmacology/OSP-PBPK-Model-Library/blob/v9.1/Erythromycin/Erythromycin_evaluation_report.pdf. accessed: 14 Dec 2020.

[227] Drugbank. Alfentanil, 2020. URL https://go.drugbank.com/drugs/DB00802. accessed: 14 Dec 2020.

[228] G. Baneyx, N. Parrott, C. Meille, A. Iliadis, and T. Lavé. Physiologically based pharmacokinetic modeling of CYP3A4 induction by rifampicin in human: influence of time between substrate and inducer administration. *European journal of pharmaceutical sciences: official journal of the European Federation for Pharmaceutical Sciences*, 56:1–15, 2014.

[229] R. Jansson, U. Bredberg, and M. Ashton. Prediction of drug tissue to plasma concentration ratios using a measured volume of distribution in combination with lipophilicity. *Journal of Pharmaceutical Sciences*, 97(6):2324–2339, jun 2008.

[230] A. N. Edginton and S. Willmann. Physiology-based simulations of a pathological condition. *Clinical Pharmacokinetics*, 47(11):743–752, 2008.

[231] L. M. Almond, S. Mukadam, I. Gardner, K. Okialda, S. Wong, O. Hatley, S. Tay, K. Rowland-Yeo, M. Jamei, A. Rostami-Hodjegan, and J. R. Kenny. Prediction of drug-drug interactions arising from CYP3A induction using a physiologically based dynamic model. *Drug metabolism and disposition: the biological fate of chemicals*, 44(6):821–32, 2016.

[232] S. M. Robertson, F. Maldarelli, V. Natarajan, E. Formentini, R. M. Alfaro, and S. R. Penzak. Efavirenz induces CYP2B6-mediated hydroxylation of bupropion in healthy subjects. *Journal of Acquired Immune Deficiency Syndromes*, 49(5):513–519, 2008.

[233] D. S. Wishart, C. Knox, A. C. Guo, S. Shrivastava, M. Hassanali, P. Stothard, Z. Chang, and J. Woolsey. DrugBank: a comprehensive resource for in silico drug discovery and exploration. *Nucleic Acids Research*, 34(Supplement 1):D668–D672, 01 2006.

[234] G. Boman and V. A. Ringberger. Binding of rifampicin by human plasma proteins. *European journal of clinical pharmacology*, 7(5):369–73, 1974.

[235] I. E. Templeton, J. B. Houston, and A. Galetin. Predictive utility of in vitro rifampin induction data generated in fresh and cryopreserved human hepatocytes, Fa2N-4, and HepaRG cells. *Drug metabolism and disposition: the biological fate of chemicals*, 39 (10):1921–9, 2011.

[236] Merck Research Laboratories. *The Merck Index 14th edition: Rifampin*. Merck & Co., Inc., Whitehouse Station, NJ, USA, 2006.

[237] U. Loos, E. Musch, J. C. Jensen, G. Mikus, H. K. Schwabe, and M. Eichelbaum. Pharmacokinetics of oral and intravenous rifampicin during chronic administration. *Klinische Wochenschrift*, 63(23):1205–11, 1985.

[238] R. G. Tirona, B. F. Leake, A. W. Wolkoff, and R. B. Kim. Human organic anion transporting polypeptide-C (SLC21A6) is a major determinant of rifampin-mediated pregnane X receptor activation. *The Journal of pharmacology and experimental therapeutics*, 304 (1):223–8, jan 2003.

[239] A. Nakajima, T. Fukami, Y. Kobayashi, A. Watanabe, M. Nakajima, and T. Yokoi. Human arylacetamide deacetylase is responsible for deacetylation of rifamycins: rifampicin, rifabutin, and rifapentine. *Biochemical pharmacology*, 82(11):1747–56, dec 2011.

[240] A. Collett, J. Tanianis-Hughes, D. Hallifax, and G. Warhurst. Predicting P-glycoprotein effects on oral absorption: correlation of transport in Caco-2 with drug pharmacokinetics in wild-type and mdr1a(-/-) mice in vivo. *Pharmaceutical research*, 21(5):819–26, may 2004.

[241] B. Greiner, M. Eichelbaum, P. Fritz, H. P. Kreichgauer, O. von Richter, J. Zundler, and H. K. Kroemer. The role of intestinal P-glycoprotein in the interaction of digoxin and rifampin. *The Journal of clinical investigation*, 104(2):147–53, 1999.

[242] A. Ramamoorthy, Y. Liu, S. Philips, Z. Desta, H. Lin, C. Goswami, A. Gaedigk, L. Li, D. A. Flockhart, and T. C. Skaar. Regulation of microRNA expression by rifampin in human hepatocytes. *Drug Metabolism and Disposition*, 41(10):1763–1768, 2013.

[243] M. Hirano, K. Maeda, Y. Shitara, and Y. Sugiyama. Drug-drug interaction between pitavastatin and various drugs via OATP1B1. *Drug metabolism and disposition: the biological fate of chemicals*, 34(7):1229–36, jul 2006.

[244] M. L. Reitman, X. Chu, X. Cai, J. Yabut, R. Venkatasubramanian, S. Zajic, J. A. Stone, Y. Ding, R. Witter, C. Gibson, K. Roupe, R. Evers, J. A. Wagner, and A. Stoch. Rifampin's acute inhibitory and chronic inductive drug interactions: experimental and model-based approaches to drug-drug interaction trial design. *Clinical pharmacology and therapeutics*, 89(2):234–42, 2011.

[245] L. I. Kajosaari, J. Laitila, P. J. Neuvonen, and J. T. Backman. Metabolism of repaglinide by CYP2C8 and CYP3A4 in vitro: effect of fibrates and rifampicin. *Basic & clinical pharmacology & toxicology*, 97(4):249–56, 2005.

[246] Y. Shimokawa, N. Yoda, S. Kondo, Y. Yamamura, Y. Takiguchi, and K. Umehara. Inhibitory Potential of Twenty Five Anti-tuberculosis Drugs on CYP Activities in Human Liver Microsomes. *Biological & Pharmaceutical Bulletin*, 38(9):1425–1429, 2015.

[247] X. Li, S. Frechen, D. Moj, T. Lehr, M. Taubert, C. hsuan Hsin, G. Mikus, P. J. Neuvonen, K. T. Olkkola, T. I. Saari, and U. Fuhr. A physiologically based pharmacokinetic model of voriconazole integrating time-dependent inhibition of CYP3A4, genetic polymorphisms of CYP2C19 and predictions of drug–drug interactions. *Clinical Pharmacokinetics*, 59(6): 781–808, 2020.

[248] Drugbank. Voriconazole, 2020. URL https://go.drugbank.com/drugs/DB00582. accessed: 14 Dec 2020.

[249] Pfizer. Voriconazole for IV infusion safety data sheet, 2018. URL https://pfe-pfizercom-prod.s3.amazonaws.com/products/material_safety_data/voriconazole_IV_infusion_22-mar-2018.pdf. accessed: 14 Dec 2020.

[250] F. Qi, L. Zhu, N. Li, T. Ge, G. Xu, and S. Liao. Influence of different proton pump inhibitors on the pharmacokinetics of voriconazole. *International Journal of Antimicrobial Agents*, 49(4):403–409, apr 2017.

[251] N. R. Zane and D. R. Thakker. A physiologically based pharmacokinetic model for voriconazole disposition predicts intestinal first-pass metabolism in children. *Clinical Pharmacokinetics*, 53(12):1171–1182, dec 2014.

[252] B. Damle, M. V. Varma, and N. Wood. Pharmacokinetics of voriconazole administered concomitantly with fluconazole and population-based simulation for sequential use. *Antimicrobial Agents and Chemotherapy*, 55(11):5172–5177, nov 2011.

[253] Pfizer Canada ULC. Product monograph VFEND voriconazole. Submission Control No: 237769, 2020. URL https://www.pfizer.ca/sites/default/files/202007/Vfend_PM_EN_237769_30June2020.pdf. accessed: 14 Dec 2020.

[254] Scientific Discussion-VFEND Procedure No. EMEA/H/ C/387/X/09. 2004.

[255] U.S. Food and Drug Administration. Pfizer Label: voriconazole for injection, tablets, oral suspension: LAB-0271-12. 2005.

[256] G. Wang, H.-P. Lei, Z. Li, Z.-R. Tan, D. Guo, L. Fan, Y. Chen, D.-L. Hu, D. Wang, and H.-H. Zhou. The CYP2C19 ultra-rapid metabolizer genotype influences the pharmacokinetics of voriconazole in healthy male volunteers. *European Journal of Clinical Pharmacology*, 65(3):281–285, mar 2009.

[257] N. Murayama, N. Imai, T. Nakane, M. Shimizu, and H. Yamazaki. Roles of CYP3A4 and CYP2C19 in methyl hydroxylated and N-oxidized metabolite formation from voriconazole, a new anti-fungal agent, in human liver microsomes. *Biochemical Pharmacology*, 73(12): 2020–2026, jun 2007.

[258] R. Hyland, B. C. Jones, and D. A. Smith. Identification of the cytochrome P450 enzymes involved in the N-oxidation of voriconazole. *Drug Metabolism and Disposition*, 31(5): 540–547, may 2003.

[259] P. Poulin, K. Schoenlein, and F. Theil. Prediction of adipose tissue: Plasma partition coefficients for structurally unrelated drugs. *Journal of Pharmaceutical Sciences*, 90(4): 436–447, apr 2001.

[260] P. Poulin and F. P. Theil. A priori prediction of tissue:plasma partition coefficients of drugs to facilitate the use of physiologically-based pharmacokinetic models in drug discovery. *Journal of pharmaceutical sciences*, 89(1):16–35, jan 2000.

[261] P. Poulin and F.-P. Theil. Prediction of pharmacokinetics prior to in vivo studies. 1. Mechanism-based prediction of volume of distribution. *Journal of pharmaceutical sciences*, 91(1):129–56, jan 2002.

[262] P. Poulin and F.-P. Theil. Prediction of pharmacokinetics prior to in vivo studies. II. Generic physiologically based pharmacokinetic models of drug disposition. *Journal of pharmaceutical sciences*, 91(5):1358–70, may 2002.

[263] M. Meyer, S. Schneckener, B. Ludewig, L. Kuepfer, and J. Lippert. Using expression data for quantification of active processes in physiologically based pharmacokinetic modeling. *Drug metabolism and disposition: the biological fate of chemicals*, 40(5):892–901, may 2012.

[264] P. Shi, M. Liao, B.-C. Chuang, R. Griffin, J. Shi, M. Hyer, J. K. Fallon, P. C. Smith, C. Li, and C. Q. Xia. Efflux transporter breast cancer resistance protein dominantly expresses on the membrane of red blood cells, hinders partitioning of its substrates into the cells, and alters drug–drug interaction profiles. *Xenobiotica*, 48(11):1173–1183, 2018.

[265] N. Kolesnikov, E. Hastings, M. Keays, O. Melnichuk, Y. A. Tang, E. Williams, M. Dylag, N. Kurbatova, M. Brandizi, T. Burdett, K. Megy, E. Pilicheva, G. Rustici, A. Tikhonov, H. Parkinson, R. Petryszak, U. Sarkans, and A. Brazma. ArrayExpress update-simplifying data submissions. *Nucleic Acids Research*, 43(D1):D1113–D1116, 2015.

[266] M. Nishimura and S. Naito. Tissue-specific mRNA expression profiles of human phase I metabolizing enzymes except for cytochrome P450 and phase II metabolizing enzymes. *Drug metabolism and pharmacokinetics*, 21(5):357–74, 2006.

[267] A. D. Rodrigues. Integrated cytochrome P450 reaction phenotyping: attempting to bridge the gap between cDNA-expressed cytochromes P450 and native human liver microsomes. *Biochemical pharmacology*, 57(5):465–80, 1999.

[268] M. Nishimura, H. Yaguti, H. Yoshitsugu, S. Naito, and T. Satoh. Tissue distribution of mRNA expression of human cytochrome P450 isoforms assessed by high-sensitivity real-time reverse transcription PCR. *Journal of the Pharmaceutical Society of Japan*, 123(5):369–75, may 2003.

[269] K. Rowland Yeo, R. L. Walsky, M. Jamei, A. Rostami-Hodjegan, and G. T. Tucker. Prediction of time-dependent CYP3A4 drug-drug interactions by physiologically based pharmacokinetic modelling: Impact of inactivation parameters and enzyme turnover. *European Journal of Pharmaceutical Sciences*, 43(3):160–73, 2011.

[270] G. Margaillan, M. Rouleau, K. Klein, J. K. Fallon, P. Caron, L. Villeneuve, P. C. Smith, U. M. Zanger, and C. Guillemette. Multiplexed targeted quantitative proteomics predicts hepatic glucuronidation potential. *Drug Metabolism and Disposition*, 43:1331–5, 2015.

[271] National Center for Biotechnology Information (NCBI). Expressed Sequence Tags (EST) from UniGene. 2019.

[272] M. Nishimura and S. Naito. Tissue-specific mRNA expression profiles of human ATP-binding cassette and solute carrier transporter superfamilies. *Drug metabolism and pharmacokinetics*, 20(6):452–77, 2005.
